# A spirocyclic backbone accesses new conformational space in an extended, dipole-stabilized foldamer

**DOI:** 10.1038/s42004-023-00868-8

**Published:** 2023-04-17

**Authors:** William Edward Roe, Toyah Mary Catherine Warnock, Peter Clarke Knipe

**Affiliations:** School of Chemistry and Chemical Engineering, Queen’s University Belfast, David Keir Building, Belfast, BT9 5AG UK

**Keywords:** Asymmetric synthesis, NMR spectroscopy, Polymer synthesis

## Abstract

Most aromatic foldamers adopt uniform secondary structures, offering limited potential for the exploration of conformational space and the formation of tertiary structures. Here we report the incorporation of spiro bis-lactams to allow controlled rotation of the backbone of an iteratively synthesised foldamer. This enables precise control of foldamer shape along two orthogonal directions, likened to the aeronautical yaw and roll axes. XRD, NMR and computational data suggest that homo-oligomers adopt an extended right-handed helix with a pitch of over 30 Å, approximately that of B-DNA. Compatibility with extant foldamers to form hetero-oligomers is demonstrated, allowing greater structural complexity and function in future hybrid foldamer designs.

## Abstract (web summary)

Foldamers containing an aromatic linker can adopt a variety of backbone shapes, but exploration of conformational space has been limited to one plane. Here, spiro bis-lactams linkers in dipole-controlled foldamers enable controlled rotation of the backbone and projection of side-chains along two orthogonal directions.

## Introduction

**Fig. 1:**
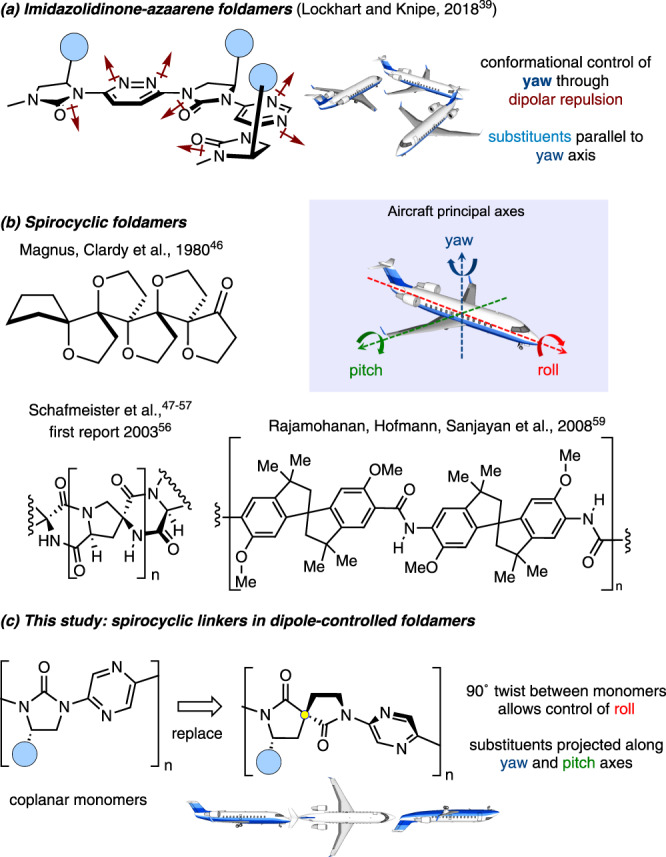
Overview of this study and its precedent. **a** Dipole-controlled foldamer allowing control of backbone shape by changing yaw angle. Localised dipoles are indicated (maroon arrows). **b** Previous foldamers incorporating spircocyles. **c** The merging of a spirocyclic monomer with the dipole-controlled foldamer concept can allow greater exploration of conformational space, and side-chain vectors along multiple axes.

Foldamers are artificial oligomers imbued with a preference to adopt well-defined conformations reminiscent of the secondary (and sometimes tertiary and quaternary) structures of biomacromolecules^1–5^. Given the vast array of functions displayed by Nature’s oligomers, there is potential for foldamers to act as a platform for molecular recognition^6–8^, catalysis^9,10^, transport^11^, and signalling^12,13^. Efficient exploration of conformational space is required to broaden the range of biomolecular structure and function that can be recapitulated. This necessitates moving beyond repetitive structures towards so-called “hetero foldamers”, where the backbone monomers within a given foldamer are dissimilar^14–16^. Backbone heterogeneity has been achieved in various ways. For example, many mixed α-/β-/γ-/δ-peptides^17–31^ and peptide-peptoid^32^ systems have been reported, allowing conformational tuning of the resulting foldamers. Sanjayan pioneered mixed aliphatic-aromatic hybrid foldamers, incorporating phenols, BINOLs and benzamides alongside aliphatic amides^33–35^. Recently, Baumann and Schmaltz inserted tricyclic and spirocyclic diproline mimetics into a collagen model peptide backbone and demonstrated that the native triple helix is retained^36,37^. We^38,39^ and the Hamilton group^40–42^ developed foldamers based on alternating azenes and cyclic ureas, leading to a predominantly planar backbone, with side-chains positioned perpendicular to that plane. Inspired by the studies of oligo-azines conducted by Lehn^43–45^, we have previously shown that the backbone of such foldamers can be contorted into a variety of shapes through judicious choice of aromatic linker (Fig. 1a)^39^. However, this general structure places limits on the exploration of conformational space that can be achieved since it is only possible to functionalise the foldamers along vectors in one plane, rather than in perpendicular directions. Since the frame of reference changes from each monomer to the next, a useful description invokes the principal axes used in aviation, where the direction of growth of the foldamer corresponds with the direction of travel of an aircraft. Thus, our previous report allows control of yaw only.

Spirocyclic linkages are rare but not unprecedented within foldamers (Fig. 1b). In 1980 Magnus, Clardy et al. reported oligospirotetrahydrofurans that possess an overall structure described by the authors as a “primary helix”^46^. Schafmeister’s “spiroligomers” comprise a repeating spiro-linked diketopiperazine-pyrrolidine motif and have been highly successful in inhibiting protein-protein interactions and as enzyme mimetics^47–57^, though the limited flexibility in these systems has led to the authors considering them a separate class from foldamers^52^. In 2008 Rajamohanan, Hofmann and Sanjayan developed spirobi(indane) oligoamide foldamers which fold in a controlled manner due to the formation of a bifurcated hydrogen bond, though the monomers were racemic so likely a mixture of stereoisomers were formed^58,59^. Parrot, Martinez et al. have reported urea-linked bis-spirolactams as PPII helix mimetics, though the structures were not oligomeric^60^.

We reasoned that introduction of a spirocyclic linker would circumvent some limitations of prior foldamers, allowing adjacent monomers to be rotated by 90° to each other along the long molecular (roll) axis (Fig. 1c). We also considered that such a system may form helices of a longer pitch than those previously reported, enabling rudimentary mimicry of larger biomacromolecules such as B-DNA.

## Results and discussion

### Synthesis of foldamers

**Fig. 2:**
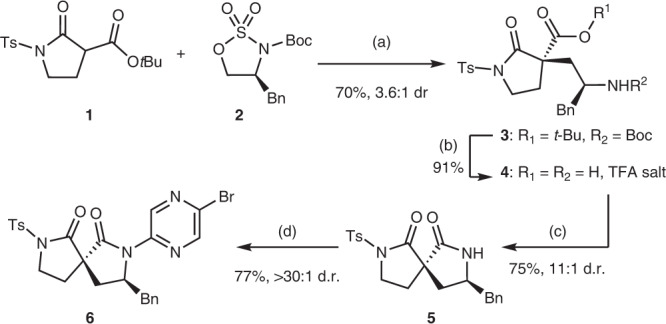
Formation of spirocycle and synthesis of iterative monomer 6. **a** **1** (1 equiv.), **2** (1.2 equiv.), Cs_2_CO_3_ (1.5 equiv.), NBu_4_Br (0.1 equiv.), PhMe:CHCl_3_ (9:1, *v*:*v*), rt, 48 h, 70% yield, 3.6:1 d.r.; **b** TFA:CH_2_Cl_2_ (1:1, *v*:*v*), rt, 24 h, 91% yield; **c** EDCI (1.1 equiv.), NMM (2.2 equiv.), CH_2_Cl_2_, rt, 24 h, 75% yield, 11:1 d.r.; **d** 2,5-dibromopyrazine (5 equiv.), Pd_2_(dba)_3_ (10 mol%), Xantphos (30 mol%), Cs_2_CO_3_ (2.5 equiv.), PhMe, 110 °C, 18 h, 77% yield, >30:1 d.r. The d.r. increases throughout the sequence due to partial separation during purification. TFA trifluoroacetic acid, EDCI 1-ethyl-3-(3-dimethylaminopropyl)carbodiimide; NMM *N-*methymorpholine.

We set about synthesising a spirocyclic bis-lactam that could act as a surrogate for the cyclic ureas used in previous studies, to determine whether this would achieve the desired control of the foldamer shape. The synthesis began with the formation of **3** by the alkylation of **1** with phenylalaninol-derived sulfamidate **2** under phase-transfer conditions according to the method of Dixon et al.^61^, which proceeded in 70% yield and 3.6:1 d.r. (Fig. 2). The absolute configuration at the new quaternary stereocentre is inferred from the single crystal structure subsequently obtained for 7. Removal of the *tert*-butyl and Boc protecting groups was achieved in 91% yield upon treatment with TFA. EDCI induced lactam formation in the resulting amino acid **4** to form monoprotected bis-lactam spirocycle **5** in 75% yield. Lastly, coupling of this spirocycle with 2,5-dibromopyrazine (present in excess to disfavour double-addition) under Buchwald–Hartwig coupling conditions generated **6**, the monomer required for iterative synthesis of the envisaged foldamer.

**Fig. 3:**
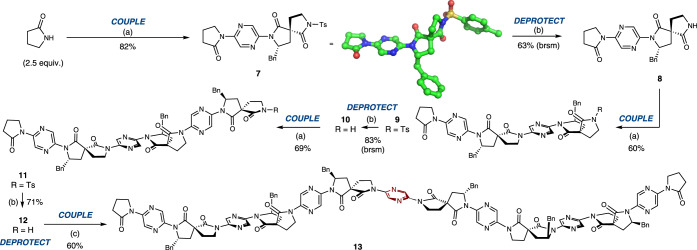
Iterative synthesis of spirocyclic foldamers. **a** **6** (1-1.2 equiv.), Pd_2_(dba)_3_ (10 mol%), Xantphos (30 mol%), Cs_2_CO_3_ (2.5 equiv.), PhMe, 80 °C; **b** TfOH (5-9 equiv.), DCE, 80 °C, 8 h; **c** 2,5-dibromopyrazine (0.5 equiv.), Pd_2_(dba)_3_ (10 mol%), Xantphos (30 mol%), Cs_2_CO_3_ (2.5 equiv.), PhMe, 80 °C, 2 h, 60% yield. Supplementary Data 2 and CCDC 2170496 contain the single crystal data for **7**. TfOH trifluoromethanesulfonic acid, DCE 1,2-dichloroethane; dba dibenzylidineacetone.

Synthesis of the foldamer commenced with 2-pyrrolidinone, which was coupled with monomer **6** under Buchwald–Hartwig conditions (Fig. 3). High temperatures and extended reaction times were deleterious to the yield in this step, leading to decomposition pathways. However, when the reaction was conducted at 80 °C for just 45 min, the coupled product **7** was obtained in 82% yield. Single crystals of **7** were obtained, and allowed unambiguous assignment of the configuration at the spirocyclic centre. Reductive methods for the removal of the *N*-tosyl protecting group (SmI_2_^62,63^; Mg/MeOH^64^; Na/naphthalene^65^, Bu_3_SnH/AIBN^66^, electrochemistry^67–69^) were low-yielding, but treatment with excess trifluoromethanesulfonic acid at 80 °C^70^ cleanly achieved the deprotection in 63% yield based on recovered starting material. In subsequent deprotections the excess of acid was increased to account for the buffering effect of the increasing number of pyrazine linkers. *N*-Deprotected monomeric foldamer **8** was coupled with monomer **6** in 60% yield to form *N*-Ts dimer **9**, which was deprotected to generate **10** in 83% yield based on recovery of starting material (brsm). Dimer **10** then underwent analogous coupling with **6** to afford trimeric foldamer **11** in 69% yield. Wishing to observe conformational behaviour in a longer oligomer, *pseudo*-hexamer **13** was rapidly constructed by the deprotection of trimer **11** in 71% yield to form **12**, followed by coupling with 0.5 equivalents of 2,5-dibromopyrazine. The *C*_*2*_-symmetrical product was obtained in 60% yield.

### Conformational analysis

**Fig. 4:**
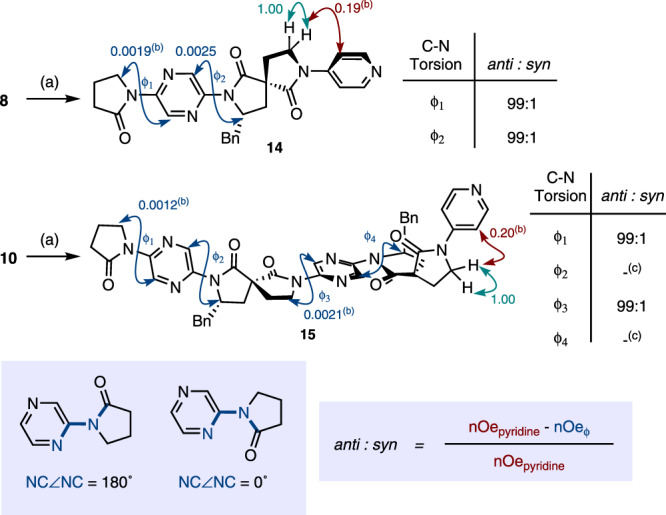
Analysis of ROESY spectral data for control compounds 14 and 15 (CDCl_3_, *t*_mix_ 0.2 s). Rotating Frame Overhauser Enhancement Spectroscopy (ROESY) cross-peak intensities are indicated, and normalised relative to the geminal enhancement (in green). *anti*: *syn* ratios about each C-N bond are approximated by the formula provided in the inset. This assumes that where cross-peaks are to a pair of diastereotopic methylene hydrogens the average of these intensities is given. **a** 4-Bromopyridine hydrochloride (1.5 equiv.), Pd_2_(dba)_3_ (10 mol%), Xantphos (30 mol%), Cs_2_CO_3_ (3.6 equiv.), PhMe, 80 °C, **14**: 4 h reaction, 75% yield, **15**: 3 h reaction, 58% yield; **b** the average value of the nOe enhancements to both diastereotopic hydrogens is given; **c** cross-peaks overlap.

With the synthesis of the foldamers thus established we sought to determine their conformational preferences. This was achieved by examination of nuclear Overhauser effect (nOe) enhancements between lactam methylene and methine and the adjacent pyrazine hydrogens (Fig. 4). For the purpose of this analysis, we assume that if a dipole-opposed *anti*-conformation were adopted (NC∠NC dihedral angle = 180°, Fig. 4 inset) such nOes would be absent. Conversely, in the dipole-aligned conformation (NC∠NC = 0°), or if the C_pyrazine_-N_lactam_ bond were freely rotating a stronger nOe would be expected. Pyridine-derived control compounds **14** and **15** were generated via Buchwald–Hartwig coupling of deprotected monomer **8** and dimer **10** with 4-bromopyridine (Fig. 4). The nOe between the pyridine *meta*-hydrogen and the adjacent methylene served as an internal control for comparison with the enhancements outlined above. The intensity of all peaks was normalised relative to a geminal methylene cross-peak since this distance is fixed across all compounds. According to this analysis, monomer **14** and dimer **15** exhibited strong preference for an *anti*-conformation about all rotatable C-N bonds, with *anti*: *syn* ratios in all cases exceeding 99:1. This approach was extended to foldamers not containing the 4-pyridyl internal control by direct integration of the nOe cross-peaks relative to the geminal reference. In all instances, weak cross-peak intensities were observed relative to the internal geminal coupling, consistent with a similar conformation to those demonstrated for **14** and **15**. The preference for an *anti*-conformation was only slightly diminished in a more polar solvent: for example, **14** gave *anti*: *syn* ratios around both ϕ_1_ and ϕ_2_ of 98:2 in *d*_*6*_-DMSO. The conformational preference was also retained at elevated temperatures (up to 348 K) in *d*_*6*_-DMSO (see Supplementary Discussion section 2.1.9).

Circular dichroism (CD) experiments were also conducted for monomer **7** and foldamers **9**, **11**, **13** and **17** in CHCl_3_ (see Supplementary Discussion section 2.2). Negative Cotton effects were observed for all compounds between ~260 and 290 nm; the fact these are observed even for **7** implies they are not indicative of secondary structure, but reflect the behaviour of individual monomers within the foldamer. However, a positive Cotton effect emerges at ~340 nm for trimer **13** and **17**, and is likely to be characteristic of the overall helical fold. Variable temperature CD experiments were consistent with NMR, showing no loss of secondary structure at elevated temperature (up to 50 °C; see Supplementary Figs. S12–S16).

**Fig. 5:**
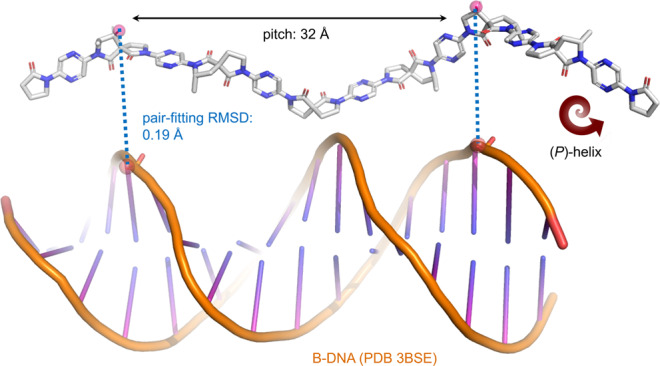
Computed lowest energy conformation of *pseudo*-hexamer 13. Side-chains are truncated to CH_3_. Semi-empirical: PM7 (MOPAC2016)^75–77^. Pair-fitting of spirocycle substituents within monomers *n* and *n* + 4 with phosphate oxygen atoms on surface of B-DNA (PDB 3BSE). For full details see Supplementary Discussion section 2.3.

The conformation of the foldamers was also investigated computationally (Fig. 5). A combined molecular mechanics/semi-empirical approach was validated by comparison of the computed structure with the single crystal data for **7** (see Supplementary Discussion section 2.3). The conformers obtained agreed with the solution phase ROESY data outlined above, with the global minimum in all cases having ϑ angles of ~0° at all C_pyrazine_-N_lactam_ linkages. These structures reveal that the molecules adopt an extended right-handed (*P*)-helical conformation, comprising a series of coplanar fragments with a 90° twist relative to their nearest neighbouring fragments, reminiscent of the herringbone foldamers reported by Huc^71^. The helix has a large overall pitch of ~32 Å and four residues per turn. *Pseudo*-hexamer **13** therefore has an overall length of ~60 Å. To our knowledge this is the largest pitch of sequence-defined helical foldamer yet reported (though such values are known in helical polymers^72–74^) and gives an overall length scale closely matching that of B-DNA (34 Å).

### Compatibility with existing foldamers

**Fig. 6:**
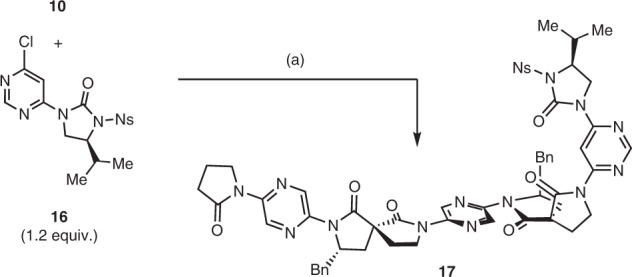
Synthesis of hybrid spirolactam-imidazolidinone 17. **a** Pd_2_(dba)_3_ (10 mol%), Xantphos (30 mol%), Cs_2_CO_3_ (2.5 equiv.), PhMe, 80 °C, 2 h, 75%. Ns 2-nitrobenzenesulfonyl.

Lastly, it was demonstrated that the spiro bis-lactam-containing foldamer is compatible with the previous imidazolidinone-containing foldamers developed within our group (Fig. 6). Coupling of dimer **10** with pyrimidine-imidazolidinone monomer **16** proceeded cleanly in 75% yield, with nOe data indicative of the expected dipole-opposed conformation in hybrid foldamer **17**, where control over both yaw (*via* the pyrimidyl-imidazolidin-2-one) and roll (*via* the spirocycle) has been achieved.

### Conclusions

Single crystal, ROESY and CD data confirm a strong and predictable conformational preference in a spirocyclic, dipole-controlled foldamer. This preference is reproduced by a semi-empirical computational approach, which predicts homo-oligomers to adopt an unusual helical conformation with a pitch of over 30 Å. Further work is required to develop functional analogues of the foldamers explored here. The simplest manner in which this may be achieved would be through incorporating functional sidechains (alcohols, amines, carboxylic acids etc.). Dixon has previously shown that spirocyclic bis-lactams bearing hydroxymethylene sidechains may be formed using the phase-transfer catalysed approach displayed in Fig. 2^61^. The strongly acidic *N*-Ts deprotection and palladium-catalysed cross-coupling conditions present a challenge for some protecting groups, so a suitable strategy must be developed. Redox triggered protecting groups may be appropriate, such as *O*-Bn groups cleaved by hydrogenolysis. This report paves the way towards functional macromolecules by allowing more complete exploration of conformational space than previously possible with related classes of foldamer, and work is ongoing to achieve control over the third principal axis—pitch. Once functional monomers and complete conformational control are achieved, these scaffolds may find applications as bespoke abiotic enzyme mimetics or as rationally designed binders of biomacromolecules.

## Methods

### General procedure for palladium-catalysed coupling of lactams with aryl halides

To a sealed tube under an inert atmosphere of argon and equipped with a magnetic stir bar was added deprotected spirocycle (1.0 equiv.), aryl halide (0.5–5.0 equiv.), freshly recrystallized Pd_2_(dba)_3_ (10 mol%), Xantphos (30 mol%) and Cs_2_CO_3_ (2.5 equiv.). Anhydrous toluene (0.1 M) was added to the flask, and the resulting suspension was then simultaneously sonicated and de-gassed by sparging with argon gas for 15–30 min. The reaction mixture was then heated at the specified temperature (80–110 °C). After complete consumption of the spirocyclic starting material by TLC analysis, the reaction was cooled to room temperature, diluted with dichloromethane (ca. 20 mL/mmol deprotected spirocycle) and filtered over Celite^®^, which was washed with ethyl acetate and the organic solvents were removed in vacuo. The crude product was purified by flash column chromatography on silica gel.

### General procedure for removal of tosyl protecting group

To a sealed tube under an inert atmosphere of argon and equipped with a magnetic stir bar was added *N*-Ts spirocycle (1.0 equiv.) and anhydrous DCE (0.04 M). The solution was then cooled to 0 °C and trifluoromethanesulfonic acid (3 equiv. + 2 equiv. per pyrazine nitrogen) added to the reaction mixture. The solution was then heated to 80 °C for 8 h, cooled to RT and quenched with a few drops of 1,2-diaminopropane, followed by addition of NaOH (1 M aq., 20 mL/mmol *N*-Ts spirocycle). The reaction mixture was transferred to a separatory funnel and extracted with CH_2_Cl_2_ (3 x ca. 20 mL/mmol *N*-Ts spirocycle). The combined organic layers were dried over MgSO_4_, filtered under gravity and concentrated *in vacuo*. The crude product was purified by flash column chromatography on silica gel.

## Supplementary information

Supplementary Information

Description of Additional Supplementary Files

Supplementary Data 1

Supplementary Data 2

## Data Availability

All data generated or analyzed during this study are included in this published article (and its supplementary information files). Supplementary Data 1 contains NMR data for all compounds; Supplementary Data 2 contains single crystal X-ray data for **7**. The X-ray crystallographic coordinates for **7** have been deposited at the Cambridge Crystallographic Data Centre (CCDC), under deposition number 2170496. These data can be obtained free of charge from The Cambridge Crystallographic Data Centre via www.ccdc.cam.ac.uk/data_request/cif. Computed lowest energy structures as shown in Fig. 3, S17–S21 are available in .mol format from 10.6084/m9.figshare.22270771. All other data are available from the corresponding author on reasonable request.
