# Supplementary material for: A spirocyclic backbone accesses new conformational space in an extended, dipole-stabilized foldamer
Source: Commun Chem. 2023 Apr 17;6:71. doi: 10.1038/s42004-023-00868-8 (PMC10110530; doi:10.1038/s42004-023-00868-8)
Supplement: Supplementary file 1 — Supplementary Information [file 42004_2023_868_MOESM1_ESM.pdf]

Supplementary Information

# A spirocyclic backbone accesses new conformational space in an extended, dipole-stabilized foldamer

William E. Roe,[a] Toyah M. C. Warnock [a] and Peter C. Knipe*[a]

[a] *School of Chemistry and Chemical Engineering, Queen's University Belfast, David Keir Building, Belfast, BT9 5AG, UK*

## Contents

1 Supplementary Methods ....3
1.1 General Experimental ....3
1.1.1 Naming and Numbering of Compounds ....3
1.1.2 Solvents and reagents ....3
1.1.3 Purification ....3
1.1.4 Spectroscopy ....3
1.1.5 Mass Spectrometry ....4
1.1.6 Crystallography ....4
1.2 Experimental procedures and characterisation data ....5
1.2.1 General procedures ....5
1.2.2 Precursors to Spirocycles ....6
1.2.3 Spirocycle synthesis ....9
1.2.4 Foldamer Characterization ....12
1.2.5 Foldamer synthesis ....15
2 Supplementary Discussion ....32
2.1 Solution-phase Conformational Analysis by NMR ....32
2.1.1 General Comments ....32
2.1.2 Quantification of *anti*:*syn* ratios ....32

2.1.3 Capped monomer 14 ....... 34
2.1.4 Ts-protected monomer 7 ....... 38
2.1.5 Deprotected monomer 8 ....... 41
2.1.6 Capped dimer 15 ....... 44
2.1.7 Ts-protected dimer 9 ....... 48
2.1.8 Deprotected dimer 10 ....... 51
2.1.9 *$d_6$*-DMSO and temperature influence on conformational control ....... 54
2.1.10 Trimer 11 ....... 65
2.1.11 Deprotected Trimer 12 ....... 69
2.1.12 Spiro-urea Hybrid Trimer 17 ....... 73
2.1.13 *pseudo*-hexamer 13 ....... 77
2.2 Circular Dichromism ....... 81
2.2.1 Homo-Spirobislactam Series at 20 °C ....... 81
2.2.2 Homo-Spirobislactam Series at 20 °C, 35 °C and 50 °C ....... 82
2.2.3 Hybrid trimer 17 ....... 86
2.3 X-ray Crystallography ....... 87
2.3.1 Single Crystal Data for 7 (CCDC 2170496) ....... 87
2.4 Computation ....... 89
2.4.1 General Method ....... 89
2.4.2 Validation ....... 89
2.4.3 Linear Conformational Searching – An Accelerated Protocol ....... 90
2.4.4 Monomer 7 ....... 91
2.4.5 Dimer 9 ....... 92
2.4.6 *pseudo*-Hexamer 13 ....... 93
3 Supplementary References ....... 94

# 1 Supplementary Methods

## 1.1 General Experimental

### 1.1.1 Naming and Numbering of Compounds

Systematic compound names are those generated by ChemBioDraw™ Ultra version 15.1.0.144 (Perkin Elmer) following IUPAC nomenclature.

### 1.1.2 Solvents and reagents

Unless stated otherwise, all other solvents and reagents used were directly obtained from commercial sources. Anhydrous and oxygen-free THF was obtained by distillation from Na/benzophenone. $Pd_2(dba)_3$ was recrystalized from Acetone/$CHCl_3$,[1] TBAB was recrystalized from toluene/n-hexane and then dried in vacuo at 60 °C,[2] $Cs_2CO_3$ was recrystalized via partial evaporation from EtOH and then dried in vacuo at 100 °C.[2]

Due to the air and moisture sensitive nature of several of the reactions, glassware was oven-dried prior to use and the reaction performed under an inert (argon) atmosphere, with solvents being dried over 3 Å molecular sieves prior to their use. Anhydrous solvents were degassed with argon for 15 minutes immediately prior to use.

### 1.1.3 Purification

Flash column chromatography was carried out using Fluorochem 60 40-63 micron silica gel. Thin-layer chromatography was carried out using Merck Kieselgel 60 F254 (230-400 mesh) fluorescent treated silica, visualized under UV light (254 nm) or by staining with aqueous potassium permanganate solution, ninhydrin or ceric ammonium molybdate solutions.

### 1.1.4 Spectroscopy

$^1H$, and $^{13}C$ NMR spectra were obtained using Bruker 600, or 400 MHz spectrometers using either $CDCl_3$ or *d*$_6$-DMSO as the solvent. To analyse and process the NMR spectra, TopSpin™ software was used, and spectra were calibrated against residual non-deuteriated solvent peaks as internal standards. The chemical shifts are reported in parts per million (ppm) and coupling constants (*J*) are reported in Hertz (Hz). $^1H$ NMR spectra are reported as follows: δ/ppm (number of protons, multiplicity, coupling constant, assignment of peak (if possible)). The multiplicities are abbreviated as follows: s = singlet, d = doublet, t = triplet, q = quartet, m = multiplet. $^{13}C$ NMR spectra are reported as follows: δ/ppm (assignment). Chemical structures are numbered arbitrarily for the purpose of assignment; this numbering scheme does not necessarily correspond with the systematic name of the compound. Two-dimensional

NMR experiments (COSY, HSQC, HMBC, NOESY, ROESY) were also recorded when necessary to help aid assignment of the proton and carbon peaks.

Infra-red (IR) spectra were recorded on an Agilent Cary 630 spectrometer equipped with an attenuated total reflectance (ATR) accessory. Samples were deposited on the ATR as a thin film or neat solid. Only selected maximum absorbances ($\nu_{max}$) of the most intense peaks are reported ($cm^{-1}$).

Optical rotations were recorded at the sodium D-line (589 nm) using a Perkin Elmer 341 polarimeter at a temperature of 20 °C and are reported in degrees using concentrations (*c*) in $g.100\ mL^{-1}$. Reported values are the average of eight readings

### 1.1.5 Mass Spectrometry

Liquid chromatography-mass spectrometry (LCMS) analyses were conducted using an instrument comprising an Agilent 1260 HPLC (equipped with Infinity II quaternary pump, vial sampler, integrated column compartment and variable wavelength detector) and MSD single quadrupole mass spectrometer. Samples were analysed using an Agilent Infinitylab poroshell 120 column (2.7 μm, 2.1 x 150 mm) under an acetonitrile/water gradient with 0.1% HCOOH additive.

High resolution mass spectra (HRMS) were recorded by Analytical Services and Environmental Projects (ASEP) at Queen's University Belfast on a Waters LCT Premier ToF mass spectrometer using the electrospray ionisation (ESI) technique.

### 1.1.6 Crystallography

Low temperature[3] single crystal Xray diffraction studies were carried out using $CuK_{\alpha}$ radiation on an Agilent Supernova diffractometer equipped with an area detector and graphite monochromator. Raw frame data were reduced using CrysAlisPRO[4] solved using SHELXT.[5] Full-matrix least-squares refinement of the structures were carried out using CRYSTALS.[6, 7] Full refinement details are given in the supplementary material (CIF). CCDC 2170496 contains the supplementary crystallographic data for this paper. These data are provided free of charge by The Cambridge Crystallographic Data Centre and copies can be obtained free of charge *via* www.ccdc.cam.ac.uk/data_request/cif.

# 1.2 Experimental procedures and characterisation data

## 1.2.1 General procedures

### General Procedure A: Palladium-Catalysed Coupling of Deprotected spirocycles with Aryl Halides

This reaction was carried out by analogy to a literature procedure.[8] To a sealed-tube under an inert atmosphere of argon and equipped with a magnetic stir bar, was added deprotected spirocycle (1.0 eq.), aryl halide (0.5-5.0 eq.), freshly recrystallized $Pd_2(dba)_3$ (10 mol%) Xantphos (30 mol%) and $Cs_2CO_3$ (2.5 eq.). Anhydrous toluene (ca. 0.1 M) was added to the flask, and the resulting suspension was then simultaneously sonicated and de-gassed by sparging with argon gas for 15-30 minutes. Following this the reaction mixture was then heated at the specified temperature until all of the spirocyclic starting material was consumed. After complete consumption of the spirocyclic starting material by TLC analysis, the reaction was cooled to room temperature, diluted with dichloromethane (ca. 20 mL/mmol deprotected urea) and filtered over Celite®, which was also then washed with EtOAc. The crude product was then purified by flash column chromatography.

### General Procedure B: Tosyl Deprotection

This reaction was carried out by analogy to a literature procedure.[9] To a sealed-tube under an inert atmosphere of argon and equipped with a magnetic stir bar, was added *N*-Ts spirocycle (1.0 eq.) and dry DCE (0.04 M), then solution was then cooled down to 0 °C and triflic acid (3eq. + 2eq. per pyrazine nitrogen) added to the reaction mixture. After which, the solution was then heated to 80 °C for 8 h. Following this the solution was then cooled down to rt and then quenched with a few drops of 1,2-Diaminopropane, followed by addition of 1.0 M $NaOH_{(aq)}$ (ca. 20 mL/mmol *N*-Ts spirocycle). The reaction mixture was then transferred to a separatory funnel and then extracted with DCM three times (ca. 20 mL/mmol *N*-Ts spirocycle). The combined organic layers were then dried over $MgSO_4$, filtered under gravity and then concentrated in vacuo. The crude product was then purified by flash column chromatography.

### 1.2.2 Precursors to Spirocycles

1-tosylpyrrolidin-2-one (**S1**)

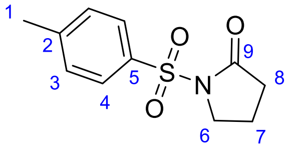


To a cooled solution (-78 °C) of 2-pyrrolidinone (1.0 eq.) in anhydrous THF (0.8 M) under argon was added *n*-BuLi (1.6 M in hexanes, 1.05 eq.) dropwise, with the reaction mixture then being left to stir at -78 °C for 1.5 h. Following this a solution of TsCl (1.05 eq.) in anhydrous THF (2.7 M) was added dropwise over a period of 20 minutes. The reaction was then left to stir at -78 °C for 20 minutes and then warmed up to room temperature and allowed to react for a further hour. After stirring for 1 h, the reaction was then quenched by slowly adding saturated $NH_4Cl_{(aq)}$. The solution was then transfer to a separatory funnel, and the aqueous layer extracted with EtOAc three times. The combined organic layers were then washed with brine, dried with $MgSO_4$, filtrated under gravity, and then concentrated in vacuo to yield an off-white solid. The product was then purified by trituration from diethyl ether, to yield a white solid, 2.0 g SM (4.48 g, 80%), 10.0 g SM (19.4 g, 69%).

**$\delta_H$** (400 MHz, $CDCl_3$): 7.93 (2H, d, *J* 8.4 Hz, **H4**), 7.34 (2H, d, *J* 8.3 Hz, **H3**), 3.90 (2H, t, *J* 7.1 Hz, **H6**), 2.45-2.40 (5H, m, **H1** and **H8**), 2.07 (2H, quin, *J* 7.5 Hz, **H7**); **$\delta_C$** (101 MHz, $CDCl_3$): 174.4 (**C9**), 145.3 (**C2/5**), 135.3 (**C2/5**), 129.8 (**C3**), 128.2 (**C4**), 47.4 (**C6**), 32.3 (**C8**), 21.8 (**C1**), 18.3 (**H7**); **HRMS** (ESI+): found 240.0694; $C_{11}H_{13}NO_3SH$, $[M+H]^+$ requires 240.0689; ***$\nu_{max}$*** **(neat)**: 3101.1, 2996.8, 2914.8, 2079.9, 1923.3, 1725.8, 1595.3, 1353.0, 1155.5, 1114.5, 1088.4; Data are consistent with Blanc *et al.*[10]

(±)-*tert*-Butyl 2-oxo-1-tosylpyrrolidine-3-carboxylate (**1**)

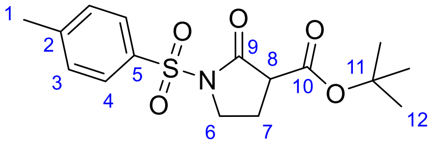


To a cooled solution (-78 °C) of 1-tosylpyrrolidin-2-one/**S1** (1.0 eq.) in anhydrous THF (0.25 M) under argon, was added NaHMDS (2.0 M in THF, 2 eq.) dropwise, followed by the dropwise addition of $(Boc)_2O$ (1 eq.) in ahydrous THF (2 M). The reaction mixture was then left to stir at -78 °C until all of the starting material had been consumed via TLC. Following the completion of the reaction it was then quenched with $NH_4Cl_{(aq)}$ and the reaction mixture warmed to rt. The reaction mixture was then transferred to a separatory funnel, and EtOAc added, and the layers

then separated. The aqueous layer was then extracted three times with EtOAc. The combined organic layers were then washed with brine, dried over $MgSO_4$, filtered under gravity and then concentrated in vacuo to yield a yellow solid. The crude product was purified by flash column chromatography using 20% → 30% EtOAc:Petroleum ether as the eluent, to yield the product as an flaky white solid: 1.5 g SM (1.50 g, 77% (BRSM)), 10.0 g SM (10.4 g, 82% (BRSM))

**$\delta_H$** (400 MHz, $CDCl_3$): 7.90 (2H, d, *J* 8.3 Hz, **H4**), 7.32 (2H, d, *J* 8.1 Hz, **H3**), 3.99-3.91 (1H, ddd, *J* 9.5, 8.2, 6.1 Hz, **H6'**), 3.88-3.80 (1H, ddd, *J* 9.7, 7.9, 5.9 Hz, **H6**), 3.30 (1H, ddd, *J* 9.7, 8.0, 5.8 Hz, **H8**), 2.42 (3H, s, **H1**), 2.40-2.20 (2H, m, **H7**), 1.34 (9H, s, **H12**); **$\delta_C$** (101 MHz, $CDCl_3$): 168.7 (**C9**), 167.0 (**C10**), 145.4 (**C2/5**), 134.9 (**C2/5**), 129.8 (**C3**), 128.3 (**C4**), 82.8 (**C11**), 50.7 (**C8**), 45.9 (**C6**), 27.9 (**C12**), 22.5 (**C7**), 21.7 (**C1**); **HRMS** (ESI+): found 378.0772; $C_{16}H_{21}NO_5SK$, $[M+K]^+$ requires 378.0772; $\nu_{max}$ (**neat**): 2978.1, 2933.4, 2363.1, 1744.4, 1595.3, 1353.0,1230.0, 1140.6, 1114.5; Data are consistent with Dixon *et al.*[11]

*tert*-Butyl (*S*)-(1-hydroxy-3-phenylpropan-2-yl)carbamate (**S2**)

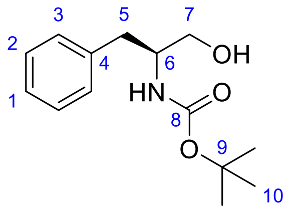


To cooled solution (0 °C) of L-phenylalaninol (1.0 eq.) in DCM (0.75 M) was added $NEt_3$ (1.05 eq.) dropwise, followed by $Boc_2O$ (1.05 eq.). The mixture was warmed to rt and then left to stir until all of the starting material had been consumed via TLC. Once all of the starting material had been consumed the reaction mixture was then diluted with DCM and brine and then transferred to a separatory funnel. The layers were then separated, and the aqueous layer washed with three times with DCM. The organic layers were then combined, dried over $MgSO_4$, and concentrated in vacuo to yield an off-white solid, no further purification was required, 1.0 g SM (1.60 g, 95%), 5.0 g SM (7.99 g, 96%).

**$\delta_H$** (400 MHz, $CDCl_3$): 7.33-7.27 (2H, m, ***Ar***), 7.25-7.18 (3H, m, ***Ar***), 4.84 (1H, s, **NH**), 3.87 (1H, bs, **H6**), 3.68-3.61 (1H, m, **H7'**), 3.58-3.50 (1H, m, **H7**), 2.83 (2H, d, *J* 7.1 Hz, **H5**), 2.69 (1H, s, **OH**), 1.41 (1H, s, **H11**); **$\delta_C$** (101 MHz, $CDCl_3$): 156.3 (**C8**), 138.0 (**C4**), 129.4 (***Ar***), 128.6 (***Ar***), 126.6 (***Ar***), 79.8 (**C9**), 64.3 (**C7**), 53.9 (**C6**), 37.6 (**C5**), 28.5 (**C10**); **HRMS** (ESI+): found 290.1147; $C_{14}H_{21}NO_3K$, $[M+K]^+$ requires 290.1153; $\nu_{max}$ (**neat**): 3354.6, 3026.6, 2981.9, 2873.8, 2102.2, 1684.8, 1528.2, 1442.5, 1315.8, 1267.3, 1166.7, 1006.4; Data are consistent with Borggraeve *et al.*[12]

*tert*-Butyl (*S*)-4-benzyl-1,2,3-oxathiazolidine-3-carboxylate 2,2-dioxide (**2**)

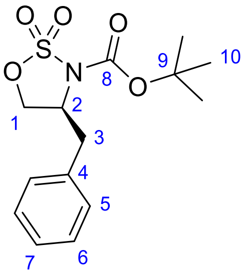


To a cooled solution (-20 °C) of *N*-Boc *L*-phenylalaninol/**S2** (1.0 eq.) in MeCN (2 M) under argon was added a solution of $SOCl_2$ (1.25 eq.) in MeCN (0.25 M) dropwise. After stirring for 10 min, pyridine (4.0 eq.) was then added dropwise and the mixture left to stir for 1 h, after which it was then warmed to 0 °C and left for a further hour. After 1 hour, $H_2O$ and EtOAc were added to the reaction mixture, which was then transferred to a separatory funnel. The layers were separated and the organic layer washed with 1M HCl. Next, the combined aqueous layers were extracted with EtOAc three times, and the combined organic layers was then washed successively with sat aq. $NaHCO_3$ and then brine. Afterwards, the combined organic layers were then dried over $MgSO_4$, filtered under gravity and then concentrated in vacuo, to yield the crude intermediate as an orange oil. Following this, the crude intermediate was dissolved in MeCN (0.25 M) and cooled to 0 °C. Afterwards, $RuCl_3.H_2O$ (10 wt%), $NaIO_4$ (1.5 eq.) and water (0.25 M) were added successively, and the reaction mixture stirred vigorously for 2 h at 0°C. Following this the reaction mixture was then diluted with $H_2O$ and $Et_2O$, which was then transfer to a separatory funnel. The layers were then separated and the aqueous layer washed with three times with $Et_2O$. The combined organic layer was then washed successively with $NaHCO_{3(aq)}$ and then brine, after which the combined aqueous layers were extracted with $Et_2O$ three times. The organic layers were then combined, dried over $MgSO_4$, filtered under gravity, and concentrated to yield an off-white solid. The crude was then purified via trituration from diethyl ether, to yield a flaky white solid: 2.6 g SM (1.83 g, 57%), 7.65 g SM (6.58 g, 69%).

**$\delta_H$** (400 MHz, $CDCl_3$): 7.38-7.20 (5H, m, ***Ar***), 4.49-4.40 (2H, m, **H3'+H2**), 4.34-4.30 (1H, m, **H3**), 3.36 (1H, dd, *J* 13.5, 4.2 Hz, **H1'**), 2.92 (1H, dd, *J* 13.5, 10.2 Hz, **H1**), 1.56 (9H, s, **H9**); **$\delta_C$** (101 MHz, $CDCl_3$): 148.6 (**C8**), 135.3 (**C4**), 129.6 (***Ar***), 129.2 (***Ar***), 127.6 (***Ar***), 85.7 (**C9**), 68.9 (**C3**), 58.7 (**C2**), 38.0 (**C1**), 28.1 (**C10**); **$[\alpha]_D^{20}$ -28.8** (*c* = 1.02, $CHCl_3$); **HRMS** (ESI+): found 314.1062; $C_{14}H_{19}NO_5SH$, $[M+H]^+$ requires 314.1057; ***$\nu_{max}$*** (**neat**): 2981.9, 1714.6, 1367.9, 1319.5, 1185.3, 1148.0; Data are consistent with Dixon *et al.*[11]

### 1.2.3 Spirocycle synthesis

*tert*-Butyl-3-((*S*)-2-((*tert*-butoxycarbonyl)amino)-3-phenylpropyl)-2-oxo-1-tosylpyrrolidine-3-carboxylate (**3**)

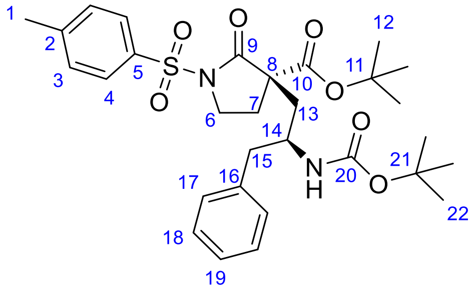


To a round-bottomed flask under an inert atmosphere of argon was added lactam **1** (1 eq.), sulfamidate **2** (1.2 eq.), recrystallised $Bu_4NBr$ (0.1 eq.), dry toluene and dry $CHCl_3$ (9:1, 0.1 M) which was then left to stir until all of the SM had dissolved. Following this, recrystallised $Cs_2CO_3$ (1.5 eq.) was added to the reaction mixture, and the reaction stirred until TLC showed complete consumption of SM (24-48 h). Once the reaction had completed, the reaction mixture was diluted with 1M HCl and $H_2O$ (10 mol per mmol of SM), followed by the addition of DCM. The solution was then transferred to a separatory funnel, and the layers separated. The aqueous layer then washed with DCM (3 x 10 mL per mmol of SM). The combined organic layers were dried over $MgSO_4$, filtered under gravity and then concentrated in vacuo to yield a yellow-orange residue. The crude product was purified by flash column chromatography using 30% $Et_2O$ in petroleum ether as the eluent, to yield the product as a sticky white solid: 803 mg SM (952 mg, 70%, 2.7:1 dr), 2.5 g (2.93 g, 70%, 3.6:1 dr).

**Major diastereomer: $\delta_H$** (400 MHz, $CDCl_3$): 7.92 (2H, d, *J* 8.3 Hz, **H4**), 7.31 (2H, d, *J* 8.3 Hz, **H3**), 7.29-7.10 (5H, m, **H17-19**), 4.28 (1H, d, *J* 10.5 Hz, **NH**), 4.06-3.96 (1H, m, **H14**), 3.92 (1H, t, *J* 9.2 Hz, **H6'**), 3.67 (1H, q, *J* 9.0 Hz, **H6**), 2.78-2.68 (2H, m, **H15**), 2.40 (3H, s, **H1**), 2.38-2.29 (1H, m, **H7'**), 2.20 (1H, dd, *J* 12.9, 7.3Hz, **H7**), 2.13 (1H, dd, *J* 15.0, 3.7 Hz, **H13'**), 2.00 (1H, dd, *J* 14.7, 11.8 Hz, **H13**), 1.39 (9H, s, **H12**), 1.08 (9H, s, **H22**); **Minor diastereomer: $\delta_H$** (400 MHz, $CDCl_3$): 7.87 (2H, d, *J* 8.5 Hz, **H4**), 7.31 (2H, m, **H3**), 7.29-7.10 (5H, m, **H17-19**), 4.42 (1H, d, *J* 10.0 Hz, **NH**), 3.88-3.85 (1H, m, **H6'**), 3.82-3.74 (2H, m, **H6+H14**), 2.80 (1H, dd, *J* 13.3, 5.5 Hz, **H15'**), 2.63 (1H, dd, *J* 13.3, 7.8 Hz, **H15**), 2.51-2.47 (1H, m, **H7**), 2.40 (3H, s, **H1**), 1.87 (1H, td, *J* 13.1, 9.3 Hz, **H7'**), 1.34 (9H, s, **H12**), 1.18 (9H, s, **H22**);* **Major $\delta_C$** (101 MHz, $CDCl_3$): 172.3 (**C9**), 168.7 (**C10**), 155.3 (**C20**), 145.2 (**C2/5**), 137.6 (**C16**),

* Due to the proximity of the minor diastereomer peaks to the major diastereomer the integration of these peaks is larger than the expected value. Additionally, **H3 & H13** minor diastereomer peaks cannot be observed due to being obscured by the major diastereomer.

135.1 (**C2/5**), 129.7 (**C3**), 129.6 (***Ar***), 128.5 (***Ar***), 128.4 (**C4**), 126.6 (***Ar***), 82.8 (**C21**), 79.8 (**C11**), 56.7 (**C8**), 48.6 (**C14**), 45.2 (**C6**), 43.1 (**C15**), 36.4 (**C13**), 28.3 (**C12**), 27.5 (**C22**), 25.7 (**C7**), 21.7 (**C1**); **[α]$_D$$^{20}$** -78.8 (*c* = 0.92, $CHCl_3$); **HRMS** (ESI+): found 573.2624; $C_{30}H_{40}N_2O_7SH$, $[M+H]^+$ requires 573.2629; $\nu_{max}$ (**neat**): 3432.9, 3391.9, 2970.7, 2914.8, 2083.6, 1710.8, 1595.3, 1498.4, 1356.8, 1237.5, 1155.5, 1110.7; Data are consistent with Dixon *et al.*[11]

(2*S*)-1-(3-carboxy-2-oxo-1-tosylpyrrolidin-3-yl)-3-phenylpropan-2-aminium (**4**)

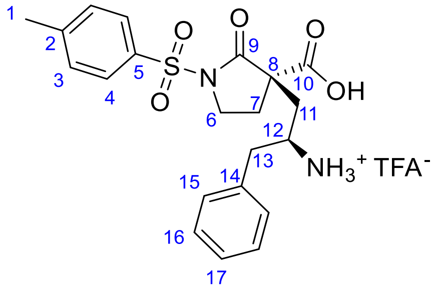


To a cooled (0°C) solution of *tert*-butyl ester **3** in dry DCM under argon was added TFA [1:1 (DCM:TFA), 0.1 M]. The reaction mixture was then warmed to rt then left to stir for 24 h. Once the reaction had completed the solvent was evaporated off under a stream of compressed air the concentrated reaction mixture was precipitated with dry diethyl ether to yield the product as a flaky white solid: 1.79 g SM (1.45 g, 90%), 2.8 g SM (2.29 g, 91%).

**Major $\delta_H$:** (400 MHz, methanol-d4): 7.90 (2H, d, *J* 8.4 Hz, **H4**), 7.40-7.32 (4H, m, **H3**+***Ar***), 7.31-7.29 (1H, m, ***Ar***), 7.19-7.15 (2H, m, ***Ar***), 3.99-3.89 (2H, m, **H6**), 3.75-3.67 (1H, m, **H12**), 2.72 (1H, dd, *J* 14.3, 9.0 Hz, **H13'**), 2.60 (1H, dd, *J* 14.3, 6.0 Hz, **H13**), 2.58-2.50 (1H, m, **H7'**), 2.35 (3H, s, **H1**), 2.13 (1H, dd, *J* 15.5, 6.1 Hz, **H11'**), 2.07-1.98 (2H, m, **H7+11**); **Minor $\delta_H$** (400 MHz, methanol-d4): 7.86 (2H, d, *J* 8.5 Hz **H4**), 7.40-7.29 (4H, m, **H3+*Ar***), 7.23-7.20 (3H, m, ***Ar***) 3.82-3.78 (2H, m, **H6**), 3.59-3.51 (1H, m, **H12**), 2.94 (1H, dd, *J* 13.8, 6.8 Hz, **H13'**), 2.79 (1H, dd, *J* 13.8, 8.2 Hz, **H13**), 2.43 (3H, s, **H1**), 2.40-2.38 (1H, m, **H7'**), 2.07-1.98 (1H, m, **H11'**), 1.91 (1H, dd, *J* 15.5, 3.1 Hz, **H11**), 1.83 (1H, td, *J* 13.3, 9.0 Hz, **H7**);[*] **Major $\delta_C$** (101 MHz, methanol-d4): 173.5 (**C10**), 173.0 (**C9**), 147.3 (**C2/5**), 136.7 (**C14**), 135.6 (**C2/5**), 130.9 (**C3**), 130.3 (***Ar***), 130.1 (***Ar***), 129.3 (**C4**), 128.7 (***Ar***), 56.8 (**C8**), 51.0 (**C12**), 46.2 (**C6**), 41.3 (**C13**), 37.3 (**C11**), 31.1 (**C7**), 21.6 (**C1**); **[α]$_D$$^{20}$** -21.3 (*c* = 1.05, MeOH); **HRMS** (ESI+): found 417.1484; $C_{21}H_{25}N_2O_5S$, $[M]^+$ requires 417.1479; $\nu_{max}$ **(neat)**: 3034.1, 2918.5, 2091.0, 1722.0, 1622.4, 1595.3, 1356.8, 1166.7, 1133.1.

---

[*] Due to the proximity of the minor diastereomer peaks to the major diastereomer, a few of the minor diastereomer peaks (**H3**+***Ar*** **& H11**) cannot be observed due to being obscured by the major diastereomer. Therefore, approximations of the position have been given as a result of cross-peaks visible in the COSY.

(8*S*)-8-Benzyl-2-tosyl-2,7-diazaspiro[4.4]nonane-1,6-dione (**5**)

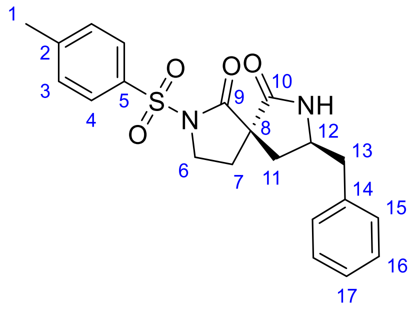

To a cooled (0 °C) solution of **4** in DCM (0.025 M) under argon was added *N*-methylmorpholine (2.2 eq.), followed by EDCI (1.1 eq.) after all the SM had dissolved. The reaction mixture was then warmed to rt and then left to stir until TLC indicated all of the SM had been consumed. Once the reaction had completed 1M HCl (20 mL per mmol of SM) and $H_2O$ (20 mL per mmol of SM) were added to the reaction mixture and the solution transferred to a separatory funnel. The layers were separated, and the aqueous layer then washed with DCM (3 x 20 ml per mmol of SM). The organic layers were then combined, dried over $MgSO_4$, filtered under gravity and then concentrated in vacuo to yield a faint orange-white solid. The crude product was purified by flash column chromatography using 0.5% MeOH:DCM as the eluent, to yield the product **5** as a white foam solid: 351 mg SM (194 mg, 71%, 11.8:1 dr), 1.4 g SM (780 mg, 73%, 11.7:1 dr), 2.2 g SM (1.30 g, 76%, 11.2:1 dr).

**Major $\delta_H$** (600 MHz, $CDCl_3$): 7.93 (2H, d, *J* 8.3 Hz, **H4**), 7.33 (2H, d, *J* 8.3 Hz, **H3**), 7.30-7.27 (2H, m, **H16**), 7.25-7.21 (1H, m **H17**), 7.14 (2H, d, *J* 7.7 Hz, **H15**), 5.59 (1H, s, **NH**), 4.06 (1H, td, *J* 9.4, 7.3 Hz, **H6'**), 3.99 (1H, td, *J* 9.0, 2.0 Hz, **H6**), 3.79-3.76 (1H, m, **H12**), 2.91-2.81 (2H, m, **H13**), 2.46 (1H, ddd, *J* 12.9, 7.0, 1.9 Hz, **H7'**), 2.44-2.38 (1H, m, **H11'**) 2.42 (3H, s, **H1**), 2.04 (2H, m, **H7 + H11**); **Minor $\delta_H$** (600 MHz, $CDCl_3$): 7.85 (2H, d, *J* 8.3 Hz, **H3/4**), 7.35-7.20 (5H, m, **H3/4 + *Ar***), 7.10 (2H, d, *J* 7.6 Hz, ***Ar***), 5.78 (1H, s, **NH**), 3.95-3.86 (1H, m, **H6'**), 2.66 (1H, dd, *J* 13.0, 6.9 Hz, **H7'**), 2.59 (1H, dd, *J* 13.6, 8.4 Hz, **H13'**), 2.42 (3H, s, **H1**), 1.94 (1H, dt, *J* 13.0, 8.8 Hz, **H11'**), 1.68 (1H, dd, *J* 13.4, 7.4 Hz, **H7**);* **Major $\delta_C$** (151 MHz, $CDCl_3$): 173.9 (**C10**), 172.3 (**C9**), 145.4 (**C2/5**), 137.4 (**C14**), 134.9 (**C2/5**), 129.8 (**C3**), 129.1 (**C15**), 129.0 (**C16**), 128.3 (**C4**), 127.1 (**C17**), 54.8 (**C8**), 53.4 (**C12**), 45.3 (**C6**), 43.2 (**C13**), 36.3 (**C11**), 30.5 (**C7**), 21.9 (**C1**); **$[\alpha]_D^{20}$** 56.5 (*c* = 1.05, $CHCl_3$); **HRMS** (ESI+): found 399.1377; $C_{21}H_{22}N_2O_4SH$, $[M+H]^+$ requires 399.1373; **$\nu_{max}$ (neat)**: 3347.1, 3220.4, 3060.1, 3026.6, 2918.5, 2117.1, 1733.2, 1692.2, 1595.3, 1449.9, 1353.0, 1226.3, 1166.7, 1088.4.

* **H6**, **H11**, **H12**, and **H13** and are missing due to being obscured by the corresponding peak of the major diastereomer

### 1.2.4 Foldamer Characterization

Due to the similarity/repeating nature of the foldamers backbone it was vital to use a range of two-dimensional NMR experiments (COSY, HSQC, HMBC, NOESY, ROESY) to allow for each bislactam and pyrazine unit to be distinguished from one another and enable full assignment of the proton and carbon peaks for all foldamer species. Detailed below are representative assignment examples for both the protected (**9**) and deprotect (**10**) dimers explaining how each bislactam and pyrazine unit was assigned based on the following interactions. This assignment methodology was also applied to all other foldamer compositions when determining peak assignment via analogues 2D NMR interactions.

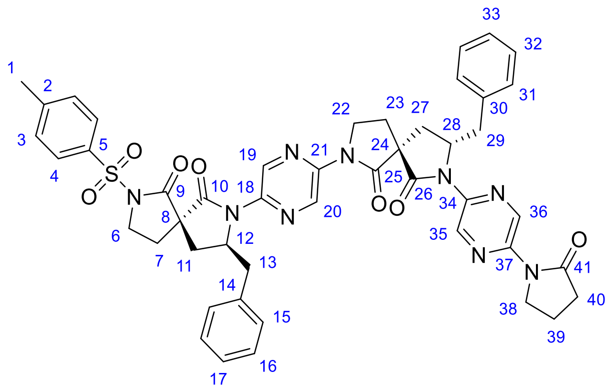

**Figure S1.** Structure and atom numbering for **dimer 9**

**Protected dimer 9:** First starting with the 2-pyrrolidinone capped unit, nOe interactions between **H38** and **H35/36** help differentiate the **1st pyrazine ring**, whilst the nOe interaction between **H28** and **H35 & H36** help determine the **1st spiro-bislactam unit**. **(Figure S2)** From **H28**, **H27** could then be determined via COSY interaction, with the connected bislactam ring (**H22/23**) being determined via a mutual HMBC interaction of spirocentre **C24**. **H22** could then be distinguished from **H23** via the presence of a nOe interaction between **H22** & **H19/20. (Figure S2)**

To confirm/establish the **2nd spiro-bislactam** (tosyl-protected), nOe interactions between **H4** and **H6** can be observed, allowing **H6** and **H7** to be assigned and differentiated from the similar environments of **H22** & **H23**. Again, mutual HMBC interaction of spirocentre **C8**, helped determine **H11 & H12;** which due to their different number of protons can be easily assigned. Corrected assignment of the **2nd spiro-bislactam** can also then be validated against **H12**'s assignment nOe interaction of **H12** & **H19/20**.

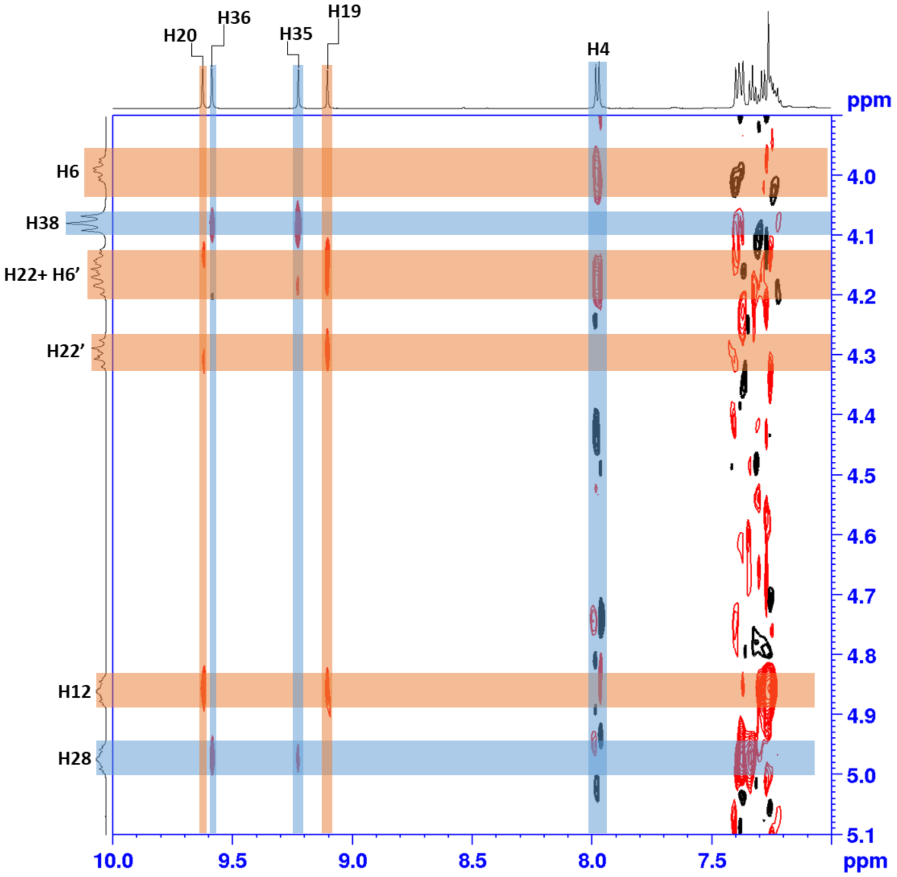


**Figure S2.** Key nOe interactions used in the peak assignment for Ts-protected **dimer 9**

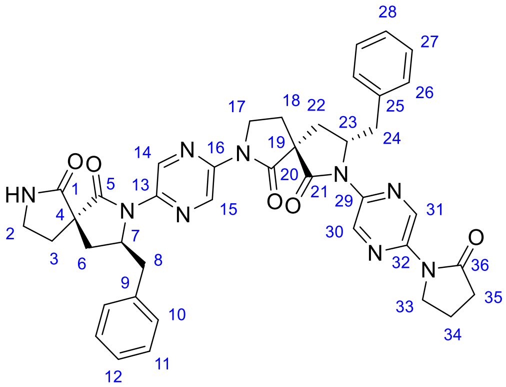

**Figure S3.** Structure and atom numbering for deprotected **dimer 10**

**Deprotected dimer 10:** Assignment of the deprotected dimer peaks follows a similar methodology to the Ts-protected species, with the 2-pyrrolidione unit being used to determine the relative pyrazine and spiro-bislactam (see description above and **Section 3.8**).

However, to establish which peaks belong to the deprotected bislactam ring a HMBC interaction between **NH** and **H2, H3, C4** can be observed, allowing for these spiro-bislactam unit to be distinguished from similar chemical environments of **H17** & **H18**. (**Figure S4**) Assignment of **H2** & **H3** can then be made via comparison to the monomers **7** & **8** or protect

dimer **9**, where the $CH_2$ protons adjacent to the amide's nitrogen are shifted more downfield relative to $CH_2$-spirocentre. From here the mutual HMBC interaction of spirocentre **C4**, helps determine **H6 & H7**, which due to their different number of protons can be easily assigned, in addition to the nOe interaction between **H7** & **H14/15**. (**See Section 3.8**)

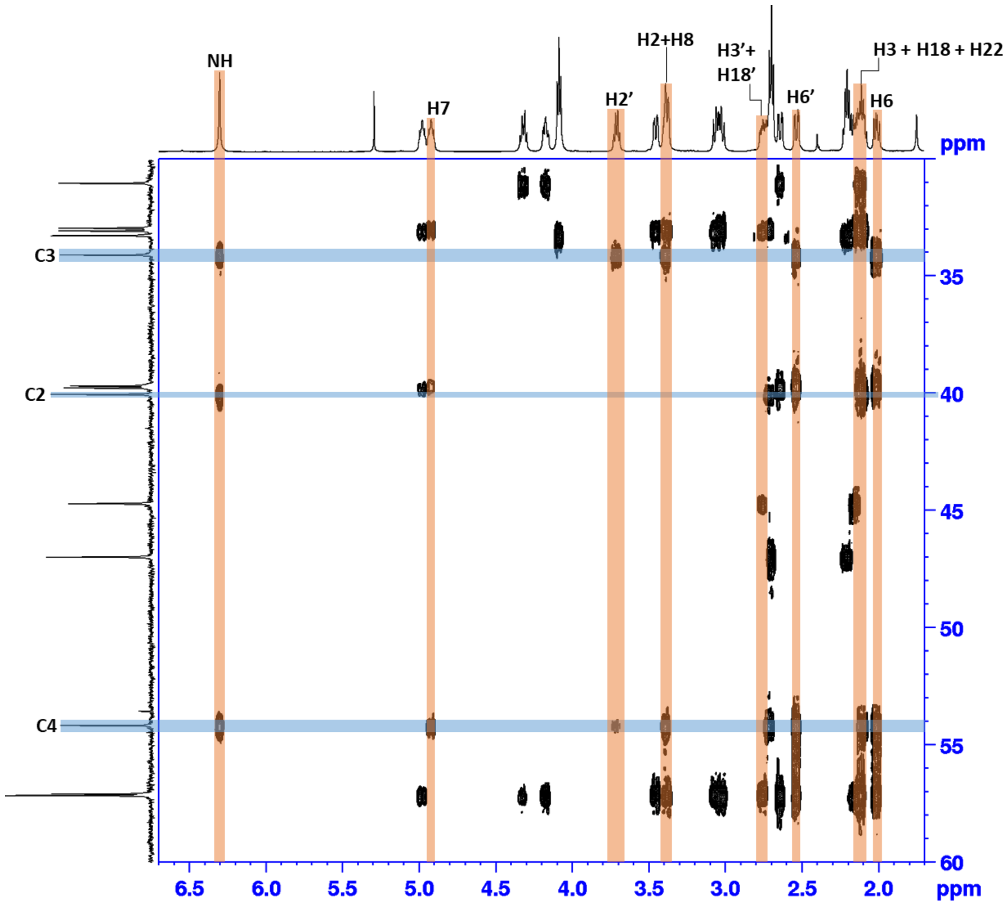


**Figure S4.** Key HMBC cross-peaks used in the assignment of **H2/H3** vs **H17/H18** for de-protected **dimer 10**

### 1.2.5 Foldamer synthesis

#### *1.2.5.1 Monomers*

(3*S*,5*R*)-3-Benzyl-2-(5-bromopyrazin-2-yl)-7-tosyl-2,7-diazaspiro[4.4]nonane-1,6-dione (**6**)

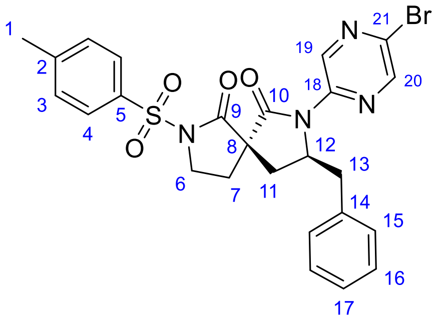


Prepared according to **General Procedure A** using **5** (1.0 g, 2.51 mmol, 1 eq.), 2,5-dibromopyrazine (2.98 g, 12.5 mmol, 5.0 eq.), $Pd_2(dba)_3$, (230 mg, 0.25 mmol, 10 mol%), Xantphos (436 mg, 0.75 mmol, 30 mol%), $Cs_2CO_3$ (2.04 g, 6.26 mmol, 2.5 eq.), and toluene (25mL). Reaction temperature = 110 °C and reaction time = 18 h. Purification by flash column chromatography (silica gel, 1% acetone:toluene) afforded the product **6** as a yellow foam solid, (1.07 g, 77%, dr >30:1).

**$\delta_H$** (400 MHz, $CDCl_3$): 9.22 (1H, d, *J* 1.4 Hz, **H19**), 8.49 (1H, d, *J* 1.5 Hz, **H20**), 7.97 (2H, d, *J* 8.4 Hz, **H4**), 7.39 (2H, d, *J* 8.1 Hz, **H3**), 7.33-7.27 (2H, m, ***Ar***), 7.26-7.23 (3H, m, ***Ar***), 4.83-4.75 (1H, m, **H12**), 4.18 (1H, m, *J* 8.6 Hz, **H6'**), 4.01 (1H, td, *J* 8.9, 3.0 Hz, **H6**), 3.31 (1H, dd, *J* 12.8, 3.6 Hz, **H13'**), 2.90 (1H, dd, *J* 12.8, 11.0 Hz, **H13**), 2.58 (1H, ddd, *J* 13.1, 7.3, 3.0 Hz, **H7'**), 2.48 (1H, s, **H1**), 2.44 (1H, dd, *J* 14.0, 2.7 Hz, **H11'**), 2.04 (1H, dt, *J* 13.1, 8.6 Hz, **H7**), 1.95 (1H, dd, *J* 14.0, 9.0 Hz, **H11**); **$\delta_C$** (101 MHz, $CDCl_3$): 171.9 (**C10**), 171.7 (**C9**), 146.3 (**C18/21**), 145.7 (**C2/5**), 144.6 (**C20**), 138.8 (**C19**), 137.4 (**C14**), 134.7 (**C2/5**+**C18/21**)*, 129.9 (**C3**), 129.7 (***Ar***), 128.8 (***Ar***), 128.3 (**C4**), 126.9 (***Ar***), 57.0 (**C12**), 56.3 (**C8**), 45.4 (**C6**), 39.3 (**C13**), 31.9 (**C7**), 31.1 (**C11**), 21.9 (**C1**); **$[\alpha]_D^{20}$** +16.5 (*c* = 1.05, $CHCl_3$); **HRMS** (ESI+): found 555.0702; $C_{25}H_{23}BrN_4O_4SH$, $[M+H]^+$ requires 555.0696; **$\nu_{max}$ (neat)**: 3026.6, 2922.2, 2119.0, 1731.3, 1697.8, 1595.3, 1444.3, 1336.3, 1228.2, 1164.8, 1086.5.

---

* HMBC cross-peaks indicate the presence of two co-incident $^{13}C$ resonances.

(3*S*,5*R*)-3-Benzyl-2-(5-(2-oxopyrrolidin-1-yl)pyrazin-2-yl)-7-tosyl-2,7-diazaspiro[4.4]nonane-1,6-dione (**7**)

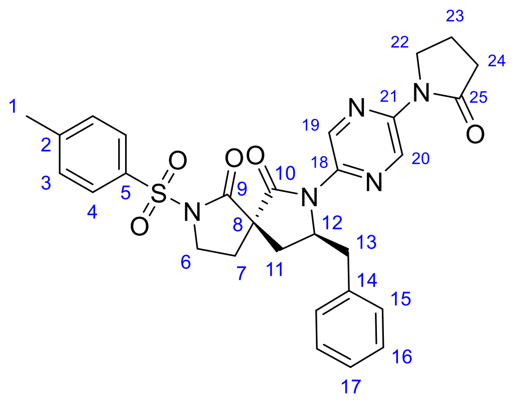


To a sealed-tube under an inert atmosphere of argon and equipped with a magnetic stir bar, was added **6** (450 mg, 0.81 mmol, 1.0 eq.), 2-pyrrolidinone (0.15 mL, 2.02 mmol, 2.5 eq.), $Pd_2(dba)_3$ (74 mg, 0.081 mmol, 10 mol%), and Xantphos (141 mg, 0.24 mmol, 30 mol%), $Cs_2CO_3$ (660 mg, 2.03 mmol, 2.5 eq.) and toluene (8 mL) was added to the flask, and the resulting suspension was then simultaneously sonicated and de-gassed by sparging with argon gas for 15-30 min. Following this the reaction mixture was then heated to 80 °C under an argon atmosphere and left to react for 1 hour. Once the reaction had completed the reaction was cooled to room temperature, and diluted with dichloromethane (ca. 20 mL/mmol spirocycle). The solution was then passed through Celite® and then washed with DCM followed by EtOAc. The organic extracts were concentrated in vacuo to yield a yellow solid. The crude product was then purified by flash column chromatography (silica gel, 5% acetone:toluene) to yield the product **7** as a yellow solid, (366 mg, 81%, dr >30:1).

**$\delta_H$** (400 MHz, $CDCl_3$): 9.55 (1H, d, *J* 1.5 Hz, **H20**), 9.07 (1H, d, *J* 1.5 Hz, **H19**), 7.97 (2H, d, *J* 8.4 Hz, **H4**), 7.38 (2H, d, *J* 8.1 Hz, **H3**), 7.32-7.20 (5H, m, ***Ar***), 4.90-4.80 (1H, m, **H12**), 4.17 (1H, q, *J* 8.5 Hz, **H6'**), 4.06 (1H, t, *J* 7.1 Hz, **H22**), 3.98 (1H, td, *J* 9.0, 3.2 Hz, **H6**), 3.30 (1H, dd, *J* 12.7, 3.3 Hz, **H13'**), 2.82 (1H, dd, *J* 12.5, 11.2 Hz, **H13**), 2.69 (1H, t, *J* 8.1 Hz, **H24**), 2.59 (1H, ddd, *J* 12.9, 7.4, 3.2 Hz, **H7'**), 2.48 (3H, s, **H1**), 2.41 (1H, dd, *J* 14.0, 3.2 Hz, **H11'**) 2.20 (1H, quint, *J* 7.6 Hz, **H23**), 2.03 (1H, dt, *J* 13.0, 8.5 Hz, **H7**) , 1.93 (1H, dd, *J* 13.9, 8.9 Hz, **H11**); **$\delta_C$** (101 MHz, $CDCl_3$): 174.9 (**C25**), 172.1 (**C9**), 171.3 (**C10**), 145.5 (**C2/5**), 145.2 (**C18/21**), 142.6 (**C18/21**), 137.6 (**C14**), 135.9 (**C19**), 134.9 (**C2/5**), 134.4 (**C20**), 129.9 (**C3**), 129.7 (***Ar***), 128.7 (***Ar***), 128.3 (**C4**), 126.8 (***Ar***), 57.0 (**C12**), 56.3 (**C8**), 46.9 (**C22**), 45.4 (**C6**), 39.5 (**C13**), 33.2 (**C24**), 32.1 (**C11**), 31.1 (**C7**), 21.9 (**C1**), 18.0 (**C23**); **$[\alpha]_D^{20}$** -10.2 (*c* = 1.08, $CHCl_3$); **HRMS** (ESI+): found 560.1968; $C_{29}H_{29}N_5O_5SH$, $[M+H]^+$ requires 560.1962; **$\nu_{max}$ (neat)**: 3026.6, 2959.5, 2363.1, 1699.7, 1595.3, 1476.0, 1367.9, 1230.0, 1170.4.

(3*S*,5*R*)-3-Benzyl-2-(5-(2-oxopyrrolidin-1-yl)pyrazin-2-yl)-2,7-diazaspiro[4.4]nonane-1,6-dione (**8**)

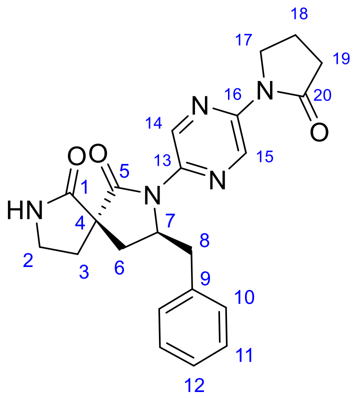

Prepared according to **General Procedure B** using **7** (1 eq.), TfOH (5 eq.) and DCE (0.04 M). Reaction time = 8 h. Purification by flash column chromatography (silica gel, 1→2% MeOH:DCM or 80→90% EtOAc:*n*-hexane) afforded the product **8** as a white solid, 100 mg SM (46 mg, 83%), 320 mg SM (126 mg, 63% (BRSM)). On the larger scale (320 mg) the reduction in yield was a result of Friedel–Crafts sulfonylation of the product, forming an undesired tosyl-transfer by-product, hence EtOAc:n-hexane eluent was used in place of DCM:MeOH, to remove this. This effect was also observed by Orentas *et al.*[9] as a result of the tosyl triflate byproduct being a potential sulfonaltion agent. It therefore may be of use to add a large excess of m-xylene to act as a tosyl triflate scavenger to minimise product loss.

**$\delta_H$** (600 MHz, $CDCl_3$): 9.57 (1H, d, *J* 1.4 Hz, **H15**), 9.25 (1H, d, *J* 1.3 Hz, **H14**), 7.37-7.34 (2H, m, ***Ar***), 7.33-7.30 (2H, m, ***Ar***), 7.25-7.21 (1H, m, ***Ar***), 5.96 (1H, s, **NH**), 4.94-4.88 (1H, m, **H7**), 4.09 (2H, t, *J* 7.1 Hz, **H17**), 3.70 (1H, q, *J* 7.8 Hz, **H2'**), 3.40-3.35 (2H, m, **H2+H8'**), 3.00 (1H, dd, *J* 12.6, 10.9 Hz, **H8**), 2.73-2.68 (1H, m, **H3'**), 2.70 (2H, t, *J* 8.1 Hz, **H19**), 2.51 (1H, dd, *J* 13.6, 3.5 Hz, **H6'**), 2.21 (2H, quint, *J* 7.6 Hz, **H18**), 2.09 (1H, dt, *J* 13.0, 8.0 Hz, **H3**), 1.99 (1H, dd, *J* 13.5, 8.7 Hz, **H6**); **$\delta_C$** (151 MHz, $CDCl_3$): 176.2 (**C1**), 174.9 (**C20**), 173.6 (**C5**), 145.0 (**C13/16**), 143.1 (**C13/16**), 138.1 (**C9**), 136.2 (**C14**), 134.4 (**C15**), 129.8 (***Ar***), 128.7 (***Ar***), 126.7 (***Ar***), 57.1 (**C7**), 54.1 (**C4**), 46.0 (**C17**), 40.0 (**C2**), 39.7 (**C8**), 34.1 (**C3**), 33.3 (**C19**), 32.9 (**C6**), 18.1 (**C18**); **$[\alpha]_D^{20}$** -24.6 (*c* = 0.80, $CHCl_3$); **HRMS** (ESI+): found 406.1883; $C_{22}H_{23}N_5O_3H$, $[M+H]^+$ requires 406.1874; **$\nu_{max}$ (thin film)**: 3248.4, 2924.1, 2245.7, 1684.8, 1466.7, 1353.0, 1263.6.

(3*S*,5*S*)-3-benzyl-2-(5-(2-oxopyrrolidin-1-yl)pyrazin-2-yl)-7-(pyridin-4-yl)-2,7-diazaspiro[4.4]nonane-1,6-dione (**14**)

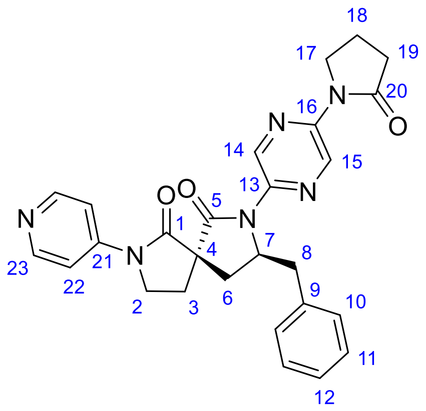

Prepared according to **General Procedure A** using **8** (27.0 mg, 0.067 mmol, 1 eq.), 4-bromopyridine hydrochloride (19.4 mg, 0.10 mmol, 1.5 eq.), $Pd_2(dba)_3$ (6.1 mg, 6.6 μmol, 10 mol%), Xantphos (11.6 mg, 0.020 mmol, 30 mol%), $Cs_2CO_3$ (75.9 mg, 0.23 mmol, 3.5 eq.), and toluene (1.0 mL). Reaction temperature = 80 °C and reaction time = 4 h. Purification by flash column chromatography (silica gel, 2% MeOH:DCM,) afforded the product as a glassy yellow solid, (24.0 mg, 75%).

**$\delta_H$** (600 MHz, $CDCl_3$): 9.59 (1H, d, *J* 1.4 Hz, **H15**), 9.21 (1H, d, *J* 1.4 Hz, **H14**), 8.60 (1H, d, *J* 6.3 Hz, **H23**), 7.69 (1H, d, *J* 6.4 Hz, **H22**), 7.40-7.36 (2H, m, **H10**), 7.33 (2H, t, *J* 7.5 Hz, **H11**), 7.24 (1H, t, *J* 7.2 Hz, **H12**), 5.00-4.93 (1H, m, **H7**), 4.23 (1H, q, *J* 8.3 Hz, **H2'**), 4.09 (2H, t, *J* 7.1 Hz, **H17**), 3.82 (1H, td, *J* 9.0, 3.1 Hz, **H2**), 3.41 (1H, dd, *J* 12.8, 3.4 Hz, **H8'**), 3.05 (1H, dd, *J* 12.7, 10.9 Hz, **H8**), 2.74 (1H, ddd, *J* 13.1, 7.6, 3.0 Hz, **H3'**), 2.70 (2H, t, *J* 8.0 Hz, **H19**), 2.61 (1H, dd, *J* 13.7, 3.2 Hz, **H6'**), 2.21 (2H, quint, *J* 7.7 Hz, **H18**), 2.14 (1H, dt, *J* 12.9, 8.6 Hz, **H3**), 2.08 (1H, dd, *J* 13.8, 8.9 Hz, **H6**); **$\delta_H$** (600 MHz, $d_6$-DMSO): 9.52 (1H, d, *J* 1.4 Hz, **H15**), 9.15 (1H, d, *J* 1.5 Hz, **H14**), 8.62 (2H, d, *J* 1.4 Hz, **H23**), 7.83 (2H, d, *J* 6.4 Hz, **H22**), 7.39 (2H, t, *J* 7.5 Hz, **H11**), 7.34 (2H, d, *J* 7.4 Hz, **H10**), 7.29 (2H, t, *J* 7.5 Hz, **H12**), 4.97-4.89 (1H, m, **H7**), 4.09 (1H, q, *J* 8.3 Hz, **H2'**), 4.04 (2H, t, *J* 7.6 Hz, **H17**), 3.97 (1H, td, 8.9, 3.4 Hz, **H2**). 3.34 (1H, dd, **H8'**),* 3.01 (1H, dd, *J* 13.0, 10.4 Hz, **H8**), 2.68 (2H, t, *J* 8.0 Hz, **H19**), 2.67-2.63 (1H, m, **H3'**), 2.47 (1H, dd, *J* 13.5, 4.0 Hz, **H6'**), 2.33-2.26 (2H, m, **H3+H6**), 2.17 (2H, quint, *J* 7.6 Hz, **H18**); **$\delta_C$** (151 MHz, $CDCl_3$): 174.9 (**C20**), 173.5 (**C1**), 172.6 (**C5**), 150.8 (**C23**), 145.8 (**C21**), 145.2 (**C13/16**), 142.8 (**C13/16**), 137.9 (**C9**), 136.1 (**C14**), 134.4 (**C15**), 129.8 (**C10**), 128.7 (**C11**), 126.8 (**C12**), 113.3 (**C22**), 57.1 (**C7**), 56.9 (**C4**), 47.0 (**C17**), 45.5 (**C2**), 39.7 (**C8**), 33.2 (**C19**), 32.8 (**C6**), 30.8 (**C3**), 18.1 (**C18**); **$[\alpha]_D^{20}$** +70.8 (*c* = 1.00, $CHCl_3$); **HRMS** (ESI+):

---

* Coupling constant not giving due to co-incidence with water peak

found 483.2144; $C_{27}H_{26}N_6O_3H$, $[M+H]^+$ requires 483.2139; $\nu_{max}$ **(thin film)**: 3378.8, 3026.6, 2924.1, 2247.6, 1686.6, 1586.0, 1466.7, 1354.9, 1310.2, 1220.7.

(*S*)-1-(6-Chloropyrimidin-4-yl)-4-isopropyl-3-((2-nitrophenyl)sulfonyl)imidazolidin-2-one (**16**)

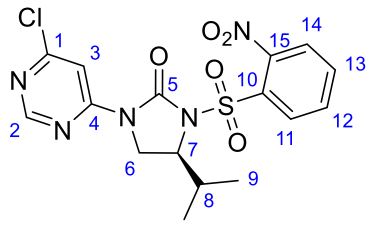


Prepared according to **General Procedure A** using (*S*)-5-isopropyl-1-((2-nitrophenyl)sulfonyl)imidazolidin-2-one (410 mg, 1.31 mmol, 1 eq.), 4,6-dichloropyrimidine (980 mg, 6.58 mmol, 5 eq.), $Pd_2(dba)_3$ (61 mg, 0.067 mmol, 5 mol%), Xantphos (111 mg, 0.19 mmol, 15 mol%), $Cs_2CO_3$ (1.04 g, 3.19 mmol, 2.5 eq.), and toluene (10 mL). Reaction time = 18 h. Purification by flash column chromatography (silica gel, 12.5%→17% EtOAc:Petroleum ether) afforded the product as a pale yellow solid, (318 mg, 57%).

$\delta_H$ (400 MHz, $CDCl_3$): 8.67 (1H, d, *J* 1.0 Hz, **H2**), 8.52-8.46 (1H, m, **H14**), 8.08 (1H, d, *J* 1.0 Hz, **H3**), 7.86-7.76 (3H, m, **H11-H13**), 4.49 (1H, m, **H7**), 4.16-4.05 (2H, m, **H6**), 2.47-2.36 (1H, m, **H8**), 1.07 (3H, d, *J* 7.0 Hz, **H9'**), 1.02 (3H, d, *J* 6.9 Hz, **H9**); $\delta_C$ (101 MHz, $CDCl_3$): 161.6 (**C1**), 158.1 (**C2**), 157.6 (**C4**), 151.2 (**C5**), 148.2 (**C15**), 135.4 (**C11/C12/C13**), 135.3 (**C14**), 132.3 (**C11/C12/C13**), 131.5 (**C10**), 124.8 (**C11/C12/C13**), 109.4 (**C3**), 59.8 (**C7**), 43.2 (**C6**), 32.6 (**C8**), 18.0 (**C9'**), 15.1 (**C9**); $[\alpha]_D^{20}$ 199.9 (*c* = 0.91, $CHCl_3$); **HRMS** (ESI+): found 426.0643; $C_{16}H_{16}ClN_5O_5SH$, $[M+H]^+$ requires 426.0634; $\nu_{max}$ **(thin film)**: 3140.3, 3103.0, 2965.1, 2875.6, 1736.9, 1561.8, 1537.5, 1455.5, 1395.9, 1358.6, 1228.2, 1172.2. These data are in agreement with previously reported values.[8]

### 1.2.5.2 Dimers

(3*S*,5*R*)-3-Benzyl-2-(5-((5*S*,8*S*)-8-benzyl-1,6-dioxo-7-(5-(2-oxopyrrolidin-1-yl)pyrazin-2-yl)-2,7-diazaspiro[4.4]nonan-2-yl)pyrazin-2-yl)-7-tosyl-2,7-diazaspiro[4.4]nonane-1,6-dione (**9**)

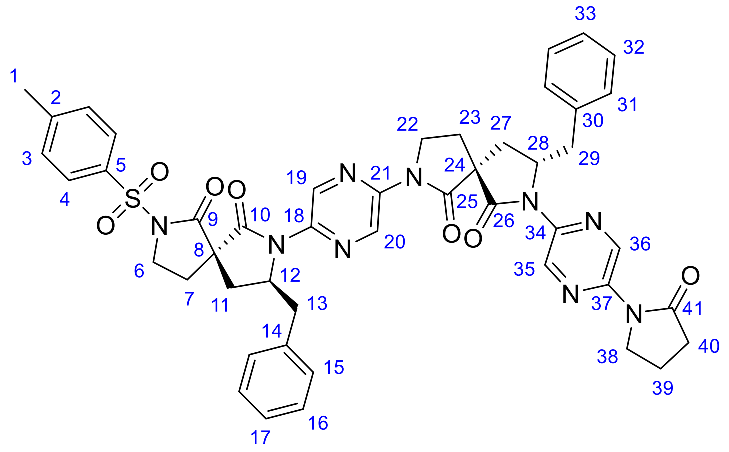

Prepared according to **General Procedure A** using **8** (87.0 mg, 0.21 mmol, 1 eq.), **6** (143 mg, 0.26 mmol, 1.2 eq.), $Pd_2(dba)_3$ (19.6 mg, 0.021 mmol, 10 mol%), Xantphos (37.2 mg, 0.064 mmol, 30 mol%), $Cs_2CO_3$ (175 mg, 0.54 mmol, 2.5 eq.), and toluene (2.2 mL). Reaction temperature = 80 °C and reaction time = 3 h. Purification by flash column chromatography (silica gel, 5 →10 →15% EtOAc:DCM) afforded the product **9** as a clear glassy solid (114 mg, 60%).

**$\delta_H$** (600 MHz, $CDCl_3$): 9.62 (1H, d, *J* 1.3 Hz, **H20**), 9.59 (1H, d, *J* 1.4 Hz, **H36**), 9.22 (1H, d, *J* 1.3 Hz, **H35**), 9.10 (1H, d, *J* 1.3 Hz, **H19**), 7.97 (2H, d, *J* 8.3 Hz, **H4**), 7.41-7.36 (4H, m, **H3+*Ar***), 7.34-7.31 (2H, m, ***Ar***), 7.30-7.27 (2H, m, ***Ar***), 7.26-7.20 (3H, m, ***Ar***), 5.00-4.94 (1H, m, **H28**), 4.89-4.83 (1H, m, **H12**), 4.29 (1H, dt, *J* 10.9, 7.9 Hz, **H22'**), 4.20-4.11 (2H, m, **H6'+H22**), 4.08 (1H, t, *J* 7.1 Hz, **H38**), 3.99 (1H, td, *J* 9.0, 3.0 Hz, **H6**), 3.44 (1H, dd, *J* 12.8, 3.4 Hz, **H29'**), 3.29 (1H, dd, *J* 12.8, 3.3 Hz, **H13'**), 3.04 (1H, dd, *J* 12.5, 11.0 Hz, **H29**), 2.84 (1H, dd, *J* 12.6, 11.1 Hz, **H13**), 2.74 (1H, ddd, *J* 12.9, 7.7, 3.4 Hz, **H23'**), 2.69 (1H, t, J 8.1 Hz, **H40**), 2.62 (1H, dd, *J* 13.8, 3.5 Hz, **H27'**), 2.60-2.57 (1H, m, **H7'**), 2.49 (3H, s, **H1**), 2.44 (1H, dd, *J* 13.9, 3.2 Hz, **H11'**), 2.20 (2H, quint, *J* 7.7 Hz, **H39**), 2.17-2.09 (2H, m, **H23+H27**), 2.05 (1H, dt, *J* 13.0, 8.5 Hz, **H7**), 1.95 (1H, *J* 13.9, 8.9 Hz, **H11**); **$\delta_C$** (151 MHz, $CDCl_3$): 174.9 (**C41**), 173.0 (**C25**), 172.7 (**C26**), 172.0 (**C9**), 171.4 (**C10**), 145.6 (**C2/5**), 145.2 (**C34/37**), 144.8 (**C18/21**), 143.1 (**C18/21**), 142.8 (**C34/37**), 137.9 (***Ar***), 137.6 (***Ar***), 136.2 (**C19**), 136.1 (**C35**), 134.8 (**C2/5**), 134.5 (**C20**), 134.4 (**C36**), 129.9 (**C3**), 129.8 (***Ar***), 129.7 (***Ar***), 128.8 (***Ar***), 128.7 (***Ar***), 128.3 (**C4**), 126.8 (***Ar***), 126.7 (***Ar***), 57.2 (**C24**), 57.1 (**C28**) 57.0 (**C12**), 56.3 (**C8**), 47.0 (**C38**), 45.4 (**C6**), 44.7 (**C22**), 39.7 (**C29**), 39.6 (**C13**), 33.2 (**C40**), 33.0 (**C27**), 32.1 (**C11**) 31.1 (**C7**), 31.0 (**C23**), 21.9 (**C1**), 18.1 (**C39**); **$[\alpha]_D^{20}$** +34.9 (*c* = 1.20, $CHCl_3$); **HRMS** (ESI+): found

880.3261; $C_{47}H_{45}N_9O_7SH$, $[M+H]^+$ requires 880.3234; **$\nu_{max}$ (thin film)**: 3026.6, 2955.8, 1690.3, 1466.7, 1351.2, 1230.0, 1172.2.

(3*S*,5*S*)-3-benzyl-2-(5-((5*S*,8*S*)-8-benzyl-1,6-dioxo-7-(5-(2-oxopyrrolidin-1-yl)pyrazin-2-yl)-2,7-diazaspiro[4.4]nonan-2-yl)pyrazin-2-yl)-2,7-diazaspiro[4.4]nonane-1,6-dione (**10**)

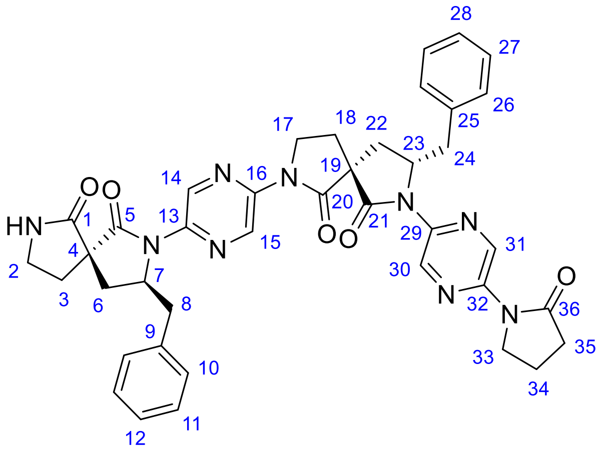

Prepared according to **General Procedure B** using **9** (1 eq.), TfOH (7 eq.) and DCE (0.04 M). Reaction time = 8 h. Purification by flash column chromatography (silica gel, 1→2% MeOH:DCM) afforded the product as a yellow glassy solid, 75 mg SM (42.7 mg, 87% (BRSM)), 113 mg SM (58.6 mg, 83% (BRSM)).

**$\delta_H$** (600 MHz, $CDCl_3$): 9.65 (1H, d, *J* 1.5 Hz, **H15**), 9.59 (1H, d, *J* 1.5 Hz, **H31**), 9.29 (1H, d, *J* 1.4 Hz, **H14**), 9.24 (1H, d, *J* 1.5 Hz, **H30**), 7.40-7.36 (2H, m, ***Ar***), 7.36-7.29 (6H, m, ***Ar***) 7.26-7.21 (2H, m, ***Ar***), 6.38 (1H, s, **NH**), 5.00-4.95 (1H, m, **H23**), 4.94-4.88 (1H, m, **H7**), 4.31 (1H, dt, *J* 10.9, 7.8 Hz, **H17'**), 4.20-4.14 (1H, m, **H17**), 4.08 (1H, t, *J* 7.2 Hz, **H33**), 3.71 (1H, q, *J* 8.0 Hz, **H2'**), 3.45 (1H, dd, *J* 12.8, 3.4 Hz, **H24'**), 3.41-3.35 (2H, m, **H2+H8'**), 3.06 (1H, dd, *J* 12.6, 10.9 Hz, **H24**), 3.02 (1H, dd, *J* 9.2, 7.4 Hz, **H8**), 2.78-2.68 (2H, m, **H3'+H18'**), 2.69 (1H, t, *J* 8.0 Hz, **H35**), 2.64 (1H, dd, *J* 13.7, 3.5 Hz, **H22'**), 2.53 (1H, dd, *J* 13.6, 3.7 Hz, **H6'**), 2.20 (2H, quint, *J* 7.6 Hz, **H34**), 2.17-2.06 (3H, m, **H3+H18+H22**), 2.01 (1H, dd, *J* 13.6, 8.7 Hz, **H6**); **$\delta_C$** (151 MHz, $CDCl_3$): 176.2 (**C1**), 174.9 (**C36**), 173.7 (**C5**), 173.0 (**C20**), 172.8 (**C21**), 145.1 (**C29/32**), 144.6 (**C13/16**), 143.5 (**C13/16**), 142.9 (**C29/32**), 138.1 (***Ar***), 137.9 (***Ar***), 136.4 (**C14**), 136.1 (**C30**), 134.4 (**C14**), 134.3, (**C31**), 129.7 (***Ar***), 128.7 (***Ar***) 128.6 (***Ar***), 126.7 (***Ar***), 126.6 (***Ar***), 57.1 (**C7**), 57.0 (**C23**), 54.2 (**C4**), 47.0 (**C33**), 44.7 (**C17**), 40.0 (**C2**), 39.7 (**C24**), 39.6 (**C8**), 34.0 (**C3**), 33.2 (**C35**), 33.0 (**C22**), 32.9 (**C6**), 31.0 (**C18**), 18.0 (**C34**);* **$[\alpha]_D^{20}$** +17.0 (*c* = 0.84, $CHCl_3$); **HRMS** (ESI+): found 726.3150; $C_{40}H_{39}N_9O_5H$, $[M+H]^+$ requires 726.3147;

* **C19** and one non-quaternary aromatic carbon not detected due to presumed overlap.

**$\nu_{max}$ (thin film)**: 3280.1, 2953.9, 2363.1, 2113.4, 1466.7, 1686.6, 1466.7, 1351.2, 1261.7, 1177.8.

(3*S*,5*S*)-3-benzyl-2-(5-((5*S*,8*S*)-8-benzyl-1,6-dioxo-7-(5-(2-oxopyrrolidin-1-yl)pyrazin-2-yl)-2,7-diazaspiro[4.4]nonan-2-yl)pyrazin-2-yl)-7-(pyridin-4-yl)-2,7-diazaspiro[4.4]nonane-1,6-dione (**15**)

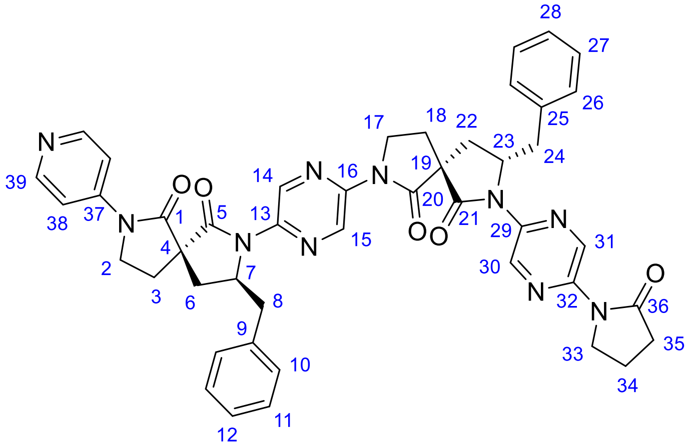


Prepared according to **General Procedure A** using **10** (26.3 mg, 0.036 mmol, 1 eq.), 4-bromopyridine hydrochloride (10.8 mg, 0.056 mmol, 1.5 eq.), $Pd_2(dba)_3$ (3.3 mg, 3.6 μmol, 10 mol%), Xantphos (6.3 mg, 0.011 mmol, 30 mol%), $Cs_2CO_3$ (42.0 mg, 0.13 mmol, 3.6 eq.), and toluene (1.0 mL). Reaction temperature = 80 °C and reaction time = 3 h. Purification by flash column chromatography (silica gel, 2% MeOH:DCM,) afforded the product **15** as a yellow solid, (17.0 mg, 58%).

**$\delta_H$** (600 MHz, $CDCl_3$): 9.66 (1H, d, *J* 1.4 Hz, **H15**), 9.59 (1H, d, *J* 1.4 Hz, **H31**), 9.25 (1H, d, *J* 1.4 Hz, **H14**), 9.23 (1H, d, *J* 1.4 Hz, **H30**), 8.61 (1H, d, *J* 5.4 Hz, **H39**), 7.70 (1H, d, *J* 5.2 Hz, **H38**), 7.40-7.36 (4H, m, ***Ar***), 7.35-7.29 (4H, m, ***Ar***), 7.26-7.21 (2H, m, ***Ar***), 5.03-4.95 (2H, m, **H7+H23**), 4.31 (1H, dt, *J* 10.9, 7.8 Hz, **H17**), 4.24 (1H, q, *J* 8.4 Hz, **H2'**), 4.19-4.14 (1H, m, **H17**), 4.09 (1H, t, *J* 7.2 Hz, **H33**), 3.84 (1H, td, *J* 9.1, 3.1 Hz, **H2**), 3.46 (1H, dd, *J* 12.8, 3.5 Hz, **H24'**), 3.41 (1H, dd, *J* 12.9, 3.4 Hz, **H8'**), 3.11-3.00 (2H, m, **H8+H24**), 2.79-2.72 (2H, m, **H3**+**H18**), 2.70 (2H, t, *J* 8.1 Hz, **H35**), 2.64 (2H, m, **H6'+H22'**), 2.20 (2H, quint, *J* 7.5 Hz, **H34**) 2.20-2.07 (4H, m, **H3+H6+H18+H22**); **$\delta_H$** (600 MHz, $d_6$-DMSO): 9.52 (1H, d, *J* 1.5 Hz, **H15**), 9.47 (1H, d, *J* 1.4 Hz, **H31**), 9.15 (1H, d, *J* 1.4 Hz, **H14**), 9.10 (1H, d, *J* 1.4 Hz, **H30**), 8.56 (1H, d, *J* 6.3 Hz, **H39**), 7.78 (1H, d, *J* 6.2 Hz, **H38**), 7.36-7.28 (8H, m, ***Ar***), 7.26-7.22 (2H, m, ***Ar***), 4.93-4.86 (2H, m, **H7+23**), 4.13 (1H, dt, *J* 10.5, 7.7 Hz, **H17'**), 4.09-4.02 (2H, m, **H2'+H17**), 3.98 (2H, t, *J* 7.00 Hz, **H33**), 3.92 (1H, dd, *J* 8.9, 4.2 Hz, **H2**), 3.32 (2H, m, **H8'+H24'**),* 3.03-2.93 (2H, m, **H8'+H24'**) 2.62 (2H, t, *J* 8.1 Hz, **H35**), 2.66-2.58 (2H, m, **H3'+H17'**), 2.47 (1H, dd, *J* 13.5, 4.1 Hz, **H22'**, 2.44 (1H, dd, *J* 13.6, 4.0 Hz, **H6'**), 2.30-2.22 (4H, **H3+H6+H18+H22**), 2.11 (2H, *J* 7.7 Hz, **H34**); **$\delta_C$** (151 MHz, $CDCl_3$): 174.9 (**C36**), 173.4 (**C1**), 173.0 (**C20**), 172.8

---

* Peak at 3.32 (**H8'/24'**) is obscured by residual water peak, however, it's interaction with 3.03-2.93 is visible via COSY.

(**C5/21**), 172.7 (**C5/21**), 150.9 (**C39**), 145.8 (**C37**), 145.2 (**C29/32**), 144.8 (**C13/16**), 143.3 (**C13/16**), 142.9 (**C29/32**), 137.9 (***Ar$_q$***), 137.8 (***Ar$_q$***), 136.4 (**C14**), 136.1 (**C30**), 134.6 (**C15**), 134.4 (**C31**), 129.8 (***Ar***), 128.8 (***Ar***), 126.8 (***Ar***), 126.7 (***Ar***), 113.3 (**C38**), 57.19 (**C7/23**), 57.17 (**C19**), 57.09 (**C7/23**), 56.9 (**C4**), 47.0 (**C33**), 45.5 (**C2**), 44.7 (**C17**), 39.8 (**C8/24**), 39.7 (**C8/24**), 33.3 (**C35**), 33.0 (**C6/22**), 32.9 (**C6/22**), 31.0 (**C18**), 30.8 (**C3**), 18.0 (**C34**);* **[α]$_D^{20}$** +24.3 (*c* = 1.06, $CHCl_3$); **HRMS** (ESI+): found 803.3412; $C_{45}H_{42}N_{10}O_5H$, $[M+H]^+$ requires 803.3413; **ν$_{max}$ (thin film)**: 2920.4, 2249.4, 1686.6, 1466.7, 1353.0, 1224.4.

### *1.2.5.3 Trimers*

(3*S*,5*S*)-3-Benzyl-7-(5-((3*S*,5*S*)-3-benzyl-7-(5-((3*S*,5*R*)-3-benzyl-1,6-dioxo-7-tosyl-2,7-diazaspiro[4.4]nonan-2-yl)pyrazin-2-yl)-1,6-dioxo-2,7-diazaspiro[4.4]nonan-2-yl)pyrazin-2-yl)-2-(5-(2-oxopyrrolidin-1-yl)pyrazin-2-yl)-2,7-diazaspiro[4.4]nonane-1,6-dione (**11**)

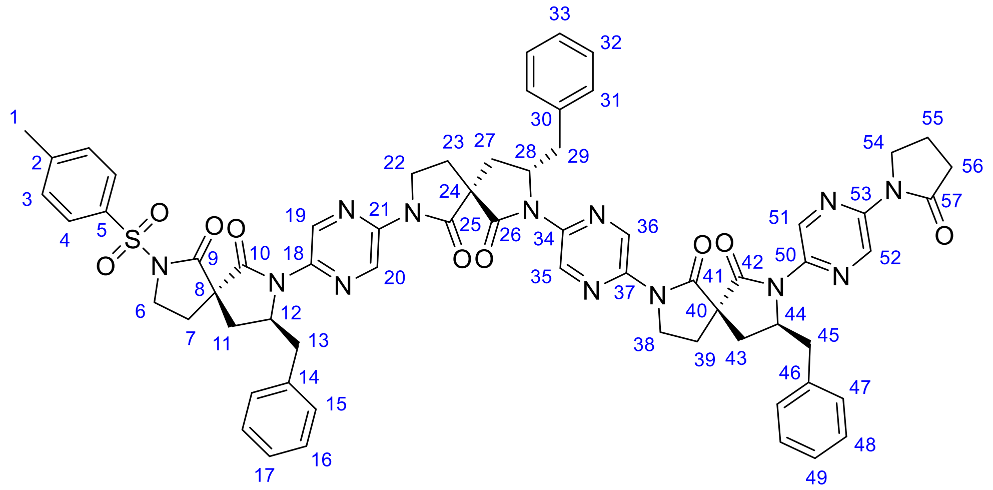

Prepared according to **General Procedure A** using **10** (56.7 mg, 0.078 mmol, 1 eq.), **6** (52.1 mg, 0.094 mmol, 1.20 eq.), $Pd_2(dba)_3$ (7.2 mg, 7.9 μmol, 10 mol%), Xantphos (13.6 mg, 0.024 mmol, 30 mol%), $Cs_2CO_3$ (63.6 mg, 0.20 mmol, 2.5 eq.), and toluene (1 mL). Reaction temperature = 80 °C and reaction time = 1.5 h. Purification by flash column chromatography (silica gel, 5 →15 →20% EtOAc:DCM) afforded the product **11** as a clear glassy solid, (64.7 mg, 69%).

**δ$_H$** (600 MHz, $CDCl_3$): 9.67 (1H, d, *J* 1.5 Hz, **H36**), 9.64 (1H, d, *J* 1.5 Hz, **H20**), 9.59 (1H, d, *J* 1.5 Hz, **H52**), 9.26 (1H, d, *J* 1.5 Hz, **H35**), 9.23 (1H, d, *J* 1.5 Hz, **H51**), 9.10 (1H, d, *J* 1.5 Hz, **H19**), 7.98 (1H, d, *J* 8.3 Hz, **H4**), 7.40 (2H, d, *J* 8.3 Hz, **H3**), 7.39-7.36 (4H, m, ***Ar***), 7.35-7.28 (8H, m, ***Ar***), 7.27-7.21 (3H, m, ***Ar***), 5.01-4.95 (2H, m, **H28 + H44**), 4.90-4.83 (1H, m, **H12**), 4.35-4.27 (2H, m, **H22' + H38'**), 4.21-4.13 (3H, m, **H6' + H22 + H38**), 4.08 (2H, t, *J* 7.2 Hz, **H54**), 4.00 (1H, td, *J* 9.1, 3.1 Hz, **H6**), 3.44 (2H, m, **H29'+ H45'**), 3.29 (1H, dd, *J* 12.9, 3.2 Hz,

---

* Two non-quaternary phenyl carbons were not detected due to presumed overlap.

**H13’**), 3.09-3.01 (2H, m, **H29 + H45**), 2.84 (1H, dd, *J* 12.5, 11.1 Hz, **H13**), 2.78-2.72 (2H, m, **H23’ + H39’**), 2.70 (2H, t, *J* 8.1 Hz, **H56**), 2.66 (1H, dd, *J* 6.3, 3.6 Hz, **H27/43**), 2.63 (1H, dd, *J* 6.2, 3.6 Hz, **H27/43**), 2.59 (1H, ddd, *J* 12.9, 7.4, 3.1 Hz, **H7’**), 2.49 (3H, s, **H1**), 2.45 (1H, dd, *J* 13.9, 3.2 Hz, **H11’**), 2.20 (2H, quint, *J* 7.6 Hz, **H55**), 2.18-2.08 (4H, m, **H23 + H27 + H39 + H43**), 2.05 (1H, dt, *J* 13.1, 8.5 Hz, **H7**), 1.95 (1H, dd, *J* 13.9, 8.9 Hz, **H11**); **$\delta_C$** (151 MHz, $CDCl_3$): 174.9 (**C57**), 173.0 (**C=O**), 172.9 (**C=O**), 172.8 (**C=O**), 172.7 (**C=O**), 172.0 (**C9**), 171.4 (**C10**), 145.6 (**C2/5**), 145.2 (**C50/53**), 144.8 (**C18/21 + C34/37**), 143.4 (**C34/37**), 143.1 (**C18/21**), 142.9 (**C50/53**), 137.9 (***$Ar_q$***), 137.8 (***$Ar_q$***), 137.6 (***$Ar_q$***), 136.4 (**C35**), 136.2 (**C19**), 136.1 (**C51**), 134.9 (**C2/5**), 134.56 (**C20/36**), 134.54 (**C20/36**), 134.4 (**C52**), 129.9 (**C3**), 129.8 (***Ar***), 129.7 (***Ar***), 128.8 (***Ar***), 128.7 (***Ar***), 128.3 (**C4**), 126.82 (***Ar***), 126.81 (***Ar***), 126.80 (***Ar***), 57.17 (**C24/40**), 57.15 (**C12/28/44**), 57.10 (**C12/28/44**), 57.06 (**C12/28/44**), 56.3 (**C8**), 47.0 (**C54**), 45.5 (**C6**), 44.7 (**C22/38**), 44.7 (**C22/38**), 39.8 (**C29/45**), 39.6 (**C13**), 33.3 (**C56**), 33.0 (**C27/43**), 32.1 (**C11**), 31.1 (**C7**), 31.0 (**C23/39**), 21.9 (**C1**), 18.1 (**C55**);* **$[\alpha]_D^{20}$** +82.2 (*c* = 0.91, $CHCl_3$); **HRMS** (ESI+): found 1200.4514; $C_{65}H_{61}N_{13}O_9SH$, $[M+H]^+$ requires 1200.4509; **$\nu_{max}$ (thin film)**: 3026.6, 2853.3, 2249.4, 1686.6, 1464.8, 1347.4, 1230.0, 1172.2, 1088.4.

---

* Several individual alkyl (**C23/C39, C24/C40, C28/C44 & C29/45**) and two non-quaternary phenyl $^{13}C$ peaks were not detected in the spectrum due to presumed overlap.

(3*S*,5*S*)-3-benzyl-7-(5-((3*S*,5*S*)-3-benzyl-7-(5-((3*S*,5*S*)-3-benzyl-1,6-dioxo-2,7-diazaspiro[4.4]nonan-2-yl)pyrazin-2-yl)-1,6-dioxo-2,7-diazaspiro[4.4]nonan-2-yl)pyrazin-2-yl)-2-(5-(2-oxopyrrolidin-1-yl)pyrazin-2-yl)-2,7-diazaspiro[4.4]nonane-1,6-dione (**12**)

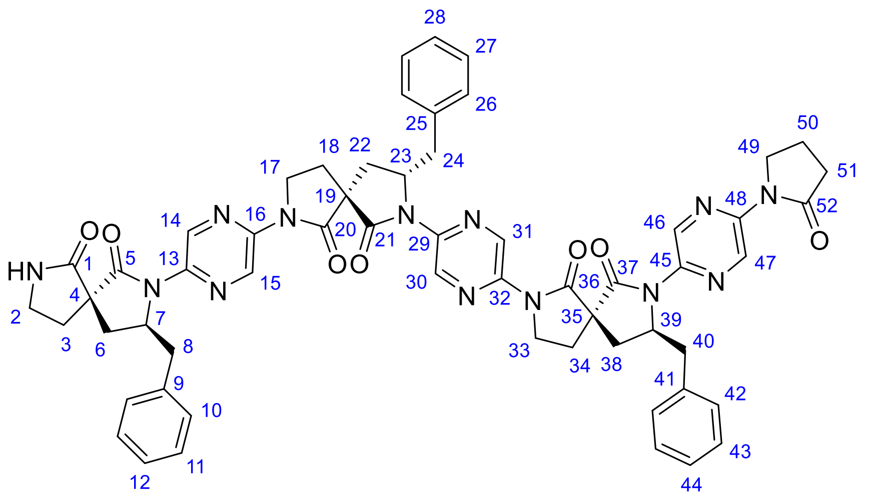

Prepared according to **General Procedure B** using **11** (1 eq.), TfOH (9 eq.), m-xylene (10 eq.) and DCE (0.04 M). Reaction time = 8 h. Purification by flash column chromatography (silica gel, 1→3% MeOH:DCM) afforded the product **12** as a clear glassy solid, 62.5 mg SM (38.4 mg, 71%).

**$\delta_H$** (600 MHz, $CDCl_3$): 9.67 (2H, d, *J* 1.2 Hz, **H31**), 9.66 (2H, d, *J* 1.2 Hz, **H15**), 9.59 (2H, d, *J* 1.3 Hz, **H47**), 9.28 (2H, d, *J* 1.3 Hz, **H14**), 9.27 (2H, d, *J* 1.3 Hz, **H30**), 9.24 (2H, d, *J* 1.2 Hz, **H46**), 7.40-7.37 (4H, m, ***Ar***), 7.36-7.29 (8H, m, ***Ar***), 7.25-7.21 (3H, m, ***Ar***), 6.12 (1H, s, **NH**), 5.02-4.95 (2H, m, **H23+H39**), 4.95-4.88 (1H, m, **H7**), 4.36-4.26 (2H, m, **H17' + H33'**), 4.22-4.13 (2H, m, **H17 + H33**), 4.08 (2H, t, *J* 7.2 Hz, **H49**), 3.71 (1H, q, *J* 8.1 Hz **H2'**), 3.49-3.42 (2H, m, **H24' + H40'**), 3.41-3.35 (2H, m, **H2 + H8'**), 3.10-2.99 (3H, m, **H8 + H24 + H40**), 2.79-2.67 (3H, m**, H3'+ H18' + H34'**), 2.70 (2H, t, *J* 8.2 Hz, **H51**), 2.68-2.62 (2H, m, **H22'+H38'**), 2.54 (1H, dd, *J* 13.5, 3.6 Hz, **H6'**), 2.20 (2H, quint, *J* 7.7 Hz, **H50**), 2.18-2.07 (5H, m, **H3+ H18 + H22 + H34 + H38**), 2.01 (1H, dd, *J* 13.5, 8.8 Hz, **H6**); **$\delta_C$** (151 MHz, $CDCl_3$): 176.1 (**C1**), 174.9 (**C52**), 173.7 (**C5**), 173.0 (**C=O**), 172.9 (**C=O**), 172.9 (**C=O**), 172.8 (**C=O**), 145.1 (**C45/48**), 144.8 (**C13/16 or C29/32**), 144.6 (**C13/16 or C29/32**), 143.6 (**C13/16 or C29/32**), 143.4 (**C13/16 or C29/32**), 142.9 (**C45/48**), 138.1 (***Ar$_q$***), 137.9 (***Ar$_q$***), 136.4 (**C14/30**), 136.3 (**C14/30**), 136.1 (**C46**), 134.5 (**C15+C31**), 134.4 (**C47**), 129.8 (***Ar***), 128.8 (***Ar***), 128.7 (***Ar***), 126.8 (***Ar***), 126.7 (***Ar***), 57.18 (**C7/23/39**), 57.17 (**C19/35**), 57.16 (**C19/35**), 57.15 (**C7/23/39**), 57.09 (**C7/23/39**), 54.1 (**C4**), 47.0 (**C49**), 44.7 (**C17/C33**), 44.6 (**C17/C33**), 40.0 (**C2**), 39.8 (**C8/24/40**), 39.7 (**C8/24/40**), 34.1 (**C3**), 33.2 (**C51**), 33.1 (**C22/38**), 33.0 (**C22/38**), 32.9 (**C6**),

31.0 (**C18/34**), 18.1 (**C50**);* **[α]$_D$$^{20}$** +67.1 (*c* =1.00, $CHCl_3$); **HRMS** (ESI+): found 1046.4427; $C_{58}H_{55}N_{13}O_7H$, $[M+H]^+$ requires 1046.4420; **$\nu_{max}$ (thin film)**: 3026.6, 2963.2, 2247.6, 1688.5, 1466.7, 1351.2, 1259.8, 1176.0.

(3*S*,5*S*)-3-benzyl-7-(5-((3*S*,5*S*)-3-benzyl-7-(6-((*S*)-4-isopropyl-3-((2-nitrophenyl)sulfonyl)-2-oxoimidazolidin-1-yl)pyrimidin-4-yl)-1,6-dioxo-2,7-diazaspiro[4.4]nonan-2-yl)pyrazin-2-yl)-2-(5-(2-oxopyrrolidin-1-yl)pyrazin-2-yl)-2,7-diazaspiro[4.4]nonane-1,6-dione (**17**)

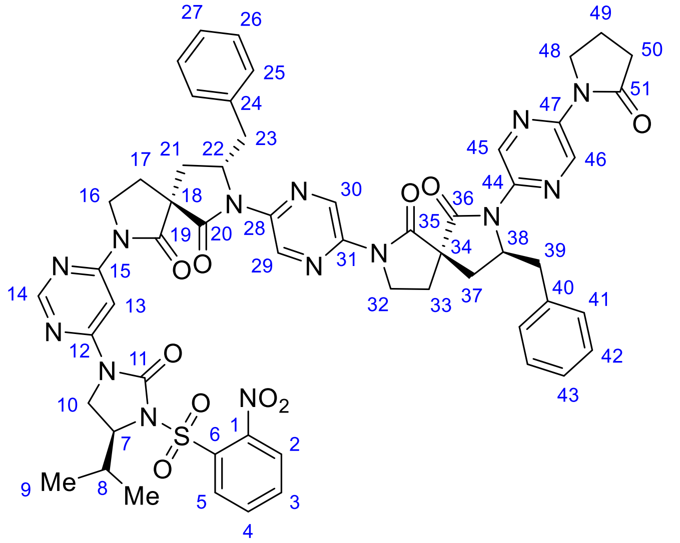

Prepared according to **General Procedure A** using **10** (28.0 mg, 0.039 mmol, 1 eq.), **16** (20.4 mg, 0.048 mmol, 1.20 eq.), $Pd_2(dba)_3$ (3.7 mg, 4.0 μmol, 10 mol%), Xantphos (6.9 mg, 0.012 mmol, 30 mol%), $Cs_2CO_3$ (27.5 mg, 0.10 mmol, 2.5 eq.), and toluene (1 mL). Reaction temperature = 80 °C and reaction time = 2 h. Purification by flash column chromatography (silica gel, 1→2% MeOH:DCM) afforded the product **17** as a yellow glassy solid, (32.4 mg, 75%).

**$\delta_H$** (600 MHz, $CDCl_3$): 9.66 (1H, d, *J* 1.4 Hz, **H30**), 9.59 (1H, d, *J* 1.4 Hz, **H46**), 9.24 (1H, d, *J* 1.4 Hz, **H45**), 9.20 (1H, d, *J* 1.4 Hz, **H29**), 9.04 (1H, d, *J* 1.0 Hz, **H13**), 8.69 (1H, d, *J* 1.0 Hz, **H14**), 8.56 (1H, m, **HNs**), 7.82-7.73 (3H, m, **HNs**), 7.40-7.37 (2H, m, ***Ar***), 7.36-7.29 (6H, m, ***Ar***), 7.26-7.22 (2H, m, ***Ar***), 5.01-4.91 (2H, m, **H22 + H38**), 4.50-4.46 (1H, m, **H7**), 4.33 (1H, dt, *J* 10.8, 7.8 Hz, **H32**), 4.27 (1H, dt, *J* 11.0, 7.9 Hz, **H16'**), 4.20-4.12 (3H, m, **H16 + H32 + H10'**), 4.11-4.06 (3H, m, **H10 + H48**), 3.46 (1H, dd, *J* 12.7, 3.3 Hz, **H39**), 3.40 (1H, dd, *J* 12.9,

* Several individual alkyl (**C8/24/40 & C18/34)** and aromatic peaks (one pyrazine & four phenyl) are not detected in the spectrum due to presumed overlap.

3.3 Hz, **H23**), 3.05 (1H, dd, *J* 12.6, 10.9 Hz, **H39**), 3.00 (1H, dd, *J* 12.8, 10.8 Hz, **H23**), 2.78-2.73 (1H, m, **H33'**), 2.70 (1H, t, *J* 8.0 Hz, **H49**), 2.68-2.62 (2H, m, **H17'+ H37'**), 2.60 (1H,dd, *J* 13.7, 3.8 Hz, **H21'**), 2.45-2.37 (1H, m, **H8**), 2.21 (2H, quint, *J* 7.6 Hz, **H50**), 2.18-2.01 (4H, m, **H17+ H33 + H21 + H37**), 1.07 (3H, d, *J* 7.0 Hz, **H9'**), 1.02 (3H, d, *J* 6.9 Hz, **H9**); **$\delta_C$** (151 MHz, $CDCl_3$): 174.9 (**C51**), 173.9 (**C19/35**), 173.0 (**C19/35**), 172.8 (**C20/36**), 172.5 (**C20/36**), 157.8 (**C12/15**), 157.7 (**C12/15**), 157.5 (**C14**), 151.1 (**C11**), 148.2 (**C1/6**), 145.2 (**C44/47**), 144.8 (**C28/31**), 143.3 (**C28/31**), 142.9 (**C44/47**), 137.9 (**C24/40**), 137.8 (**C24/40**), 136.5 (**C29**), 136.1 (**C45**), 135.6 (**C2-5**), 135.0 (**C2-5**), 134.6 (**C30**), 134.4 (**C46**), 132.4 (**C2-5**), 131.8 (**C1/6**), 129.8 (***Ar***), 129.7 (***Ar***), 128.8 (***Ar***), 128.7 (***Ar***), 126.8 (***Ar***), 126.7 (***Ar***), 124.5 (**C2-5**), 98.1 (**C13**), 59.6 (**C7**), 57.4 (**C18**), 57.2 (**C34**), 57.1 (**C22+C38**), 47.0 (**C48**), 44.9 (**C16**), 44.7 (**C32**), 43.2 (**C10**), 39.9 (**C23**), 39.8 (**C39**), 33.3 (**C50**), 33.0 (**C21 + C37**), 32.6 (**C8**), 31.0 (**C33**), 30.6 (**C17**), 18.1 (**C49**), 18.0 (**C9'**), 15.1 (**C9**);* **$[\alpha]_D^{20}$** +18.1 (*c* = 0.93, $CHCl_3$); **HRMS** (ESI+): found 1115.3925; $C_{56}H_{54}N_{14}O_{10}SH$, $[M+H]^+$ requires 1115.3941; **$\nu_{max}$ (thin film)**: 3695.7, 2968.8, 2922.2, 2868.2, 2844.0, 2251.3, 1690.3, 1541.3, 1464.8, 1353.0, 1259.8, 1172.2.

* **C21/C37 & C22/38** peaks are not detected due to presumed overlap

### 1.2.5.4 pseudo-hexamer

(3*S*,3'*S*,5*S*,5'*S*)-7,7'-(((3*S*,3'*S*,5*S*,5'*S*)-(((3*S*,3'*S*,5*S*,5'*S*)-pyrazine-2,5-diylbis(3-benzyl-1,6-dioxo-2,7-diazaspiro[4.4]nonane-7,2-diyl))bis(pyrazine-5,2-diyl))bis(3-benzyl-1,6-dioxo-2,7-diazaspiro[4.4]nonane-7,2-diyl))bis(pyrazine-5,2-diyl))bis(3-benzyl-2-(5-(2-oxopyrrolidin-1-yl)pyrazin-2-yl)-2,7-diazaspiro[4.4]nonane-1,6-dione) (**13**)

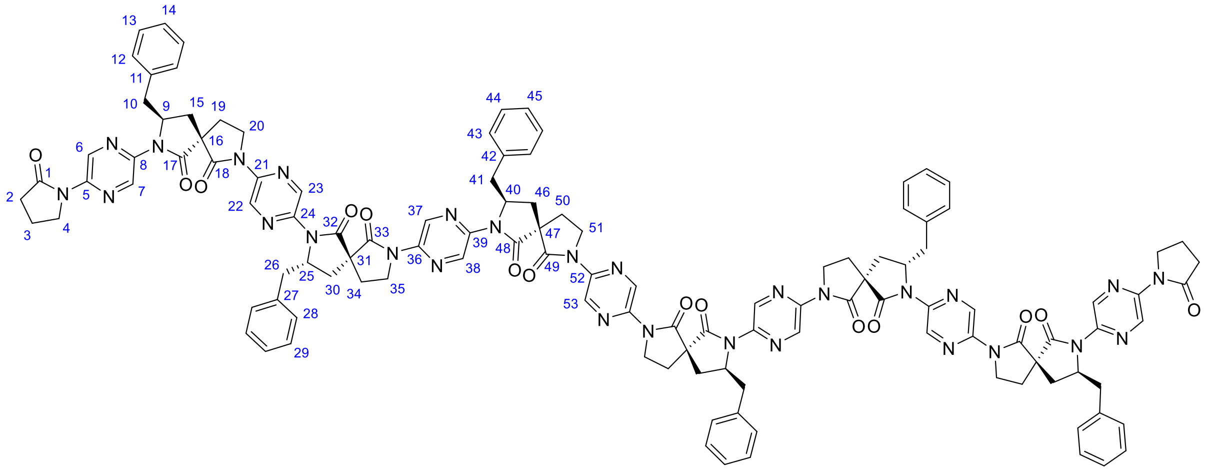

Prepared according to **General Procedure A** using **10** (34 mg, 0.033 mmol, 1 eq.), 2,5-dibromopyrazine (3.9 mg, 0.016 mmol, 0.5 eq.), $Pd_2(dba)_3$ (1.0 mg, 3.3 μmol, 10 mol%), Xantphos (5.0 mg, 9.8 μmol, 30 mol%), $Cs_2CO_3$ (26.5 mg, 0.081 mmol, 2.5 eq.), and toluene (1.0 mL). Reaction temperature = 80 °C and reaction time = 2 h. Purification by flash column chromatography (silica gel, 15% EtOAc:DCM then 2% MeOH:DCM) afforded the product **13** as a yellow solid (21.1 mg, 60%).

**$\delta_H$** (600 MHz, $CDCl_3$): 9.68 (2H, d, *J* 1.3 Hz, **H22/H37**), 9.67 (2H, d, *J* 1.3 Hz, **H22/H37**), 9.59 (2H, d, *J* 1.3 Hz, **H6**), 9.54 (2H, s, **H53**), 9.29 (2H, d, *J* 1.3 Hz, **H23/H38**), 9.28 (2H, d, *J* 1.3 Hz, **H23/H38**), 9.24 (2H, d, *J* 1.3 Hz, **H7**), 7.40-7.31 (24H, m, ***Ar***), 7.27-7.23 (6H, m, ***Ar***), 5.05-4.93 (6H, m, **H9+H25+40**), 4.38-4.26 (6H, m, **H20'+H35'+H51'**), 4.23-4.14 (6H, m, **H20+H35+H51**), 4.08 (4H, t, *J* 7.3 Hz, **H4**), 3.48-3.41 (6H, m, **H10'+H26'+H41'**), 3.10-3.00 (6H, m, **H10+H26+H41**), 2.80-2.72 (6H, m, **H19'+H34'+H50'**), 2.70 (4H, t, *J* 8.0 Hz, **H2**), 2.68-2.61 (6H, m, **H15'+H30'+H46'**), 2.20 (4H, quint, *J* 7.5 Hz, **H3**), 2.19-2.08 (12H, m, **H15+H30+H46 + H19+H34+H50**);* **$\delta_C$** (151 MHz, $CDCl_3$): 174.9 (**C1**), 173.0 (**C=O**), 172.92 (**C=O**), 172.91 (**C=O**), 172.80 (**C=O**), 172.7 (**C=O**), 145.2 (**C5/8**), 144.8 (**C21/24 + C36/39**), 144.5 (**C52**), 143.4 (**C21/24 or C36/39**), 143.3 (**C21/24 or C36/39**), 142.9 (**C5/8**), 137.9 (***Ar$_q$***), 136.5 (**C23/38**), 136.4 (**C23/38**), 136.1 (**C7**), 134.5 (**C22+C37**), 134.4 (**C6**), 134.3 (**C53**), 129.8 (***Ar***),

* Assignment of pyrazine peak H22/35 vs H23/H38 has been based on the assignment of the analogous peaks in dimers **9/10** and trimers **11/12** as definitive assignment cannot be made due to overlap of key interactions in ROESY spectrum

128.7 (***Ar***), 126.8 (***Ar***), 57.18 (**C16/C31/C47**), 57.16 (**C9/C25/C40**), 57.11 (**C9/C25/C40**), 57.10 (**C9/C25/C40**), 47.0 (**C4**), 44.7 (**C20/C35/C51**), 44.6 (**C20/C35/C51**), 39.8 (**C10/C26/C41**), 33.4 (**C15/C30/C46**), 33.3 (**C2**), 33.1 (**C15/C30/C46**), 33.0 (**C15/C30/C46**), 31.0 (**C19/C34/C50**), 18.1 (**C3**);* **$[\alpha]_D^{20}$** +179.1 (*c* = 1.28, $CHCl_3$); **$\nu_{max}$ (thin film)**: 2933.4, 2342.6, 2253.2, 1686.6, 1464.8, 1351.2, 1176.0; **MS** (MALDI-TOF): found 2168.9; $C_{120}H_{111}N_{28}O_{14}$, $[M+H]^+$ requires 2168.9; found 2190.9; $C_{120}H_{110}N_{28}O_{14}Na$, $[M+Na]^+$ requires 2190.9.

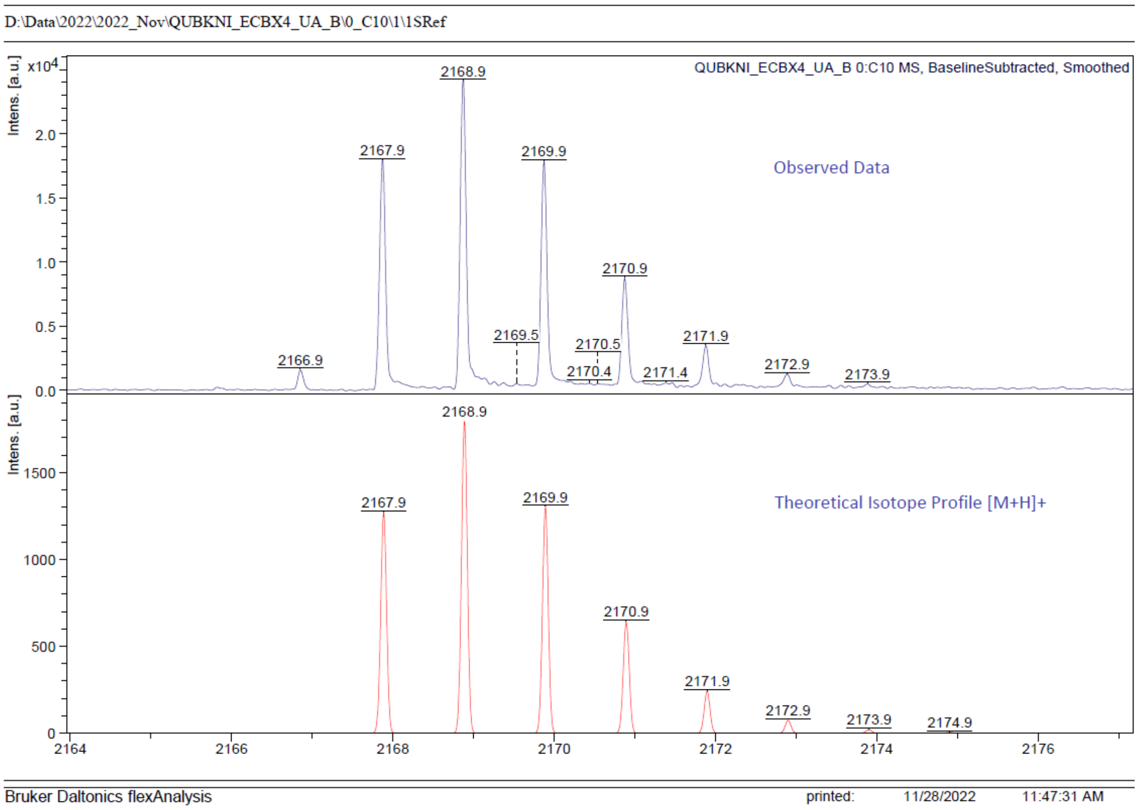


---

* Several alkyl (**C9/C25/C40, C10/26/41, C15/C30/C46 & C19/C34/C50**) and aromatic peaks (**C22+C37, C21/24 + C36/39** & two sets of Ph) are not detected due to presumed overlap.

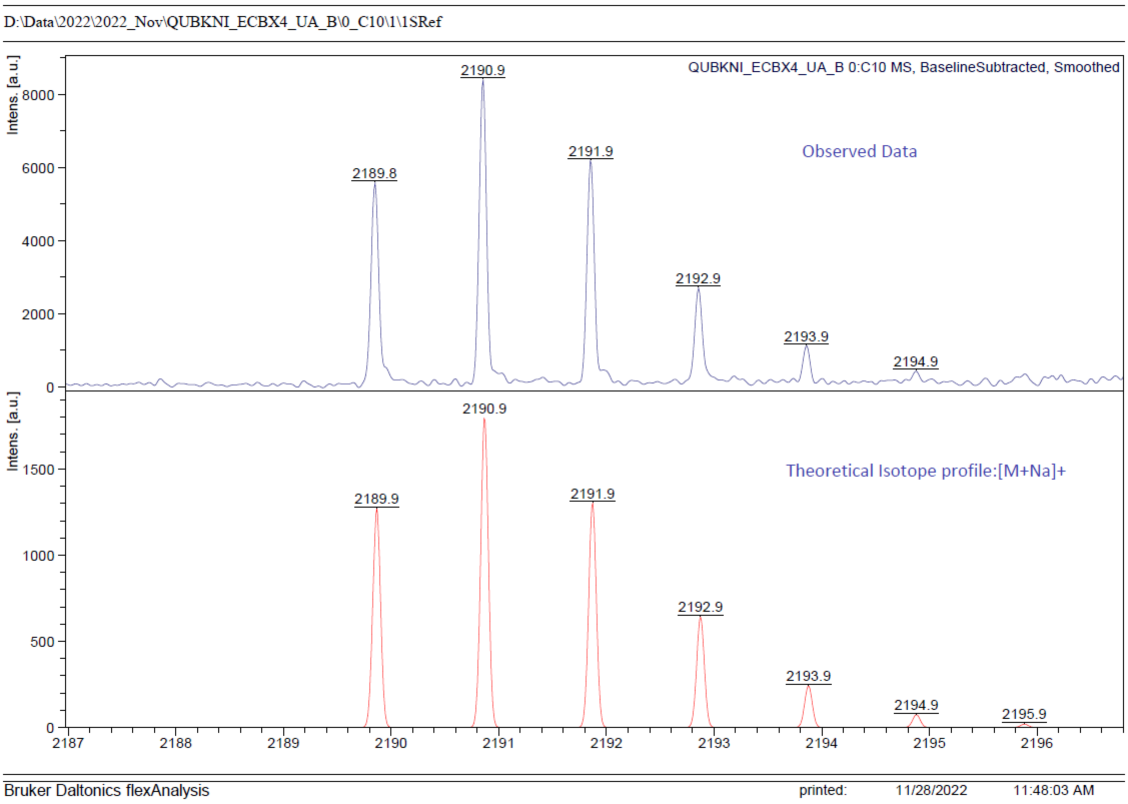


**Figure S5**. Observed (blue) and predicted (red) isotope patterns for **13** detected by MALDI-TOF MS. Top – $[M+H]^+$; bottom – $[M+Na]^+$.

# 2 Supplementary Discussion

## 2.1 Solution-phase Conformational Analysis by NMR

### 2.1.1 General Comments

All NOESY and ROESY data were acquired on a Bruker 600 MHz spectrometer with a mixing time ($t_{mix}$) of 200 ms. Unless otherwise stated, spectra were acquired in $CDCl_3$ at room temperature. Full NOESY/ROESY spectra at two levels of zoom are given, followed by a zoomed view of the cross-peak region of greatest interest. Integrations were carried out manually. Where coloured bands are placed over spectra and with alternating orange/blue colouration, this is done to aid visualization and the colours are arbitrary (unless otherwise stated). Since many of the signals examined are very weak, bands of noise are frequently present at similar levels of intensity.

### 2.1.2 Quantification of ***anti***:***syn*** ratios

To help elucidate the solution-phase conformational behaviour of the spiro-foldamers, nuclear Overhauser effect (nOe) correlations from rotating frame nuclear Overhauser effect (ROESY) spectroscopy were examined using the intensities of the cross-peak areas.

We hypothesised that if a dipole-opposed conformation (NC ∠ NC dihedral angle θ = 180°) is adopted these nOes would be absent (Figure S6). Conversely, in the dipole-aligned conformation (θ = 0°), or if the $C_{pyrazine}$-$N_{lactam}$ bond were freely rotating, a stronger nOe would be expected.

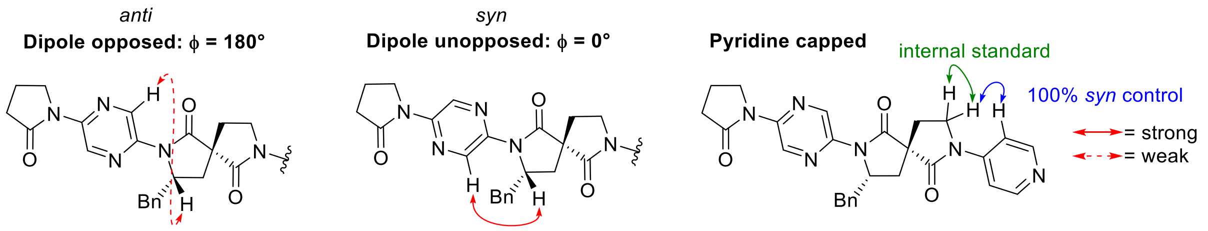


**Figure S6**. nOes expected in the *anti* and *syn* conformations, and an internal standard for quantification.

To obtain a representative value of an nOe interaction for an ortho proton that lies in dipole aligned conformation, both the monomer and dimer were capped with 4-bromopyridine, where the nOe between the pyridine *meta*-hydrogen and the adjacent methylene serves as an internal control, the intensity of which is assumed to equal the nOe observed when one of the bonds of interest adopts a fully *syn* conformation.

To allow for comparison with the non-capped foldamers (i.e. excluding 4-pyridyl), the value of all interactions were normalised relative to a geminal lactam methylene cross-peak internal

standard since the distance between these geminal hydrogens is fixed across all compound. For all compounds the geminal-standard protons (N(CO)C**H**$_2$) were set as the lactam ring furthest away from the 2-pyrrolidinone capped terminus. To give a representative value of bias towards the dipole opposed conformation, the normalised intensity of $H_{Pyrazine}$-$H_{Lactam}$ (dipole opposed) and the normalised intensity of the 4 -pyridyl internal-standard $H_{Pyridine}$-$H_{Lactam}$ (dipole aligned) is represented as a ratio, with a larger value representing a stronger bias towards the dipole opposed conformation:

$$anti{:}syn \text{ ratio} = \left(\frac{H_{Pyridine} \leftrightarrow H_{Lactam} - H_{Pyrazine} \leftrightarrow H_{lactam}}{H_{Pyridine} \leftrightarrow H_{Lactam}}\right) * 100$$

The values obtained are a first approximation, based on the assumptions that (i) each bond examined is only ever in the *syn* or *anti* conformation (since this allows conjugation with the adjacent π-system); (ii) that in the dipole-opposed (*anti*) conformation the cross-peak of interest would have negligible intensity; and (iii) that in the dipole-aligned (*syn*) conformation the intensity would equal that of the internal pyridine control. Assumption (i) is made on the basis of the crystal structure data obtained herein and in previous studies from our group,[8] where imidazolidin-2-ones and lactams in every case adopt a conformation where the carbonyl lies coplanar with the adjacent aromatic heterocycle, and on computational work conducted by Thompson, Hamilton *et. al*, showing the Boltzmann-weighted distribution of conformers in a related system is significantly weighted towards the planar arrangement.[13] Assumption (ii) is made on the basis of the weakness of the nOes observed here. However, if this assumption were incorrect and there is, in fact, an observable nOe in the *anti* conformation then our analysis would under-state the *anti*:*syn* ratio. We assert assumption (iii) on the basis of the structural similarity between the relative positions of the hydrogens in the internal control *versus* those under investigation.

### 2.1.3 Capped monomer 14

**ROESY, $CDCl_3$, 600 MHz, $t_{mix}$ = 0.2 s**

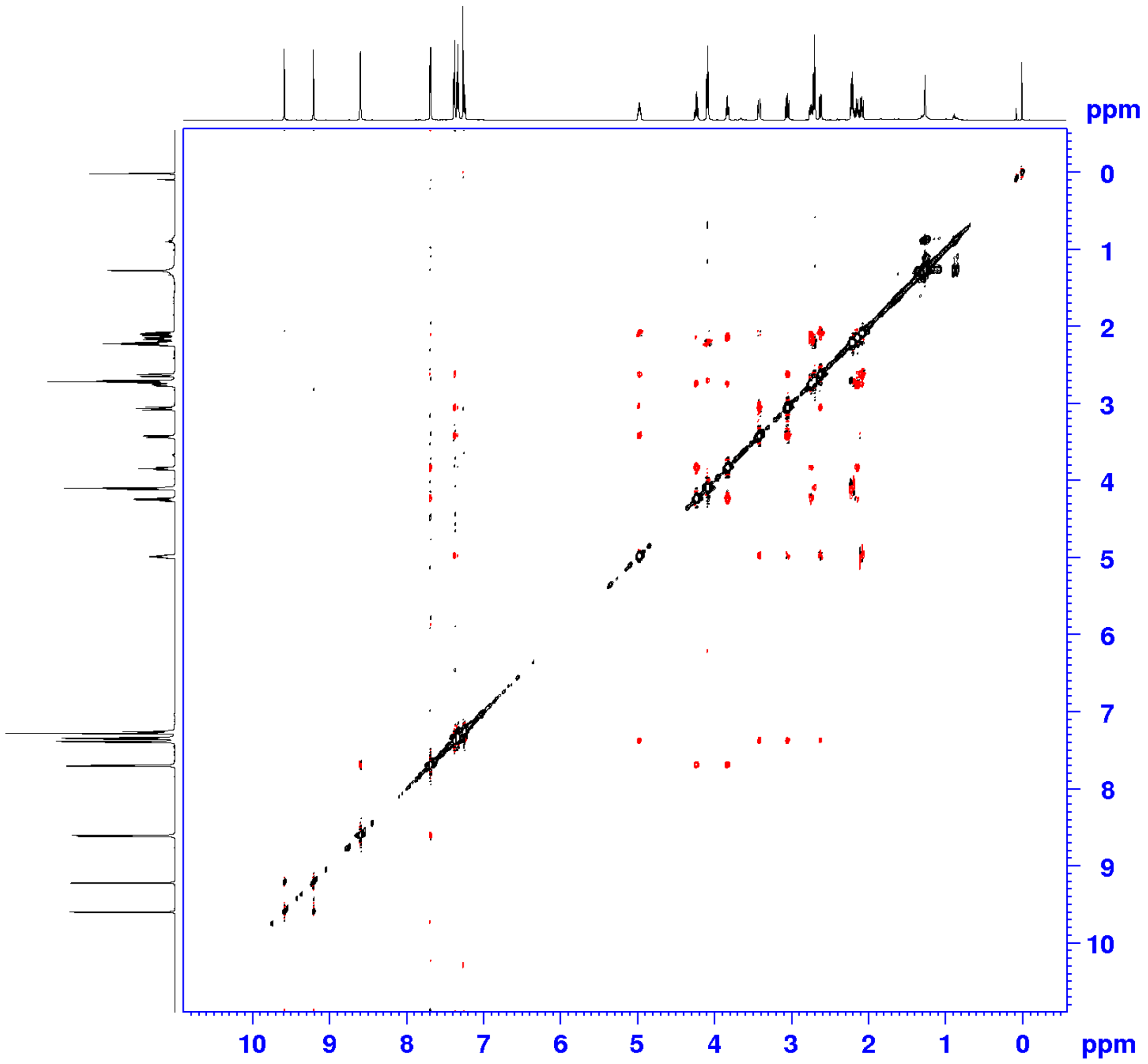

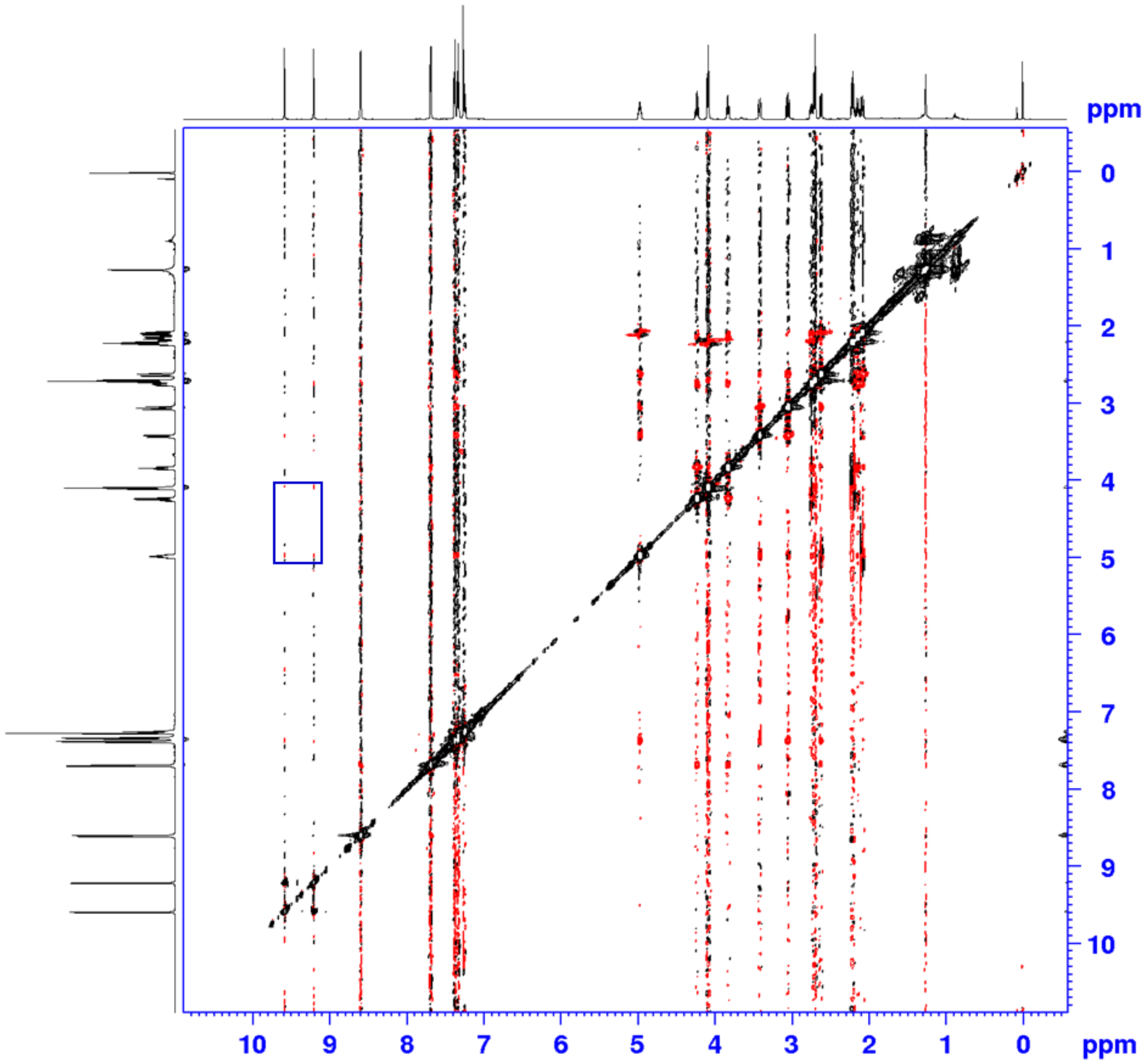
ppm
0
1
2
3
4
5
6
7
8
9
10
10
9
8
7
6
5
4
3
2
1
0
ppm

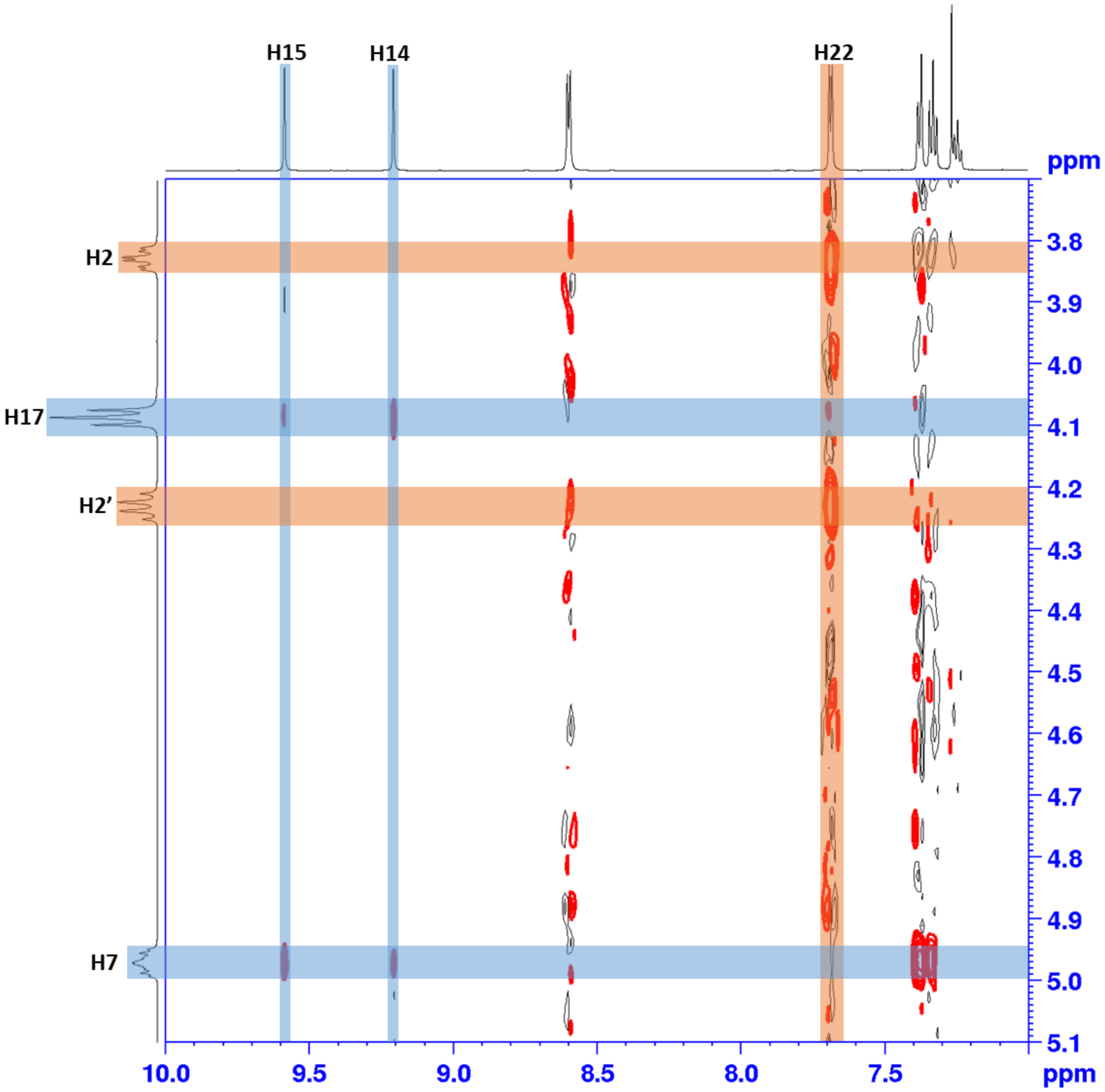


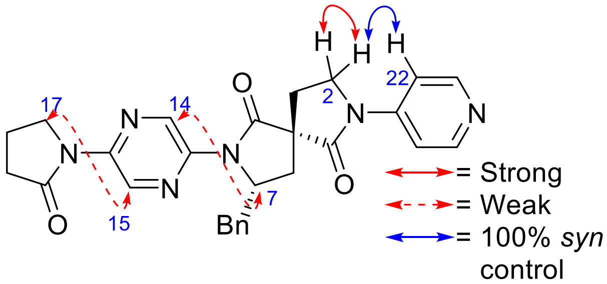


**Table S1. Selected nOe cross-peak intensities of capped monomer 14 in $CDCl_3$**

| Peak | Normalized intensity | *Anti*:*Syn* |
|---|---|---|
| H2’-H2 | 1.00 | - |
| H2[b] -H22 | 0.19 | - |
| H17[a]-H15 | 0.0019 | 99:1 |
| H7-H14 | 0.0025 | 99:1 |

[a] Isochronous peaks, value taken as an average of the two protons
[b] Taken as an average of the two protons

Using the strong nOe interaction between H2↔H22 and standardizing this against the fixed constant of the lactam methylene geminal protons H2'↔H2 for integration of the peaks, the nOe intensities of H17↔H15 and H7↔H14 are significantly weaker than the nOe of H2↔H22 (the internal standard corresponding to a 100% syn conformation), indicating these protons are being orientated away for lactam ring as a result of dipole repulsion from the pyrazine's nitrogen and lactam's carbonyl moieties, C13-*N*. Both H17↔H15 and H7↔H14 gave the same *Anti:Syn* ratio of 99:1, therefore indicating there is a strong conformational bias/dipole-repulsion experienced by both units, and is in agreement with the crystal structure of **7**.

Looking at the intensities of the nOe cross-peaks for both the deprotected (**8**) and tosyl-protected (**7**) monomers standardized against lactam methylene geminal protons, the representative interactions (H12↔H19 & H22↔H20 for **7** and H17↔H15 & H7↔H14 for **8**) are of similar intensity to those in control compound **14**, indicating that the strong dipolar repulsion and conformational bias is consistent and is being exhibited in these structures.

### 2.1.4 Ts-protected monomer 7

**ROESY, $CDCl_3$, 600 MHz, $t_{mix}$ = 0.2 s**

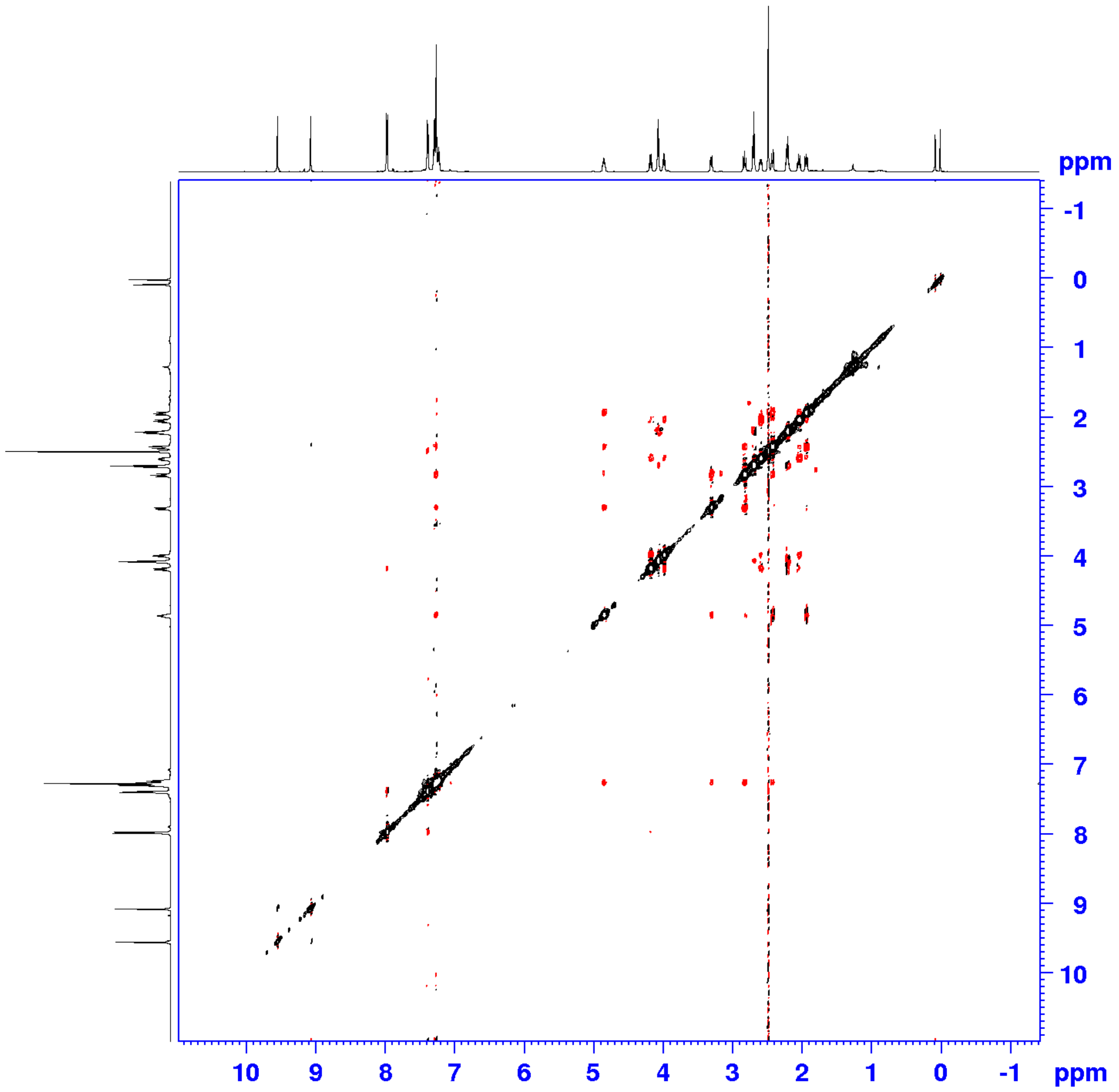

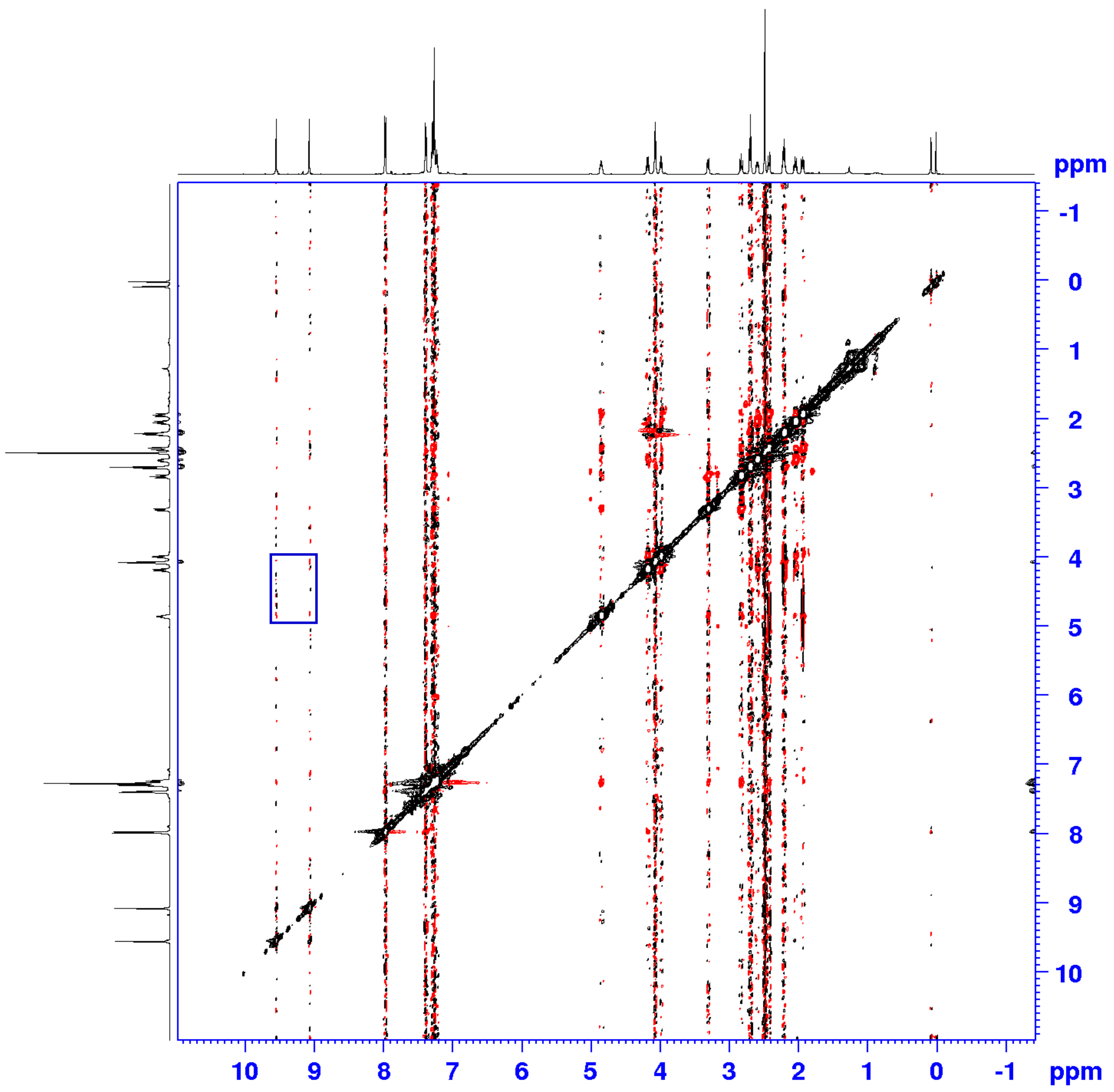
ppm
-1
0
1
2
3
4
5
6
7
8
9
10
10
9
8
7
6
5
4
3
2
1
0
-1
ppm

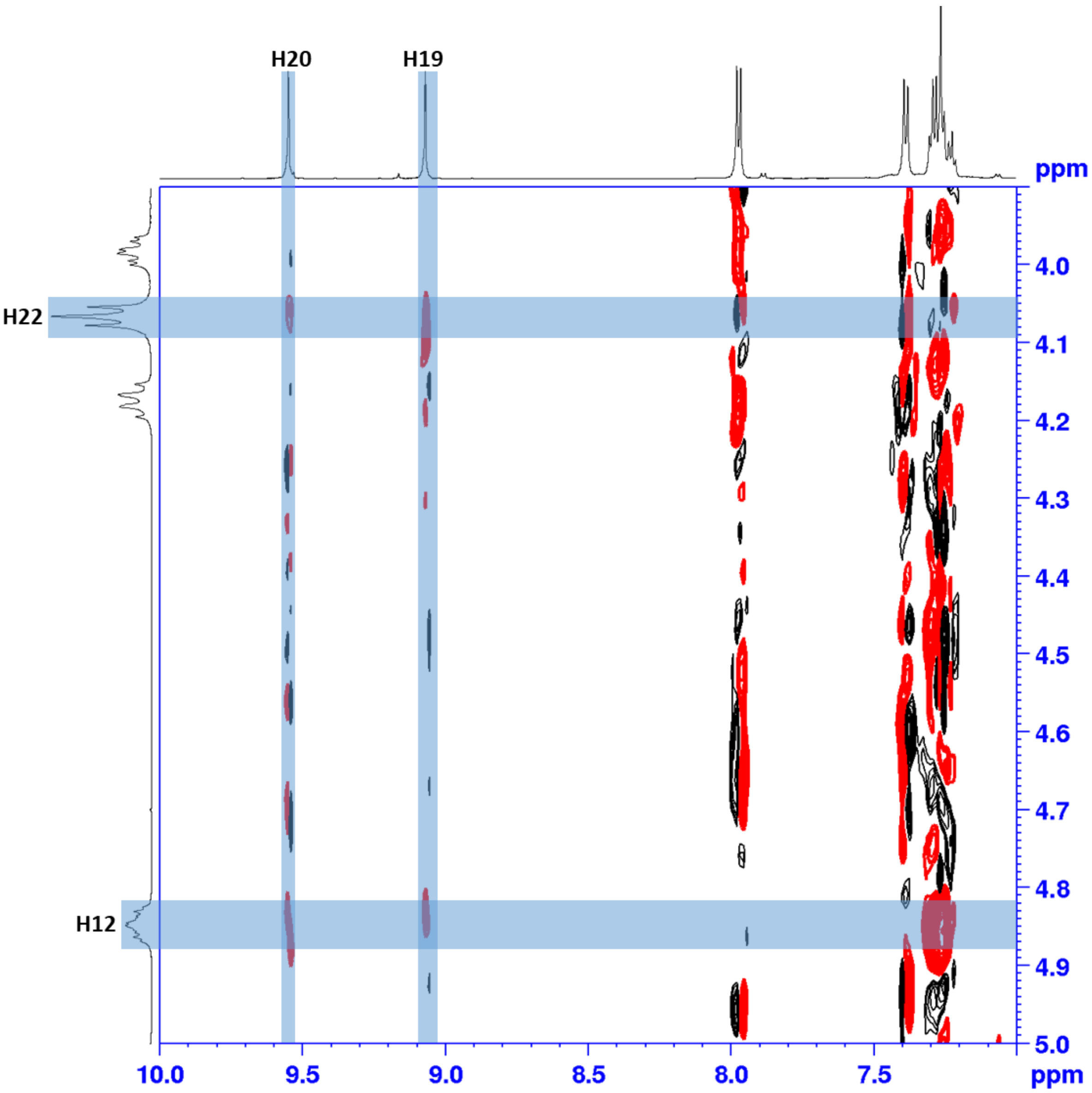


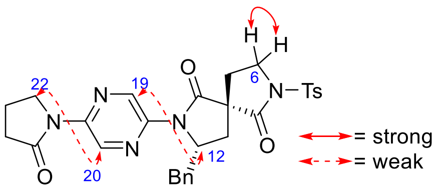


**Table S2. Selected nOe cross-peak intensities of Ts-protected monomer 7 in $CDCl_3$**

| Peak | Normalized intensity |
| --- | --- |
| H6'-H6 | 1.00 |
| H22[a]-H20 | 0.0016 |
| H12-H19 | 0.0031 |

[a] Isochronous peaks, value taken as an average of the two protons

### 2.1.5 Deprotected monomer 8

**ROESY, $CDCl_3$, 600 MHz, $t_{mix}$ = 0.2 s**

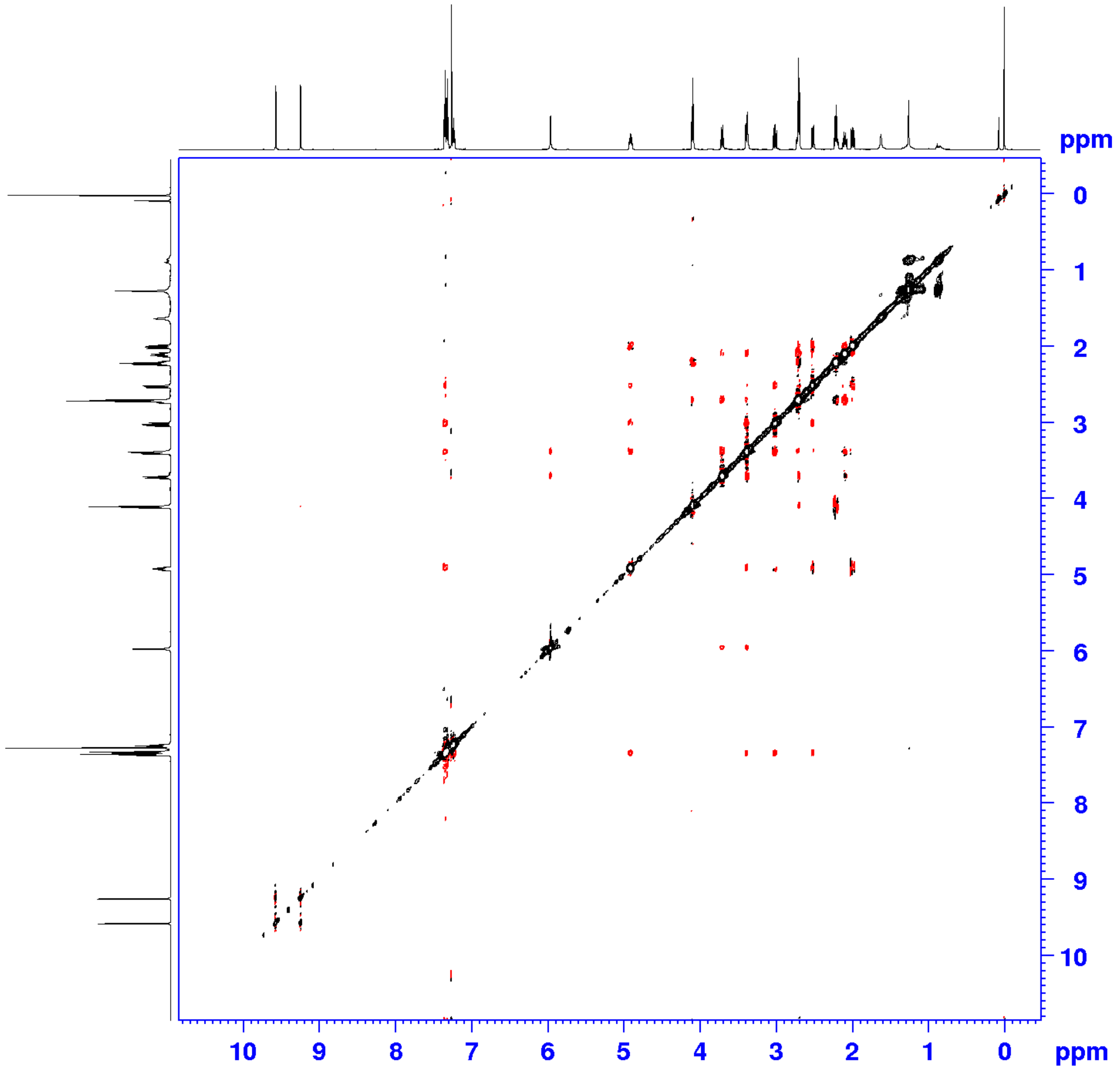

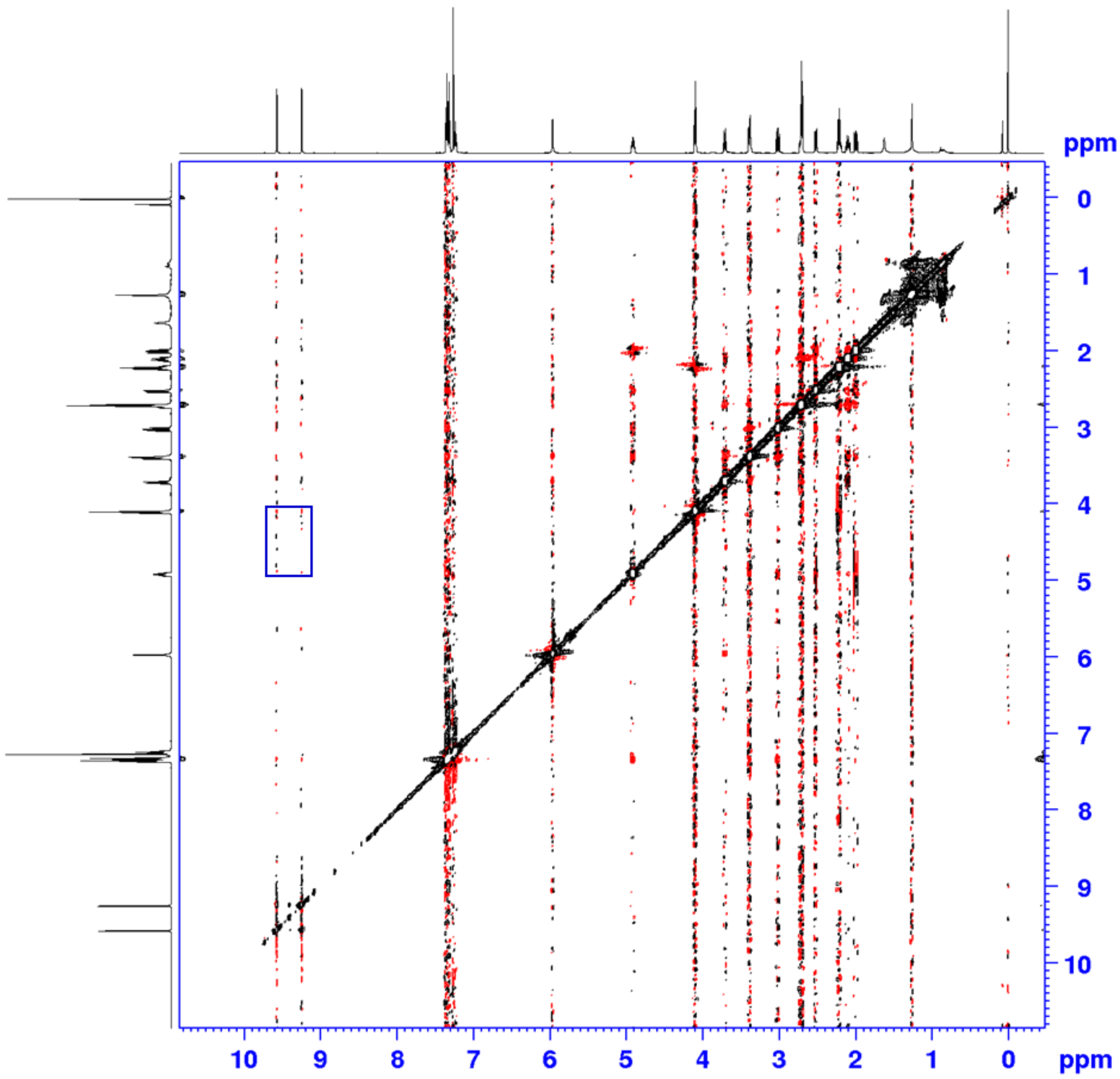
ppm
0
1
2
3
4
5
6
7
8
9
10
10
9
8
7
6
5
4
3
2
1
0
ppm

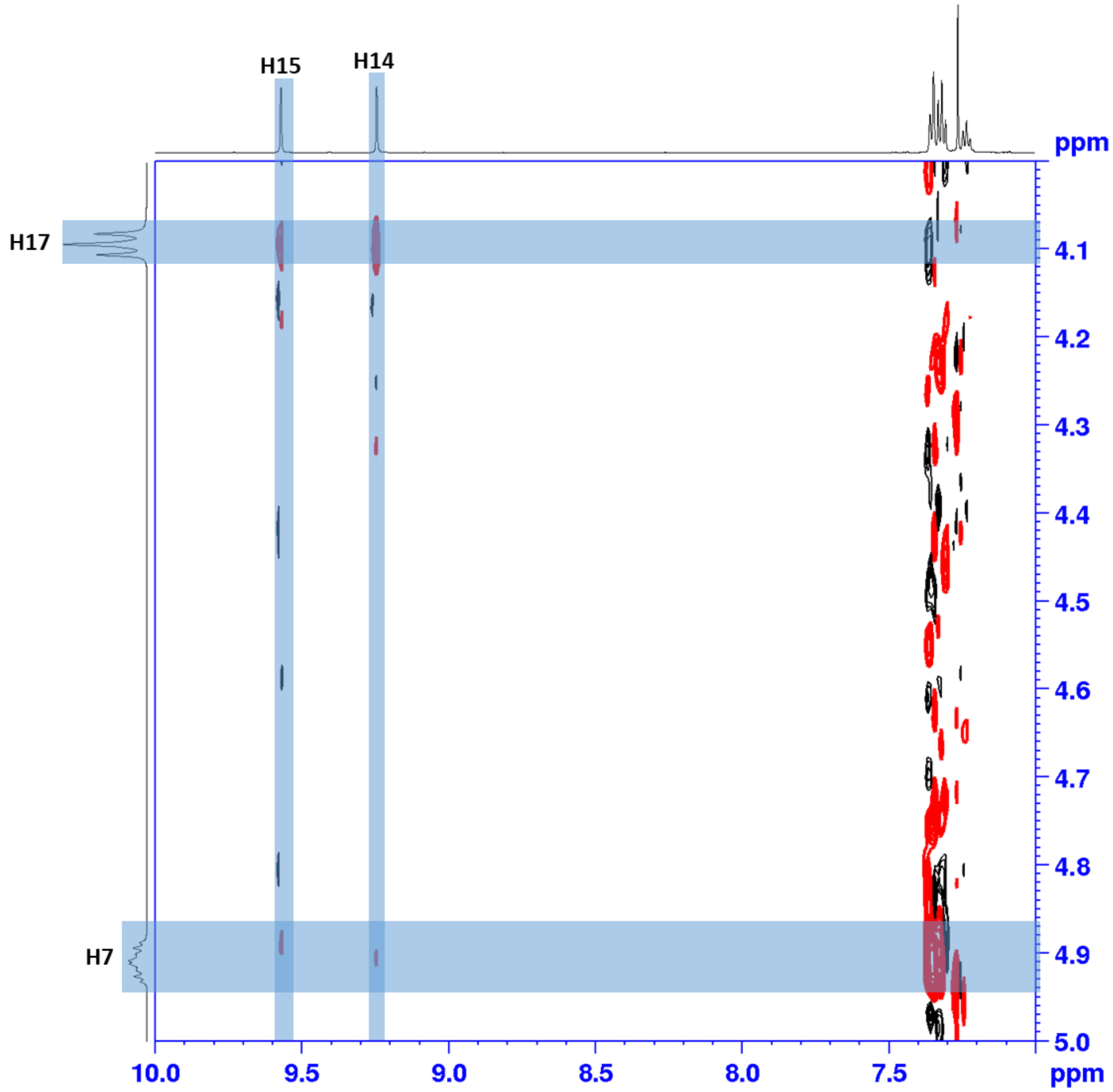


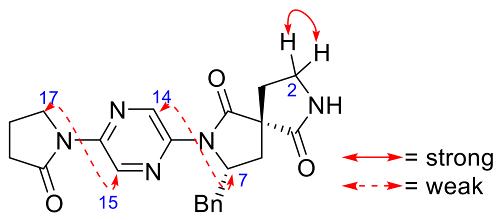


**Table S3. Selected nOe cross-peak intensities of deprotected monomer 8 in $CDCl_3$**

| Peak | Normalized intensity |
|---|---|
| H2'-H2 | 1.00 |
| H17[a]-H15 | 0.0023 |
| H7-H14 | 0.0037 |

[a] Isochronous peaks, value taken as an average of the two protons

### 2.1.6 Capped dimer 15

**ROESY, $CDCl_3$, 600 MHz, $t_{mix}$ = 0.2 s**

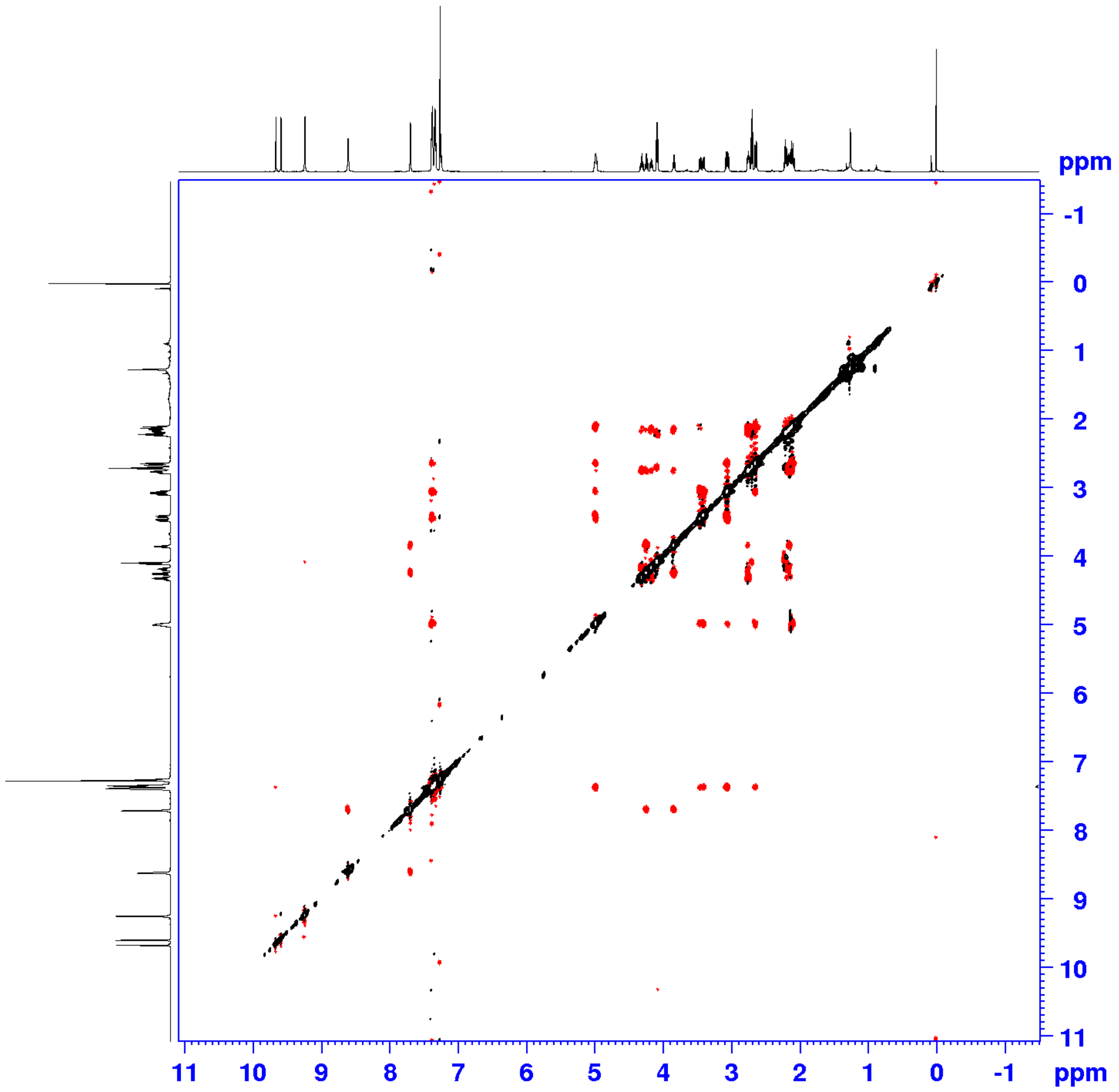

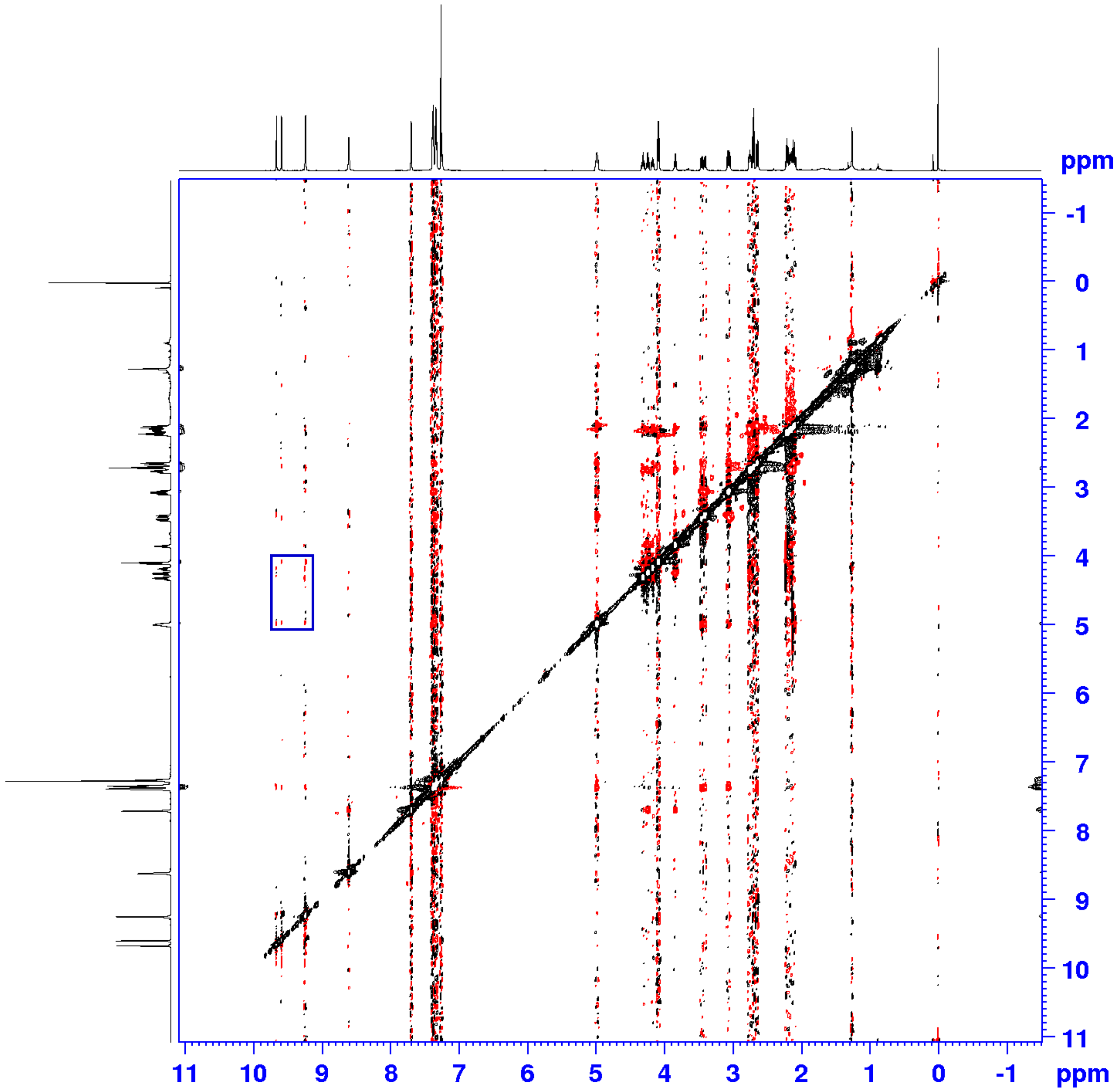
ppm
-1
0
1
2
3
4
5
6
7
8
9
10
11
11
10
9
8
7
6
5
4
3
2
1
0
-1
ppm

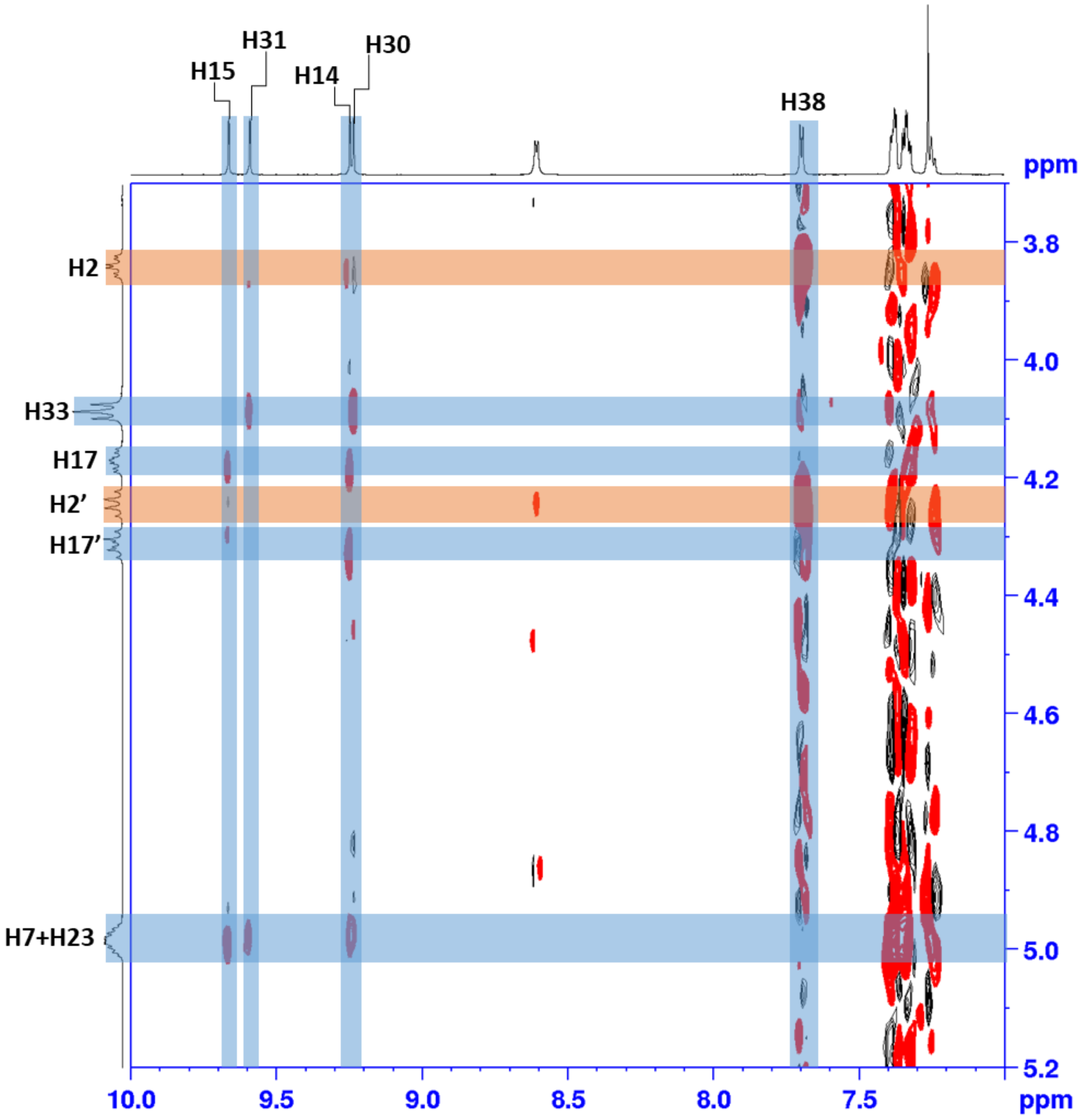

H15
H31
H14
H30
H38
ppm
H2
H33
H17
H2'
H17'
H7+H23
3.8
4.0
4.2
4.4
4.6
4.8
5.0
5.2
10.0
9.5
9.0
8.5
8.0
7.5
ppm

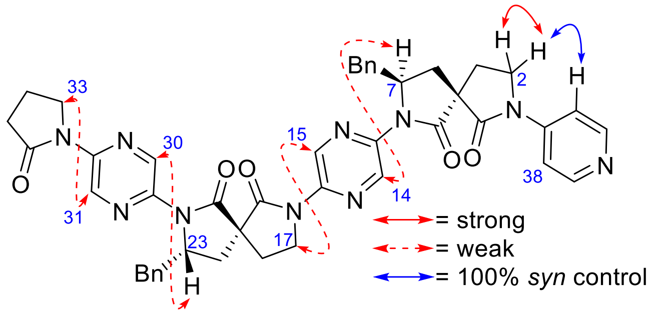


**Table S4. Selected nOe cross-peak intensities of capped dimer 15 in $CDCl_3$**

| Peak | Normalized intensity | Anti:Syn |
|---|---|---|
| H2'-H2 | 1.00 | - |
| H2[b]-H38 | 0.20 | - |
| H33[a]-H31 | 0.0012 | 99:1 |
| H17'-H15 | 0.0016 | - |
| H17-H15 | 0.0025 | - |
| H17-H15 (average) | 0.0021 | 99:1 |
| [H7+H23]–[H14+H30] | 0.0038 | [c] |

[a] Isochronous peaks, value taken as an average of the two protons [b] Taken as an average of the two protons, [c] The values of H23↔H30 & H7↔H14 cannot be calculated due to overlap between the cross-peaks

Using the strong nOe interaction between H2↔H38 and standardizing this against the fixed constant of the lactam methylene geminal protons H2'-H2 for integration of peaks. We can once again see that all other interactions (H33↔H31 & H17'/17↔15) compared with the pyridine's ortho proton (H2↔H38), are still significantly weaker, with all *anti*:*syn* ratios being 99:1. Although, due to overlap of the cross-peaks associated with H23↔H30 & H7↔H14 the exact normalized intensity cannot be calculated, assuming that each proton is responsible for half the area, then this would give a normalized intensity of 0.0019. These values remain in a similar range to those of monomer **14**, showing a consistent level of dipolar control between the two structures.

Looking at the intensities for the nOe cross-peaks for both the deprotected (**10**) and tosyl (**9**) protected dimers standardized against lactam methylene geminal protons, the representative interactions (**see Table S5 and S6**) values for the normalized intensities remain very similar throughout, indicating that the strong dipolar repulsion and conformational bias is consistent and is still being exhibited in these structures.

### 2.1.7 Ts-protected dimer 9

**ROESY, $CDCl_3$, 600 MHz, $t_{mix}$ = 0.2 s**

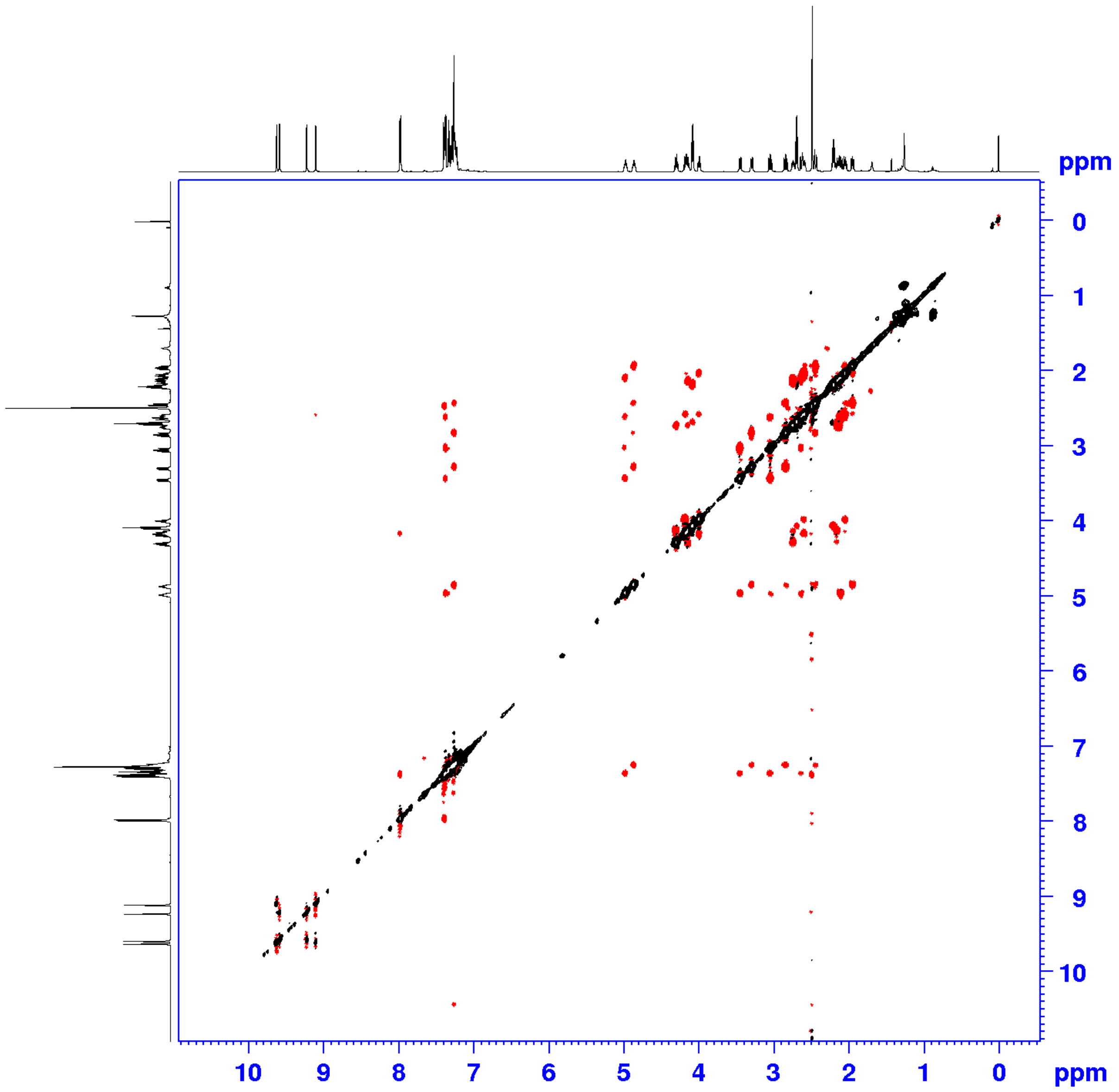

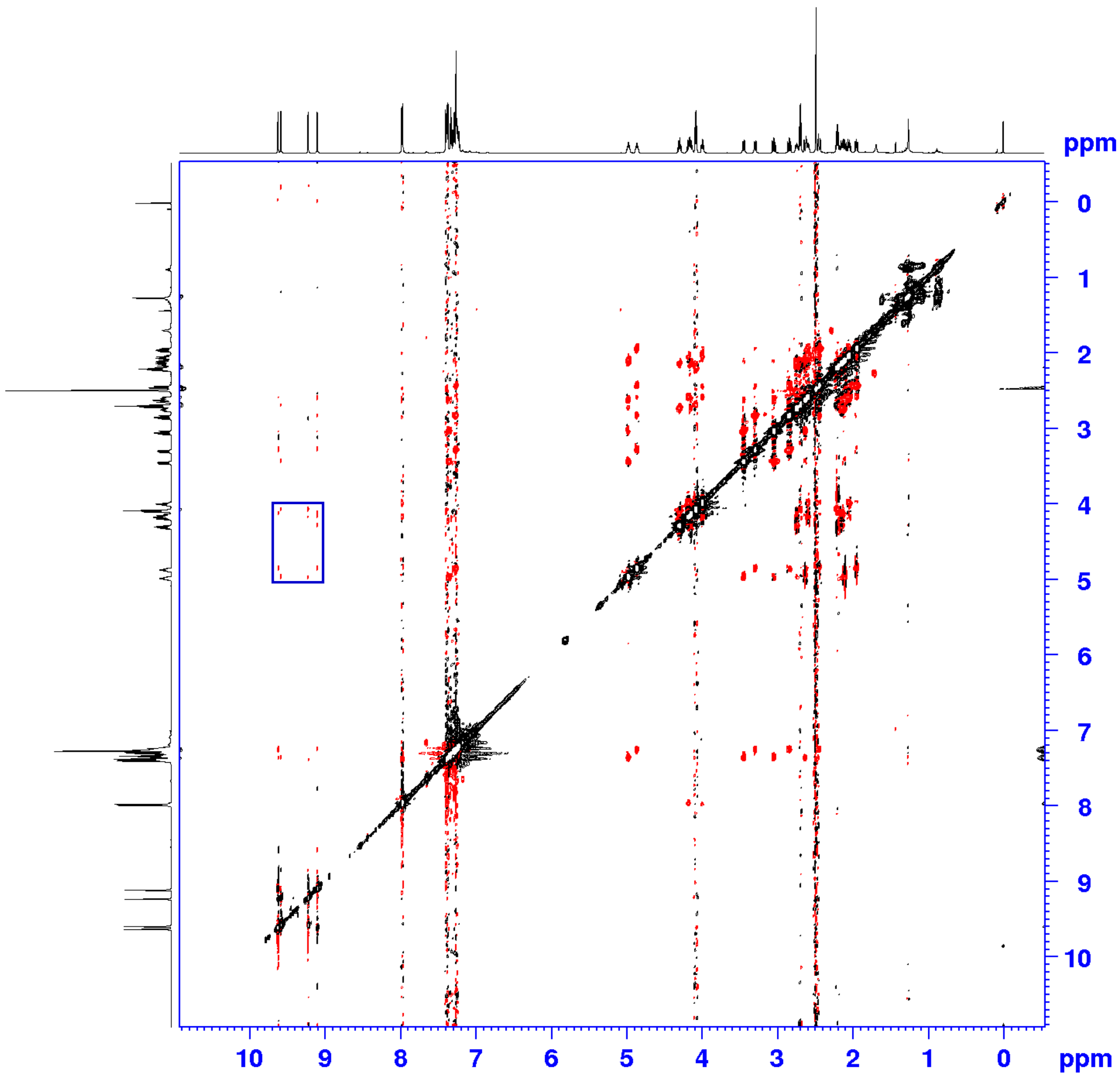
ppm
0
1
2
3
4
5
6
7
8
9
10
10
9
8
7
6
5
4
3
2
1
0
ppm

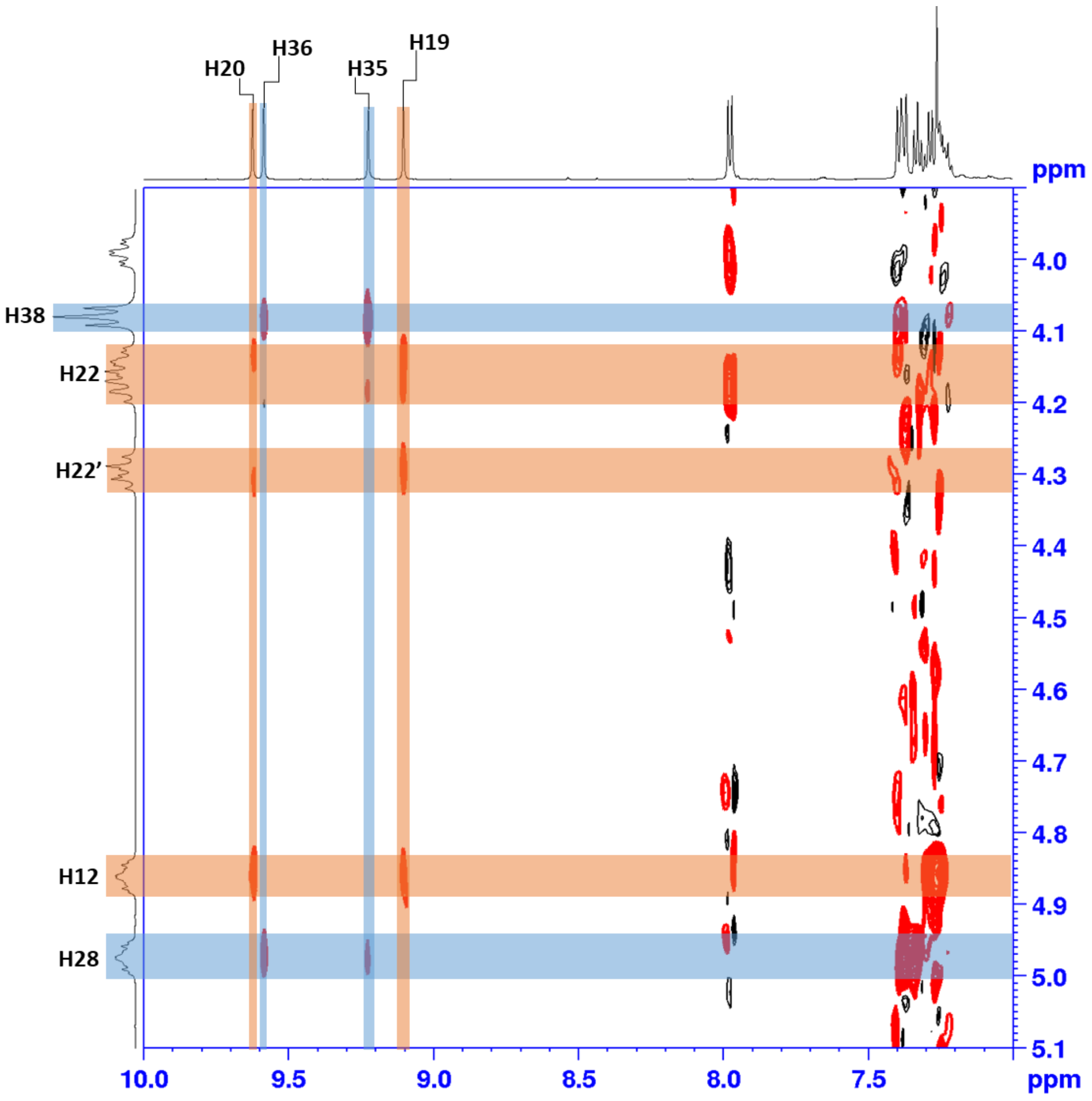


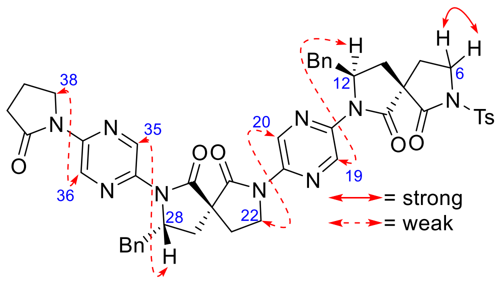


**Table S5. Selected nOe cross-peak intensities of Ts-protected dimer 9 in $CDCl_3$**

| Peak | Normalized intensity |
|---|---|
| H6'-H6 | 1.00 |
| H38[a]-H36 | 0.0024 |
| H28-H35 | 0.0044 |
| H22'-H20 | 0.0026 |
| H22-H20 | 0.0024 |
| H12-H19 | 0.0056 |

[a] Isochronous peaks, value taken as an average of the two protons

### 2.1.8 Deprotected dimer 10

**ROESY, $CDCl_3$, 600 MHz, $t_{mix}$ = 0.2 s**

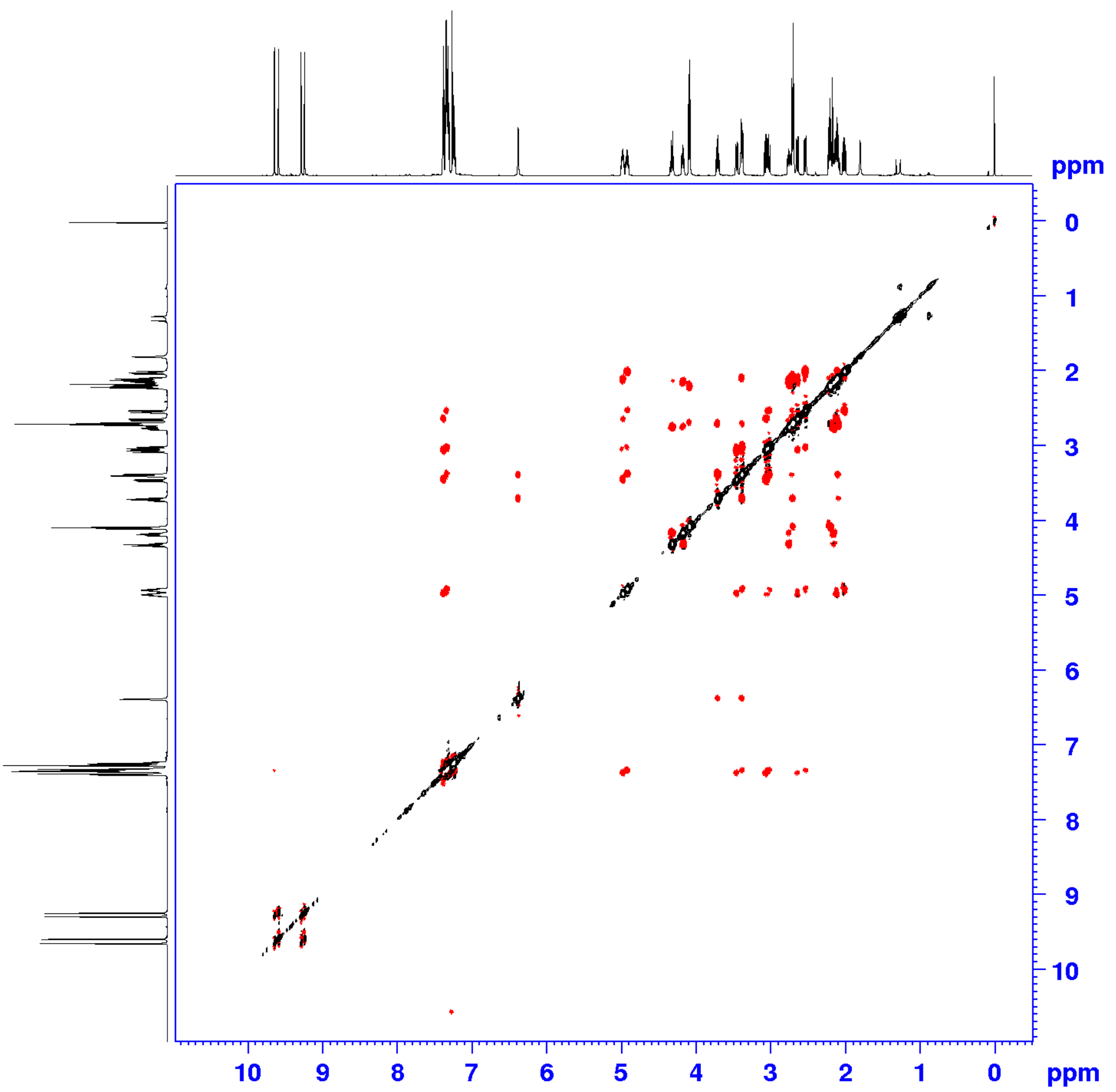

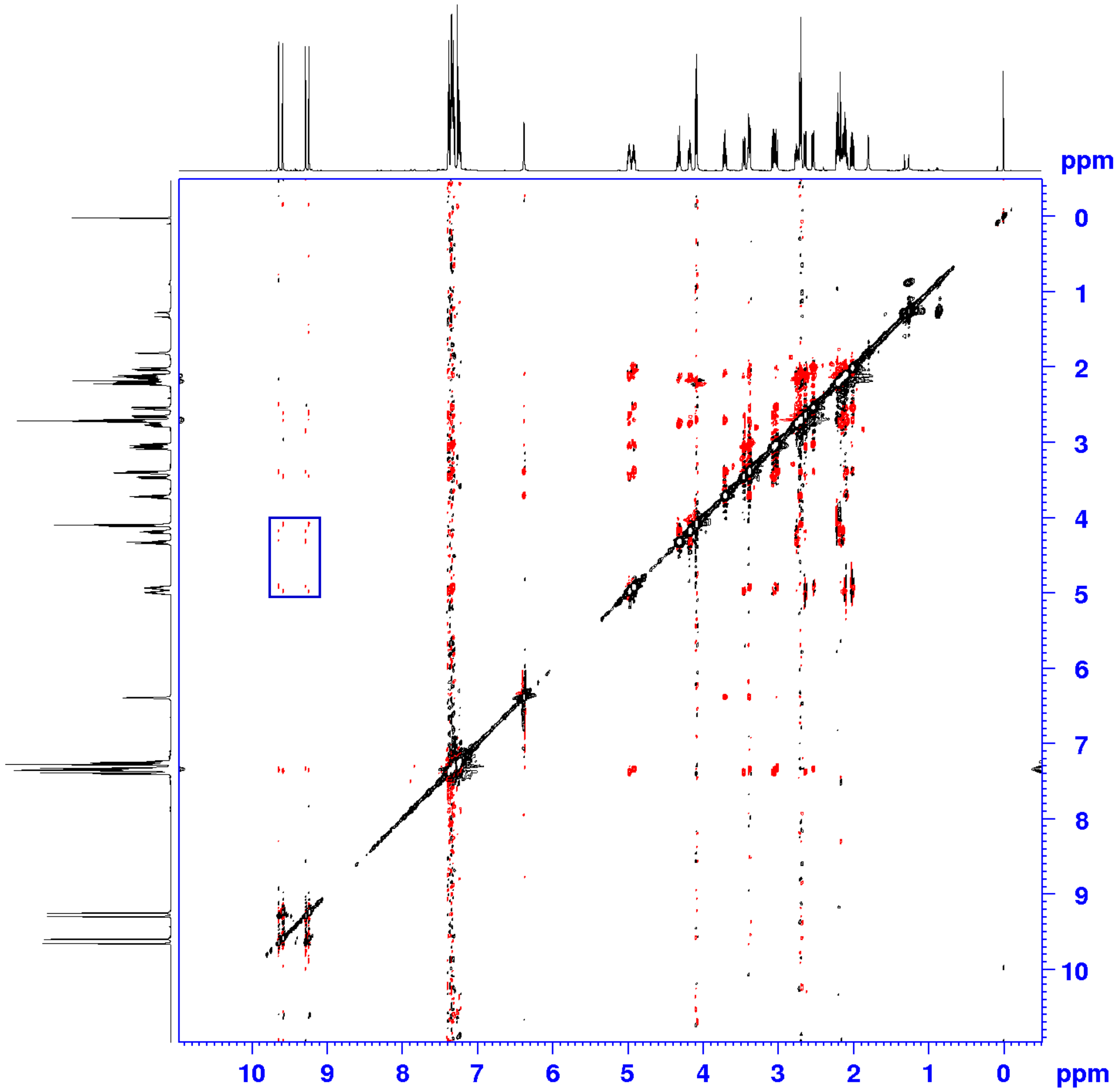
ppm
0
1
2
3
4
5
6
7
8
9
10
10
9
8
7
6
5
4
3
2
1
0
ppm

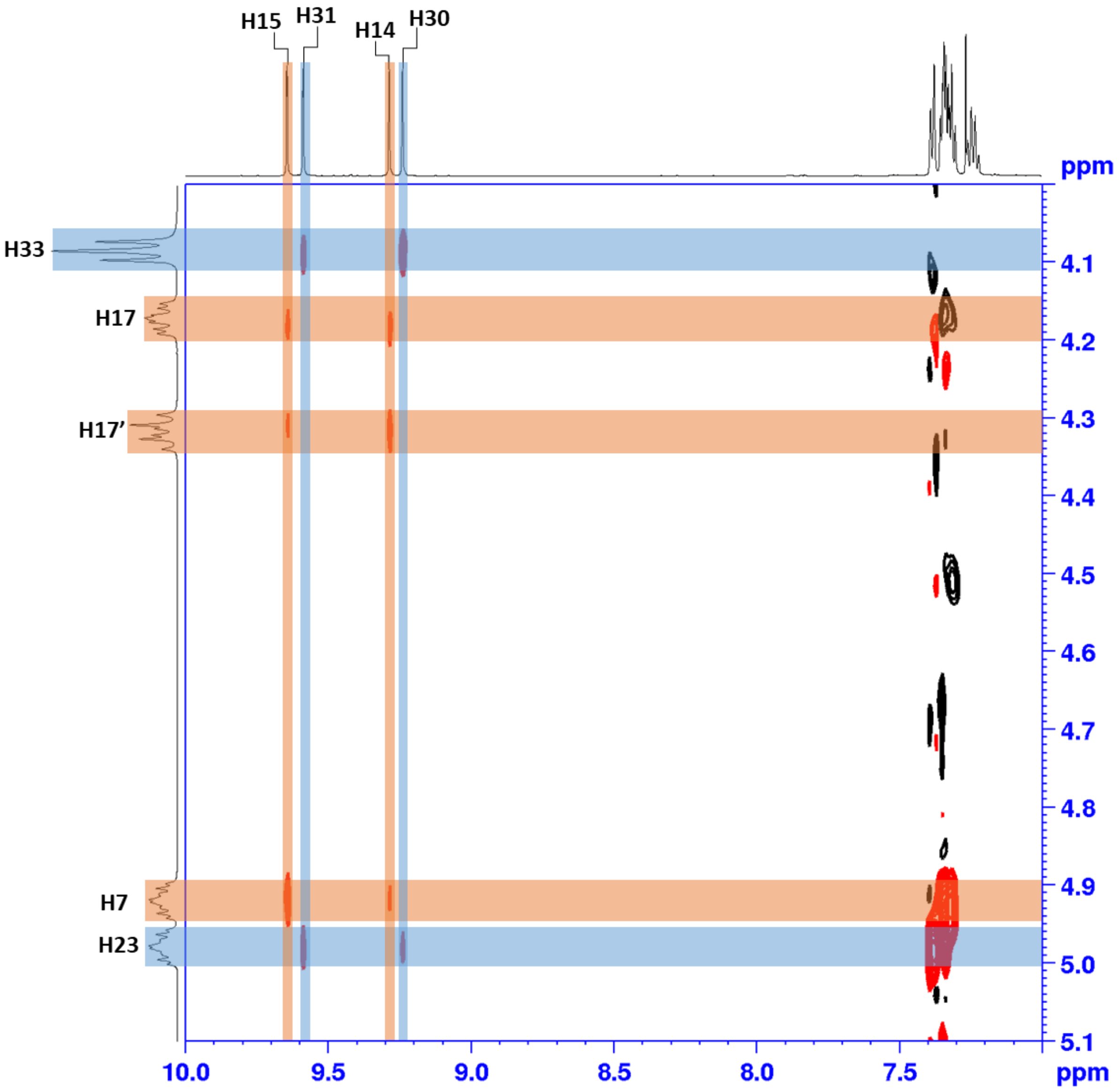


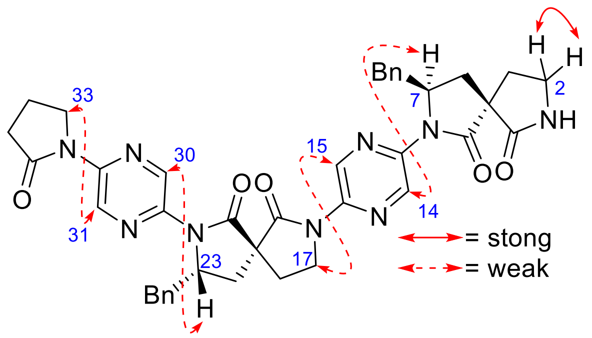


**Table S6. Selected nOe cross-peak intensities of deprotected dimer 10 in $CDCl_3$**

| Peak | Normalized intensity |
|---|---|
| H2'-H2 | 1.00 |
| H33[a]-H31 | 0.0030 |
| H23-H30 | 0.0047 |
| H17'-H15 | 0.0045 |
| H17-H15 | 0.0032 |
| H7-H14 | 0.0042 |

[a] Isochronous peaks, value taken as an average of the two protons

## 2.1.9 *$d_6$*-DMSO and temperature influence on conformational control

In addition to the above studies in $CDCl_3$ (dielectric constant ε 4.8), the conformation of 4-pyridyl capped monomer **14** and dimer **15**, was examined in the much more polar $d_6$-DMSO (ε 46.7), and at elevated temperature to determine the influence on the dipole repulsion-mediated conformational control (Figure S7). In order to determine the effects of $d_6$-DMSO and temperature the *anti:syn* ratio was used since a decrease in the value would indicate a decrease in the biasing effect.

### *2.1.9.1 Capped monomer 14*

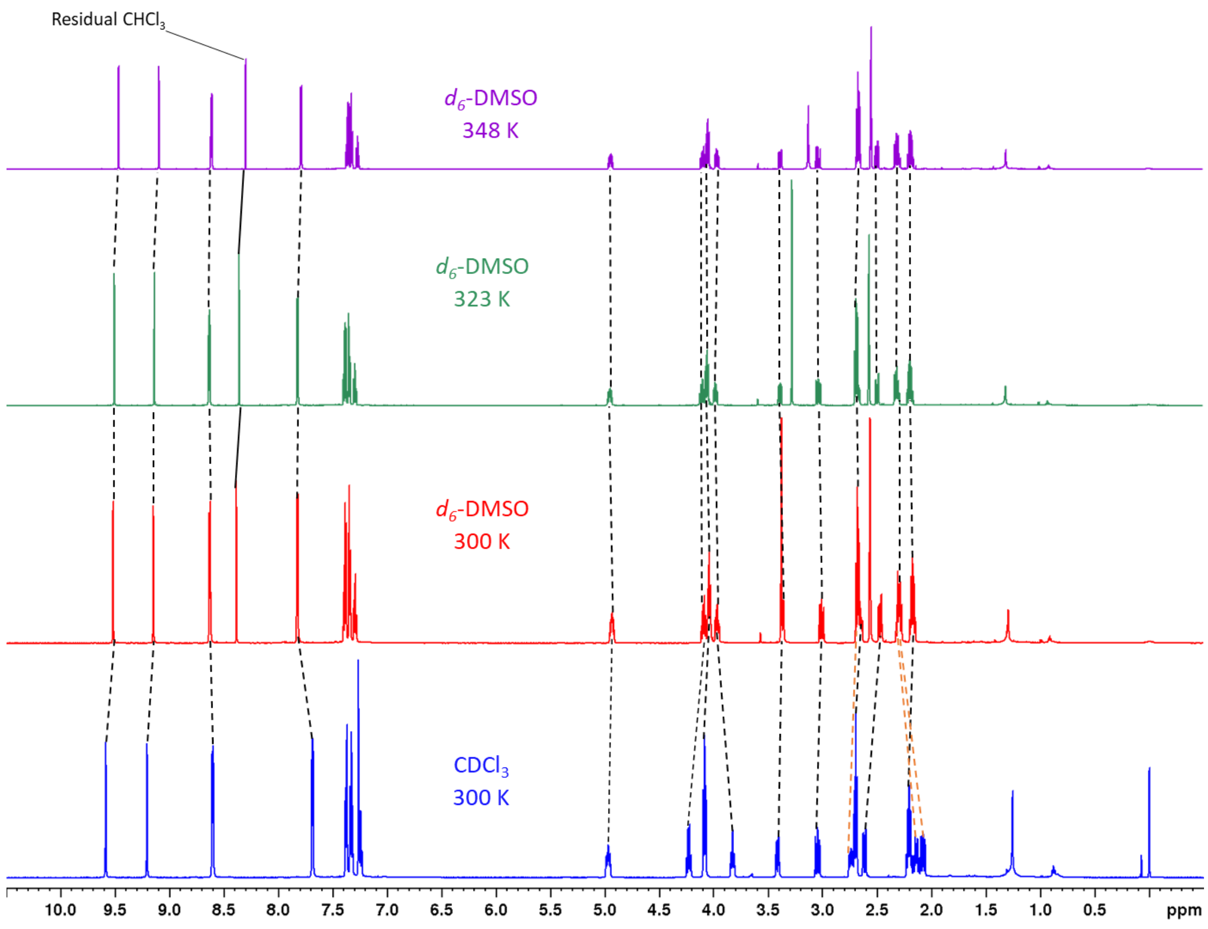


**Figure S7**. Temperature and solvent dependence of $^1$H NMR spectra of capped monomer **14** (600 MHz).

**ROESY, $d_6$-DMSO, 600 MHz, $t_{mix}$ = 0.2 s**

**300 K**

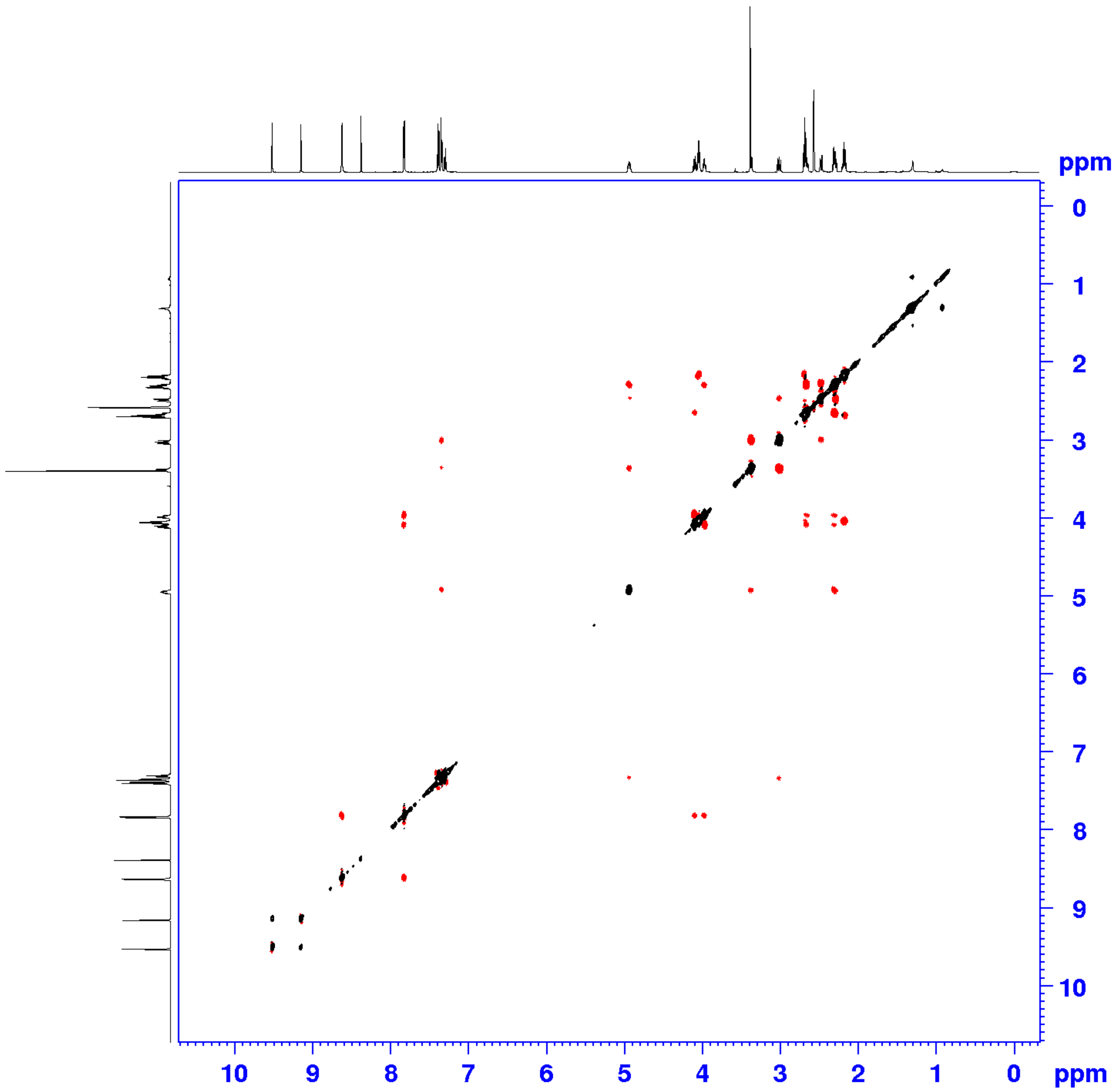

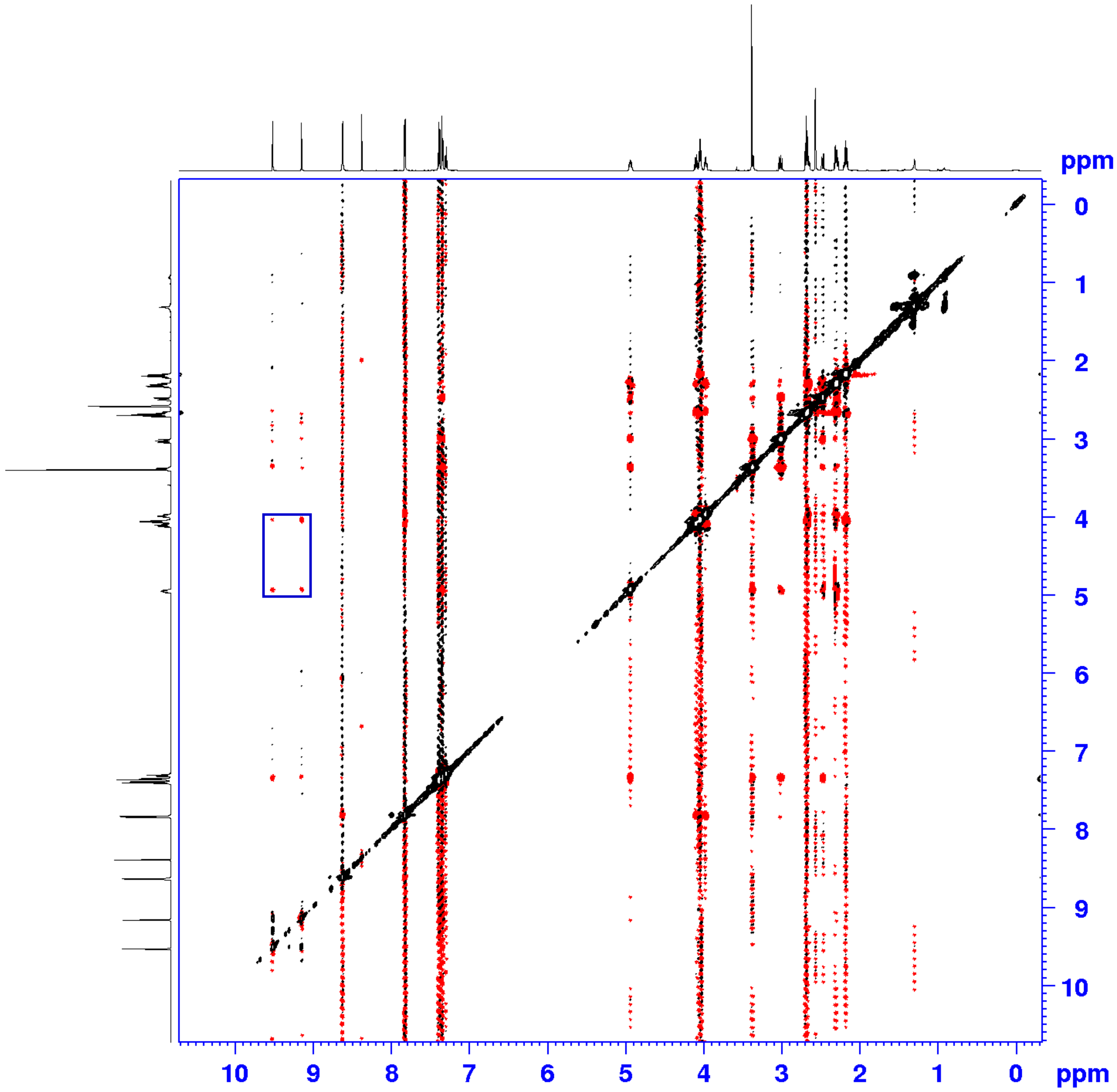
ppm
0
1
2
3
4
5
6
7
8
9
10
10
9
8
7
6
5
4
3
2
1
0
ppm

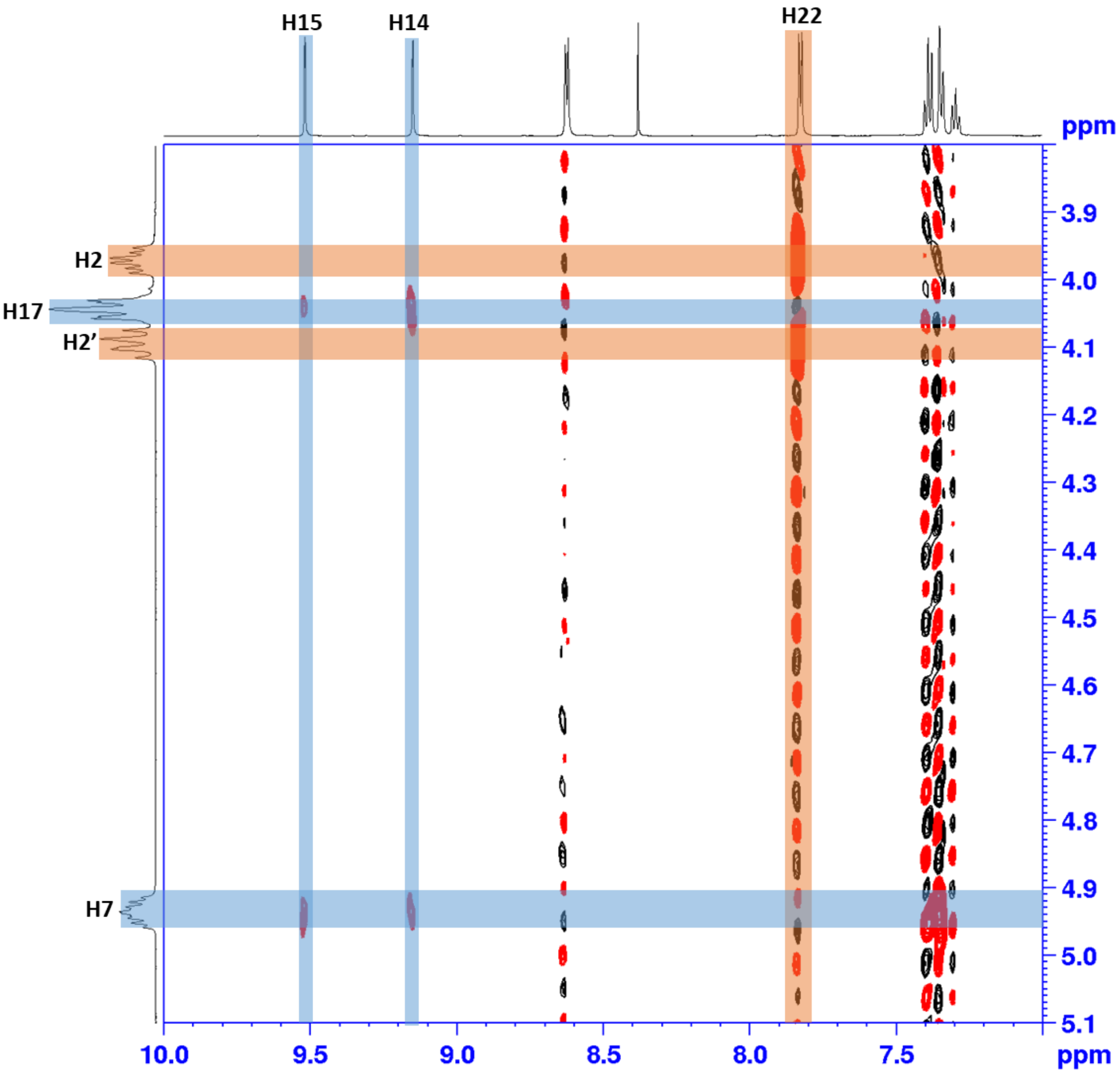


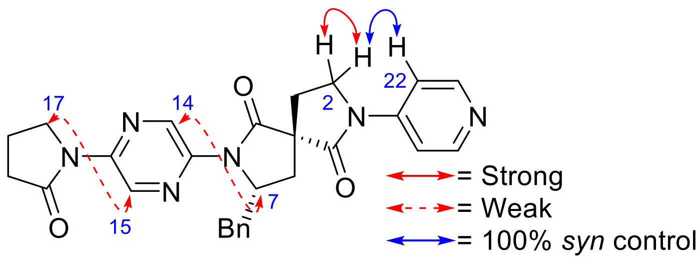


**Table S7.** The effect of solvent polarity and temperature on the dipole repulsion/conformational control for the 4-pyridyl capped monomer **14**.

| Solvent | Temperature (K) | 4-pyridyl standard (H2↔22) | H17↔H15[a] | *Anti*:*Syn* | H7↔H14 | *Anti*:*Syn* |
|---|---|---|---|---|---|---|
| $CDCl_3$ | 300 | 0.19 | 0.0019 | 99:1 | 0.0025 | 99:1 |
| $d_6$-DMSO | 300 | 0.31 | 0.0032 | 99:1 | 0.0071 | 98:2 |
| $d_6$-DMSO | 323 | 0.34 | 0.0037 | 99:1 | 0.0077 | 98:2 |
| $d_6$-DMSO | 348 | 0.32 | 0.0039 | 99:1 | 0.0075 | 98:2 |

[a] isochronous peaks, therefore value taken as an average of the two protons

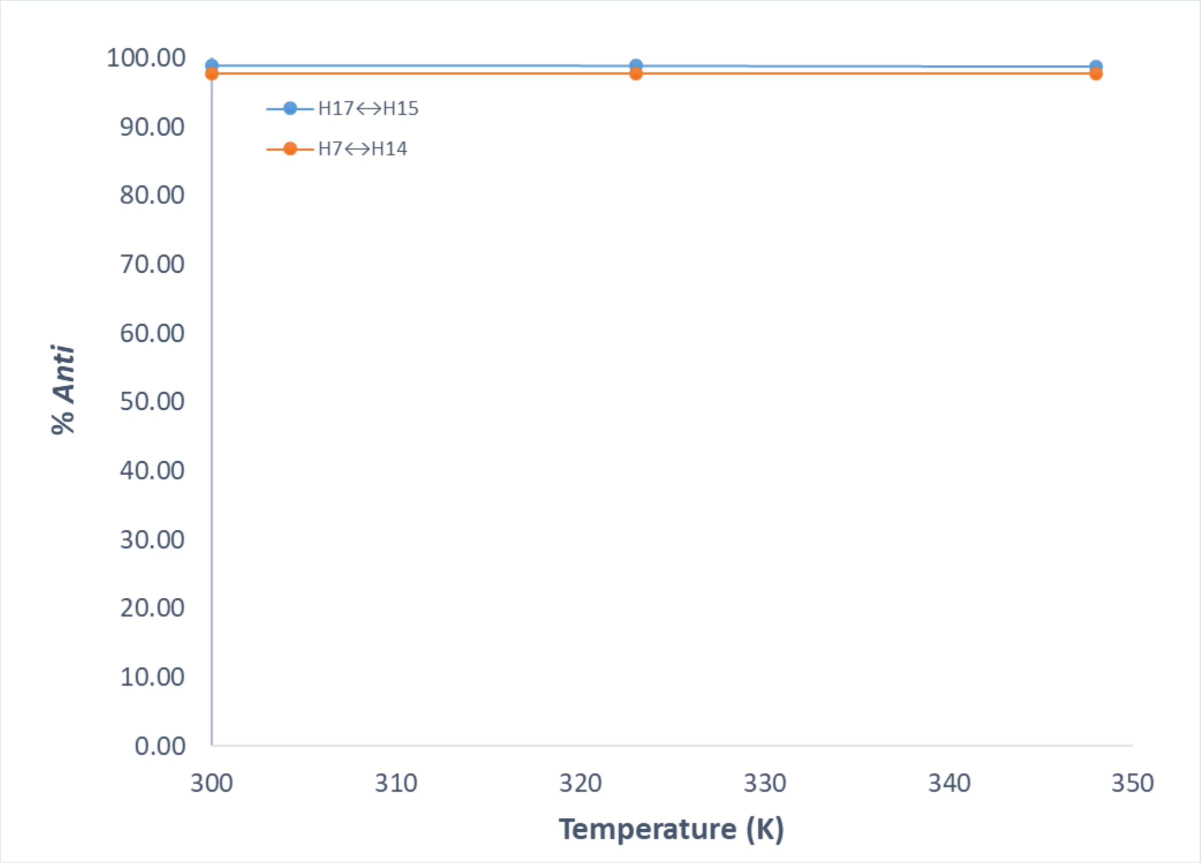


**Figure S8.** The effect of temperature on the dipolar conformational control for capped monomer **14** in $d_6$-DMSO

Firstly, looking at the influence of solvent polarity and comparing the *Anti:Syn* ratio with $CDCl_3$, (**Table S7**) the capped monomer **14** in $d_6$-DMSO still retains its dipole-opposed conformation, with the interactions between H17↔H15 and H7↔H14 being similar in magnitude to those observed in $CDCl_3$. Looking at the ratio of *Anti:Syn,* there was no change for H17↔H15 whilst H7↔H14 experience a minimal reduction of 99:1 → 98:2, which therefore indicates that even in a more polar solvent that has the ability to disrupt the conformation, a strong conformation bias is still retained.

Secondly, looking at the influence of temperature on **14** in $d_6$-DMSO, we can see that as the temperature was raised, the value for the cross-peak area increased very slightly (**Table S7 and Figure S8**), e.g. for H17↔H15, at 300K is 0.0032 whilst at 348 K it is 0.0039). Therefore, the *Anti:Syn* ratio was largely unaffected, indicating that even at the high temperature of 348 K, the conformation bias is still very strong and the dipole opposed conformation is still retained.

### 2.1.9.2 Capped dimer 15

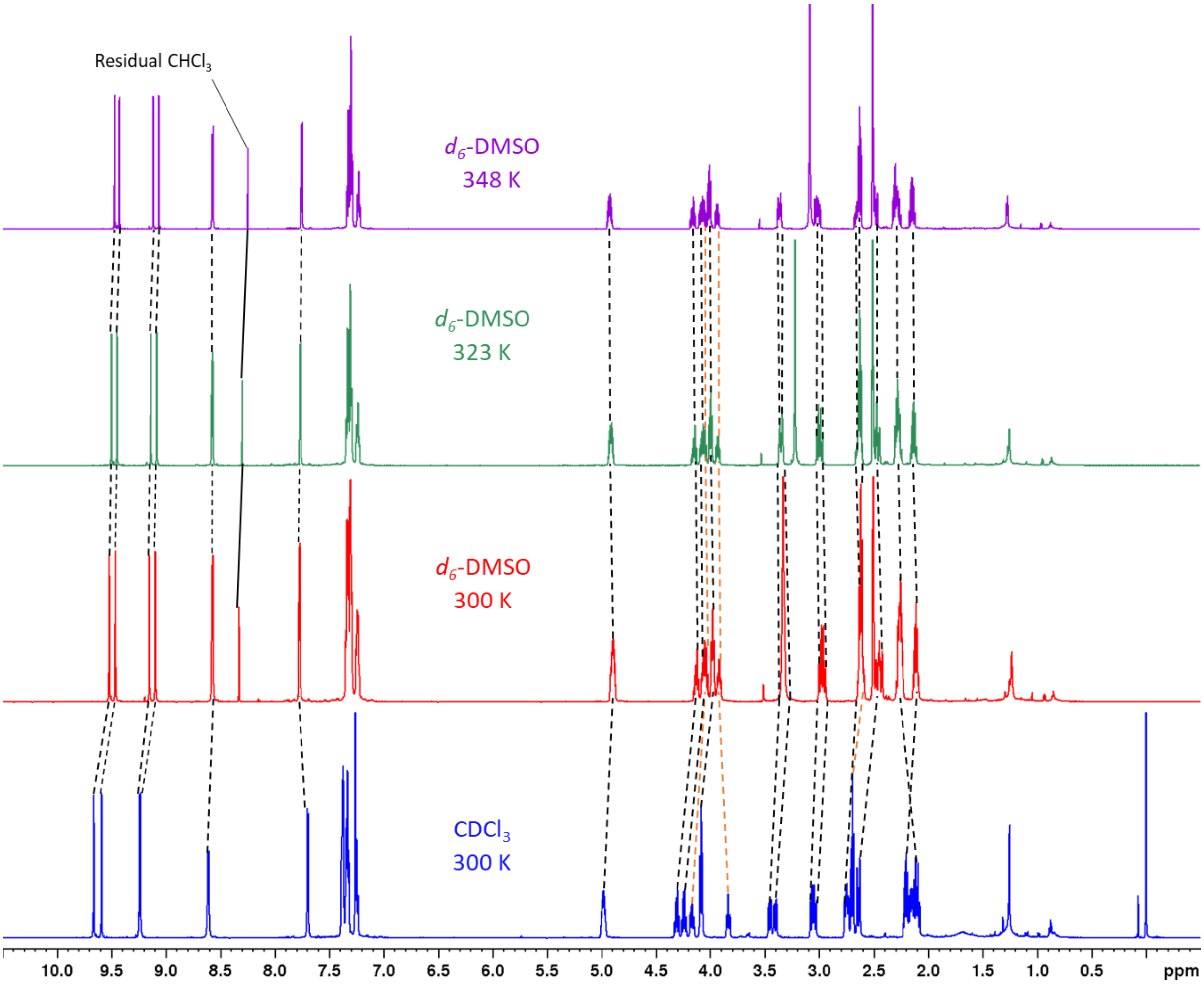


**Figure S9.** Temperature and solvent dependence of $^{1}$H NMR spectra of capped dimer **15** (600 MHz).

**Capped dimer 15**

**ROESY, $d_6$-DMSO, 600 MHz, $t_{mix}$ = 0.2 s**

**300 K**

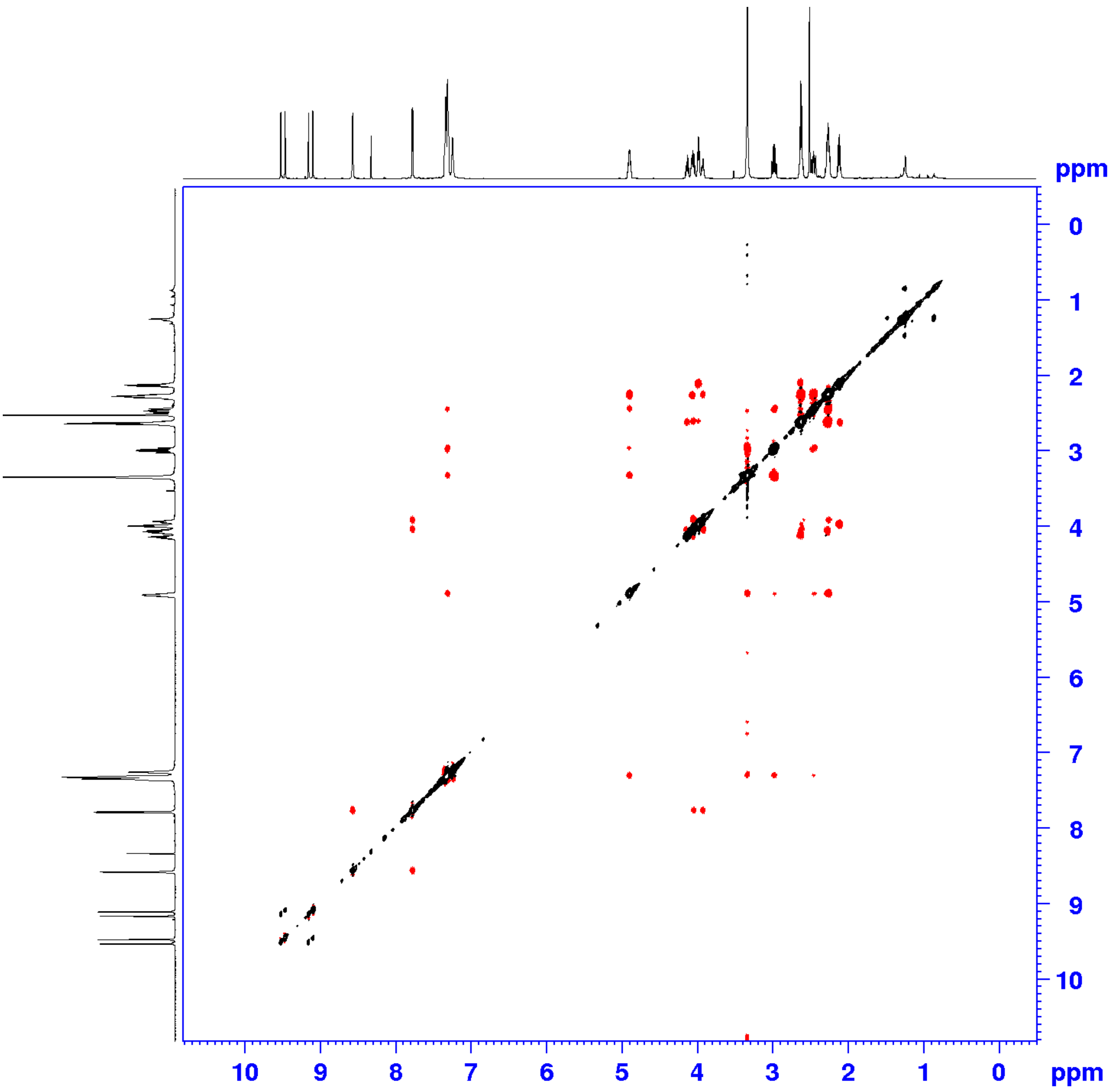

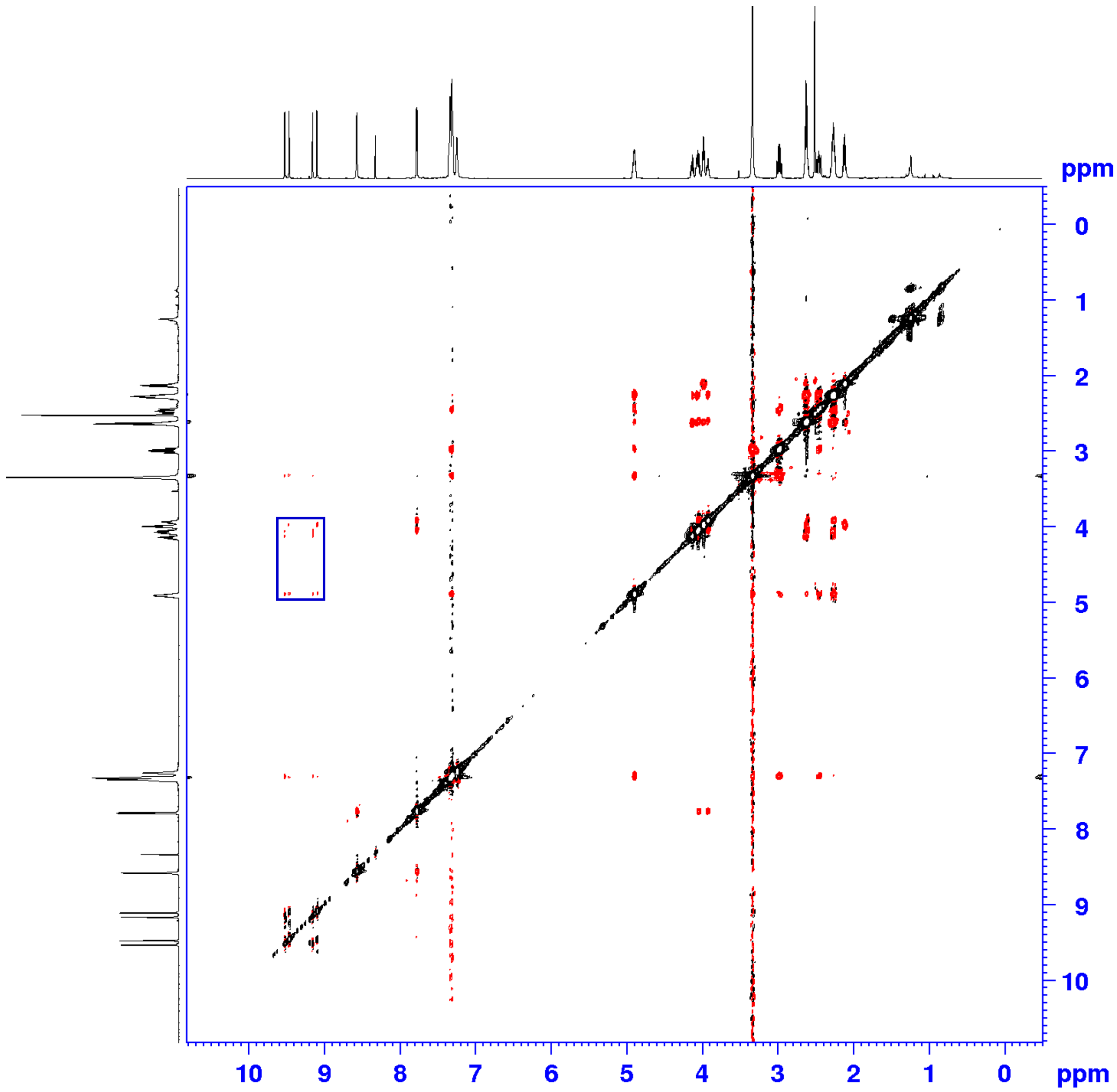

ppm
0
1
2
3
4
5
6
7
8
9
10
10
9
8
7
6
5
4
3
2
1
0
ppm

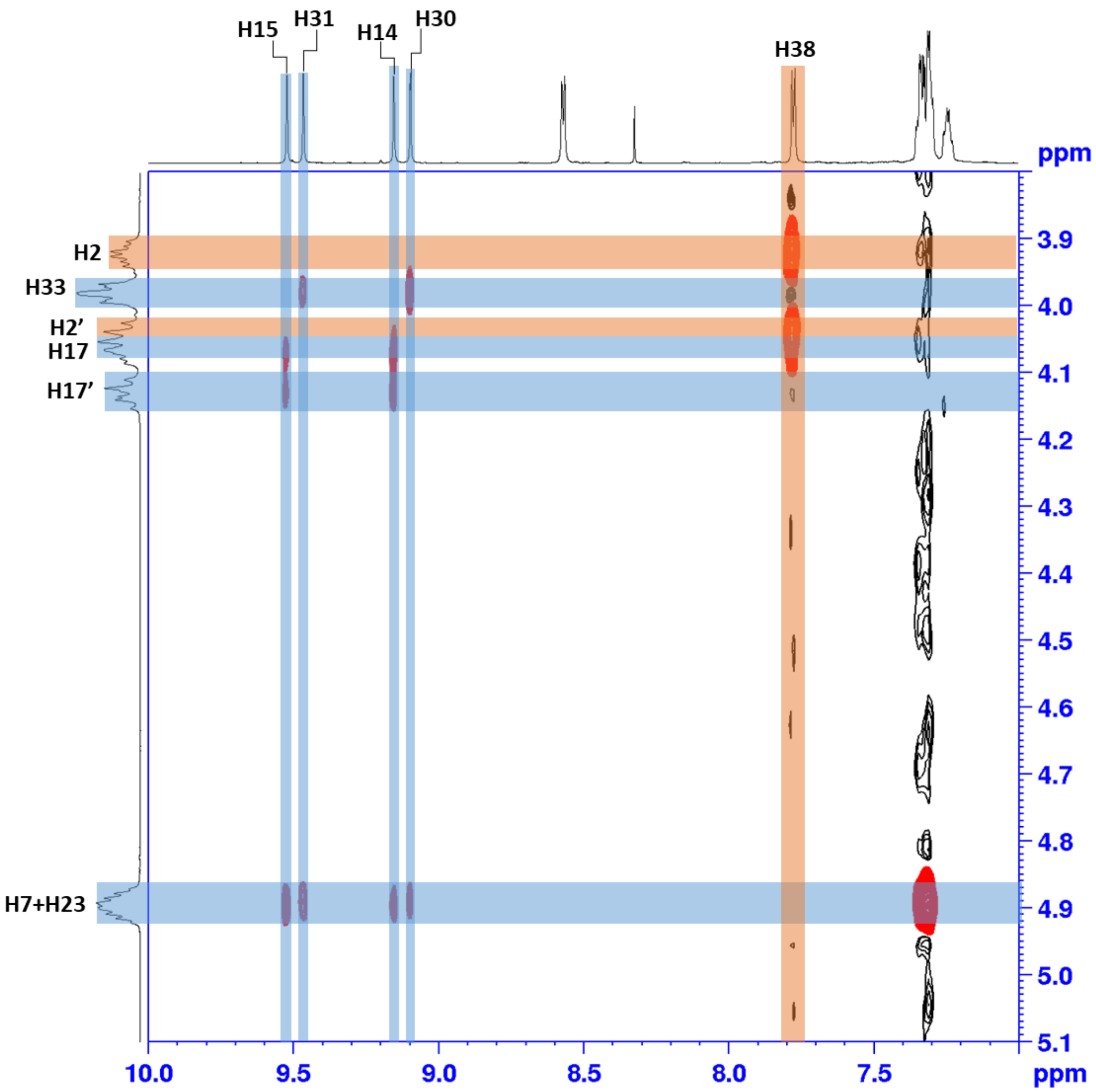
H15
H31
H14
H30
H38
ppm
H2
H33
H2’
H17
H17’
H7+H23
3.9
4.0
4.1
4.2
4.3
4.4
4.5
4.6
4.7
4.8
4.9
5.0
5.1
10.0
9.5
9.0
8.5
8.0
7.5
ppm

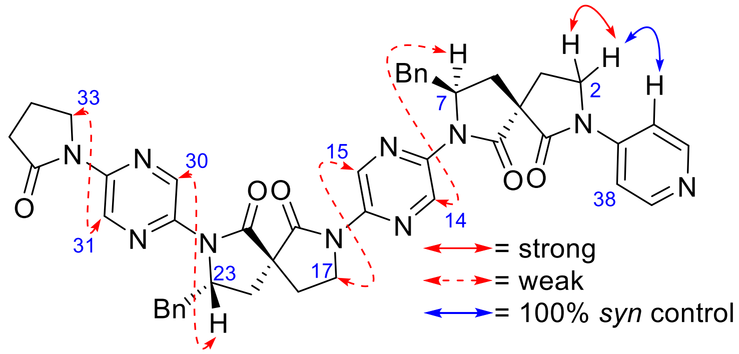


| **Table S8. The effect of solvent polarity and temperature on the dipole repulsion/conformational control for the 4-pyridyl capped dimer 15.** | | | | | | | |
|---|---|---|---|---|---|---|---|
| **Solvent** | **Temperature (K)** | | **4-pyridyl standard (H2↔38)** | **H33[a]↔H31** | **H23↔H30** | **H17[b]↔H15** | **H7↔H14** |
| **$CDCl_3$** | 300 | **NSI** | 0.20 | 0.0012 | [c] | 0.0021 | [c] |
| | | ***A:S*** | - | 99:1 | [c] | 99:1 | [c] |
| ***$d_6$*-DMSO** | 300 | **NSI** | 0.34 | 0.0056 | 0.0092 | 0.0087 | 0.0146 |
| | | ***A:S*** | - | 98:2 | 97:3 | 97:3 | 96:4 |
| ***$d_6$*-DMSO** | 323 | **NSI** | 0.32 | 0.0062 | 0.0108 | 0.0093 | 0.0116 |
| | | ***A:S*** | - | 98:2 | 97:3 | 97:3 | 96:4 |
| ***$d_6$*-DMSO** | 348 | **NSI** | 0.25 | 0.0029 | 0.0050 | 0.0058 | 0.0090 |
| | | ***A:S*** | - | 99:1 | 98:2 | 98:2 | 96:4 |

**NSI:** Normalised standard intensity, **A:S-** *Anti* to *Syn* ratio
[a]: Isochronous peaks, value taken as an average of the two protons
[b]: Value taken as an average of the two protons
[c]: The values of H23↔H30 & H7↔H14 can't be calculated due to overlap between the cross-peaks

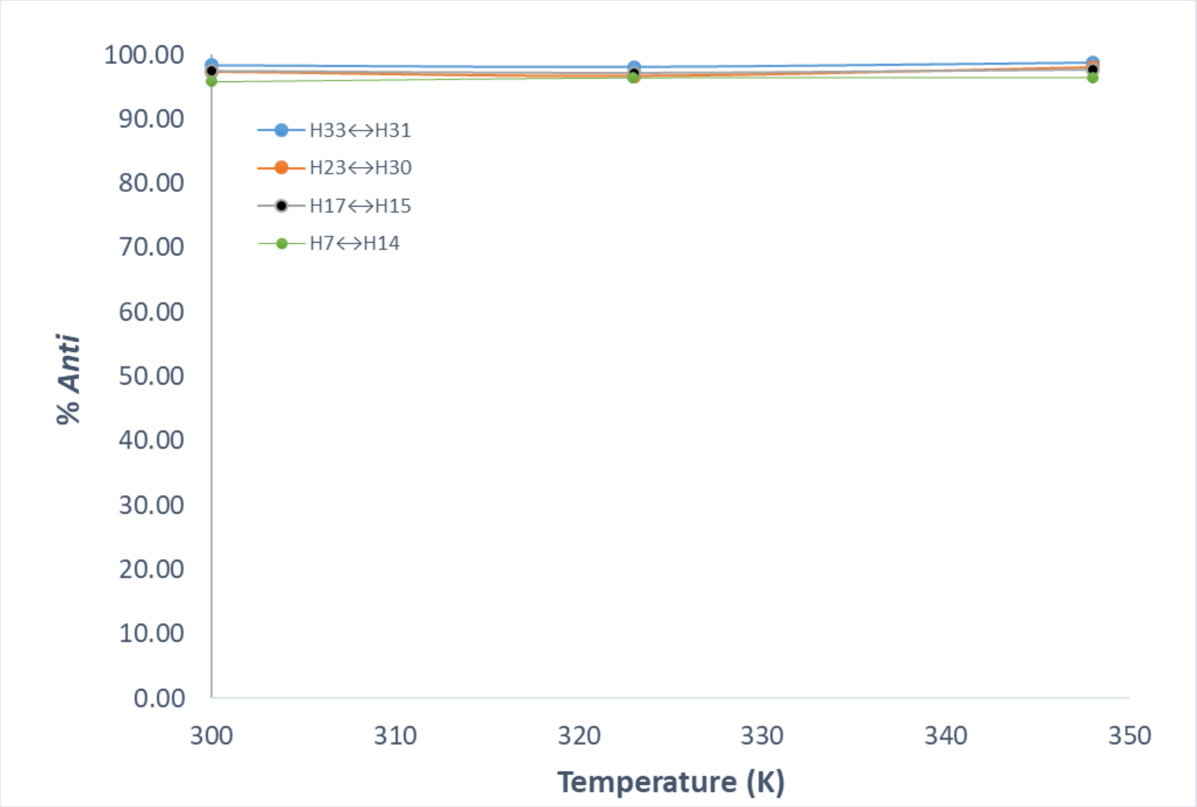


**Figure S10.** The effect of temperature on the dipolar conformational control in the capped dimer **15** in $d_6$-DMSO.

Repeating again the $d_6$-DMSO and variable temperature experiments for the 4-pyridyl capped dimer **15**, we can once again see that the influence of $d_6$-DMSO on the conformational bias. Comparing the *Anti:Syn* of **15** in $CDCl_3$ with the more polar $d_6$-DMSO, (**Table S8**) it had a very slight effect on disrupting the dipole-repelled conformation compared with H33↔H31 decreasing from 99:1 → 98:2, whilst H17↔H15 decreased from 99:1 → 97:3. However, the interactions still remained much weaker than the representative *Syn*-ortho proton (H2↔38), showing that the dipole-opposed conformation is retained.

Looking then at the effect of temperature of the conformational bias whilst in $d_6$-DMSO, between 300 K and 323 K there was very little change in the *Anti:Syn* ratio, (**Table S8 and Figure S10**) with H33↔H31, H23↔H30, H17↔H15, & H7↔H14, all remaining the same. This would therefore suggest the temperature increase had little influence and effect on the rigidity of the backbone and that the dipole-opposed conformation is still retained.

75 °C however seemed to be an anomalous result as the *Anti:Syn* ratio for all of the proton interactions increased compared with rt and 50 °C. This is unexpected and might be a consequence of high temperature resulting in rotation the 4-pyridyl unit and therefore no longer lying truly *syn* relative to H2. The cross-peaks areas however didn't increase which would have indicated that at elevated temperatures the dipole opposed conformation was being diminished.

### 2.1.10 Trimer 11

**ROESY, $CDCl_3$, 600 MHz, $t_{mix}$ = 0.2 s**

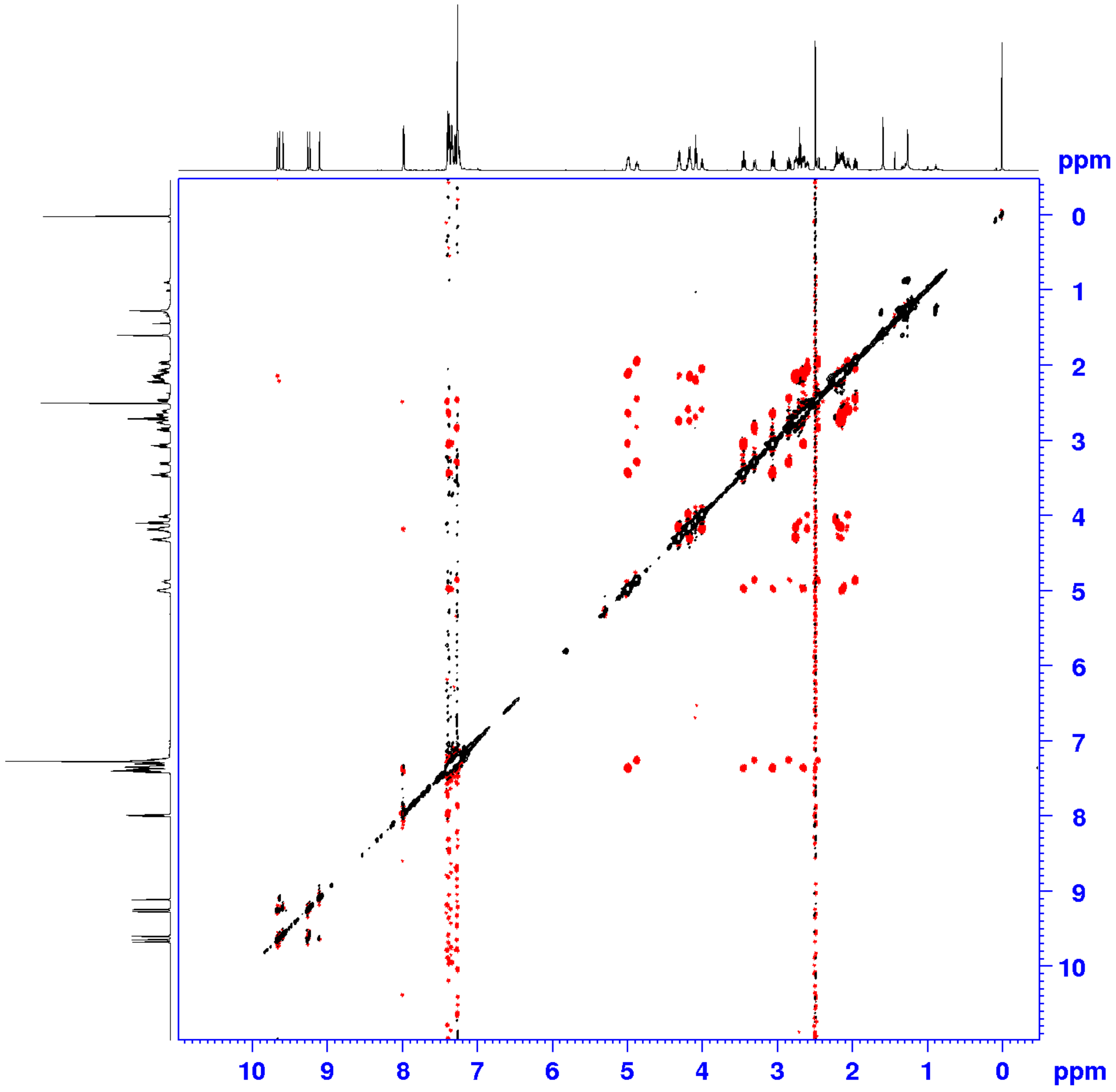

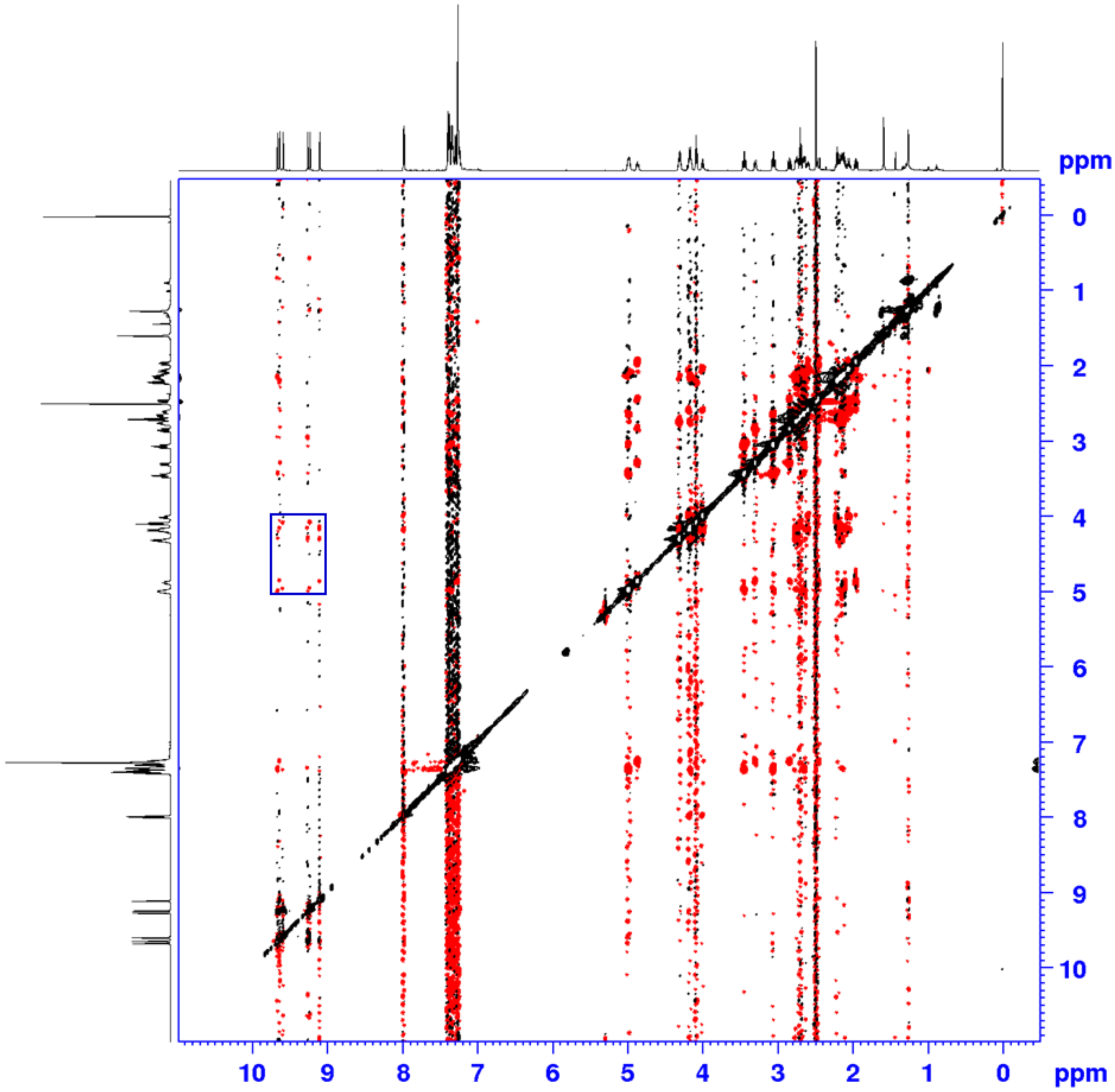
ppm
0
1
2
3
4
5
6
7
8
9
10
10
9
8
7
6
5
4
3
2
1
0
ppm

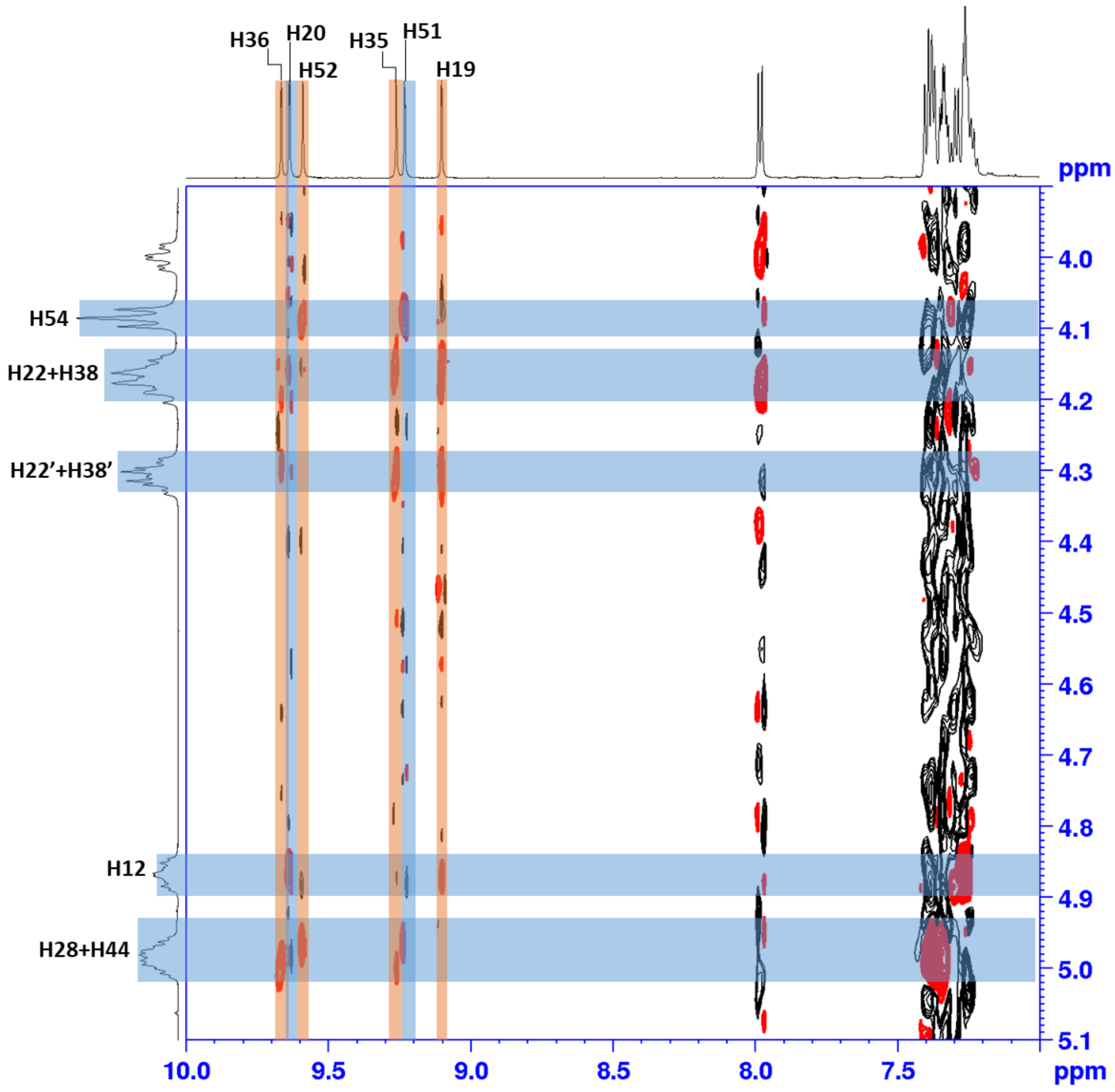

H36
H20
H52
H35
H51
H19
H54
H22+H38
H22'+H38'
H12
H28+H44
ppm
4.0
4.1
4.2
4.3
4.4
4.5
4.6
4.7
4.8
4.9
5.0
5.1
10.0
9.5
9.0
8.5
8.0
7.5
ppm

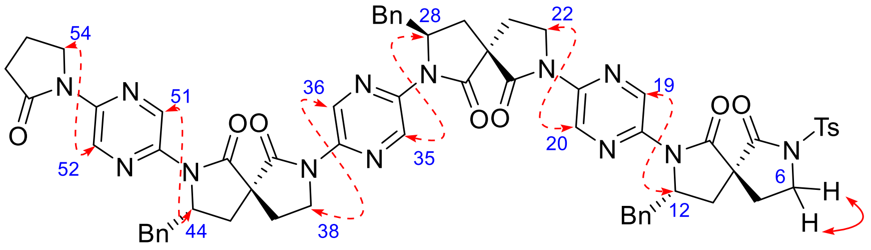


**Table S9. Selected nOe cross-peak intensities of trimer 11 in $CDCl_3$**

| Peak | Normalized intensity |
|---|---|
| H6’-H6 | 1.00 |
| H54[a]-H52 | 0.0034 |
| H44-H51 | 0.0056 |
| H38’-H36 | 0.0016 |
| H38-H36 | 0.0049 |
| H28-H35 | 0.0059 |
| H22’-H20 | 0.0026 |
| H22-H20 | 0.0054 |
| H12-H19 | 0.0034 |

[a] Isochronous peaks, value taken as an average of the two protons

Using the strong nOe between H6’ and H6 as an internal standard for integration of peaks, all of trimer **11**’s normalised interactions are in a similar range and are close to the representative peaks present in both the monomer **7** and dimer **9**. For the capped monomer and dimer, the interaction for the 4-pyridyl ortho proton and lactam’s methylene protons was 0.19 and 0.20 respectively, comparing this to the measured intensities of all of the trimer proton interaction, they are all significantly weaker and is therefore consistent with the proposed dipole-opposed conformation.

### 2.1.11 Deprotected Trimer 12

**ROESY, $CDCl_3$, 600 MHz, $t_{mix}$ = 0.2 s**

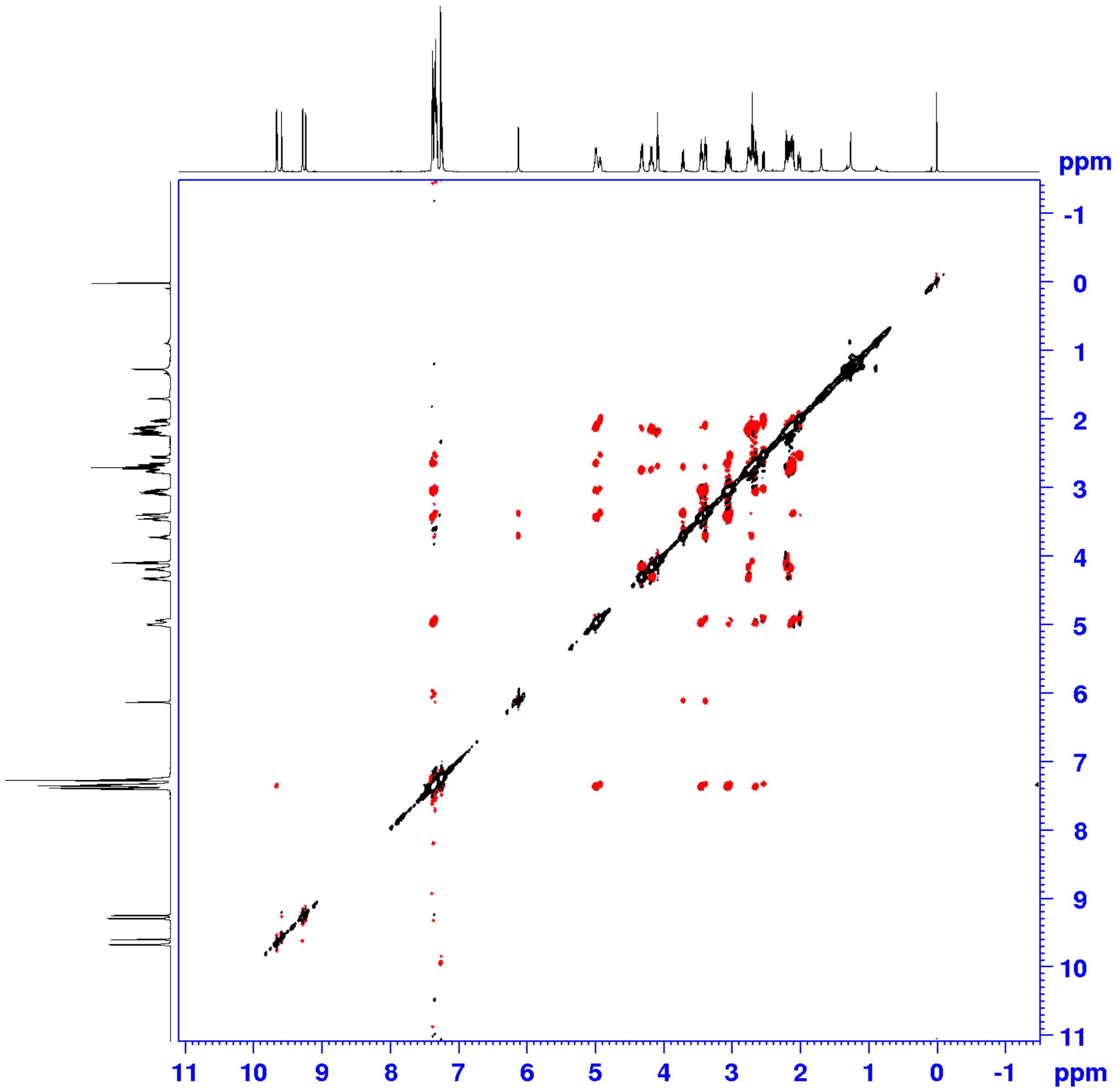

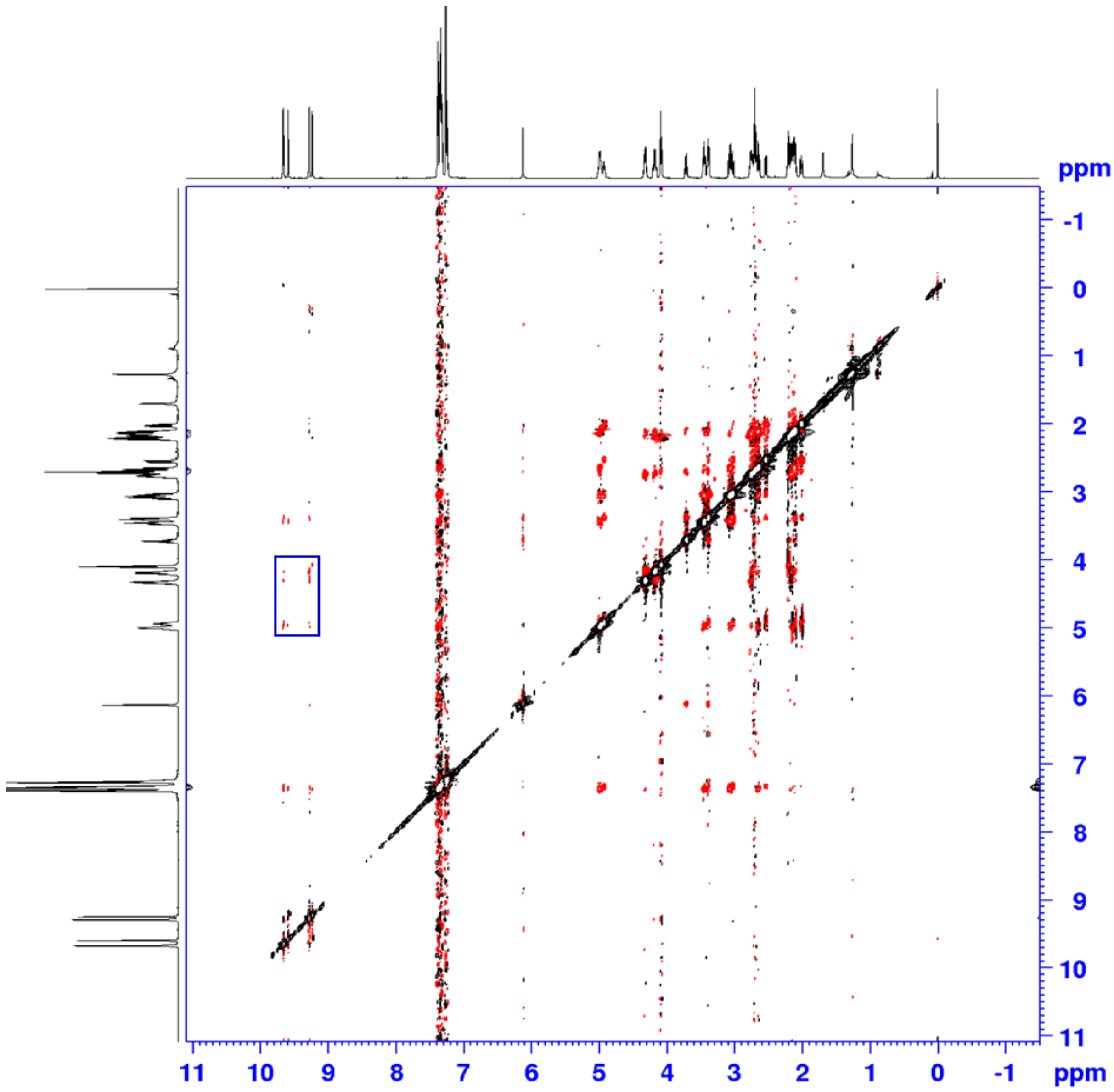
ppm
-1
0
1
2
3
4
5
6
7
8
9
10
11
11
10
9
8
7
6
5
4
3
2
1
0
-1
ppm

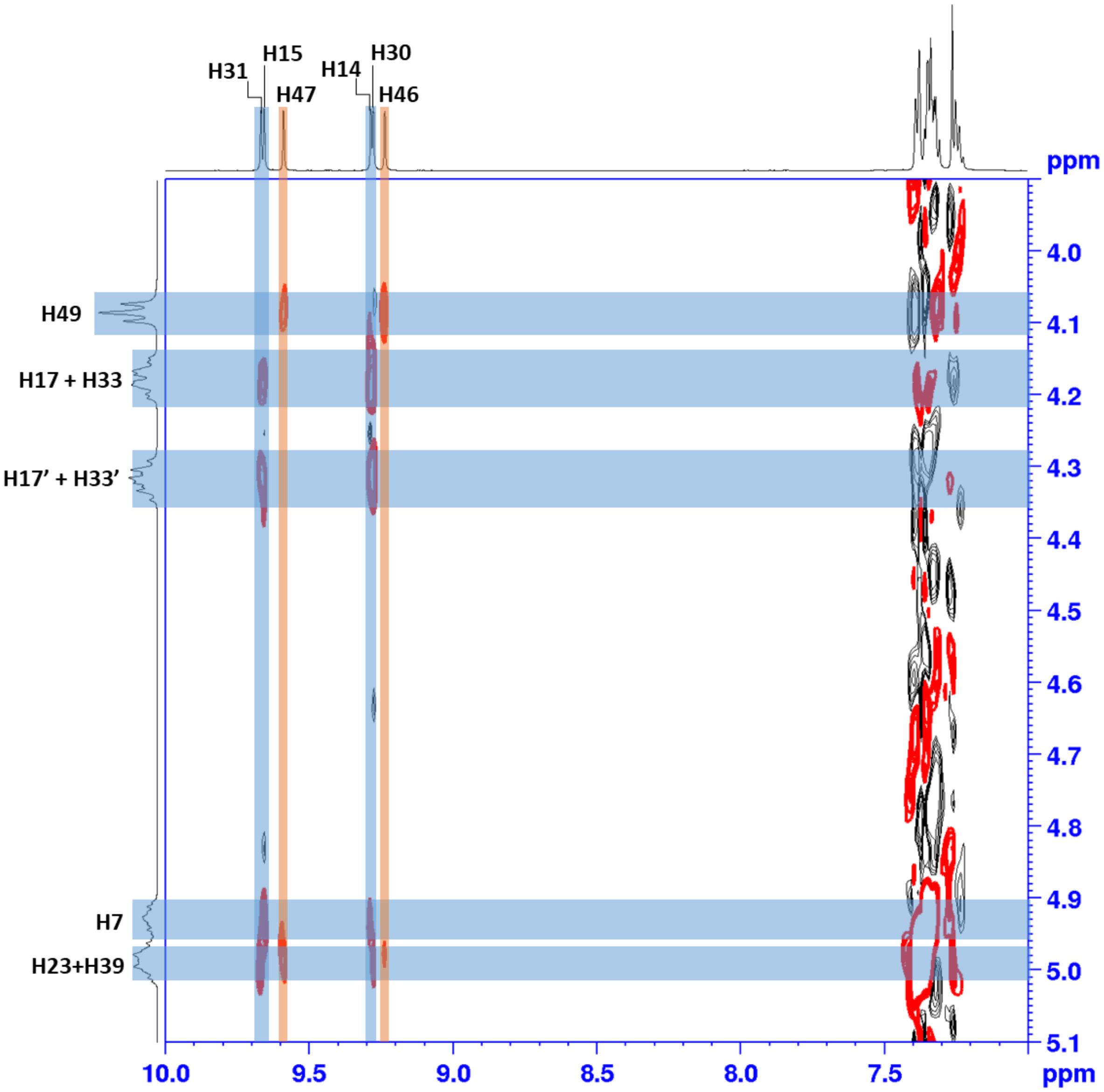

H31
H15
H47
H14
H30
H46
ppm
H49
H17 + H33
H17' + H33'
H7
H23+H39
4.0
4.1
4.2
4.3
4.4
4.5
4.6
4.7
4.8
4.9
5.0
5.1
10.0
9.5
9.0
8.5
8.0
7.5
ppm

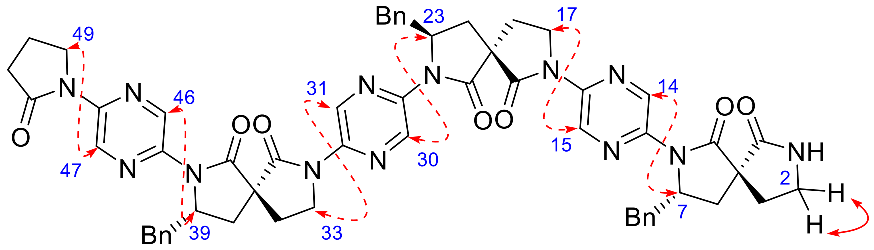


**Table S10. Selected nOe cross-peak intensities of deprotected trimer 12 in $CDCl_3$**

| Peak | Normalized intensity |
|---|---|
| H2'-H2 | 1.00 |
| H49[a]-H47 | 0.0036 |
| H39-H46 | 0.0036 |
| [H33'+H17']-[H31+H15][b] | 0.0118 |
| [H33+H17]-[H31+H15][b] | 0.0078 |
| [H23+H7]-[H30+H14][c] | 0.0084 |

[a] Isochronous peaks, value taken as an average of the two protons
[b] Coalesced cross-peaks could not be unambiguously integrated. The value given is the combined integral for both cross-peaks and is as an average of the two protons
[c] Coalesced cross-peaks could not be unambiguously integrated.

Using the strong nOe between H2' and H2 as an internal standard for integration of peaks, all of trimer **12's** normalised interactions are in a similar range and are close to the representative peaks present in both the monomer **8** and dimer **10**. Due to the similarity in chemical environments for [H33'+H17'], [H33+H17] and [H23+H7], the cross-peaks associated with the respective proton interaction coalesce, see **Table S10**. However, assuming that half the peak area is responsible for each single interaction; 0.0059, 0.0039, 0.0042 respectively, then these values are still in the normal range for an ortho proton in the dipole-opposed conformation.

Again, comparing these interactions with the 4-pyridyl ortho proton and lactam's methylene protons; 0.19 (monomer, **14**) and 0.20 (dimer, **15**), the deprotected trimer **12's** interactions still remain significantly weaker and is therefore consistent with the proposed dipole-opposed conformation.

### 2.1.12 Spiro-urea Hybrid Trimer 17

**ROESY, $CDCl_3$, 600 MHz, $t_{mix}$ = 0.2 s**

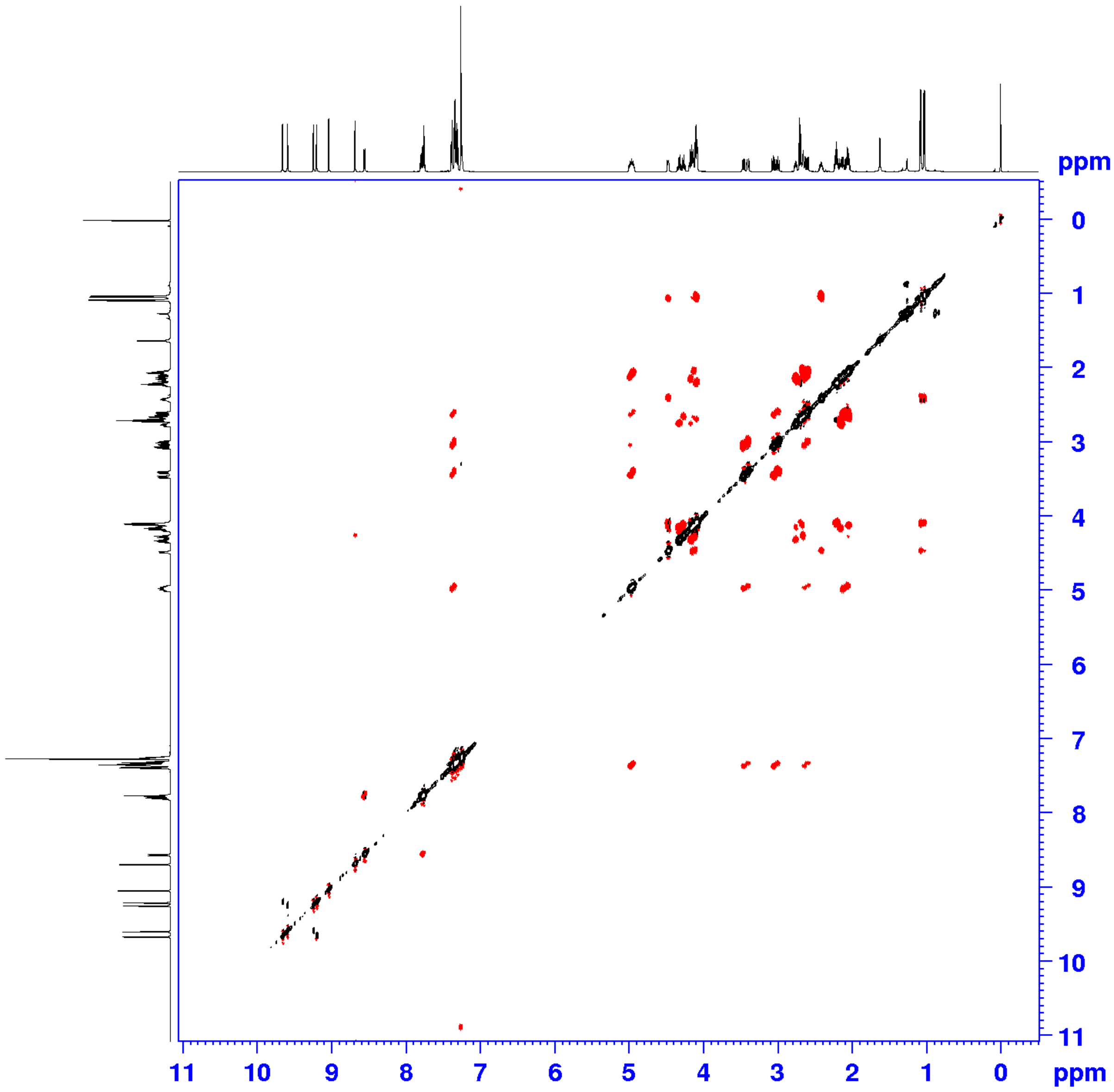

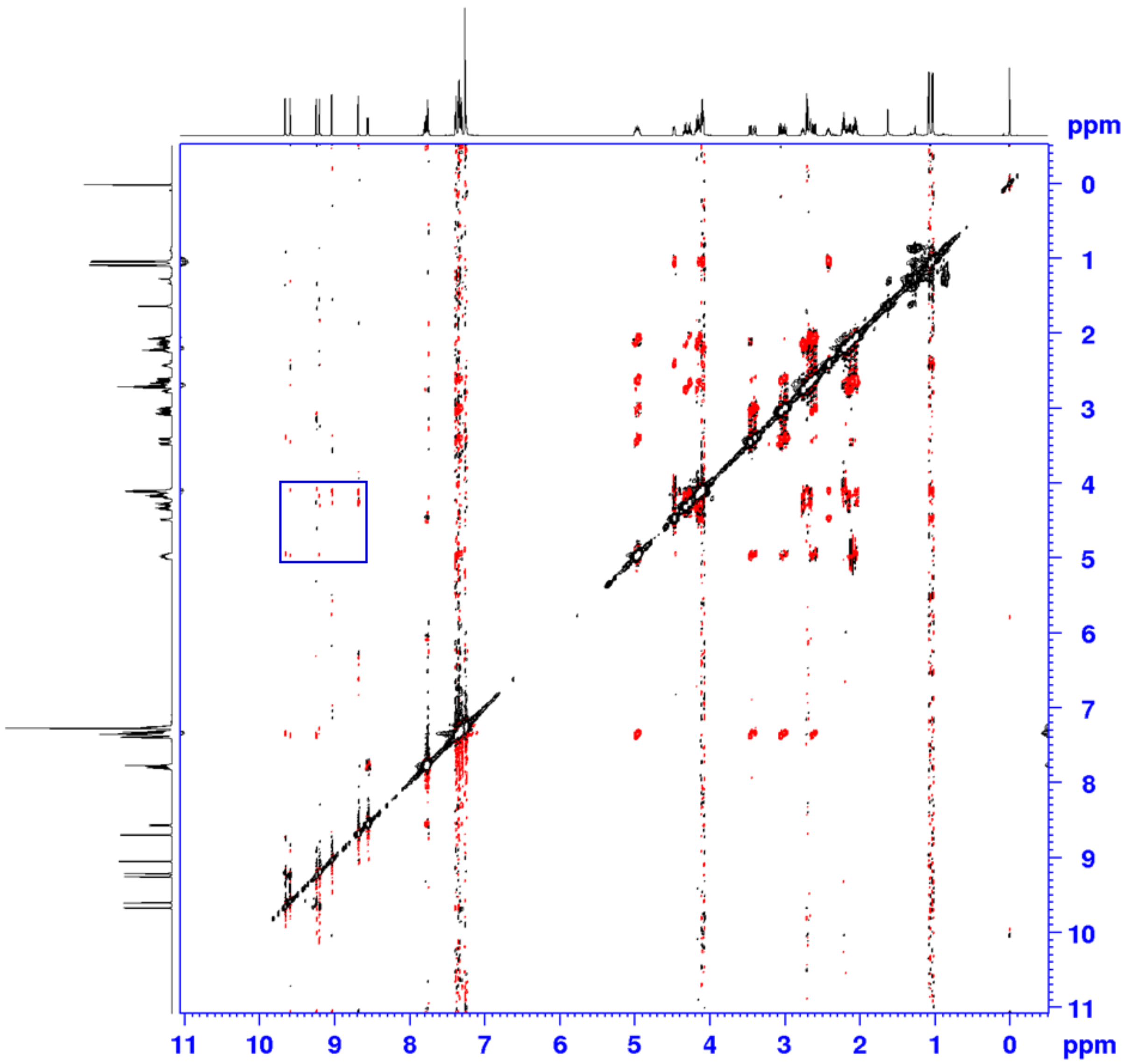
ppm
ppm
0
1
2
3
4
5
6
7
8
9
10
11
11
10
9
8
7
6
5
4
3
2
1
0

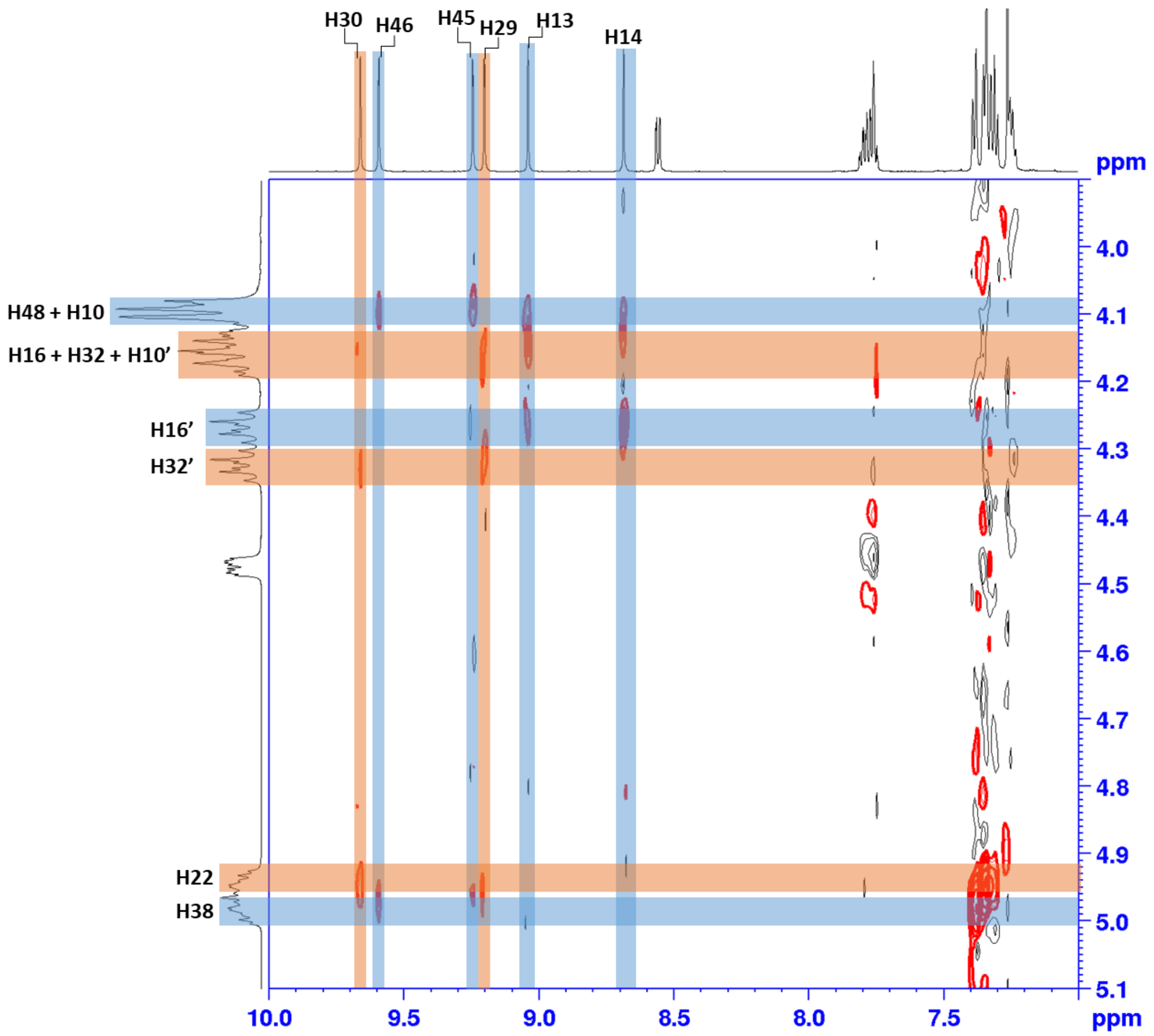
H30
H46
H45
H29
H13
H14
ppm
H48 + H10
H16 + H32 + H10'
H16'
H32'
H22
H38
4.0
4.1
4.2
4.3
4.4
4.5
4.6
4.7
4.8
4.9
5.0
5.1
10.0
9.5
9.0
8.5
8.0
7.5
ppm

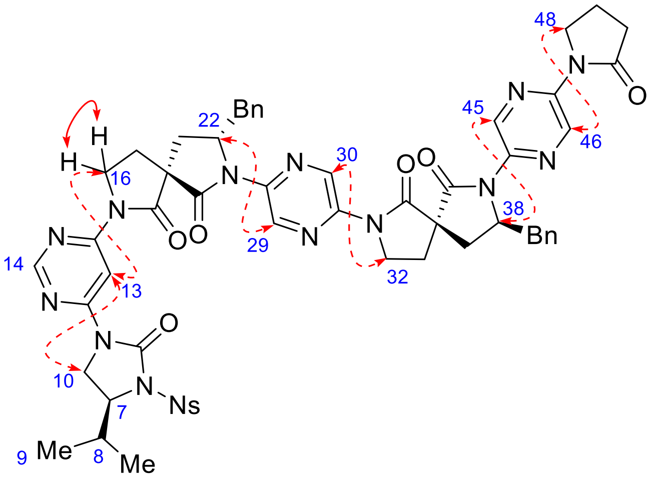


**Table S11. Selected nOe cross-peak intensities of hybrid trimer 17 in $CDCl_3$**

| Peak | Normalized intensity |
|---|---|
| H16’-H16 | 1.00 |
| H48[a]-H46 | 0.0035 |
| H38-H45 | 0.0072 |
| H32’+H30 | 0.0048 |
| H32+H30 | 0.0045 |
| H22-H29 | 0.0051 |
| H16’+H13 | 0.0071 |
| [H16+H10’+H10]- H13[b] | 0.0220 |

[a] Isochronous peaks, value taken as an average of the two protons
[b] Coalesced cross-peaks could not be unambiguously integrated. The value given is the combined integral for both cross-peaks.

Using the strong nOe between H16’ and H16 as an internal standard for integration of peaks, we can see that the normalised intensities for all of the interaction are maintained at a consistent value which are akin in range to those seen throughout for the spirocyclic units of mono/di/trimeric length. Indicating like those species, the dipole-opposed conformation is maintained about C28/31-*N* and C44/47-*N*

As for the urea-pyrimidine unit, in a dipole unopposed conformation, the interaction between H16’-H13 would be enhanced due to H13 lying *syn* to H16, however, this is not the case and the normalized intensity is on par with the values seen throughout for an *anti*-ortho proton. Therefore, dipolar repulsion about C15-*N* is still maintained and favoured upon the addition of the urea-pyrimidine unit. Additionally, although the only unambiguous cross-peak was H16’↔H13, [H16+H13]-[H10+H10'+H13] being made up of three combined interactions still has a per proton interaction of 0.0073, indicating that C12-*N* urea moiety also displays the characteristic dipole-opposed conformation.

### 2.1.13 *pseudo*-hexamer 13

**ROESY, $CDCl_3$, 600 MHz, $t_{mix}$ = 0.2 s**

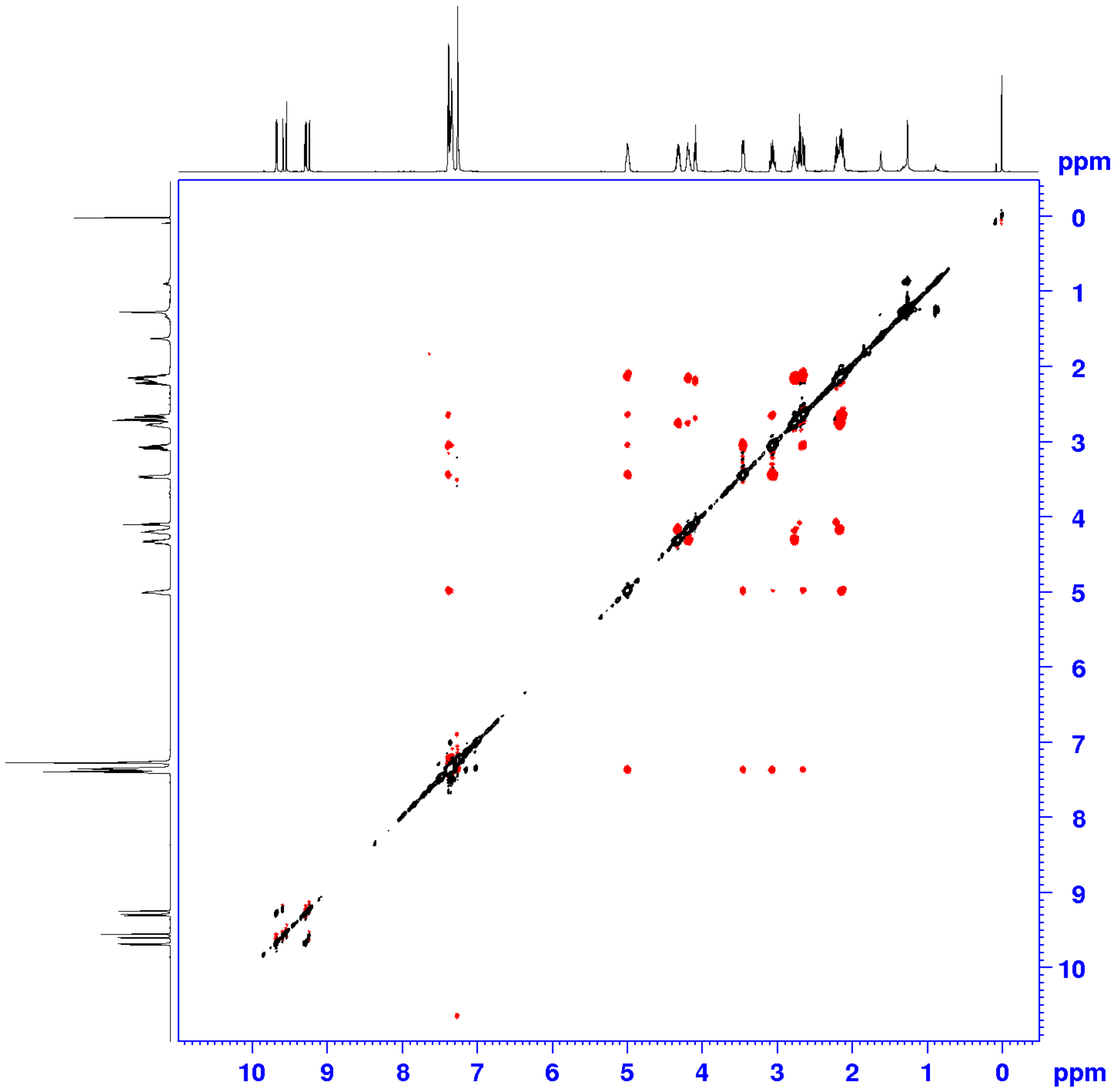

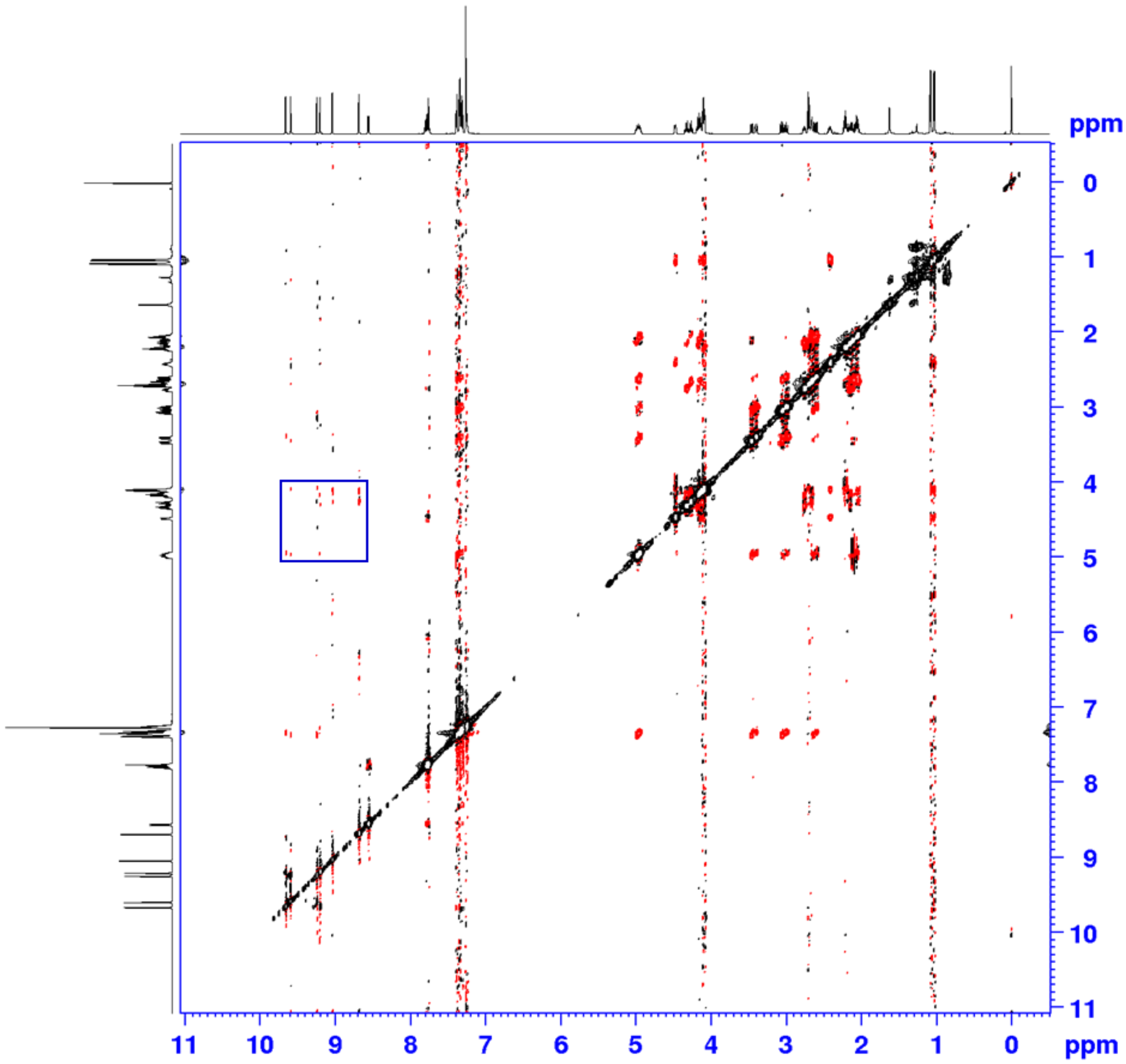
ppm
0
1
2
3
4
5
6
7
8
9
10
11
11
10
9
8
7
6
5
4
3
2
1
0
ppm

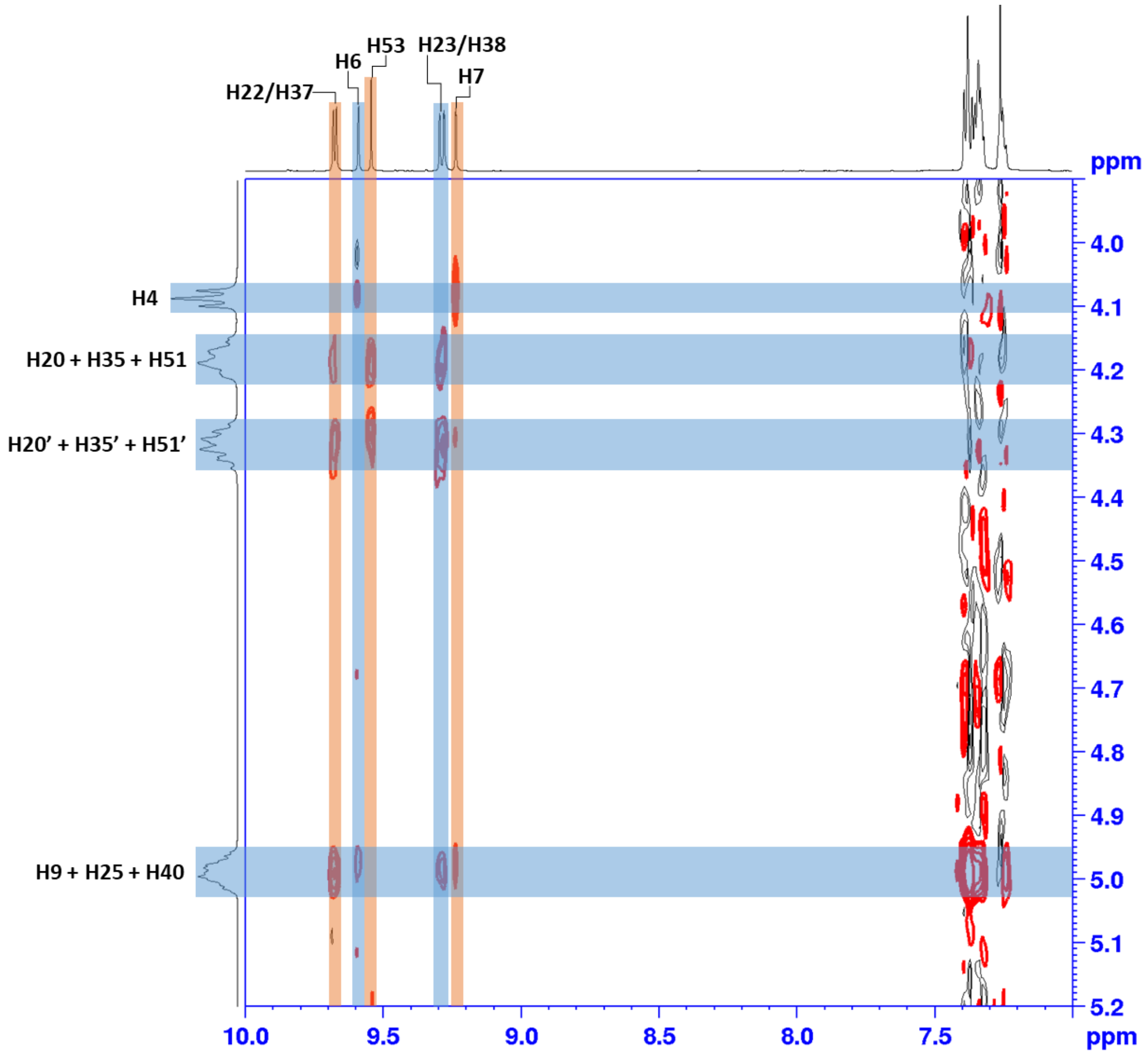
H22/H37
H6
H53
H23/H38
H7
H4
H20 + H35 + H51
H20' + H35' + H51'
H9 + H25 + H40
ppm
4.0
4.1
4.2
4.3
4.4
4.5
4.6
4.7
4.8
4.9
5.0
5.1
5.2
10.0
9.5
9.0
8.5
8.0
7.5
ppm

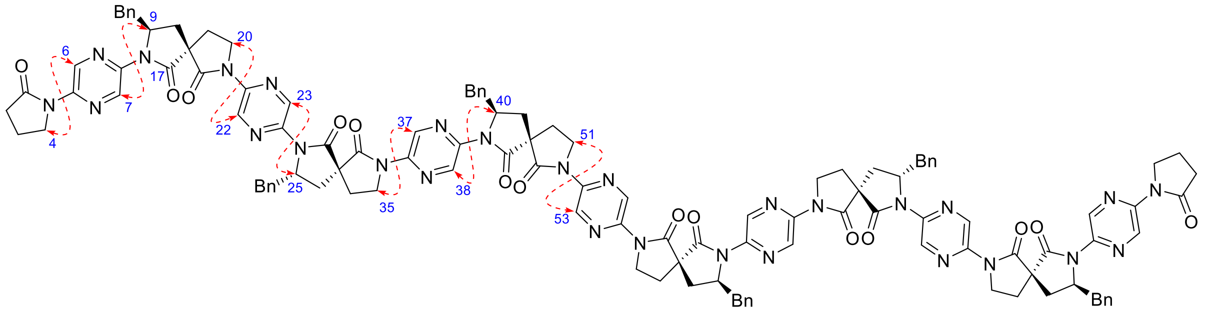

**Table S12. Selected nOe cross-peak intensities of pseudo-hexamer 13 in $CDCl_3$**

| Peak | Normalized Intensity[d] |
|---|---|
| [H20'+H35'+H51']-[H20+H35+H51] | 3.00 |
| H4[a]-H6 | 0.0035 |
| H9-H7 | 0.0078 |
| [H20'+H35']-[H22+H37][b] | 0.0213 |
| [H20+H35]-[H22+H37][b] | 0.0135 |
| [H25+H40]-[H23+H38][c] | 0.0180 |
| H51'-H53 | 0.0210 |
| H51-H53 | 0.0234 |

[a] Isochronous peaks, value taken as an average of the two protons
[b] Coalesced cross-peaks could not be unambiguously integrated, value taken as an average of the two protons
[c] Coalesced cross-peaks could not be unambiguously integrated
[d] In previous analyses all cross-peaks were normalised relative to a single geminal coupling, set to a value of 1.00. In this compound the geminal cross-peaks corresponding to H20-H20', H35-H35' and H51-H51' are co-incident. To obtain comparable normalised values to the prior compounds, nOes in this column were normalised by setting the relative integration of the combined geminal cross-peak to 3

Using the strong nOe between [H20'+H35'+H51']-[H20+H35+H51] as an internal standard for integration of peaks, as seen with all previous other species, the normalised intensities for all of the pseudo-hexamer's interaction maintain consistent values in an intensity indicative of dipole-repulsion being exhibited/enforced throughout the backbone for each spirocyclic unit, with 2-pyrrolidione capped pyrazine experiencing the weakest interactions (H4-H6 and H9-H7 were 0.0035 & 0.0078 respectively), whilst the central pyrazine H51'-H53 had the largest at 0.0210 and 0.0234 which is a reasonable increase. This is likely due to the overall symmetry of the molecule: in general, when the foldamers adopt an *anti*-conformation, weak nOes are observed both to the adjacent *ortho*-pyrazine C-H and to the *meta* C-H. In the case of the interactions of H51, both the *ortho* and *meta* C-Hs on the adjacent pyrazine are symmetry-equivalent (H53), so the observed nOe is the sum of both these interactions.

Overall thought these interactions still demonstrates that even at large and extended backbone lengths; computationally calculated to be ~60 Å, the biasing effect of dipole repulsion is still exhibited and remains a highly controlling force.

## 2.2 Circular Dichromism

Samples were prepared at specified concentrations of either 10 or 100 μM in the solvent indicated, and placed in a 10 mm path length quartz cuvette. Data were acquired between 240 and 400 nm, since below 240 nm $CHCl_3$ absorbs significantly, and preliminary analysis indicated no peaks occurred above 400 nm. The CD spectra were acquired using a data pitch of 1 nm, a continuous scanning speed of 50 nm $min^{-1}$, at three different temperatures (20 °C, 35 °C and 50 °C). Each spectrum was acquired twice, and the data averaged. The resulting raw data was smoothed using the Savitsky-Golay method, with a window size of 25 nm.

### 2.2.1 Homo-Spirobislactam Series at 20 °C

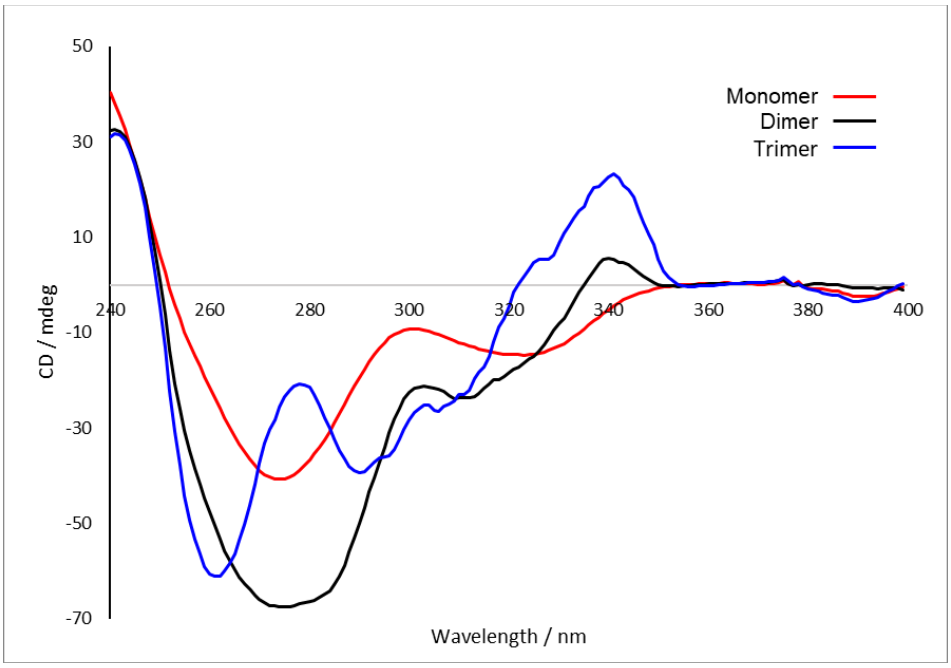


**Figure S11.** CD spectra of monomer **7** (red), dimer **9** (black), and trimer **11** (blue); $CHCl_3$, 100 μM, 20 °C.

Monomer **7**, Dimer **9**, and Trimer **11** all demonstrated qualitatively similar spectra, with monomer **7** and dimer **9** both possessing strong minima at ~275 nm and ~320 nm (**Figure S11**). However, in Trimer **11**, the minima became slight blue-shifted to ~260 nm and ~290 nm. In addition, a new maximum can also be observed for the dimer **9** and trimer **11** at ~ 340 nm, with the intensity of this peak increasing from dimer **9** to trimer **11**.

### 2.2.2 Homo-Spirobislactam Series at 20 °C, 35 °C and 50 °C

CD spectra for all species were also then obtained at 35 °C and 50 °C to observe the influence of temperature on disrupting the dipole repulsion-mediated conformational control, with any change in the appearance of the CD spectra being indicative to a change in the conformation. Pleasing, for all of the examined species the spectra remained consistent over the temperature range, with a minimal decrease in intensity CD/mdeg being observed as the temperature increased. This therefore suggests that the dipolar repulsion is still exerting highly rigid conformational control at the examined temperatures, and that the conformation is not being disrupted. This is in good agreement with the VT-NMR study of capped monomer **14** and dimer **15**, which experienced a minimal change in nOe intensity over a temperature of 27 to 75 °C in $d_6$-DMSO. For an overlay of each individual species at 20 °C, 35 °C, and 50 °C, see CD spectra bellow.

**Monomer 7**

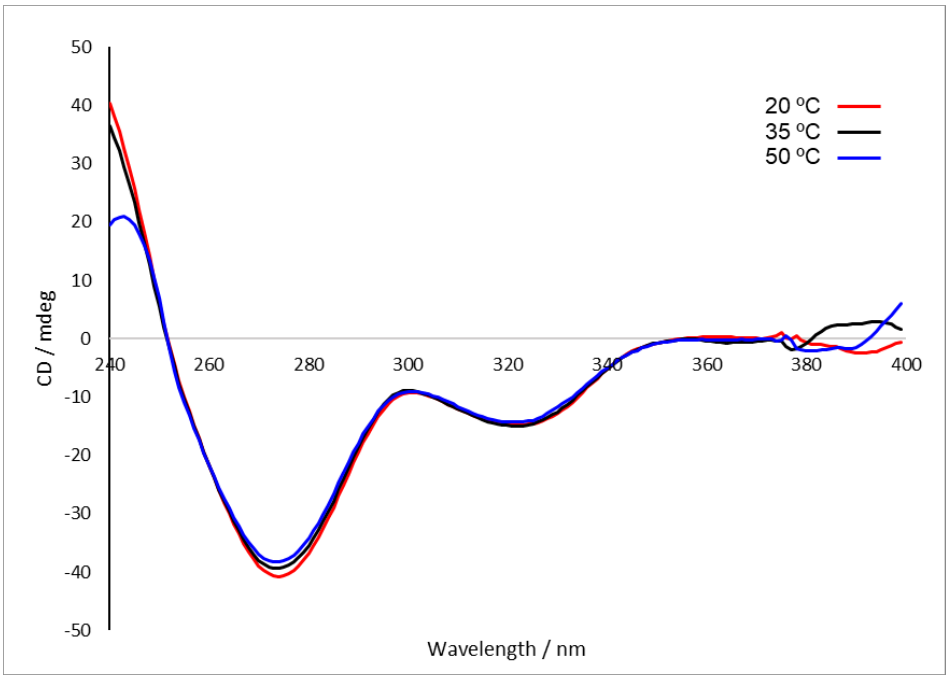


**Figure S12.** CD spectra of momomer **7** taken at 20 °C (red), 35 °C (black), and 50 °C (blue); $CHCl_3$, 100 μM.

Monomer **7** contained strong minima at 275 nm and 320 nm, with all spectra remaining consistent over the specified temperature range, and a minimal decrease in intensity CD/mdeg being observed as the temperature increase (**Figure S12**).

**Dimer 9**

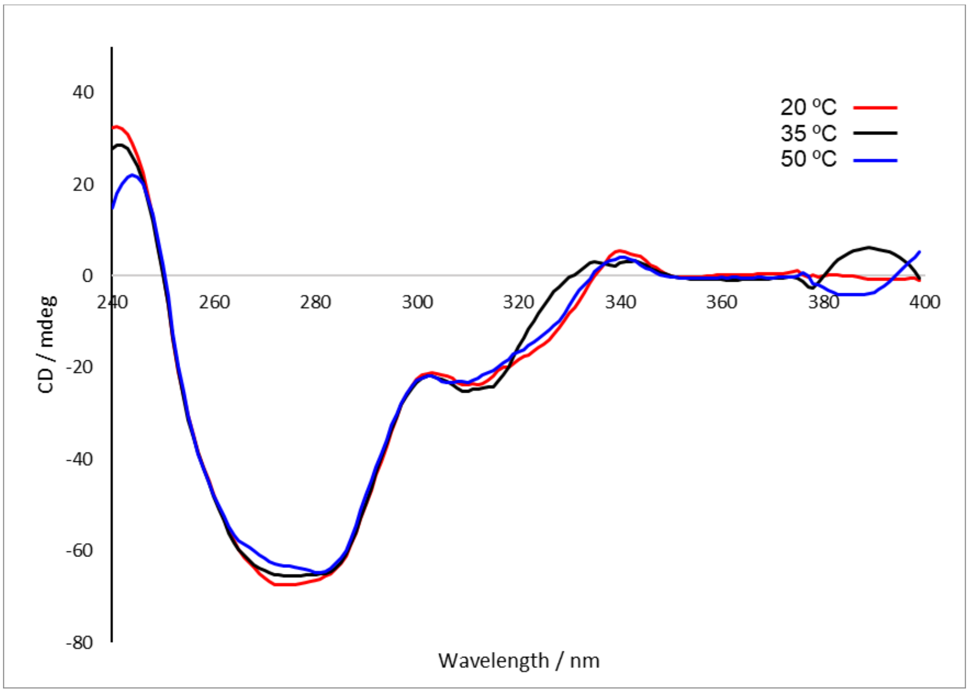


**Figure S13.** CD spectra of Dimer **9** taken at 20 °C (red), 35 °C (black), and 50 °C (blue); $CHCl_3$, 100 µM.

Dimer **9** contained strong minima at 275 nm and 315 nm and maximum at 340 nm, with all spectra remaining consistent over the specified temperature range, and a minimal decrease in intensity CD/mdeg being observed as the temperature increase (**Figure S13**).

**Trimer 11**

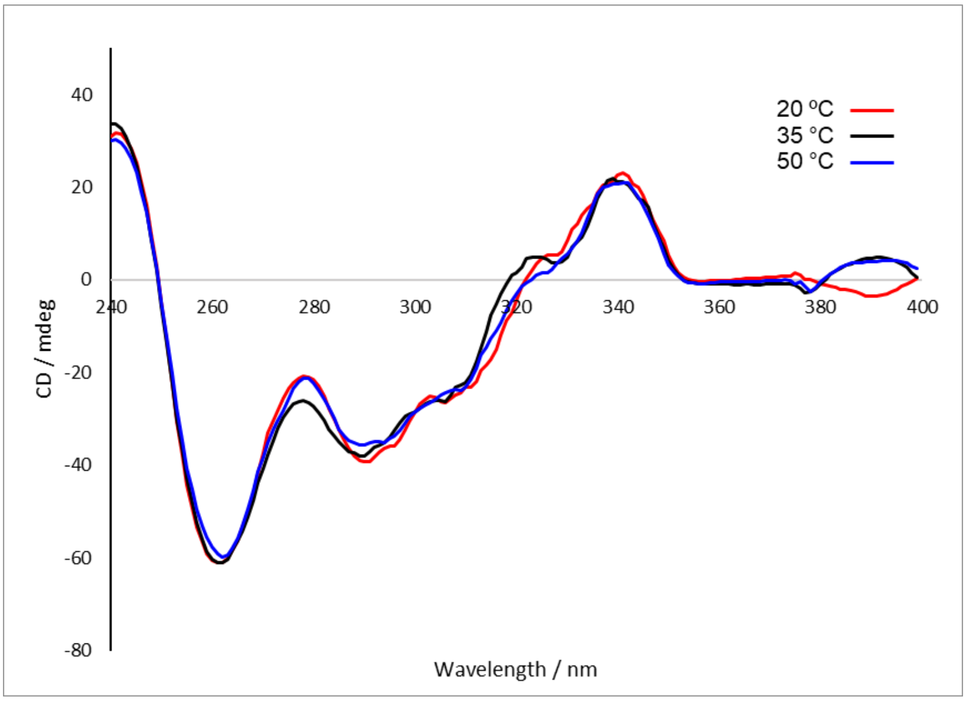


**Figure S14.** CD spectra of trimer **11** taken at 20 °C (red), 35 °C (black), and 50 °C (blue); $CHCl_3$, 100 μM.

Trimer **11** contained strong minima at 260 nm and 290 nm and maximum at 340 nm, with all spectra remaining consistent over the specified temperature range, and a minimal decrease in intensity CD/mdeg being observed as the temperature increase (**Figure S14**).

**Pseudo hexamer 13**

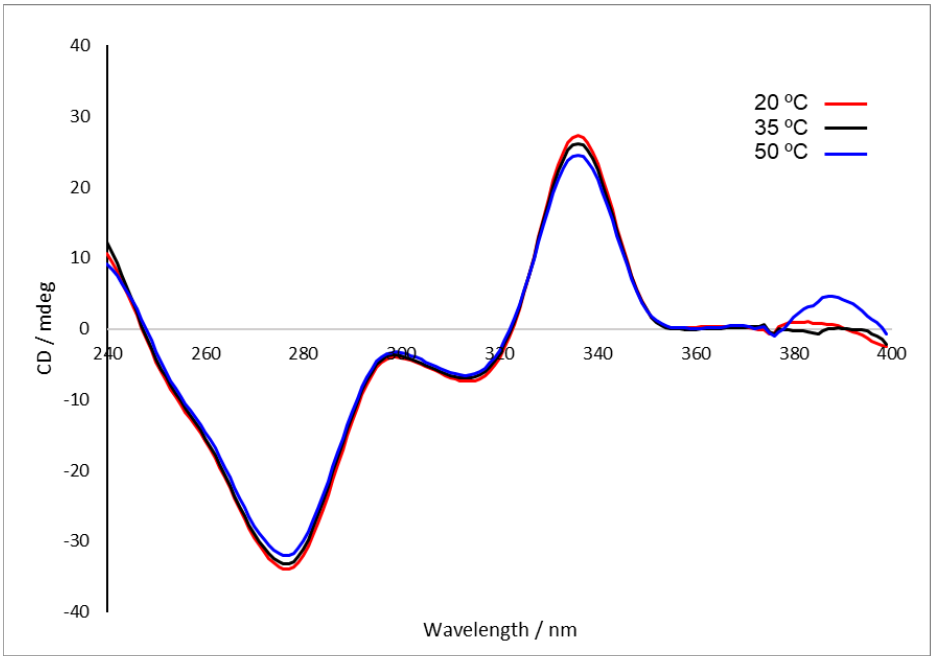


**Figure S15.** CD spectra of pseudo-hexamer **13** taken at 20 °C (red), 35 °C (black), and 50 °C (blue); $CHCl_3$,10 μM.

Due to the intensity of CD spectra of pseudo-hexamer **13**, a concentration of 10 μM was required, as a concentration above 25 μM resulted in too little light reaching the detector (**Figure S15**). Comparing the CD spectra of pseudo-hexamer **13** with the rest of the homo series, it possessed a qualitatively similar spectrum, with minima at ~275 nm and ~320 nm and maximum at 340 nm being observed. As four residues of the spirocyclic backbone are required to create a full turn of the *P*-helix, and pseudo-hexamer **13** possessing 1.5 turns, the peaks at the minimum of ~280 nm and 315 nm and maximum at 340 nm, may be diagnostic of the formation of a (*P*)-helix.

In addition to this, pseudo-hexamer **13**'s spectra also remained consistent over the examined temperature range (20 °C, 35 °C, 50 °C), again demonstrating that even at large and extended backbone lengths; computationally calculated to be ~60 Å, the dipolar-repulsion is dominant enough to retain the highly rigid conformation at elevated temperatures.

### 2.2.3 Hybrid trimer 17

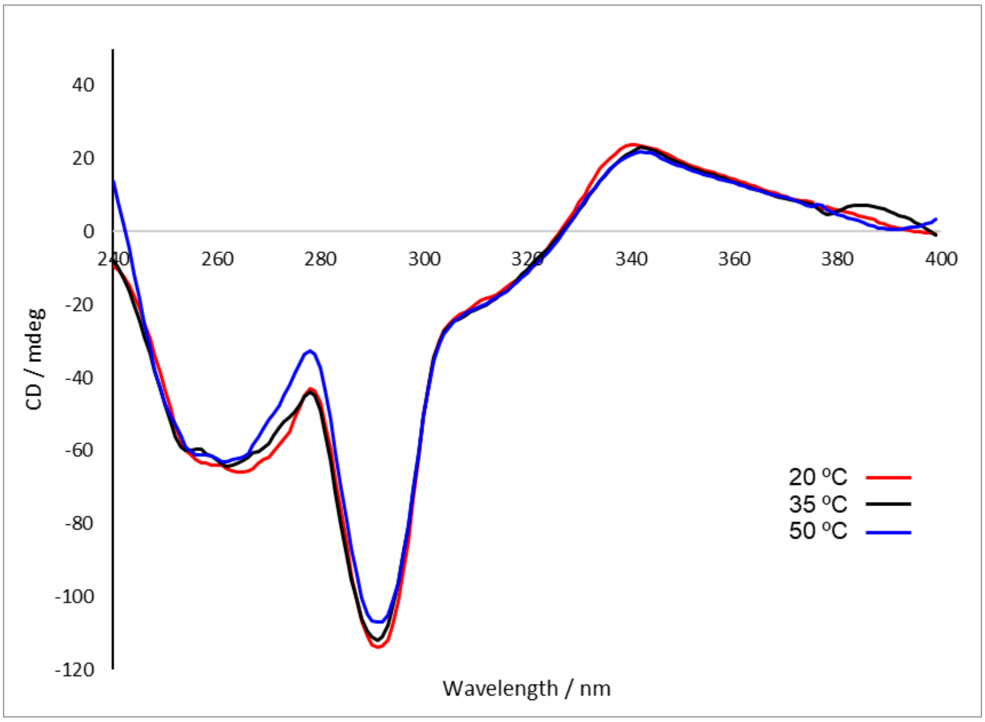


**Figure S16.** CD spectra of hybrid-trimer **17** taken at 20 °C (red), 35 °C (blue), and 50 °C (black); $CHCl_3$,100 μM.

Hybrid trimer **17**, although containing a urea-pyrimidine unit, the spectrum remained very closely related to the homo-series, with minima at ~260 nm and ~290 nm and maximum at 340 nm, akin to those seen trimer **15** (**Figure S16**). Much like all the other species, hybrid trimer **17**, still retained its highly conformational controlled structure over the analysed temperature range, with the spectra remaining consistent and a minimal decrease in intensity CD/mdeg being observed.

## 2.3 X-ray Crystallography

### 2.3.1 Single Crystal Data for 7 (CCDC 2170496)

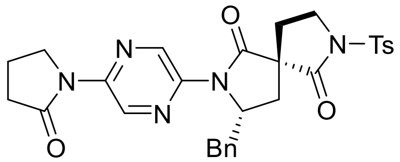


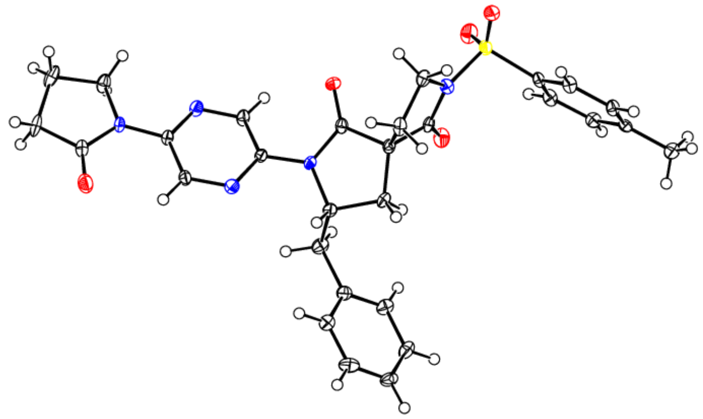

| | |
|---|---|
| Crystal data | |
| Chemical formula | $C_{29}H_{29}N_5O_5S$ |
| $M_r$ | 559.63 |
| Crystal system, space group | Orthorhombic, $P2_12_12_1$ |
| Temperature (K) | 100 |
| *a*, *b*, *c* (Å) | 5.65890 (1), 12.302100 (17), 37.31390 (2) |
| *V* (Å$^3$) | 2597.66 (1) |
| *Z* | 4 |
| Radiation type | Cu *K*α |
| μ (mm$^{-1}$) | 1.54 |
| Crystal size (mm) | 0.28 × 0.06 × 0.06 |
| Data collection | |
| Diffractometer | Oxford Diffraction SuperNova |
| Absorption correction | Multi-scan<br>*CrysAlis PRO* (Rigaku Oxford Diffraction, 2017) |
| $T_{min}$, $T_{max}$ | 0.68, 0.91 |
| No. of measured, independent and<br>observed [$I > 2.0\sigma(I)$] reflections | 23951, 4978, 4764 |
| $R_{int}$ | 0.000 |
| $(\sin \theta/\lambda)_{max}$ (Å$^{-1}$) | 0.615 |
| Refinement | |
| $R[F^2 > 2\sigma(F^2)]$, $wR(F^2)$, $S$ | 0.051, 0.126, 0.95 |
| No. of reflections | 4978 |
| No. of parameters | 362 |
| H-atom treatment | H-atom parameters constrained |
| $\Delta\rho_{max}$, $\Delta\rho_{min}$ (e Å$^{-3}$) | 0.66, −0.68 |

| | |
|---|---|
| Absolute structure | Parsons, Flack & Wagner (2013), 2015 Friedel Pairs |
| Absolute structure parameter | 0.012 (11) |

## 2.4 Computation

### 2.4.1 General Method

The lowest energy conformation of sidechain truncated ($CH_3$) analogues of **7**, **9**, **11** and **13** were determined computationally. This was carried out using the open-source VegaZZ software package.[14] Unconstrained conformational searching was conducted using molecular mechanics: the AMMP algorithm under Boltzmann jump conditions ($T$ =300 K, RMSD = 60, dielectric constant = 4.72) with the SP4 force field and standard bonding, angle, and non-bonding interaction potentials. The lowest energy structure thus obtained was minimised using semi-empirical methods (MOPAC 2016,[15] PM7 method[16]). Computed lowest energy structures as shown in Figures 3, S17-S21 are available in .mol format from www.doi.org/10.6084/m9.figshare.22270771.

### 2.4.2 Validation

This approach was initially validated by minimization of monomer **7**, for which single crystal data were available (**Figure S17**). The structure obtained from a molecular mechanics approach alone (10,000 iterations) displayed the correct, dipole-opposed conformation, but with a much larger torsion around the $N_{lactam}$-$C_{pyrazine}$ bond, presumably because this method does not adequately account for π-conjugation effects. Further minimization of this structure using a semi-empirical method (PM7) led to much greater planarity at these linkages, and to close agreement with the single crystal structure, with the exception of the N-Ts terminal group. Pair-fitting of atomic coordinates in the minimized structures *versus* the single crystal structure allowed quantification of this better agreement, with an RMSD of 0.55 Å for the MM, and 0.25 Å for the semi-empirical minimum.

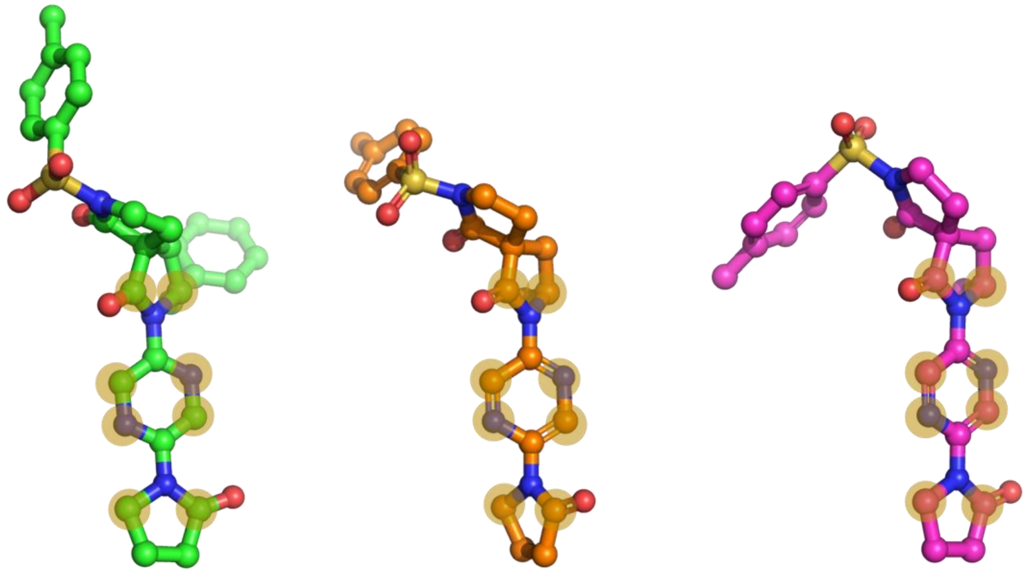

***single crystal***

***computed structure***
Molecular mechanics (SP4)
(sidechain truncated to $CH_3$)
**rmsd = 0.55 Å**

***computed structure***
Semi-empirical (PM7)
(sidechain truncated to $CH_3$)
**rmsd = 0.25 Å**

**Figure S17.** Left: single crystal structure of **7**. Middle: lowest energy conformation of **7** (sidechain truncate) obtained using MM methods. Right: lowest energy conformation of **7** (sidechain truncate) obtained using semi-empirical methods. Pair-fitting was conducted between the atoms indicated in yellow.

### 2.4.3 Linear Conformational Searching – An Accelerated Protocol

Since each $N_{lactam}$-$C_{pyrazine}$ linkage is distant from the adjacent linkages, we reasoned that rotation about each axis could be carried out *independently*. This would vastly reduce the time required for conformational searching since it would allow the search to scale linearly rather than exponentially with the size of the oligomer.

This was initially tested on trimer **11** (**Figure S18**). First, the relevant torsions were identified and added to the list of rotatable bonds within the AMMP conformational search function (for example, in *pseudo*-hexamer **13** there are 7 independent $N_{lactam}$-$C_{pyrazine}$ linkages). When the conformational search was conducted under Boltzmann jump conditions, 50,000 iterations were necessary to locate a global minimum where the dipole opposed conformation was displayed at all C-N linkages. This minimum was then subjected to semi-empirical minimisation using MOPAC (PM7).

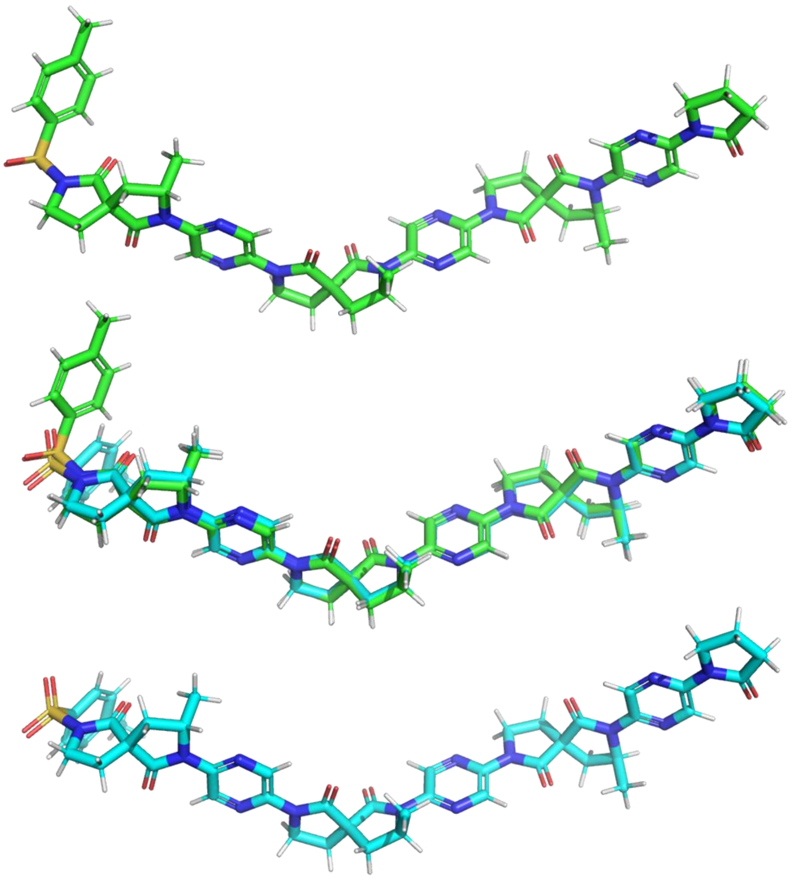

*MM (Boltzmann 50k steps) then PM7*

*overlay*

*MM (linear search, 168 steps) then PM7*

**Figure S18**. Comparison of minimized structures obtained from Boltzmann-weighted random search (top) and systematic linear search of each rotatable bond independently (bottom).

The linear search approach was then tested: each rotatable bond was selected and subjected to a systematic conformational search (0 to 360°, 24 steps). Once the conformational search was completed for all bonds, the minimum obtained was subjected to semi-empirical minimization using MOPAC (PM7). The conformer thus obtained was virtually identical to that found by the Boltzmann-weighted random search (with the exception of the tosyl protecting group). By contrast with the Boltzmann approach, the linear method located the same minimum by sampling just 24 x 7 = 168 conformations, and can be completed in mere minutes.

### 2.4.4 Monomer 7

Energy minimum located by the Boltzmann weighted random search approach in 10,000 iterations (**Figure S19**).

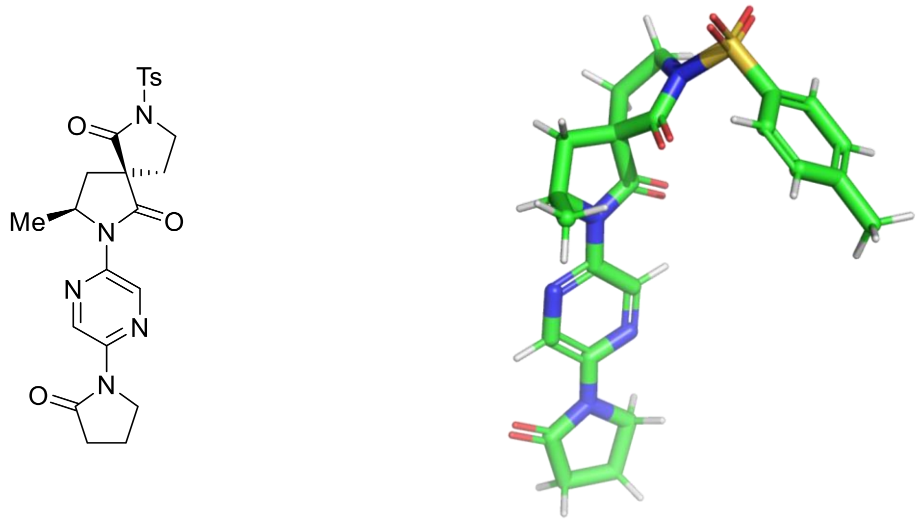

**Figure S19**. Energy-minimised structure of monomer **7** (PM7). Side-chains truncated to $CH_3$ for computational brevity.

### 2.4.5 Dimer 9

Energy minimum located by the Boltzmann weighted random search approach in 10,000 iterations (**Figure S20**).

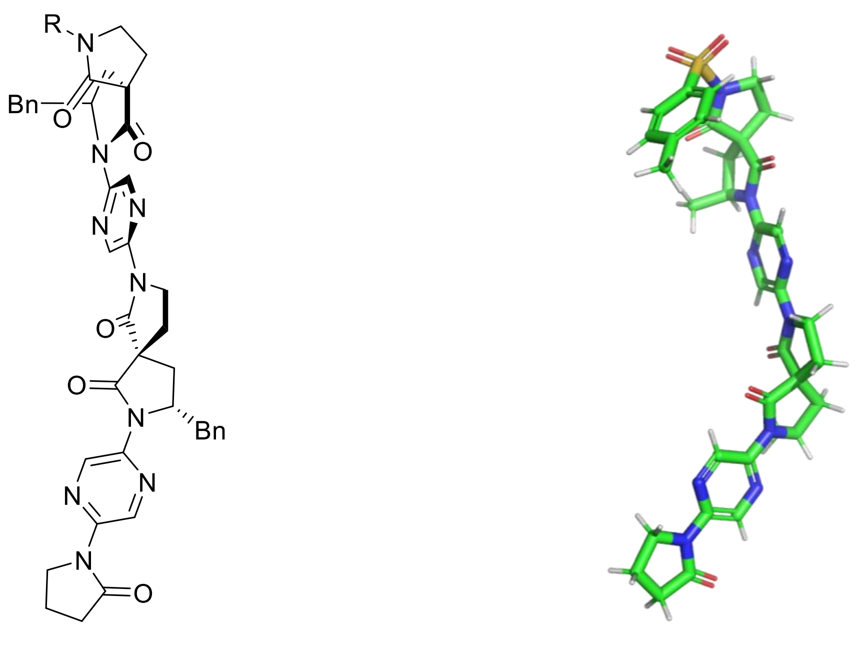

**Figure S20**. Energy-minimised structure of dimer **9** (PM7). Side-chains truncated to $CH_3$ for computational brevity.

#### *2.4.5.1 Trimer 11*

Energy minimum located by the Boltzmann weighted random search approach in 50,000 iterations (**Figure S21**).

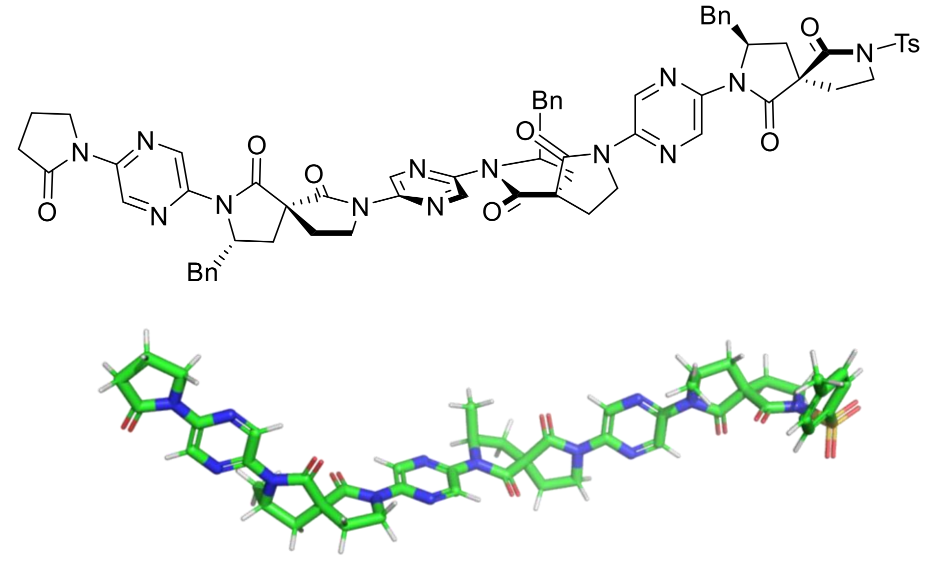

**Figure S21**. Energy-minimised structure of trimer **11** (PM7). Side-chains truncated to $CH_3$ for computational brevity.

### 2.4.6 *pseudo*-Hexamer 13

Energy minimum located by the linear search approach in 336 iterations (14 rotatable bonds, 24 steps per bond, **Figure S22**).

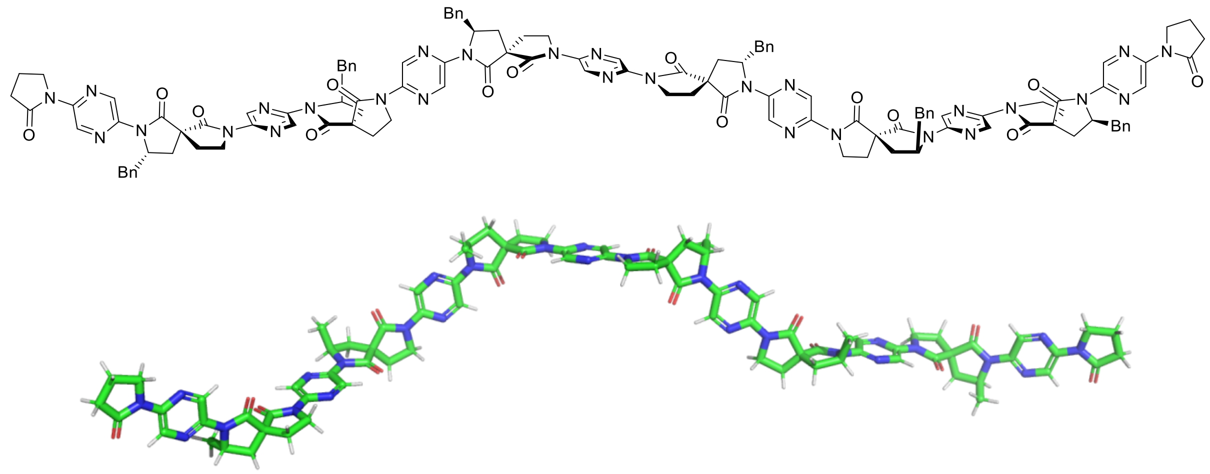

**Figure S22**. Energy-minimised structure of *pseudo*-hexamer **13** (PM7). Side-chains truncated to $CH_3$ for computational brevity.

# 3 Supplementary References


1. Zalesskiy SS, Ananikov VP. Pd<sub>2</sub>(dba)<sub>3</sub>as a Precursor of Soluble Metal Complexes and Nanoparticles: Determination of Palladium Active Species for Catalysis and Synthesis. *Organometallics* 2012, **31**(6)**:** 2302-2309.

2. Armarego WLF, Chai CLL. *Purification of Laboratory Chemicals*, 5th edn. Butterworth-Heinemann, 2003.

3. Cosier J, Glazer AM. A nitrogen-gas-stream cryostat for general X-ray diffraction studies. *J Appl Crystallogr* 1986, **19:** 105-107.

4. CrysAlisPRO. Yarnton, England: Oxford Diffraction/Agilent Technologies UK Ltd.

5. Sheldrick GM. SHELXT - integrated space-group and crystal-structure determination. *Acta Crystallographica A* 2015, **71**(Pt 1)**:** 3-8.

6. Betteridge PW, Carruthers JR, Cooper RI, Prout K, Watkin DJ. CRYSTALS version 12: software for guided crystal structure analysis. *J Appl Crystallogr* 2003, **36:** 1487.

7. Cooper RI, Thompson AL, Watkin DJ. CRYSTALS enhancements: dealing with hydrogen atoms in refinement. *J Appl Crystallogr* 2008, **43:** 1100-1107.

8. Lockhart Z, Knipe PC. Conformationally Programmable Chiral Foldamers with Compact and Extended Domains Controlled by Monomer Structure. *Angew Chem Int Ed* 2018, **57:** 8478-8482.

9. Javorskis T, Orentas E. Chemoselective Deprotection of Sulfonamides Under Acidic Conditions: Scope, Sulfonyl Group Migration, and Synthetic Applications. *J Org Chem* 2017, **82**(24)**:** 13423-13439.

10. Kern N, Felten A-S, Weibel J-M, Pale P, Blanc A. Robust Synthesis of <i>N</i>-Sulfonylazetidine Building Blocks via Ring Contraction of α-Bromo <i>N</i>-Sulfonylpyrrolidinones. *Org Lett* 2014, **16**(23)**:** 6104-6107.

11. Moss TA, Barber DM, Kyle AF, Dixon DJ. Catalytic Asymmetric Alkylation Reactions for the Construction of Protected Ethylene-Amino and Propylene-Amino Motifs Attached to Quaternary Stereocentres. *Chemistry - A European Journal* 2013, **19**(9)**:** 3071-3081.

12. Nelissen J, Nuyts K, Dehaen W, De Borggraeve WM. Synthesis of the orthogonally protected amino alcohol Phaol and analogs. *J Pept Sci* 2011, **17**(7)**:** 527-532.

13. German EA, Ross JE, Knipe PC, Don MF, Thompson S, Hamilton AD. β-Strand Mimetic Foldamers Rigidified through Dipolar Repulsion. *Angew Chem Int Ed* 2015, **54:** 2649-2652.

14. Pedretti A, Villa L, Vistoli G. VEGA -- An open platform to develop chemo-bio-informatics applications, using plug-in architecture and script programming. *J Comput Aided Mol Des* 2004, **18:** 167-173.

15. Stewart JJP. MOPAC 2016. Colorado Springs, CO (USA): Stewart Computational Chemistry; 2016.

16. Stewart JJP. Optimization of parameters for semiempirical methods VI: more modifications to the NDDO approximations and re-optimization of parameters. *J Mol Model* 2013, **19:** 1-32.
