# Supplementary material for: A spirocyclic backbone accesses new conformational space in an extended, dipole-stabilized foldamer
Source: Commun Chem. 2023 Apr 17;6:71. doi: 10.1038/s42004-023-00868-8 (PMC10110530; doi:10.1038/s42004-023-00868-8)
Supplement: Supplementary file 2 — Description of Additional Supplementary Files [file 42004_2023_868_MOESM2_ESM.pdf]

Description of Additional Supplementary Files

File name: Supplementary Data 1

Description: ^{1}H and ^{13}C NMR spectra for all compounds

File name: Supplementary Data 2

Description: Single crystal X-ray data for 7 in cif format.
