# Supplementary material for: A spirocyclic backbone accesses new conformational space in an extended, dipole-stabilized foldamer
Source: Commun Chem. 2023 Apr 17;6:71. doi: 10.1038/s42004-023-00868-8 (PMC10110530; doi:10.1038/s42004-023-00868-8)
Supplement: Supplementary file 3 — Supplementary Data 1 [file 42004_2023_868_MOESM3_ESM.pdf]

Supplementary Information

A spirocyclic backbone accesses new conformational space in an extended, dipole-stabilized foldamer

William E. Roe,[a] Toyah M. C. Warnock [a] and Peter C. Knipe*[a]

[a] *School of Chemistry and Chemical Engineering, Queen's University Belfast, David Keir Building, Belfast, BT9 5AG, UK*

NMR Spectra

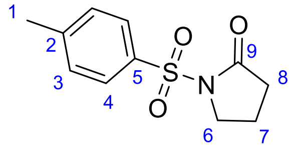

S1

^{1}H NMR

400 MHz

$CDCl_3$

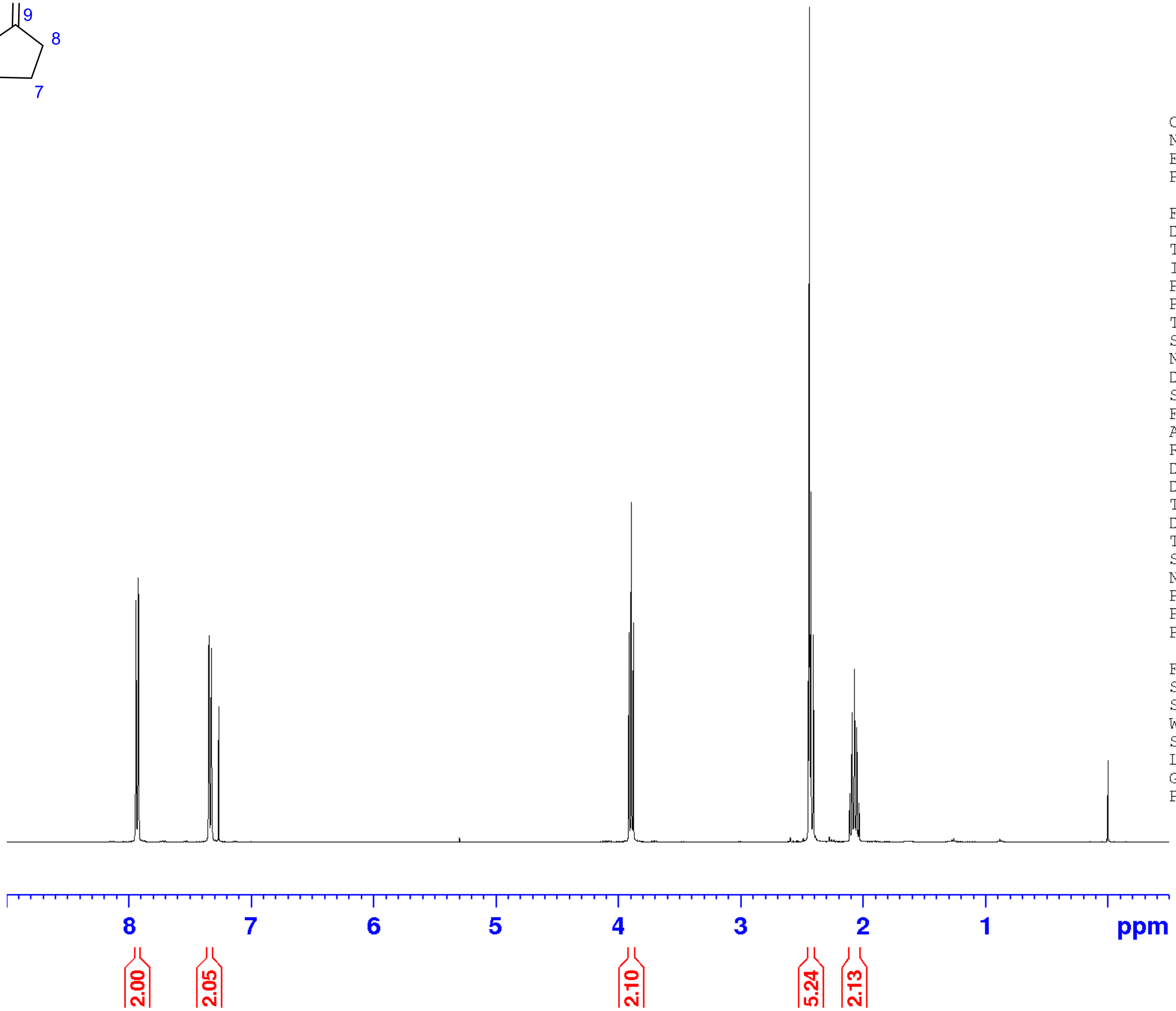

Current Data Parameters
NAME WR 1.32
EXPNO 10
PROCNO 1

F2 - Acquisition Parameters
Date_ 20200225
Time 17.59 h
INSTRUM AVIII_400
PROBHD Z108618_0146 (
PULPROG zg30
TD 65536
SOLVENT CDCl3
NS 16
DS 2
SWH 8223.685 Hz
FIDRES 0.250967 Hz
AQ 3.9845889 sec
RG 128
DW 60.800 usec
DE 17.42 usec
TE 300.0 K
D1 1.00000000 sec
TD0 1
SFO1 400.1124708 MHz
NUC1 1H
P0 5.00 usec
P1 15.00 usec
PLW1 17.29199982 W

F2 - Processing parameters
SI 32768
SF 400.1100062 MHz
WDW EM
SSB 0
LB 0.30 Hz
GB 0
PC 1.00

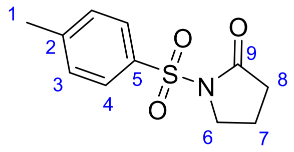

S1

^{13}C NMR

101 MHz

$CDCl_3$

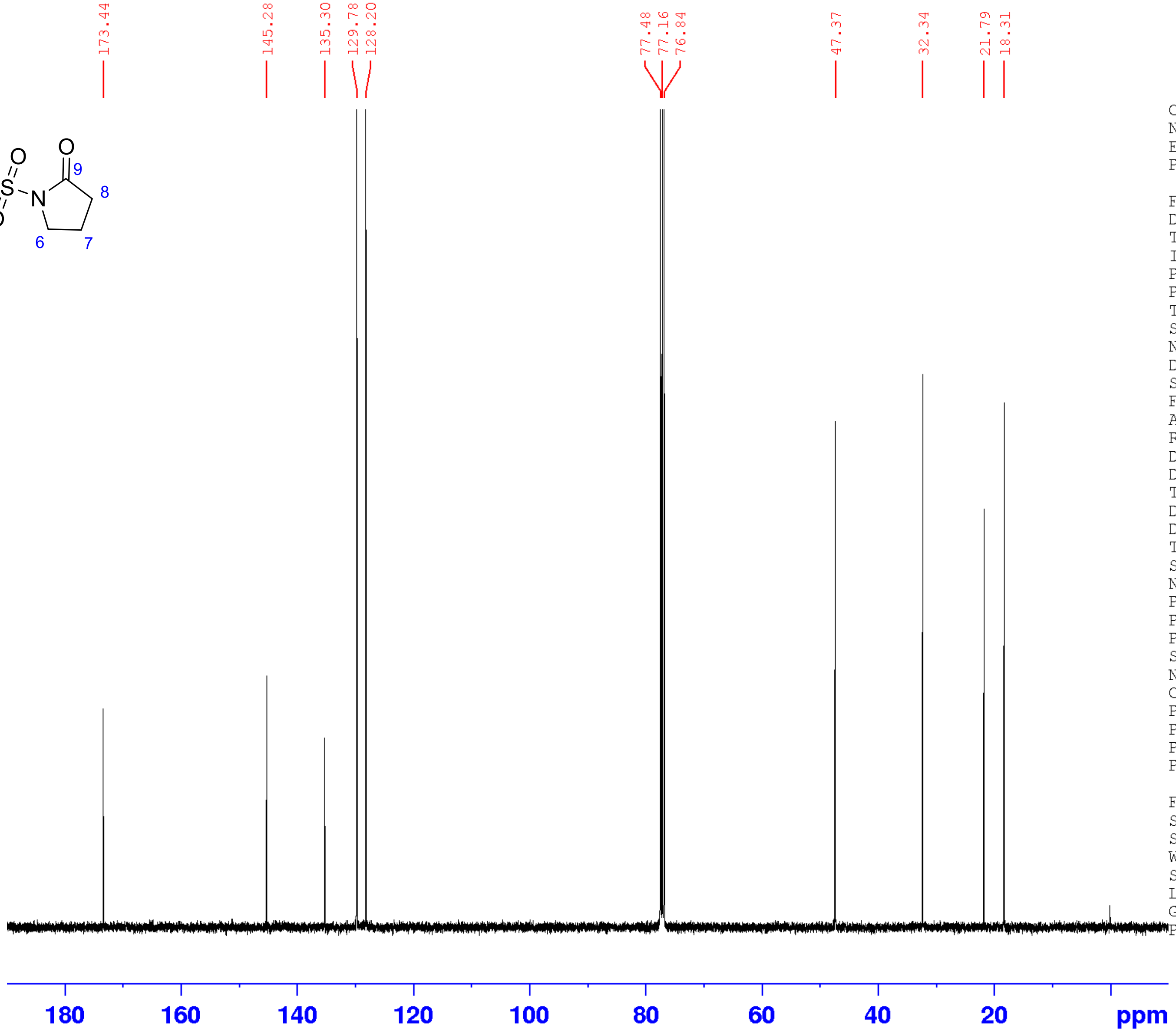


```
Current Data Parameters
NAME                WR 1.32
EXPNO                    11
PROCNO                    1

F2 - Acquisition Parameters
Date_              20200225
Time                  21.41 h
INSTRUM           AVIII_400
PROBHD    Z108618_0146 (
PULPROG             zgpg30
TD                    96150
SOLVENT               CDCl3
NS                     1024
DS                        4
SWH               24038.461 Hz
FIDRES             0.500020 Hz
AQ                1.9999200 sec
RG                     2050
DW                   20.800 usec
DE                     6.50 usec
TE                    300.0 K
D1               1.00000000 sec
D11              0.03000000 sec
TD0                       1
SFO1            100.6178003 MHz
NUC1                    13C
P0                     3.00 usec
P1                     9.00 usec
PLW1            96.68000031 W
SFO2            400.1116004 MHz
NUC2                     1H
CPDPRG[2            waltz64
PCPD2                 90.00 usec
PLW2            17.29199982 W
PLW12            0.48032999 W
PLW13            0.24160001 W

F2 - Processing parameters
SI                   131072
SF              100.6077291 MHz
WDW                      EM
SSB                       0
LB                     1.00 Hz
GB                        0
PC                     1.40
```

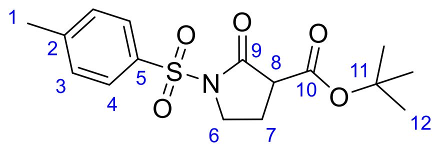

1

^{1}H NMR

400 MHz

$CDCl_3$

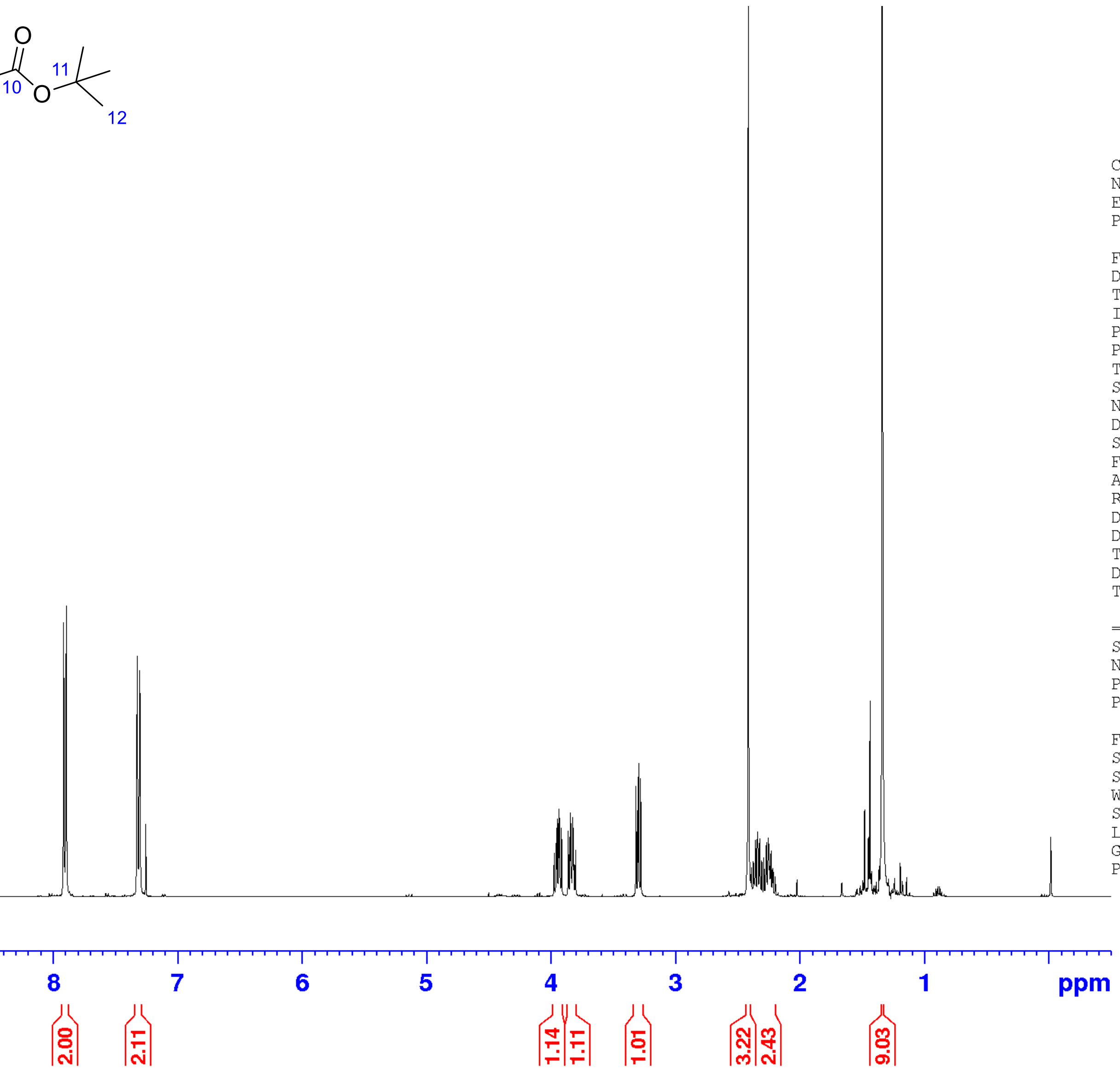


```
Current Data Parameters
NAME              WR 1.33
EXPNO                  10
PROCNO                  1

F2 - Acquisition Parameters
Date_            20200228
Time                10.31
INSTRUM         AVIII_400
PROBHD   5 mm PABBO BB/
PULPROG              zg30
TD                  65536
SOLVENT             CDCl3
NS                     16
DS                      2
SWH              8223.685 Hz
FIDRES           0.125483 Hz
AQ              3.9845889 sec
RG                     36
DW                 60.800 usec
DE                   6.50 usec
TE                  298.0 K
D1            1.00000000 sec
TD0                     1

======== CHANNEL f1 ========
SFO1          399.9124696 MHz
NUC1                   1H
P1                  15.00 usec
PLW1          17.29199982 W

F2 - Processing parameters
SI                  32768
SF            399.9100094 MHz
WDW                    EM
SSB                     0
LB                   0.30 Hz
GB                      0
PC                   1.00
```

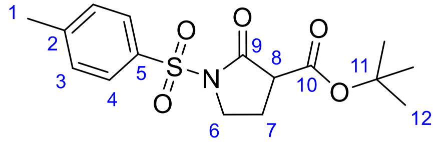

1

^{13}C NMR

101 MHz

$CDCl_3$

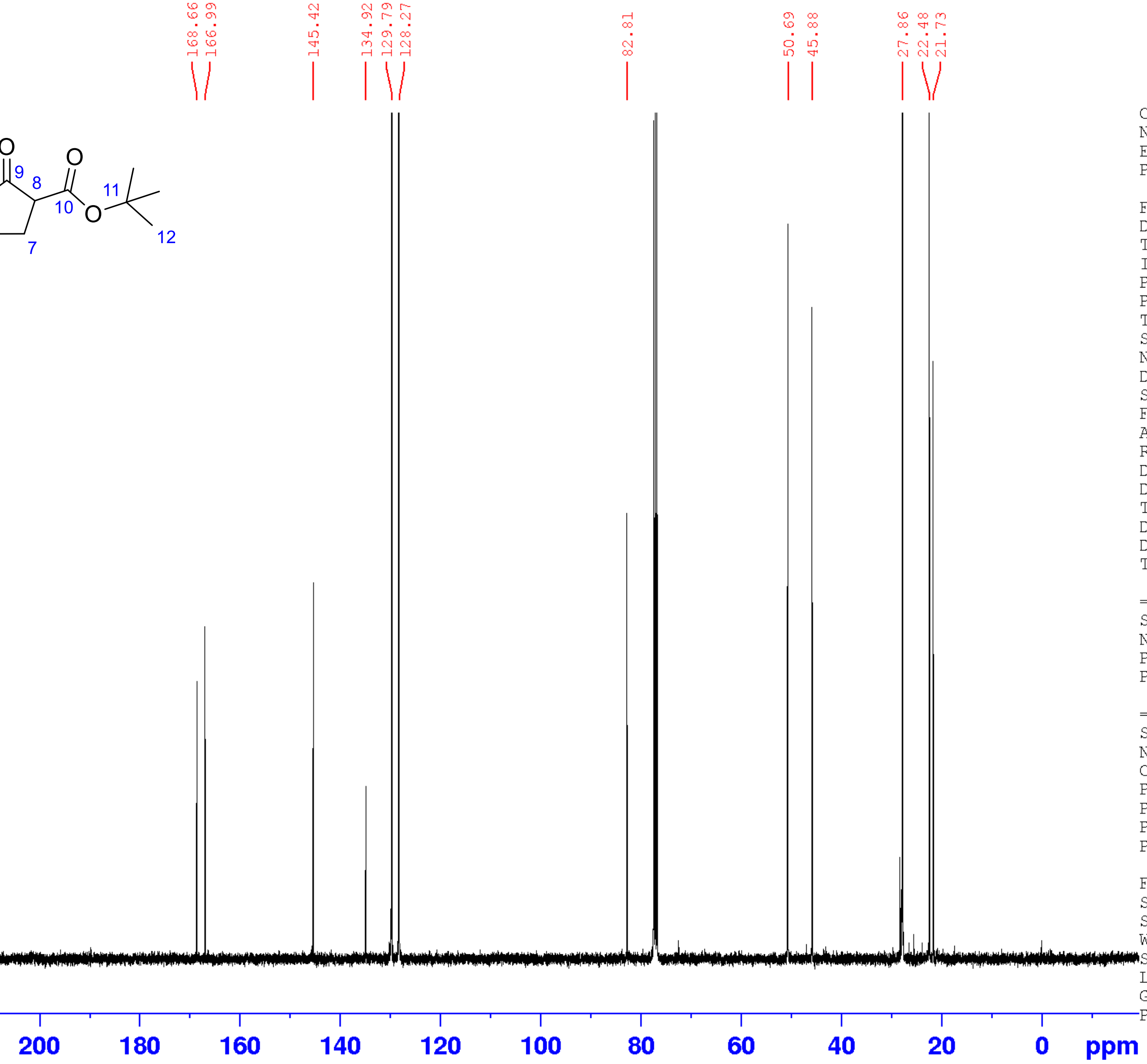


```
Current Data Parameters
NAME              WR 1.33
EXPNO                  11
PROCNO                  1

F2 - Acquisition Parameters
Date_            20200228
Time                20.03
INSTRUM         AVIII_400
PROBHD     5 mm PABBO BB/
PULPROG           zgpg30
TD                 96150
SOLVENT            CDCl3
NS                  1024
DS                     4
SWH            24038.461 Hz
FIDRES          0.250010 Hz
AQ             1.9999200 sec
RG                   161
DW                20.800 usec
DE                  6.50 usec
TE                 301.5 K
D1            1.00000000 sec
D11           0.03000000 sec
TD0                    1

======== CHANNEL f1 ========
SFO1         100.5675047 MHz
NUC1                 13C
P1                  9.00 usec
PLW1         96.68000031 W

======== CHANNEL f2 ========
SFO2         399.9115996 MHz
NUC2                  1H
CPDPRG[2         waltz64
PCPD2              90.00 usec
PLW2         17.29199982 W
PLW12         0.48032999 W
PLW13         0.38907000 W

F2 - Processing parameters
SI                131072
SF           100.5574388 MHz
WDW                   EM
SSB                    0
LB                  1.00 Hz
GB                     0
PC                  1.40
```

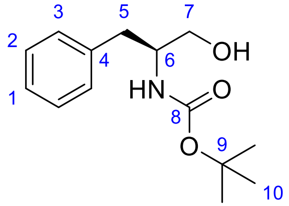

S2

^{1}H NMR

400 MHz

$CDCl_3$

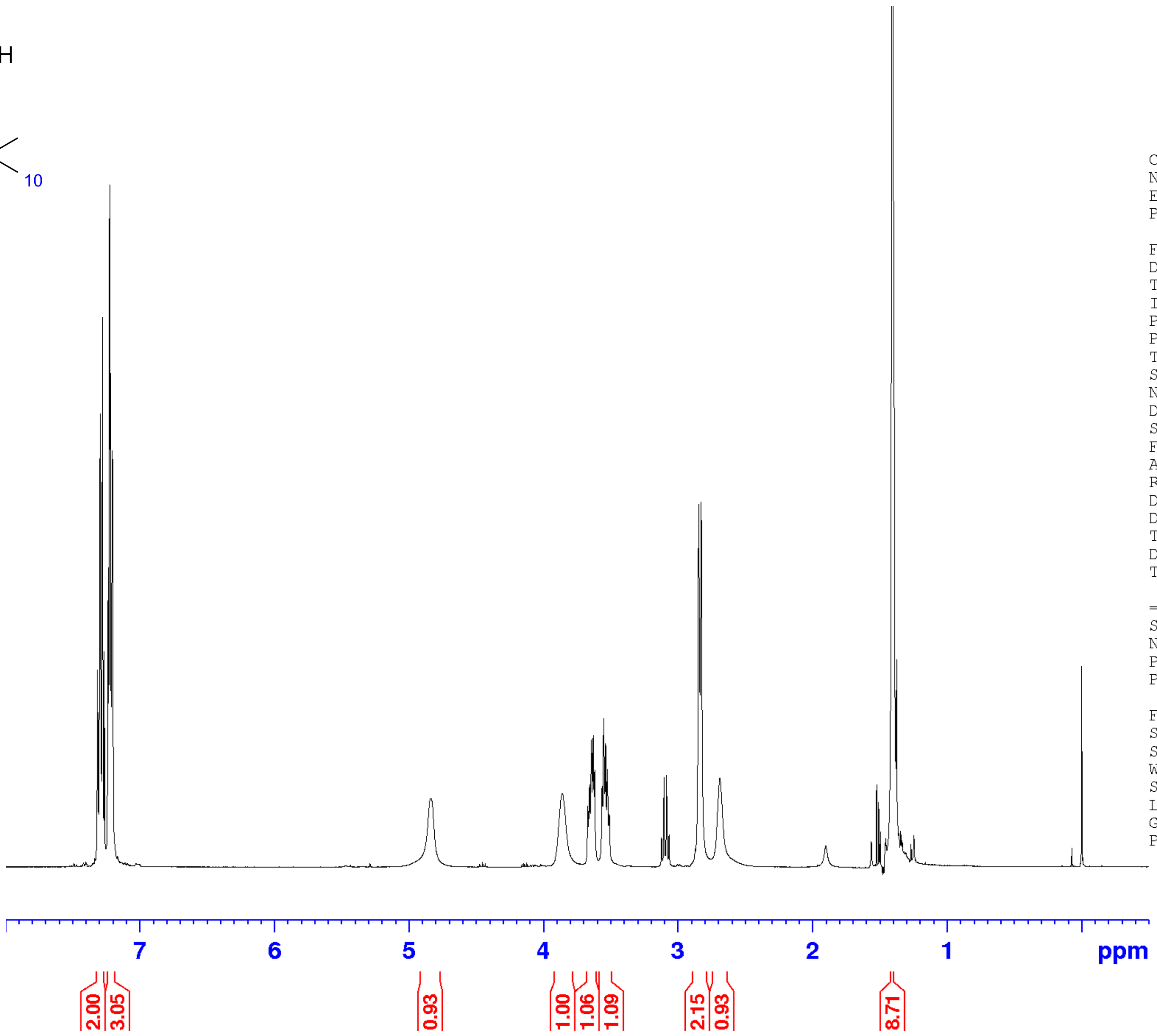


```
Current Data Parameters
NAME              WR 1.28
EXPNO                  10
PROCNO                  1

F2 - Acquisition Parameters
Date_            20200221
Time                 9.28
INSTRUM         AVIII_400
PROBHD   5 mm PABBO BB/
PULPROG              zg30
TD                  65536
SOLVENT             CDCl3
NS                     16
DS                      2
SWH              8223.685 Hz
FIDRES           0.125483 Hz
AQ              3.9845889 sec
RG                     36
DW                 60.800 usec
DE                   6.50 usec
TE                  297.0 K
D1             1.00000000 sec
TD0                     1

======== CHANNEL f1 ========
SFO1          399.9124696 MHz
NUC1                   1H
P1                  15.00 usec
PLW1          17.29199982 W

F2 - Processing parameters
SI                  32768
SF            399.9100084 MHz
WDW                    EM
SSB                     0
LB                   0.30 Hz
GB                      0
PC                   1.00
```

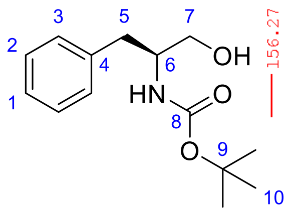

S2

^{13}C NMR

101 MHz

$CDCl_3$

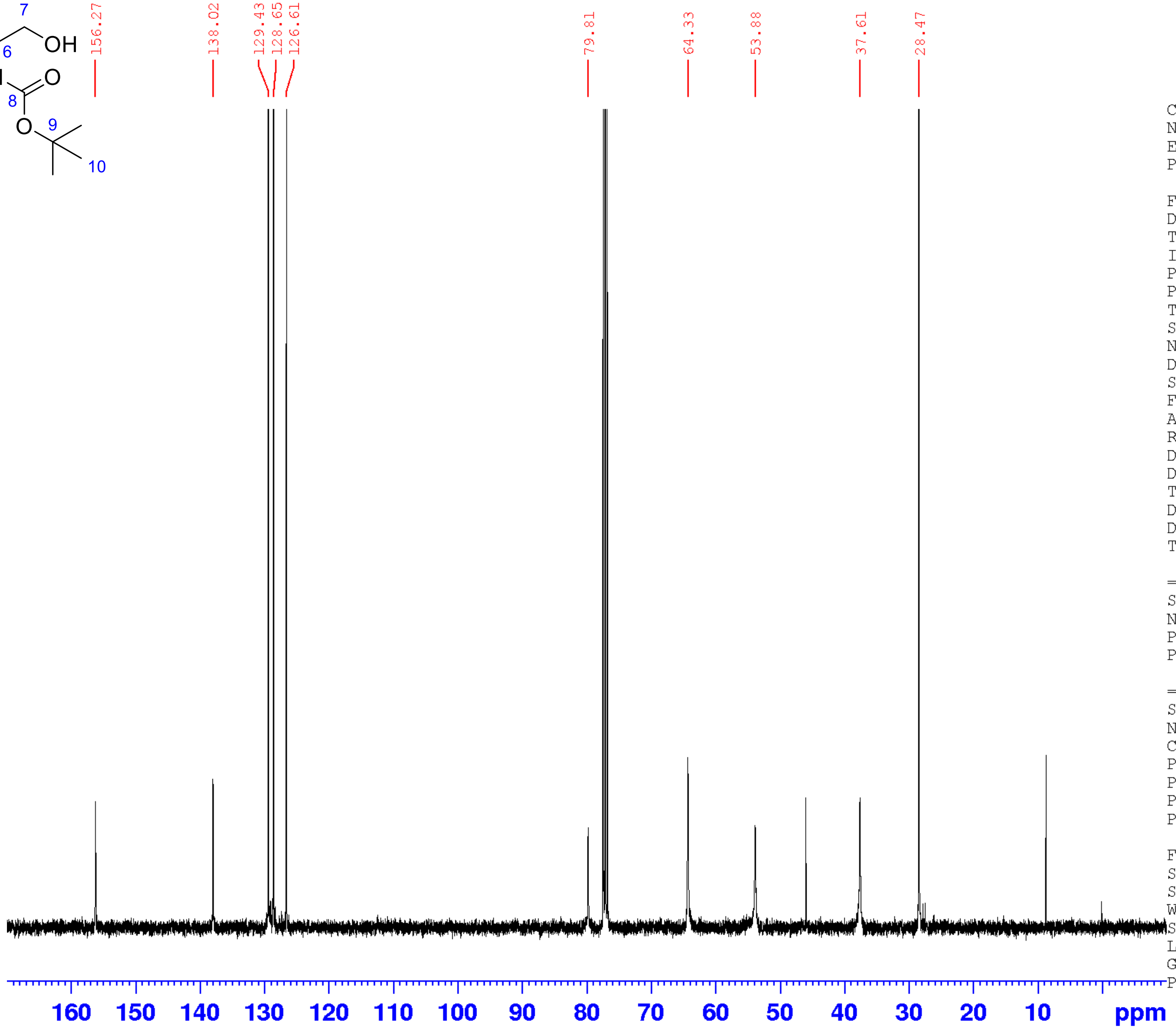


```
Current Data Parameters
NAME               WR 1.28
EXPNO                   11
PROCNO                   1

F2 - Acquisition Parameters
Date_             20200221
Time                 19.58
INSTRUM          AVIII_400
PROBHD     5 mm PABBO BB/
PULPROG            zgpg30
TD                   96150
SOLVENT              CDCl3
NS                    1024
DS                       4
SWH              24038.461 Hz
FIDRES            0.250010 Hz
AQ               1.9999200 sec
RG                     181
DW                  20.800 usec
DE                    6.50 usec
TE                   301.0 K
D1              1.00000000 sec
D11             0.03000000 sec
TD0                      1

======== CHANNEL f1 ========
SFO1           100.5675047 MHz
NUC1                   13C
P1                    9.00 usec
PLW1           96.68000031 W

======== CHANNEL f2 ========
SFO2           399.9115996 MHz
NUC2                    1H
CPDPRG[2           waltz64
PCPD2                90.00 usec
PLW2           17.29199982 W
PLW12           0.48032999 W
PLW13           0.38907000 W

F2 - Processing parameters
SI                  131072
SF             100.5574377 MHz
WDW                     EM
SSB                      0
LB                    1.00 Hz
GB                       0
PC                    1.40
```

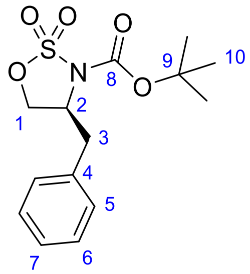

2

^{1}H NMR

400 MHz

$CDCl_3$

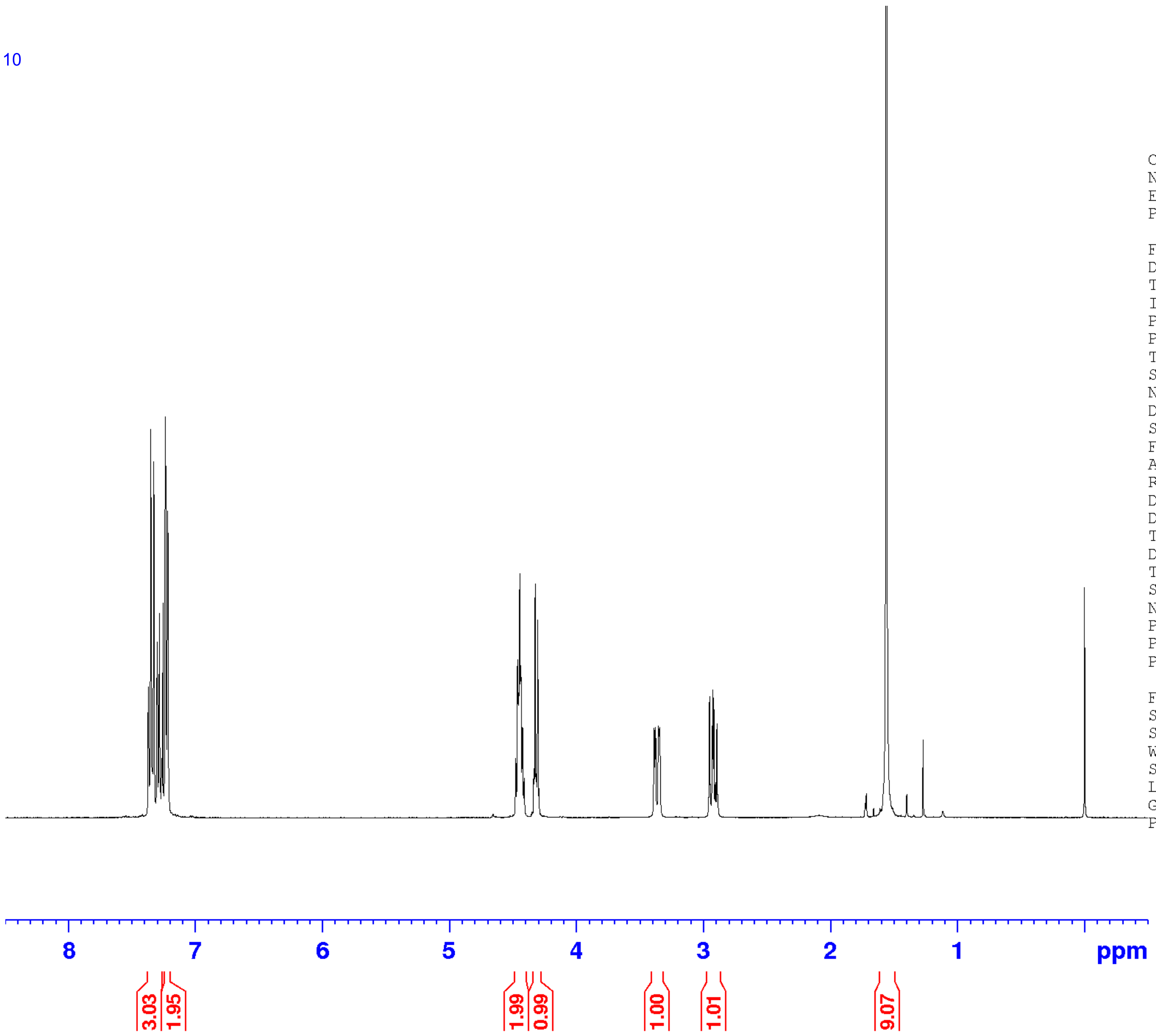


```
Current Data Parameters
NAME              WR 2.213
EXPNO                   10
PROCNO                   1

F2 - Acquisition Parameters
Date_             20220506
Time                 16.29 h
INSTRUM          AVIII_400
PROBHD      Z108618_0146 (
PULPROG               zg30
TD                   65536
SOLVENT              CDCl3
NS                      16
DS                       2
SWH               8223.685 Hz
FIDRES            0.250967 Hz
AQ               3.9845889 sec
RG                     128
DW                  60.800 usec
DE                   17.42 usec
TE                   300.0 K
D1              1.00000000 sec
TD0                      1
SFO1           400.1124708 MHz
NUC1                    1H
P0                    5.00 usec
P1                   15.00 usec
PLW1           17.29199982 W

F2 - Processing parameters
SI                   32768
SF             400.1100100 MHz
WDW                     EM
SSB                      0
LB                    0.30 Hz
GB                       0
PC                    1.00
```

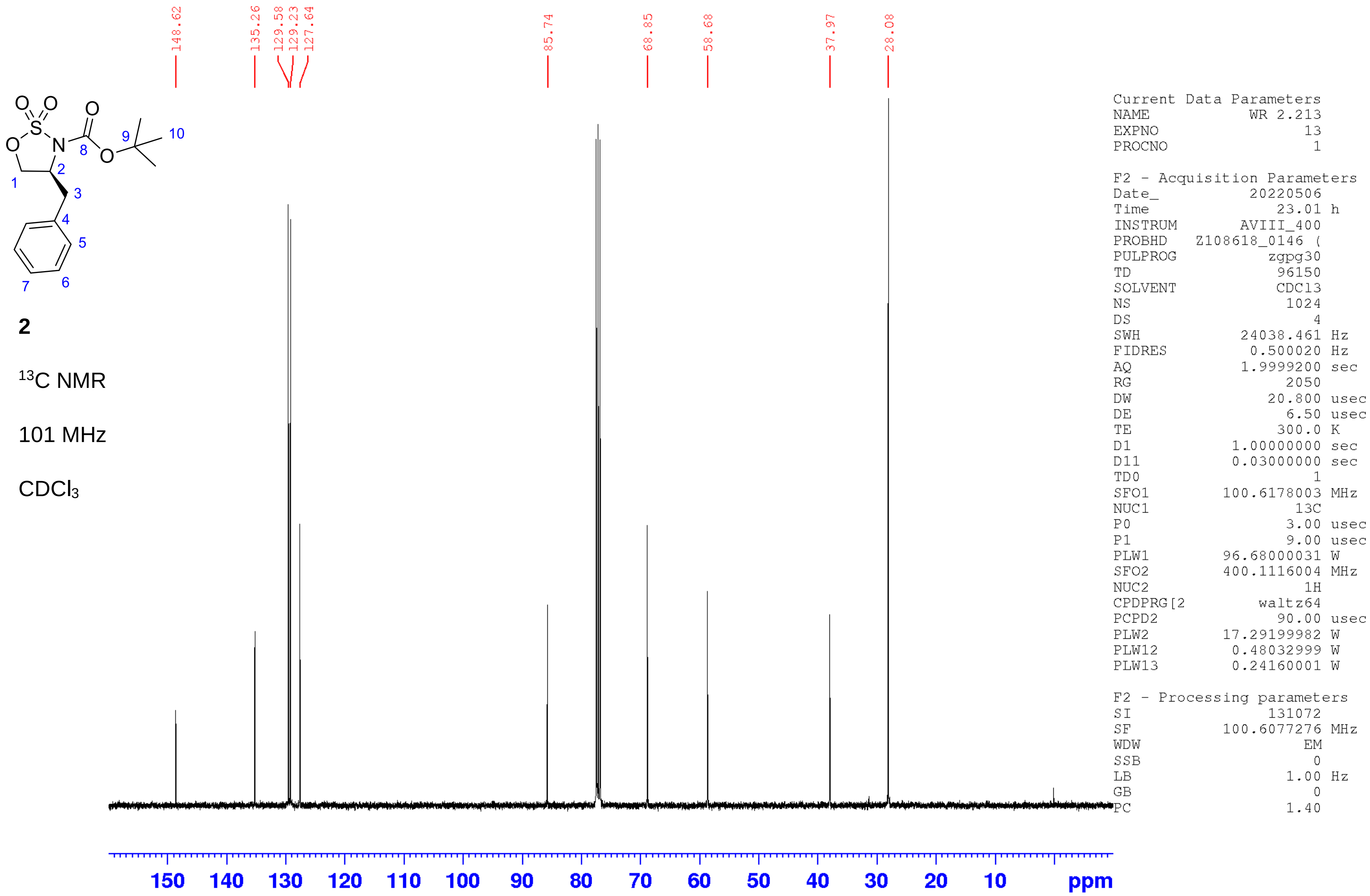

148.62
135.26
129.58
129.23
127.64
85.74
68.85
58.68
37.97
28.08
2
^{13}C NMR
101 MHz
$CDCl_3$
Current Data Parameters
NAME WR 2.213
EXPNO 13
PROCNO 1
F2 - Acquisition Parameters
Date_ 20220506
Time 23.01 h
INSTRUM AVIII_400
PROBHD Z108618_0146 (
PULPROG zgpg30
TD 96150
SOLVENT CDCl3
NS 1024
DS 4
SWH 24038.461 Hz
FIDRES 0.500020 Hz
AQ 1.9999200 sec
RG 2050
DW 20.800 usec
DE 6.50 usec
TE 300.0 K
D1 1.00000000 sec
D11 0.03000000 sec
TD0 1
SFO1 100.6178003 MHz
NUC1 13C
P0 3.00 usec
P1 9.00 usec
PLW1 96.68000031 W
SFO2 400.1116004 MHz
NUC2 1H
CPDPRG[2 waltz64
PCPD2 90.00 usec
PLW2 17.29199982 W
PLW12 0.48032999 W
PLW13 0.24160001 W
F2 - Processing parameters
SI 131072
SF 100.6077276 MHz
WDW EM
SSB 0
LB 1.00 Hz
GB 0
PC 1.40
150 140 130 120 110 100 90 80 70 60 50 40 30 20 10 ppm

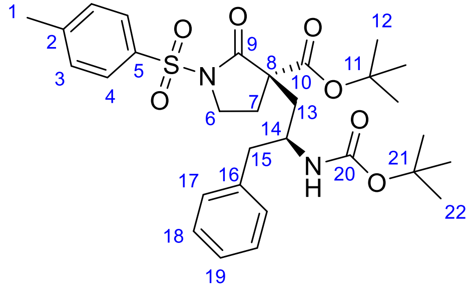

3

Major diastereomer

^{1}H NMR

400 MHz

$CDCl_3$

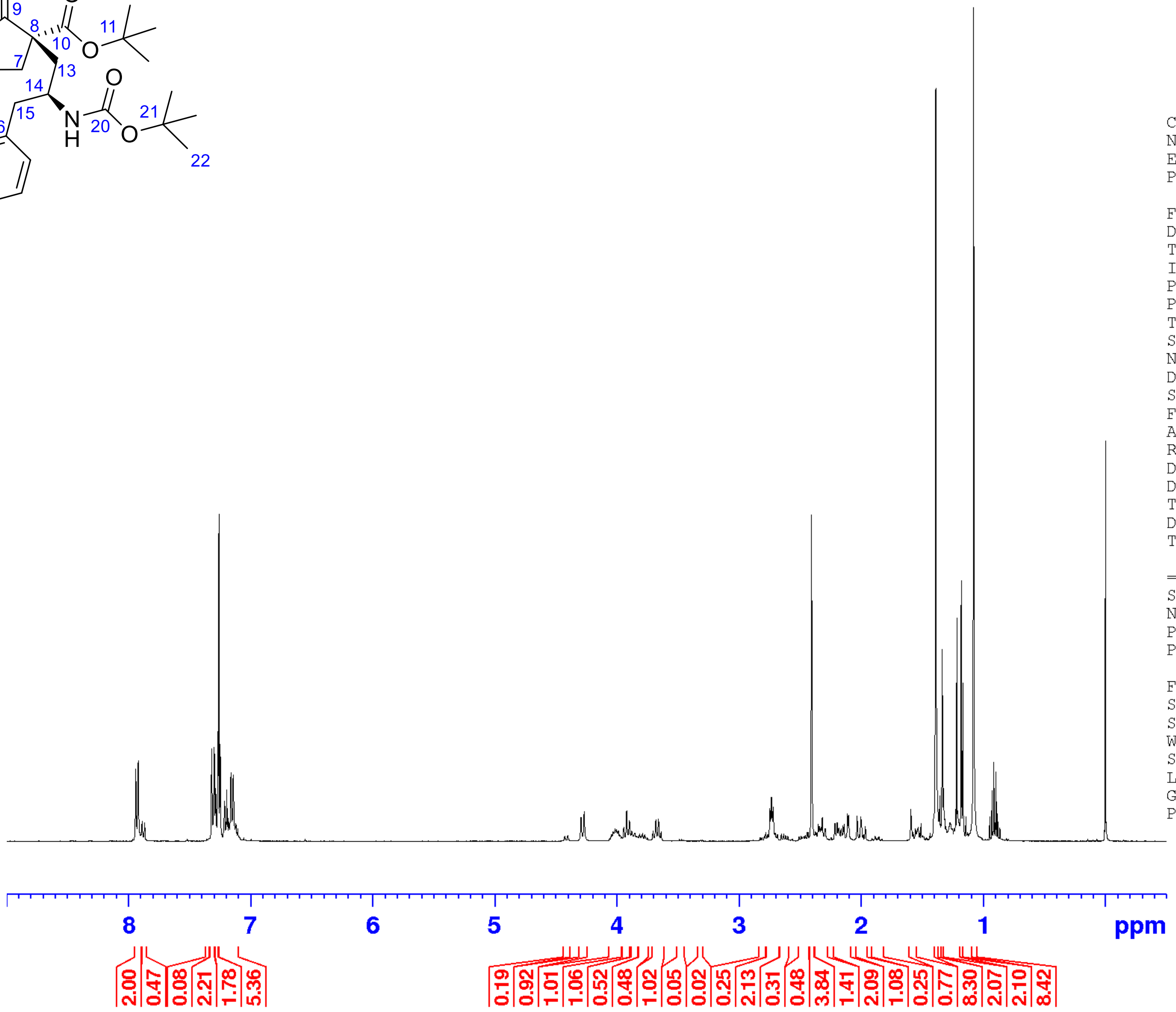

Current Data Parameters
NAME WR 1.74
EXPNO 10
PROCNO 1

F2 - Acquisition Parameters
Date_ 20201214
Time 14.06
INSTRUM AVIII_400
PROBHD 5 mm PABBO BB/
PULPROG zg30
TD 65536
SOLVENT CDCl3
NS 16
DS 2
SWH 8223.685 Hz
FIDRES 0.125483 Hz
AQ 3.9845889 sec
RG 80.6
DW 60.800 usec
DE 6.50 usec
TE 293.3 K
D1 1.00000000 sec
TD0 1

======== CHANNEL f1 ========
SFO1 399.9124696 MHz
NUC1 1H
P1 15.00 usec
PLW1 17.29199982 W

F2 - Processing parameters
SI 32768
SF 399.9100084 MHz
WDW EM
SSB 0
LB 0.30 Hz
GB 0
PC 1.00

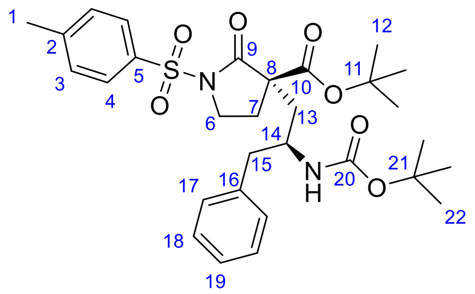

3

Peaks pick for Minor diastereomer

^{1}H NMR

400 MHz

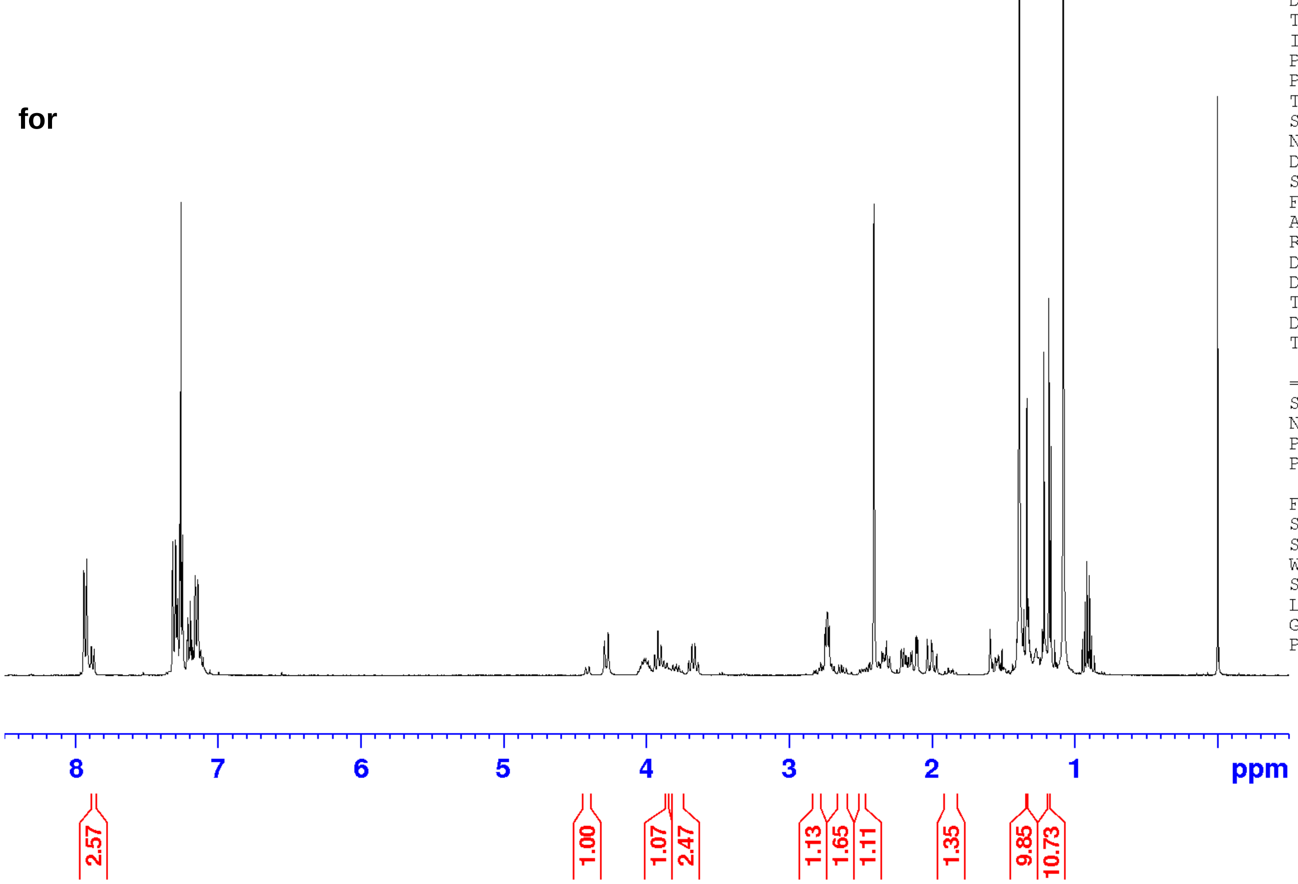


```
Current Data Parameters
NAME              WR 1.74
EXPNO                  15
PROCNO                  1

F2 - Acquisition Parameters
Date_            20201214
Time                14.06
INSTRUM         AVIII_400
PROBHD    5 mm PABBO BB/
PULPROG              zg30
TD                  65536
SOLVENT             CDCl3
NS                     16
DS                      2
SWH              8223.685 Hz
FIDRES           0.125483 Hz
AQ              3.9845889 sec
RG                   80.6
DW                 60.800 usec
DE                   6.50 usec
TE                  293.3 K
D1             1.00000000 sec
TD0                     1

======== CHANNEL f1 ========
SFO1          399.9124696 MHz
NUC1                   1H
P1                  15.00 usec
PLW1          17.29199982 W

F2 - Processing parameters
SI                  32768
SF            399.9100084 MHz
WDW                    EM
SSB                     0
LB                   0.30 Hz
GB                      0
PC                   1.00
```

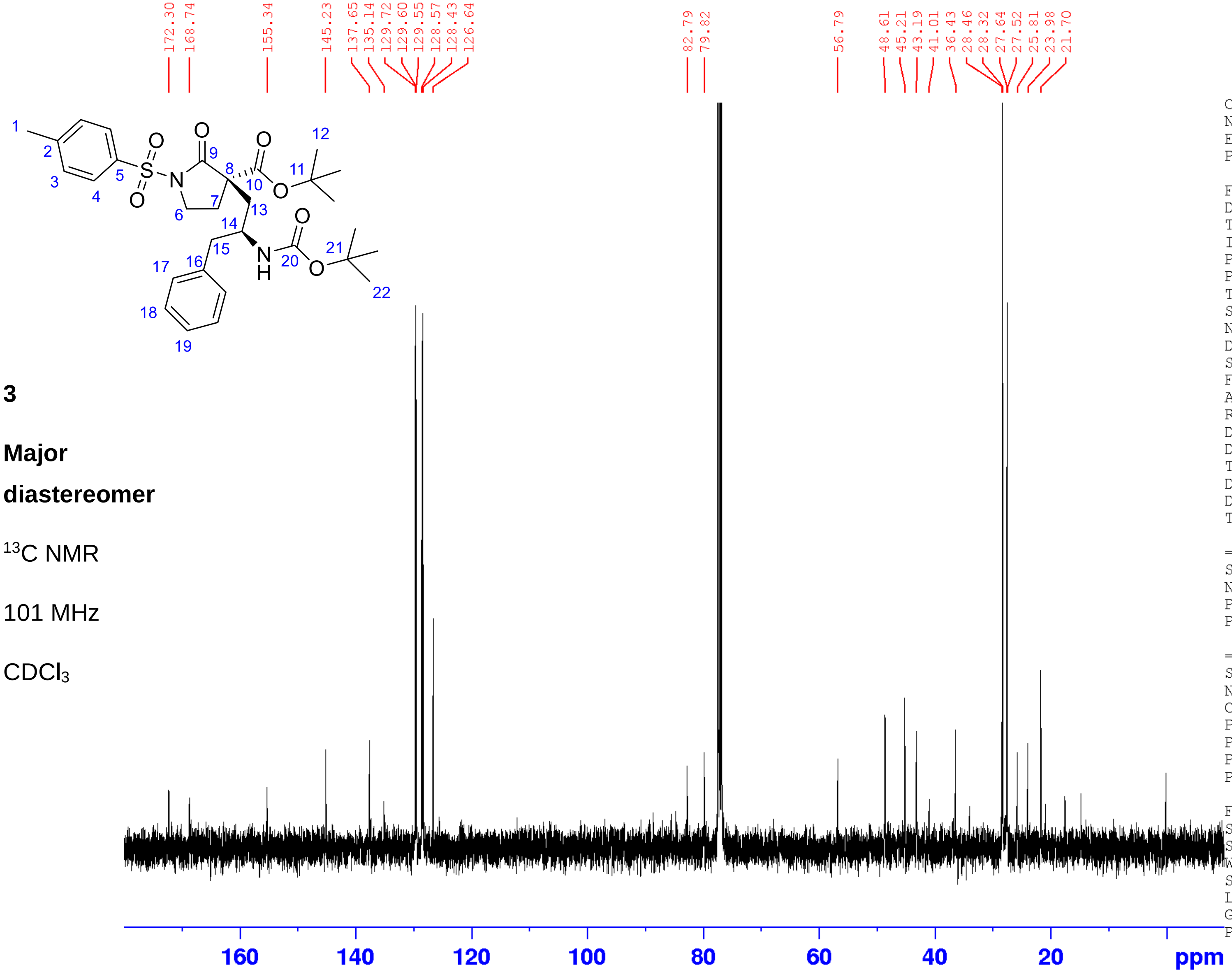

3

Major diastereomer

^{13}C NMR

101 MHz

$CDCl_3$


```
Current Data Parameters
NAME              WR 1.74
EXPNO                  11
PROCNO                  1

F2 - Acquisition Parameters
Date_            20201214
Time                15.31
INSTRUM          AVIII_400
PROBHD    5 mm PABBO BB/
PULPROG            zgpg30
TD                  96150
SOLVENT             CDCl3
NS                   1024
DS                      4
SWH             24038.461 Hz
FIDRES           0.250010 Hz
AQ              1.9999200 sec
RG                    144
DW                 20.800 usec
DE                   6.50 usec
TE                  299.5 K
D1             1.00000000 sec
D11            0.03000000 sec
TD0                     1

======== CHANNEL f1 ========
SFO1         100.5675047 MHz
NUC1                  13C
P1                   9.00 usec
PLW1          96.68000031 W

======== CHANNEL f2 ========
SFO2         399.9115996 MHz
NUC2                   1H
CPDPRG[2          waltz64
PCPD2               90.00 usec
PLW2          17.29199982 W
PLW12          0.48032999 W
PLW13          0.38907000 W

F2 - Processing parameters
SI                 131072
SF            100.5574345 MHz
WDW                    EM
SSB                     0
LB                   1.00 Hz
GB                      0
PC                   1.40
```

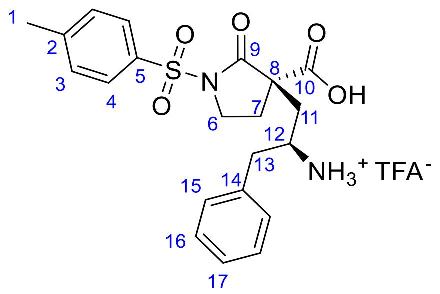

4

Major diastereomer

^{1}H NMR

400 MHz

methanol-d4

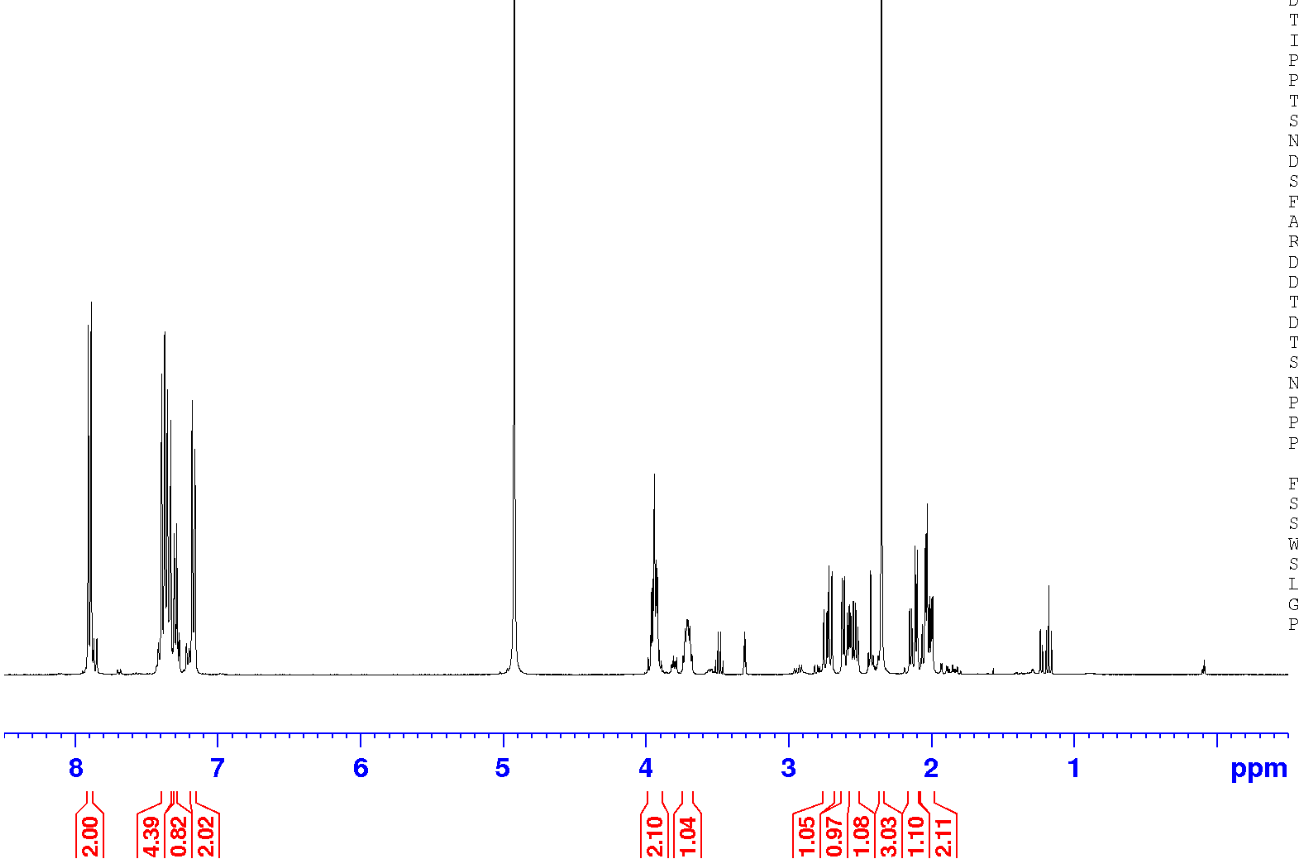


```
Current Data Parameters
NAME                WR 1.64
EXPNO                    10
PROCNO                    1

F2 - Acquisition Parameters
Date_              20201002
Time                  16.09 h
INSTRUM           AVIII_400
PROBHD    Z108618_0146 (
PULPROG                zg30
TD                    65536
SOLVENT                MeOD
NS                       16
DS                        2
SWH                8223.685 Hz
FIDRES             0.250967 Hz
AQ                3.9845889 sec
RG                      128
DW                   60.800 usec
DE                    17.42 usec
TE                    300.0 K
D1               1.00000000 sec
TD0                       1
SFO1            400.1124708 MHz
NUC1                     1H
P0                     5.00 usec
P1                    15.00 usec
PLW1            17.29199982 W

F2 - Processing parameters
SI                    32768
SF              400.1100075 MHz
WDW                      EM
SSB                       0
LB                     0.30 Hz
GB                        0
PC                     1.00
```

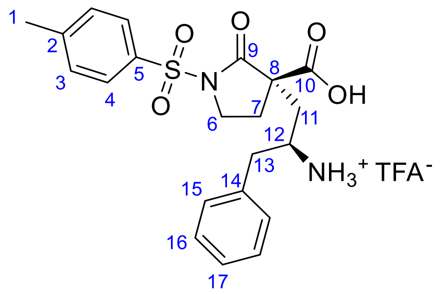

4

Peaks picked for Minor diastereomer

^{1}H NMR

400 MHz

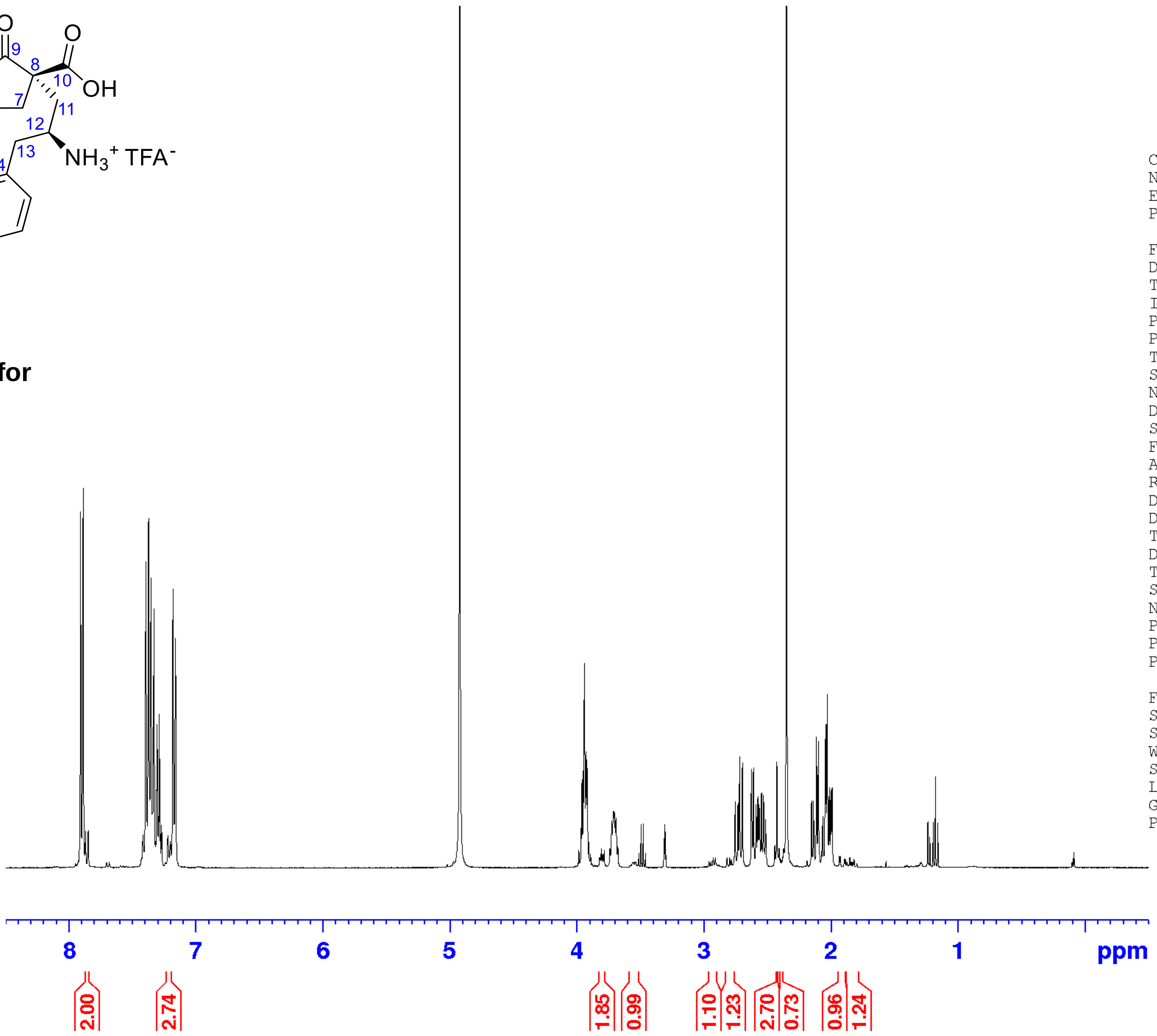

Current Data Parameters
NAME WR 1.64
EXPNO 20
PROCNO 1

F2 - Acquisition Parameters
Date_ 20201002
Time 16.09 h
INSTRUM AVIII_400
PROBHD Z108618_0146 (
PULPROG zg30
TD 65536
SOLVENT MeOD
NS 16
DS 2
SWH 8223.685 Hz
FIDRES 0.250967 Hz
AQ 3.9845889 sec
RG 128
DW 60.800 usec
DE 17.42 usec
TE 300.0 K
D1 1.00000000 sec
TD0 1
SFO1 400.1124708 MHz
NUC1 1H
P0 5.00 usec
P1 15.00 usec
PLW1 17.29199982 W

F2 - Processing parameters
SI 32768
SF 400.1100075 MHz
WDW EM
SSB 0
LB 0.30 Hz
GB 0
PC 1.00

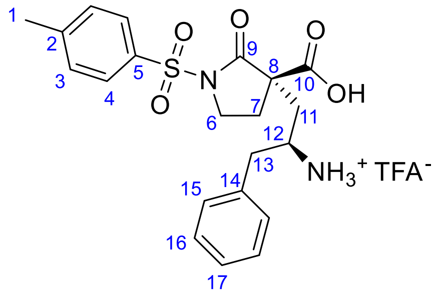

4

^{13}C NMR

101 MHz

methanol-d4

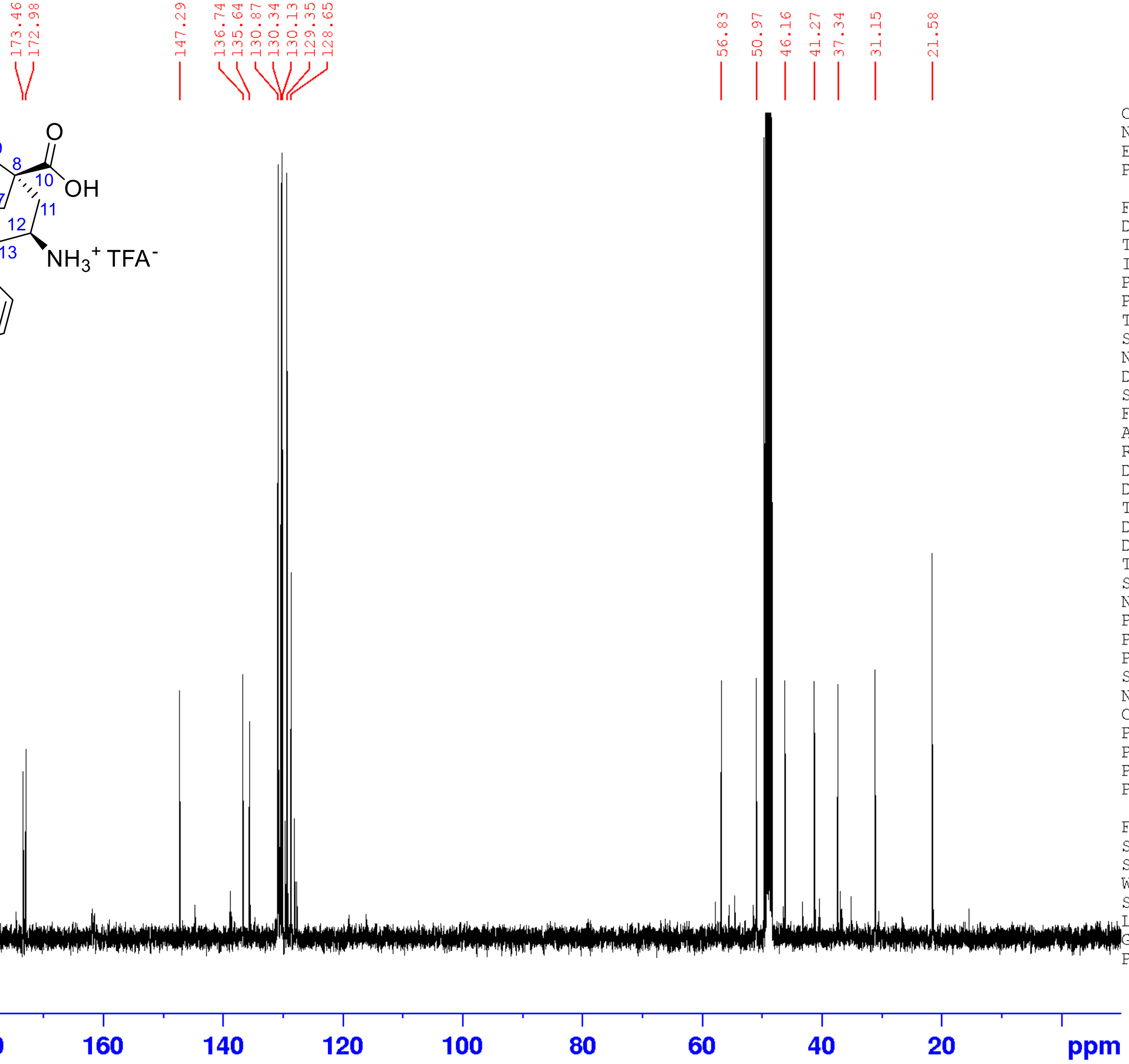

Current Data Parameters
NAME WR 1.64
EXPNO 11
PROCNO 1

F2 - Acquisition Parameters
Date_ 20201002
Time 18.41 h
INSTRUM AVIII_400
PROBHD Z108618_0146 (
PULPROG zgpg30
TD 96150
SOLVENT MeOD
NS 1024
DS 4
SWH 24038.461 Hz
FIDRES 0.500020 Hz
AQ 1.9999200 sec
RG 2050
DW 20.800 usec
DE 6.50 usec
TE 300.0 K
D1 1.00000000 sec
D11 0.03000000 sec
TD0 1
SFO1 100.6178003 MHz
NUC1 13C
P0 3.00 usec
P1 9.00 usec
PLW1 96.68000031 W
SFO2 400.1116004 MHz
NUC2 1H
CPDPRG[2 waltz64
PCPD2 90.00 usec
PLW2 17.29199982 W
PLW12 0.48032999 W
PLW13 0.24160001 W

F2 - Processing parameters
SI 131072
SF 100.6075996 MHz
WDW EM
SSB 0
LB 1.00 Hz
GB 0
PC 1.40

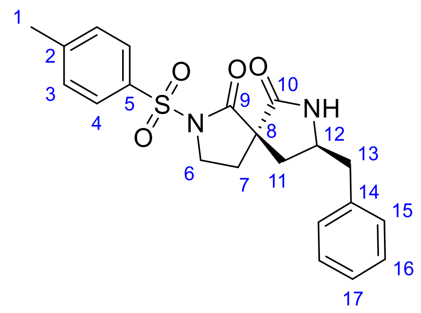

5

Major diastereomer

^{1}H NMR

600 MHz

$CDCl_3$

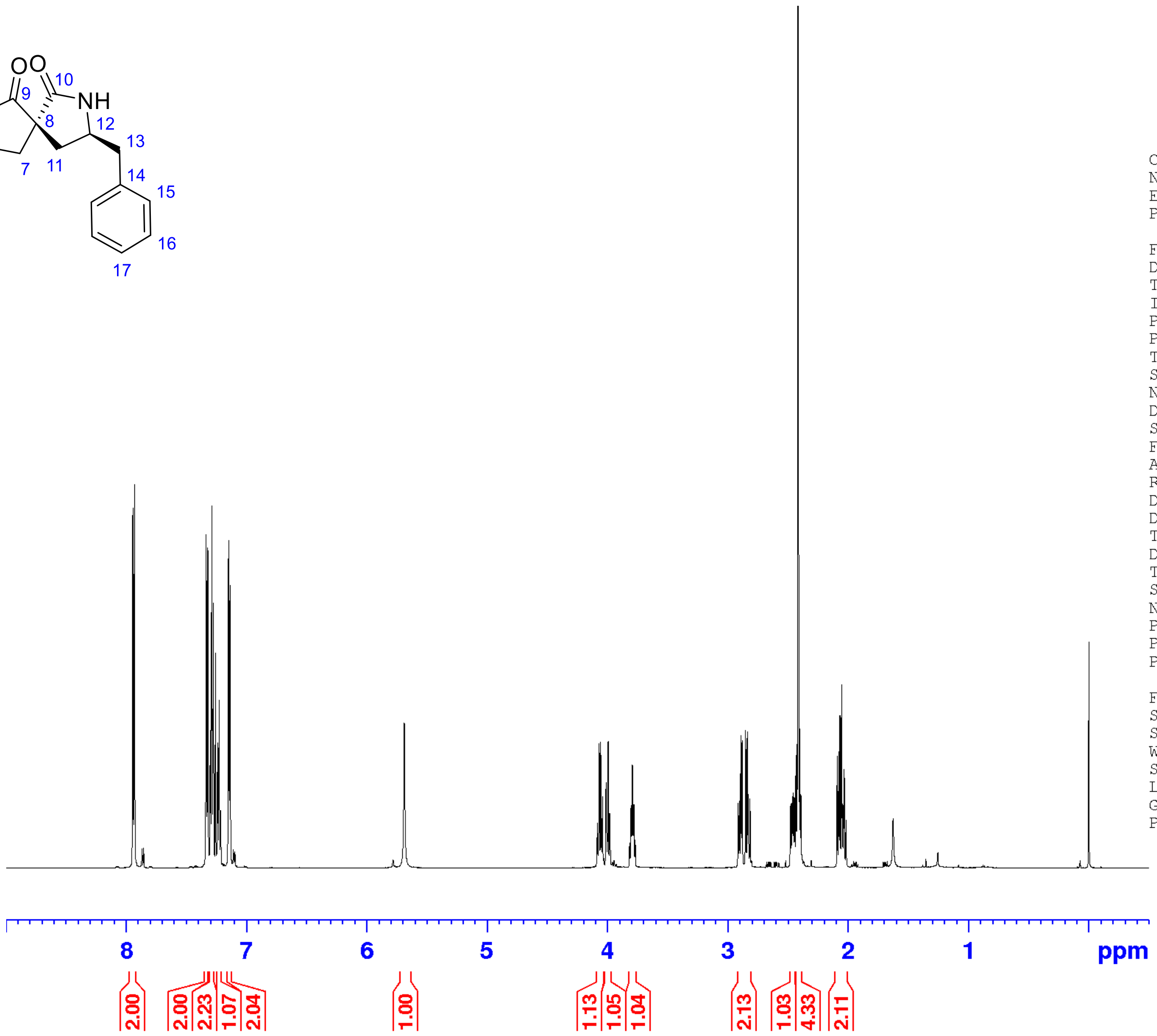


```
Current Data Parameters
NAME       WR 1.121 (600)
EXPNO                  10
PROCNO                  1

F2 - Acquisition Parameters
Date_            20220318
Time                17.51 h
INSTRUM             spect
PROBHD     Z114607_0188 (
PULPROG              zg30
TD                  65536
SOLVENT             CDCl3
NS                     16
DS                      2
SWH             12019.230 Hz
FIDRES           0.366798 Hz
AQ              2.7262976 sec
RG                  74.91
DW                 41.600 usec
DE                  12.10 usec
TE                  300.0 K
D1             1.00000000 sec
TD0                     1
SFO1          600.1337058 MHz
NUC1                   1H
P0                   3.33 usec
P1                  10.00 usec
PLW1          26.60000038 W

F2 - Processing parameters
SI                  65536
SF            600.1300145 MHz
WDW                    EM
SSB                     0
LB                   0.30 Hz
GB                      0
PC                   1.00
```

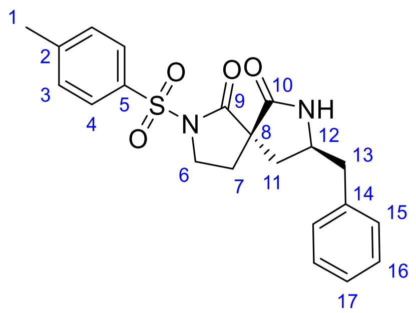

5

Peaks pick for Minor diastereomer

^{1}H NMR

600 MHz

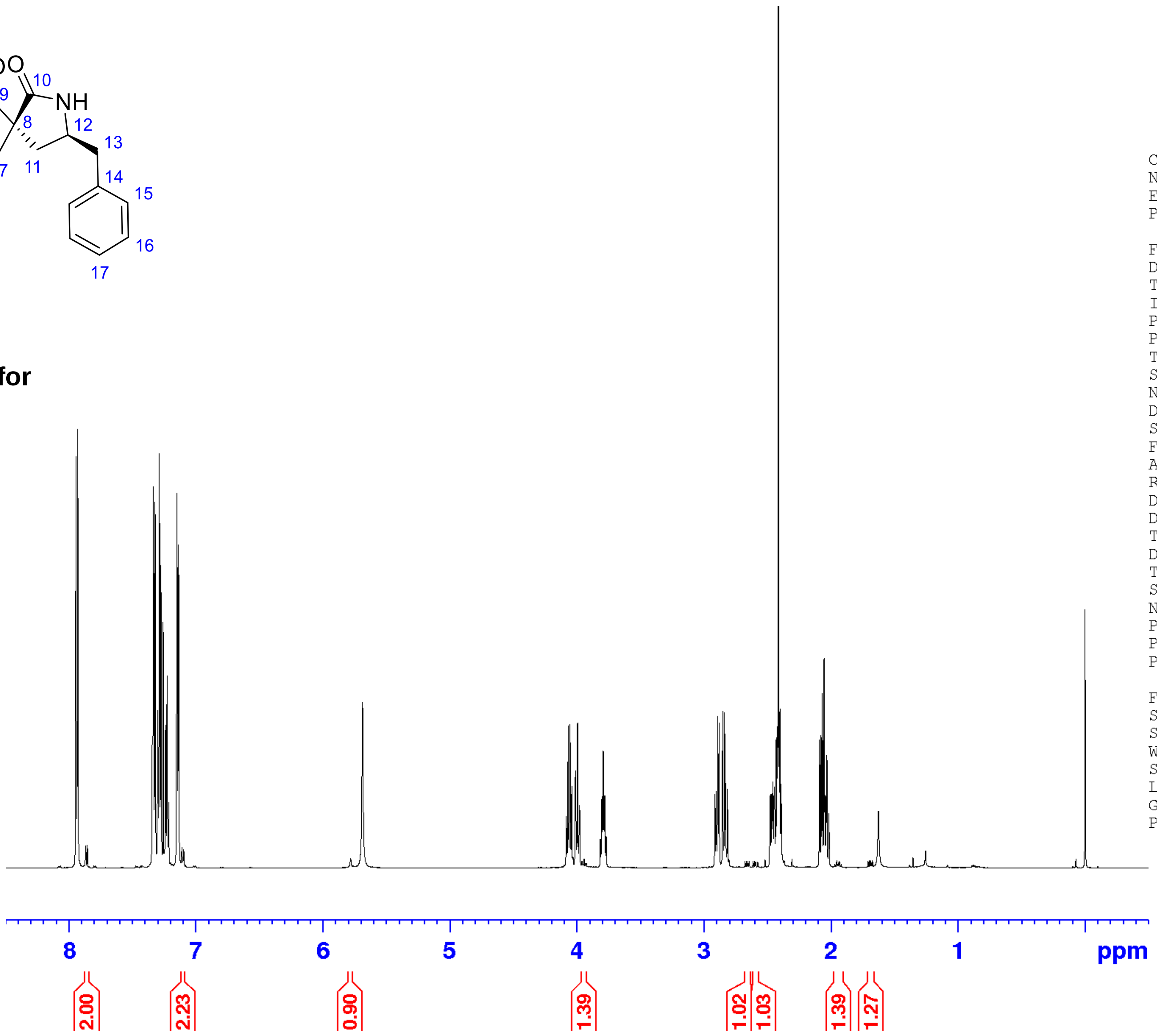

Current Data Parameters
NAME WR 1.121 (600)
EXPNO 20
PROCNO 1

F2 - Acquisition Parameters
Date_ 20220318
Time 17.51 h
INSTRUM spect
PROBHD Z114607_0188 (
PULPROG zg30
TD 65536
SOLVENT CDCl3
NS 16
DS 2
SWH 12019.230 Hz
FIDRES 0.366798 Hz
AQ 2.7262976 sec
RG 74.91
DW 41.600 usec
DE 12.10 usec
TE 300.0 K
D1 1.00000000 sec
TD0 1
SFO1 600.1337058 MHz
NUC1 1H
P0 3.33 usec
P1 10.00 usec
PLW1 26.60000038 W

F2 - Processing parameters
SI 65536
SF 600.1300145 MHz
WDW EM
SSB 0
LB 0.30 Hz
GB 0
PC 1.00

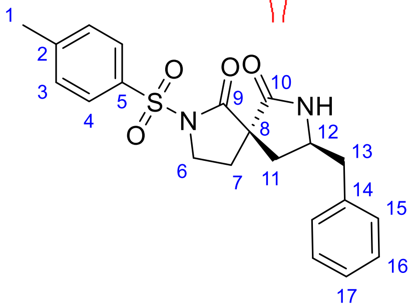

5

Major diastereomer

^{13}C NMR

151 MHz

$CDCl_3$

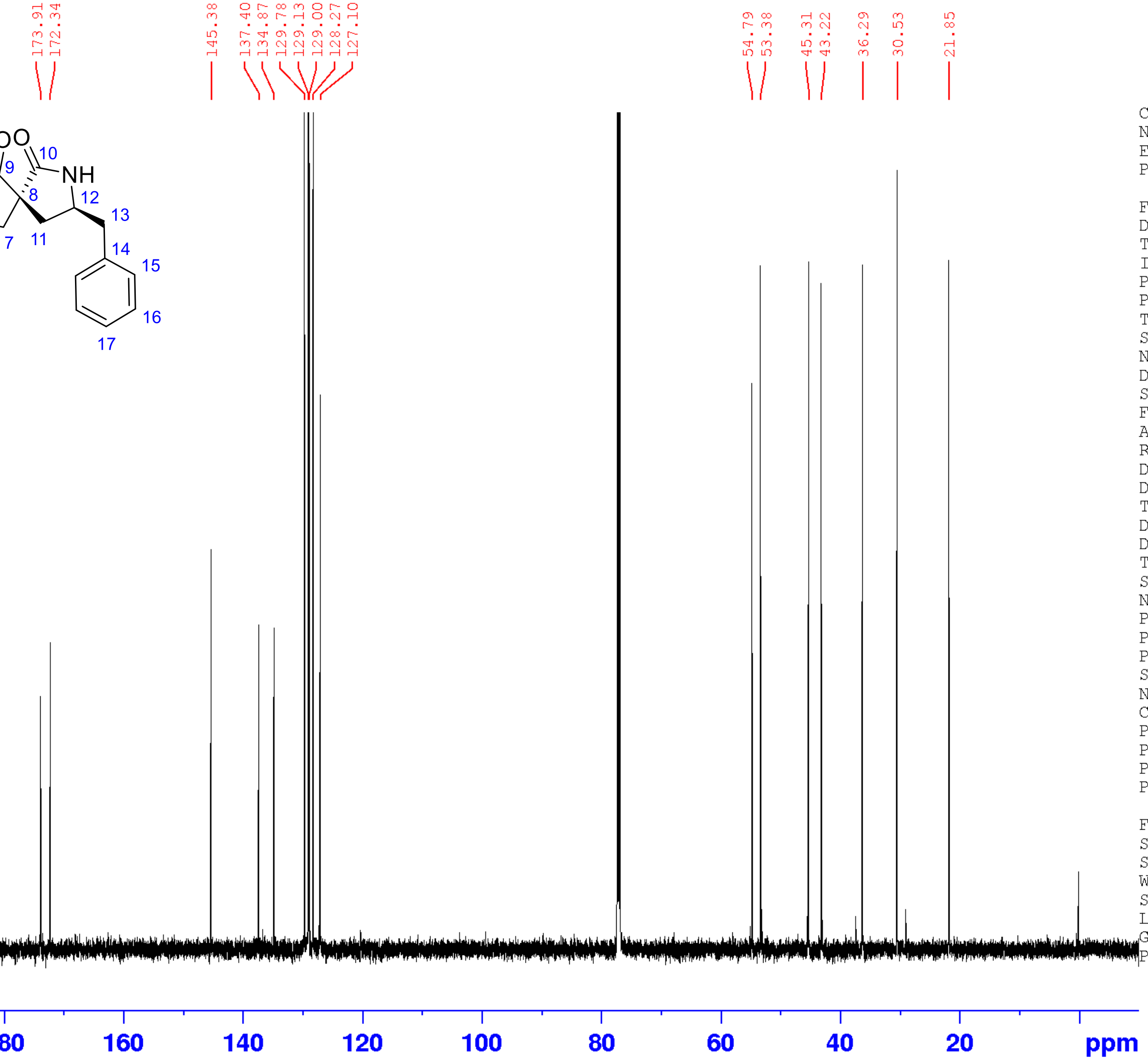

Current Data Parameters
NAME WR 1.121 (600)
EXPNO 11
PROCNO 1

F2 - Acquisition Parameters
Date_ 20220318
Time 18.43 h
INSTRUM spect
PROBHD Z114607_0188 (
PULPROG zgpg30
TD 65536
SOLVENT CDCl3
NS 1024
DS 4
SWH 36231.883 Hz
FIDRES 1.105709 Hz
AQ 0.9043968 sec
RG 186.92
DW 13.800 usec
DE 6.50 usec
TE 300.0 K
D1 2.00000000 sec
D11 0.03000000 sec
TD0 1
SFO1 150.9178988 MHz
NUC1 13C
P0 3.93 usec
P1 11.80 usec
PLW1 85.00000000 W
SFO2 600.1324005 MHz
NUC2 1H
CPDPRG[2 waltz65
PCPD2 70.00 usec
PLW2 27.00000000 W
PLW12 0.57327998 W
PLW13 0.28836000 W

F2 - Processing parameters
SI 32768
SF 150.9027920 MHz
WDW EM
SSB 0
LB 1.00 Hz
GB 0
PC 1.40

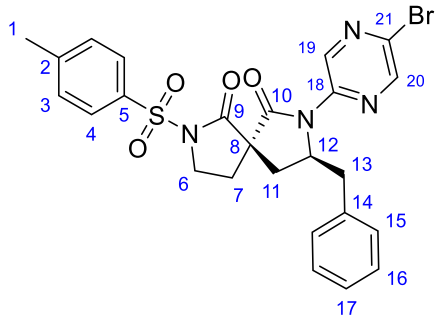

6

^{1}H NMR

400 MHz

$CDCl_3$

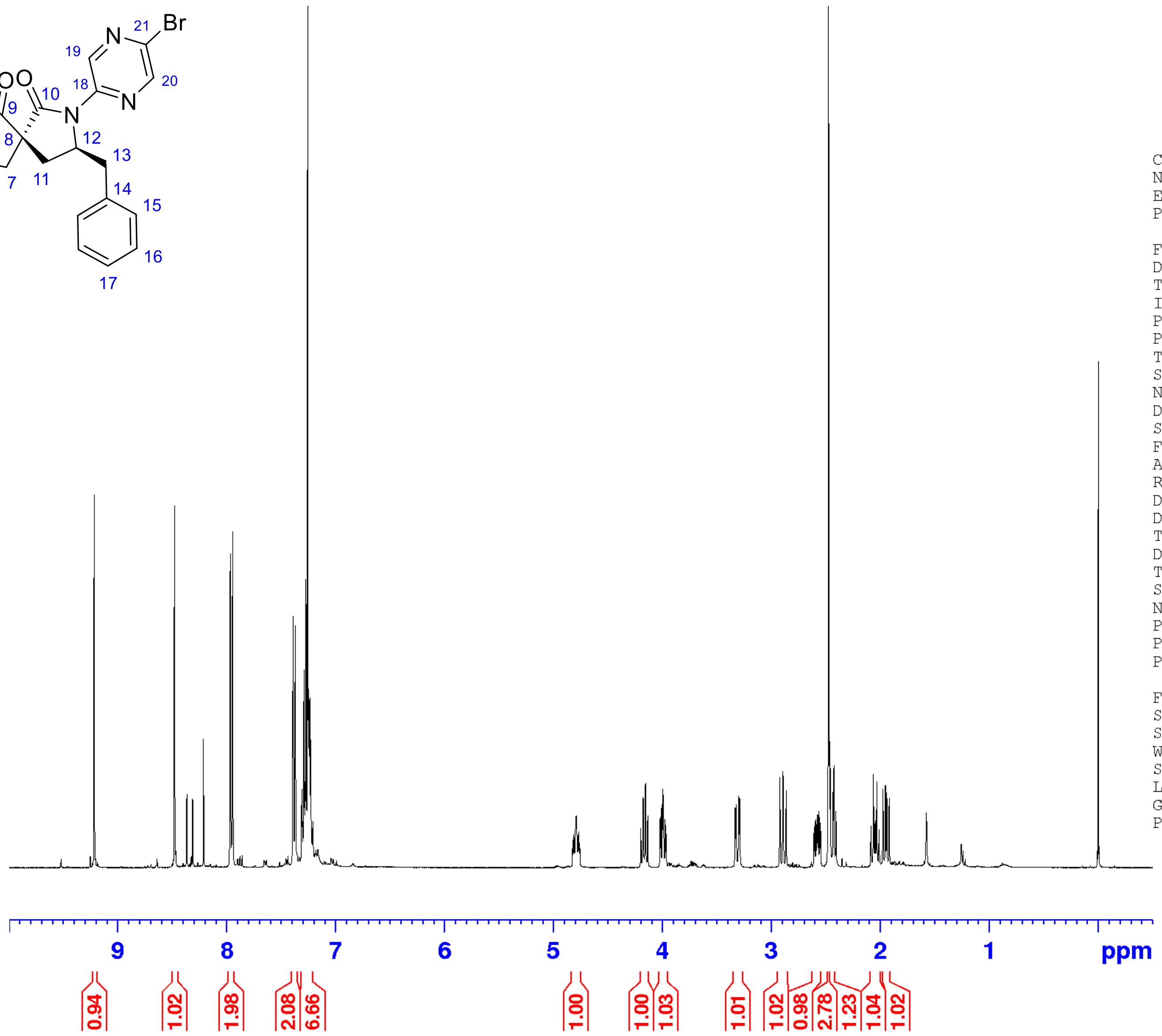


```
Current Data Parameters
NAME             WR 2.281
EXPNO                  10
PROCNO                  1

F2 - Acquisition Parameters
Date_            20220316
Time                16.22 h
INSTRUM         AVIII_400
PROBHD     Z108618_0146 (
PULPROG              zg30
TD                  65536
SOLVENT             CDCl3
NS                     16
DS                      2
SWH              8223.685 Hz
FIDRES           0.250967 Hz
AQ              3.9845889 sec
RG                    181
DW                 60.800 usec
DE                  17.42 usec
TE                  300.0 K
D1             1.00000000 sec
TD0                     1
SFO1          400.1124708 MHz
NUC1                   1H
P0                   5.00 usec
P1                  15.00 usec
PLW1          17.29199982 W

F2 - Processing parameters
SI                  32768
SF            400.1100097 MHz
WDW                    EM
SSB                     0
LB                   0.30 Hz
GB                      0
PC                   1.00
```

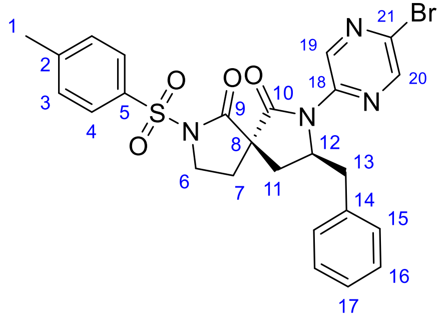

6

^{13}C NMR

101 MHz

$CDCl_3$

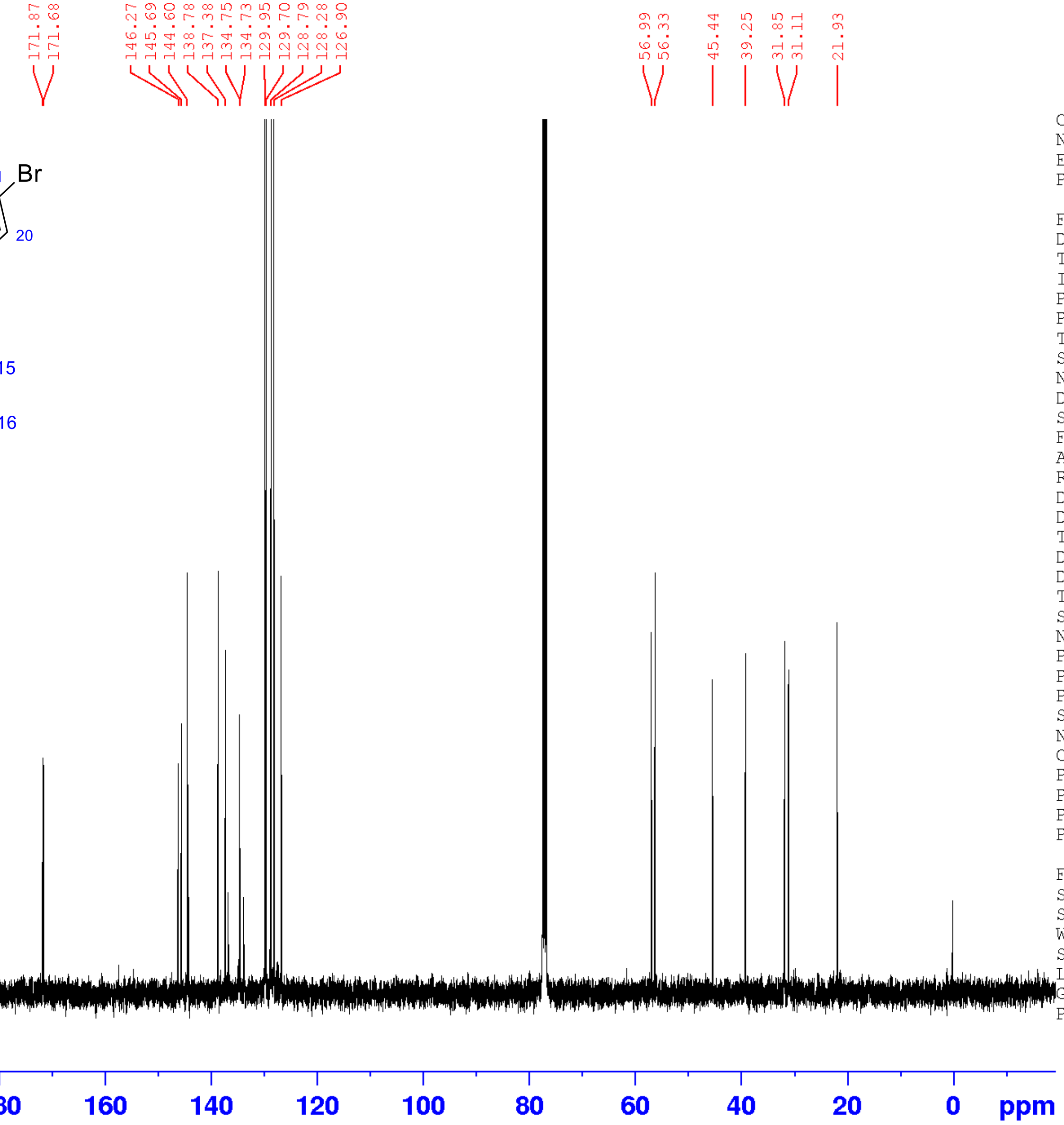

Current Data Parameters
NAME WR 2.281
EXPNO 11
PROCNO 1

F2 - Acquisition Parameters
Date_ 20220317
Time 15.00 h
INSTRUM AVIII_400
PROBHD Z108618_0146 (
PULPROG zgpg30
TD 96150
SOLVENT CDCl3
NS 1024
DS 4
SWH 24038.461 Hz
FIDRES 0.500020 Hz
AQ 1.9999200 sec
RG 2050
DW 20.800 usec
DE 6.50 usec
TE 300.0 K
D1 1.00000000 sec
D11 0.03000000 sec
TD0 1
SFO1 100.6178003 MHz
NUC1 13C
P0 3.00 usec
P1 9.00 usec
PLW1 96.68000031 W
SFO2 400.1116004 MHz
NUC2 1H
CPDPRG[2 waltz64
PCPD2 90.00 usec
PLW2 17.29199982 W
PLW12 0.48032999 W
PLW13 0.24160001 W

F2 - Processing parameters
SI 131072
SF 100.6077277 MHz
WDW EM
SSB 0
LB 1.00 Hz
GB 0
PC 1.40

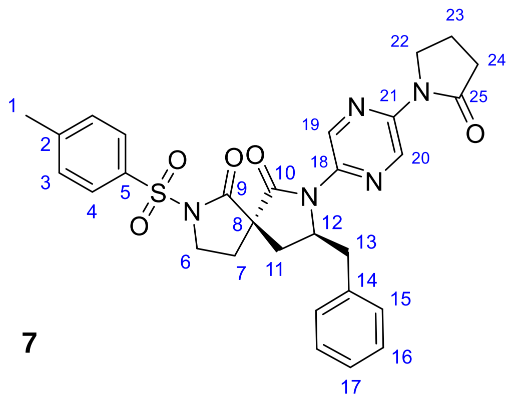

7

^{1}H NMR

400 MHz

$CDCl_3$

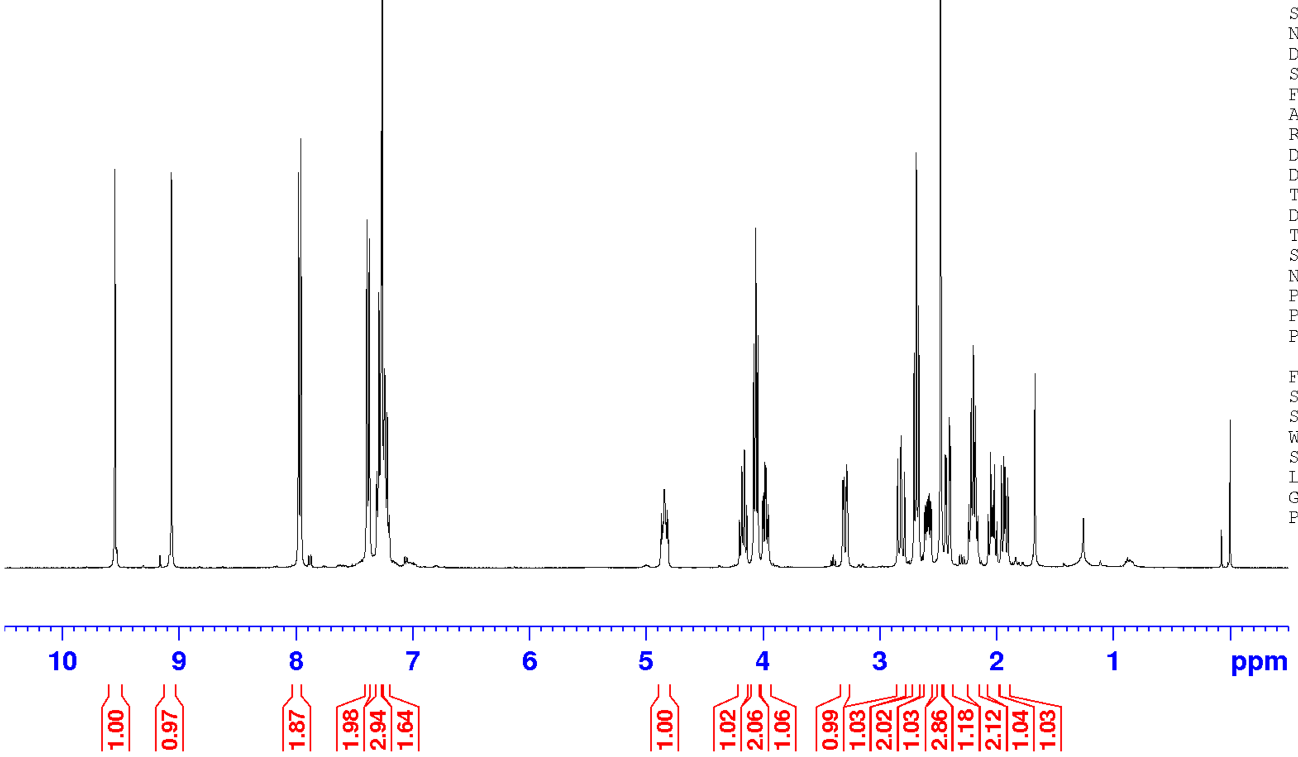


```
Current Data Parameters
NAME             WR 2.289
EXPNO                  10
PROCNO                  1

F2 - Acquisition Parameters
Date_            20220311
Time                17.46 h
INSTRUM          AVIII_400
PROBHD    Z108618_0146 (
PULPROG              zg30
TD                  65536
SOLVENT             CDCl3
NS                     16
DS                      2
SWH              8223.685 Hz
FIDRES           0.250967 Hz
AQ              3.9845889 sec
RG                    114
DW                 60.800 usec
DE                  17.42 usec
TE                  300.0 K
D1             1.00000000 sec
TD0                     1
SFO1          400.1124708 MHz
NUC1                   1H
P0                   5.00 usec
P1                  15.00 usec
PLW1          17.29199982 W

F2 - Processing parameters
SI                  32768
SF            400.1100081 MHz
WDW                    EM
SSB                     0
LB                   0.30 Hz
GB                      0
PC                   1.00
```

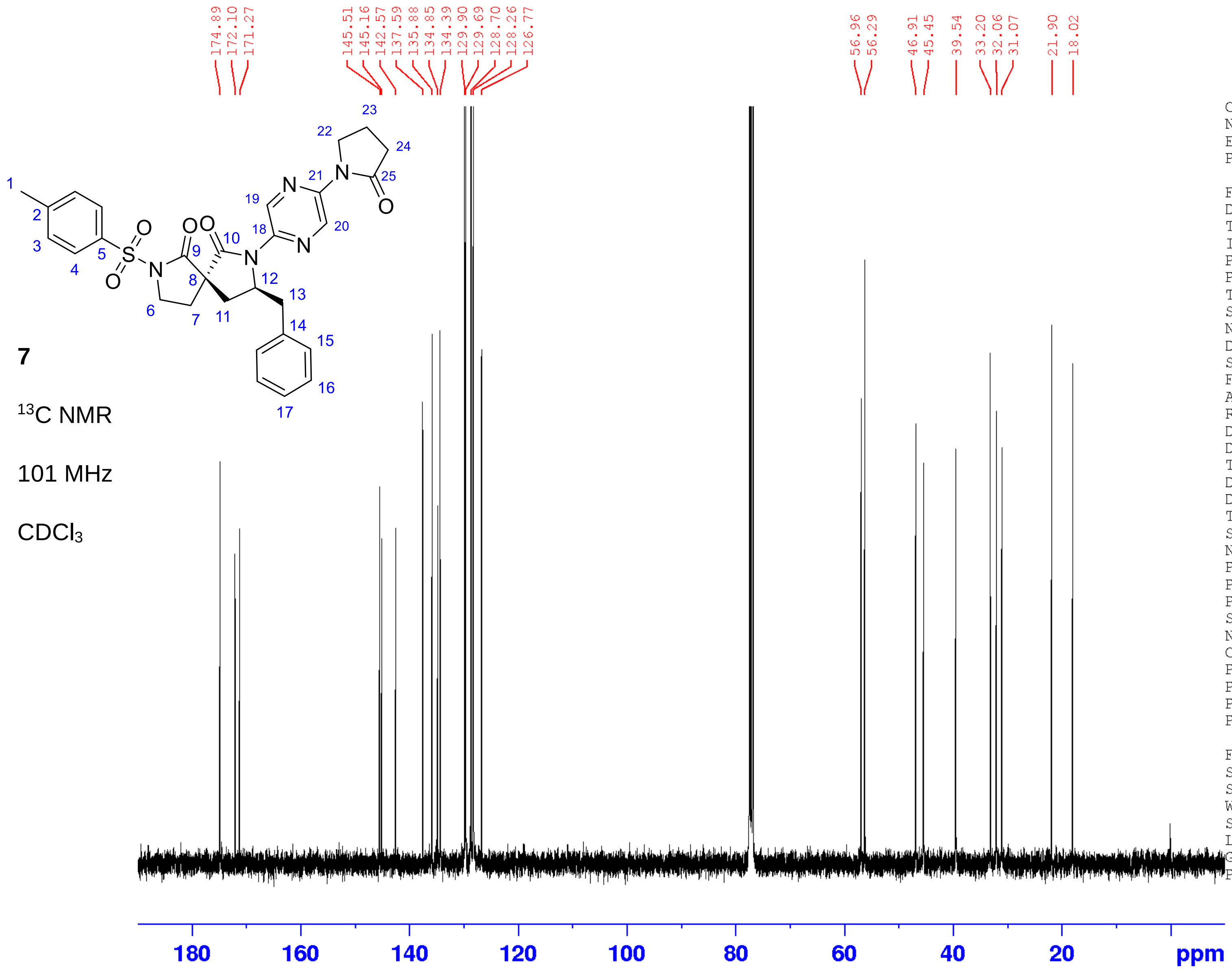

7

^{13}C NMR

101 MHz

$CDCl_3$


```
Current Data Parameters
NAME             WR 2.289
EXPNO                  11
PROCNO                  1

F2 - Acquisition Parameters
Date_            20220311
Time                18.40 h
INSTRUM          AVIII_400
PROBHD    Z108618_0146 (
PULPROG            zgpg30
TD                  96150
SOLVENT             CDCl3
NS                   1024
DS                      4
SWH             24038.461 Hz
FIDRES           0.500020 Hz
AQ              1.9999200 sec
RG                   2050
DW                 20.800 usec
DE                   6.50 usec
TE                  300.0 K
D1             1.00000000 sec
D11            0.03000000 sec
TD0                     1
SFO1          100.6178003 MHz
NUC1                  13C
P0                   3.00 usec
P1                   9.00 usec
PLW1          96.68000031 W
SFO2          400.1116004 MHz
NUC2                   1H
CPDPRG[2          waltz64
PCPD2               90.00 usec
PLW2          17.29199982 W
PLW12          0.48032999 W
PLW13          0.24160001 W

F2 - Processing parameters
SI                 131072
SF            100.6077299 MHz
WDW                    EM
SSB                     0
LB                   1.00 Hz
GB                      0
PC                   1.40
```

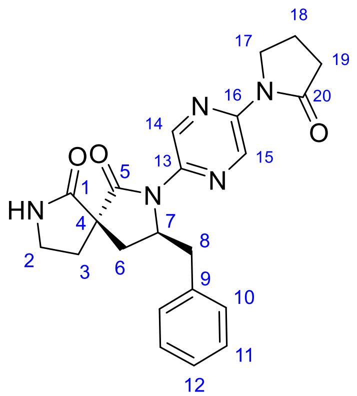

8

^{1}H NMR

600 MHz

$CDCl_3$

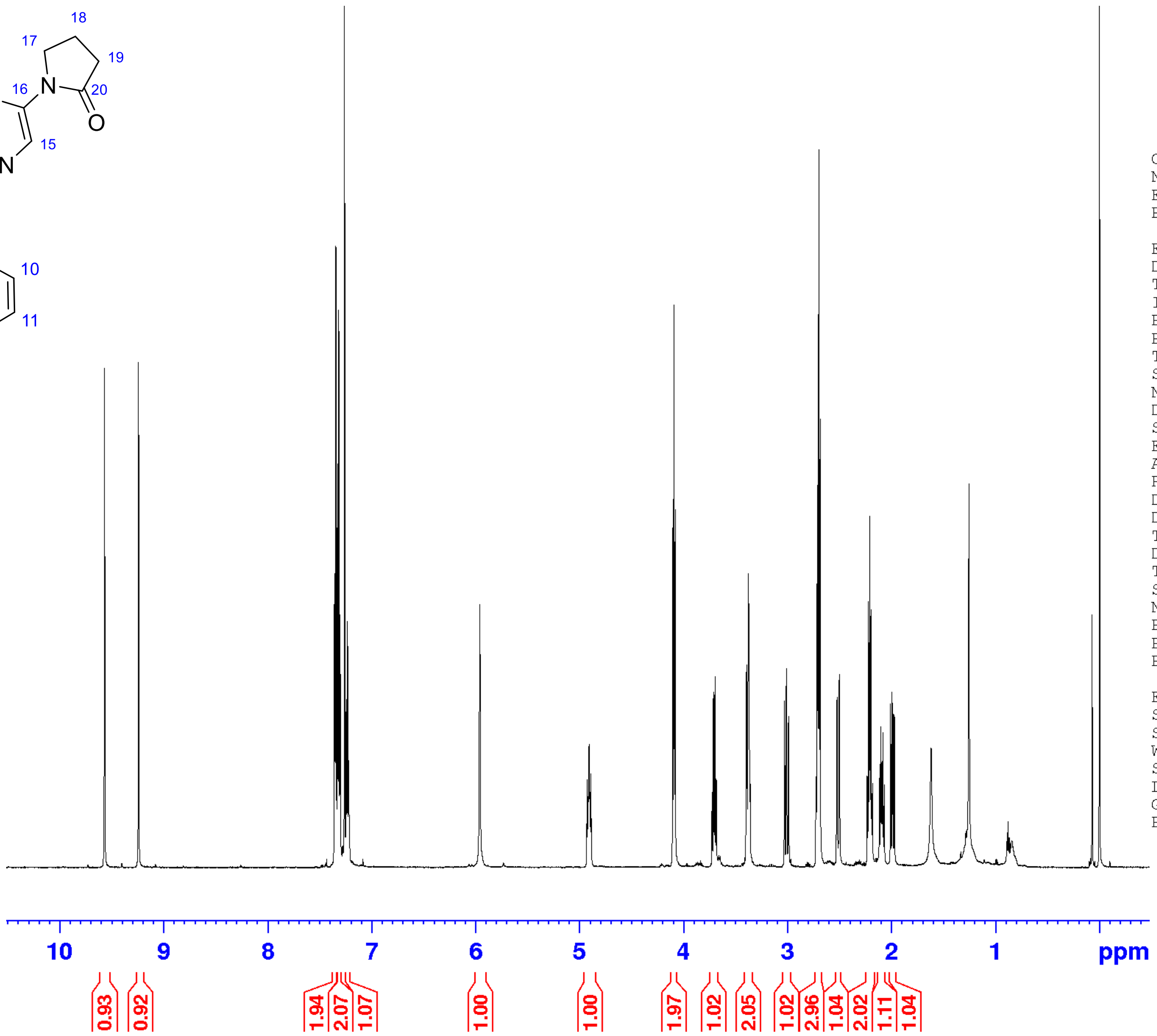


```
Current Data Parameters
NAME      WR 2.204 (600) V3
EXPNO                    10
PROCNO                    1

F2 - Acquisition Parameters
Date_              20220408
Time                  19.43 h
INSTRUM               spect
PROBHD     Z114607_0188 (
PULPROG                zg30
TD                    65536
SOLVENT               CDCl3
NS                       16
DS                        2
SWH               12019.230 Hz
FIDRES             0.366798 Hz
AQ                2.7262976 sec
RG                   105.21
DW                   41.600 usec
DE                    12.10 usec
TE                    298.2 K
D1               1.00000000 sec
TD0                       1
SFO1            600.1337058 MHz
NUC1                     1H
P0                     3.33 usec
P1                    10.00 usec
PLW1            26.60000038 W

F2 - Processing parameters
SI                    65536
SF              600.1300130 MHz
WDW                      EM
SSB                       0
LB                     0.30 Hz
GB                        0
PC                     1.00
```

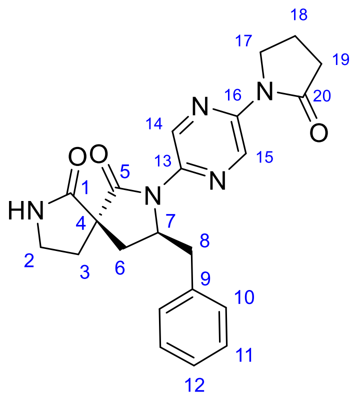

8

^{13}C NMR

151 MHz

$CDCl_3$

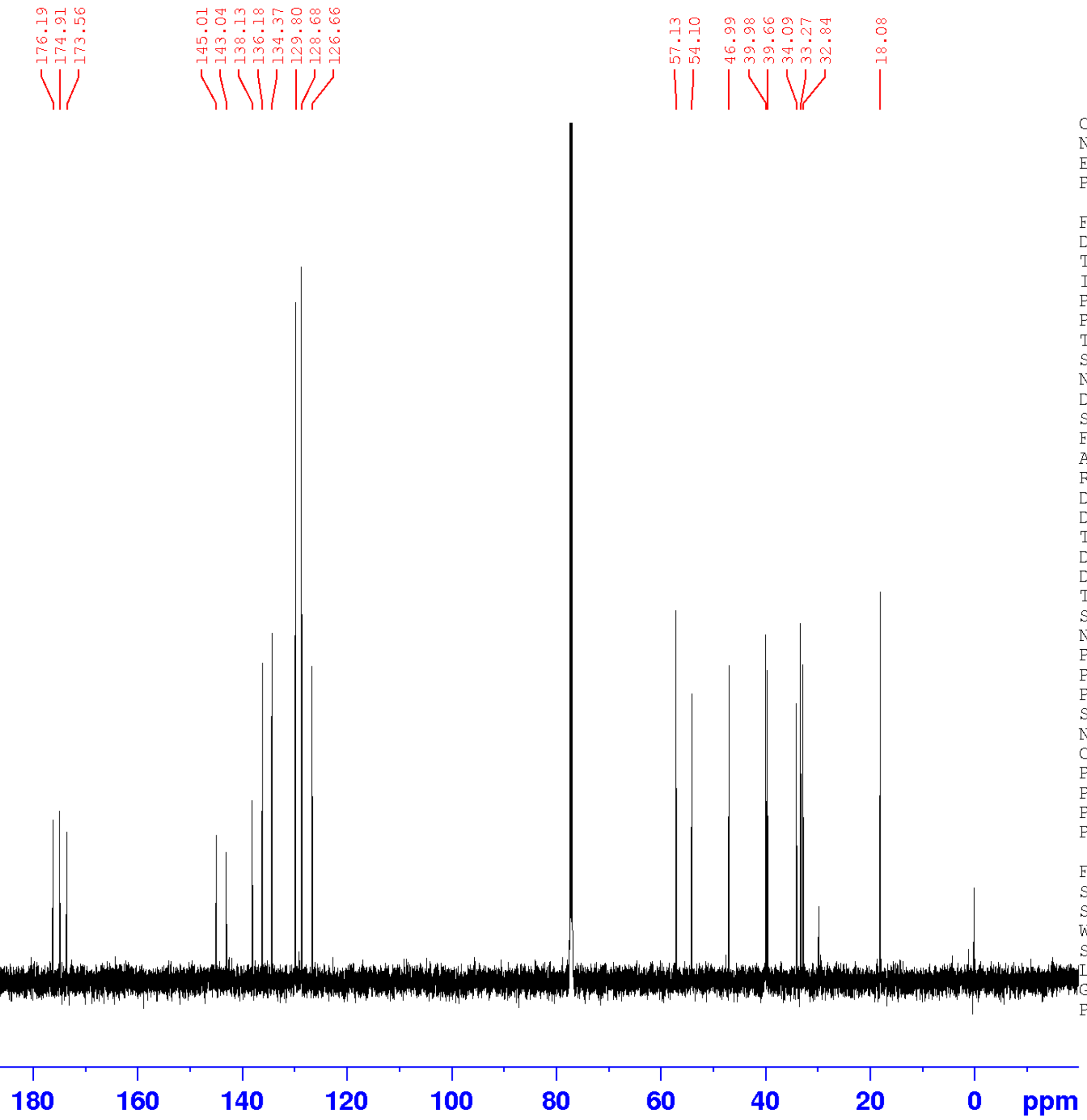

Current Data Parameters
NAME WR 2.204 (600) V3
EXPNO 11
PROCNO 1

F2 - Acquisition Parameters
Date_ 20220408
Time 20.35 h
INSTRUM spect
PROBHD Z114607_0188 (
PULPROG zgpg30
TD 65536
SOLVENT CDCl3
NS 1024
DS 4
SWH 36231.883 Hz
FIDRES 1.105709 Hz
AQ 0.9043968 sec
RG 186.92
DW 13.800 usec
DE 6.50 usec
TE 298.2 K
D1 2.00000000 sec
D11 0.03000000 sec
TD0 1
SFO1 150.9178988 MHz
NUC1 13C
P0 3.93 usec
P1 11.80 usec
PLW1 85.00000000 W
SFO2 600.1324005 MHz
NUC2 1H
CPDPRG[2 waltz65
PCPD2 70.00 usec
PLW2 27.00000000 W
PLW12 0.57327998 W
PLW13 0.28836000 W

F2 - Processing parameters
SI 32768
SF 150.9027889 MHz
WDW EM
SSB 0
LB 1.00 Hz
GB 0
PC 1.40

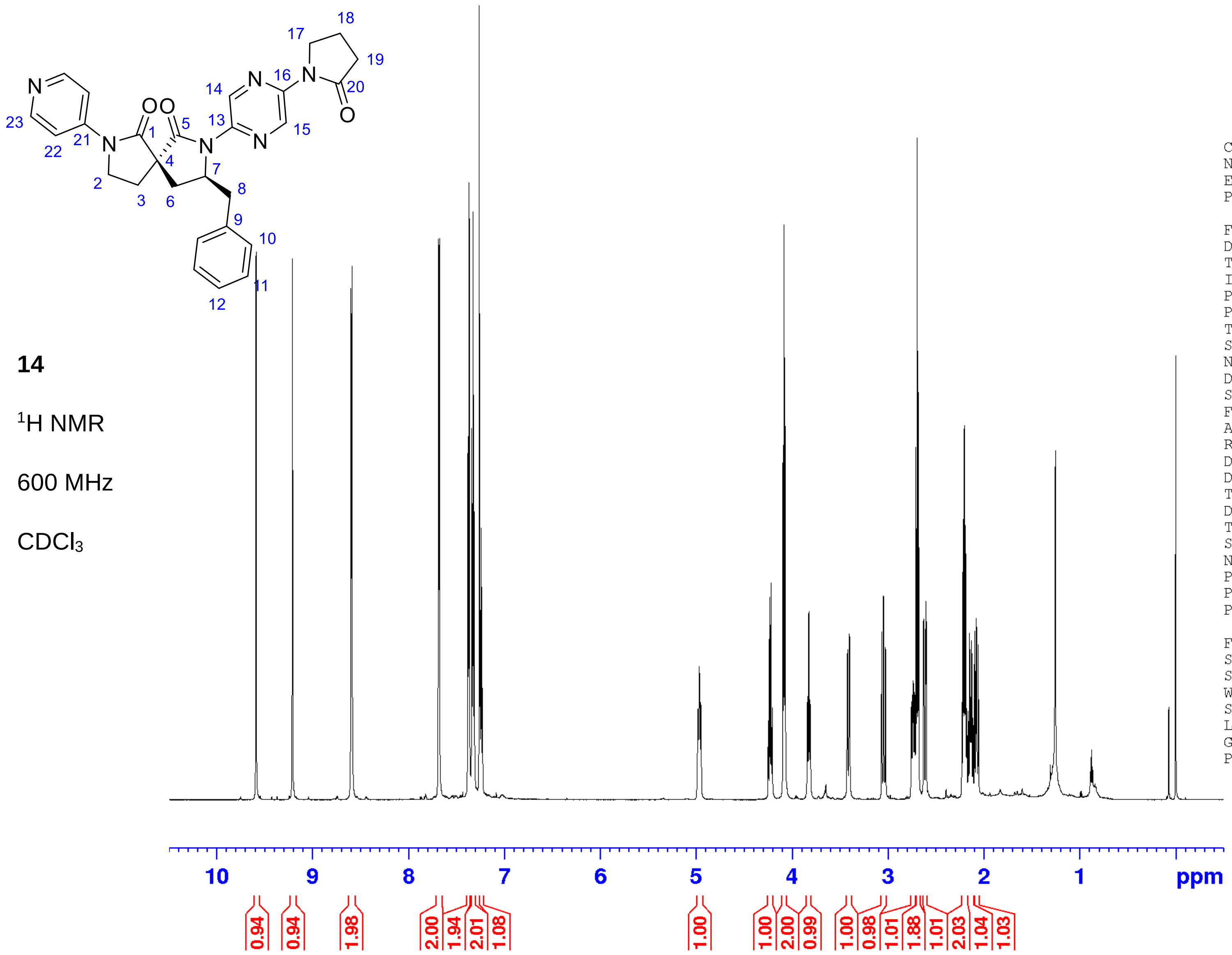

14

^{1}H NMR

600 MHz

$CDCl_3$

```
Current Data Parameters
NAME       WR 3.300 (600)
EXPNO                  10
PROCNO                  1

F2 - Acquisition Parameters
Date_            20220408
Time                 0.29 h
INSTRUM             spect
PROBHD    Z114607_0188 (
PULPROG              zg30
TD                  65536
SOLVENT             CDCl3
NS                     16
DS                      2
SWH             12019.230 Hz
FIDRES           0.366798 Hz
AQ              2.7262976 sec
RG                     68
DW                 41.600 usec
DE                  12.10 usec
TE                  300.0 K
D1             1.00000000 sec
TD0                     1
SFO1          600.1337058 MHz
NUC1                   1H
P0                   3.33 usec
P1                  10.00 usec
PLW1          26.60000038 W

F2 - Processing parameters
SI                  65536
SF            600.1300109 MHz
WDW                    EM
SSB                     0
LB                   0.30 Hz
GB                      0
PC                   1.00
```

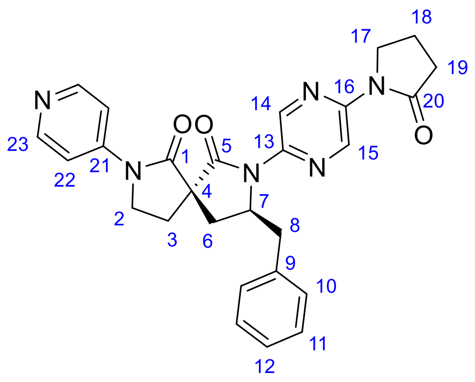

14

^{1}H NMR

600 MHz

d_6-DMSO

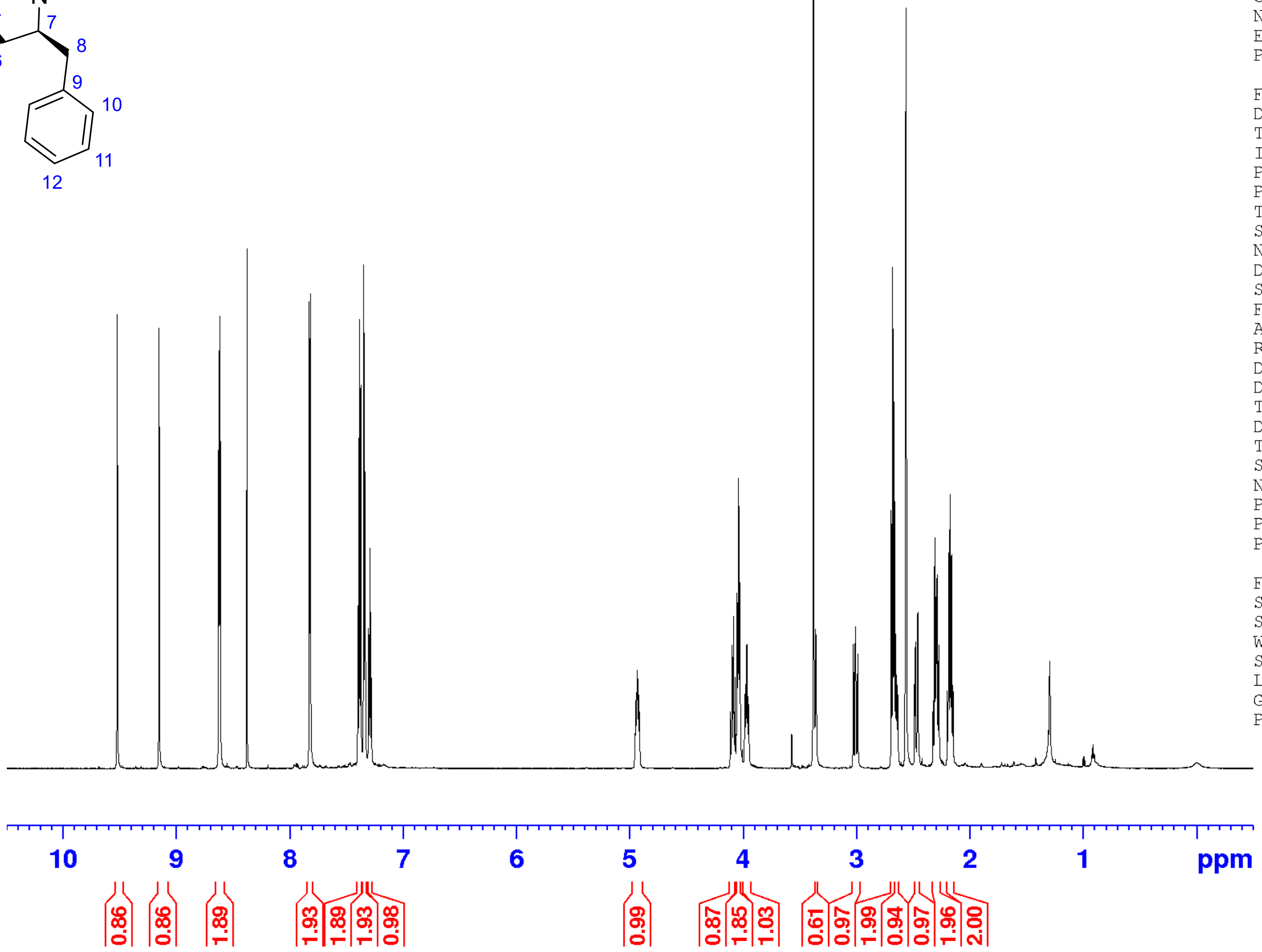


```
Current Data Parameters
NAME      WR 3.300 (DMSO)
EXPNO                  10
PROCNO                  1

F2 - Acquisition Parameters
Date_           20220411
Time               23.50 h
INSTRUM            spect
PROBHD   Z114607_0188 (
PULPROG             zg30
TD                 65536
SOLVENT             DMSO
NS                    16
DS                     2
SWH            12019.230 Hz
FIDRES          0.366798 Hz
AQ             2.7262976 sec
RG                  97.5
DW                41.600 usec
DE                 12.10 usec
TE                 300.0 K
D1            1.00000000 sec
TD0                    1
SFO1         600.1337058 MHz
NUC1                  1H
P0                  3.33 usec
P1                 10.00 usec
PLW1         26.60000038 W

F2 - Processing parameters
SI                 65536
SF           600.1299648 MHz
WDW                   EM
SSB                    0
LB                  0.30 Hz
GB                     0
PC                  1.00
```

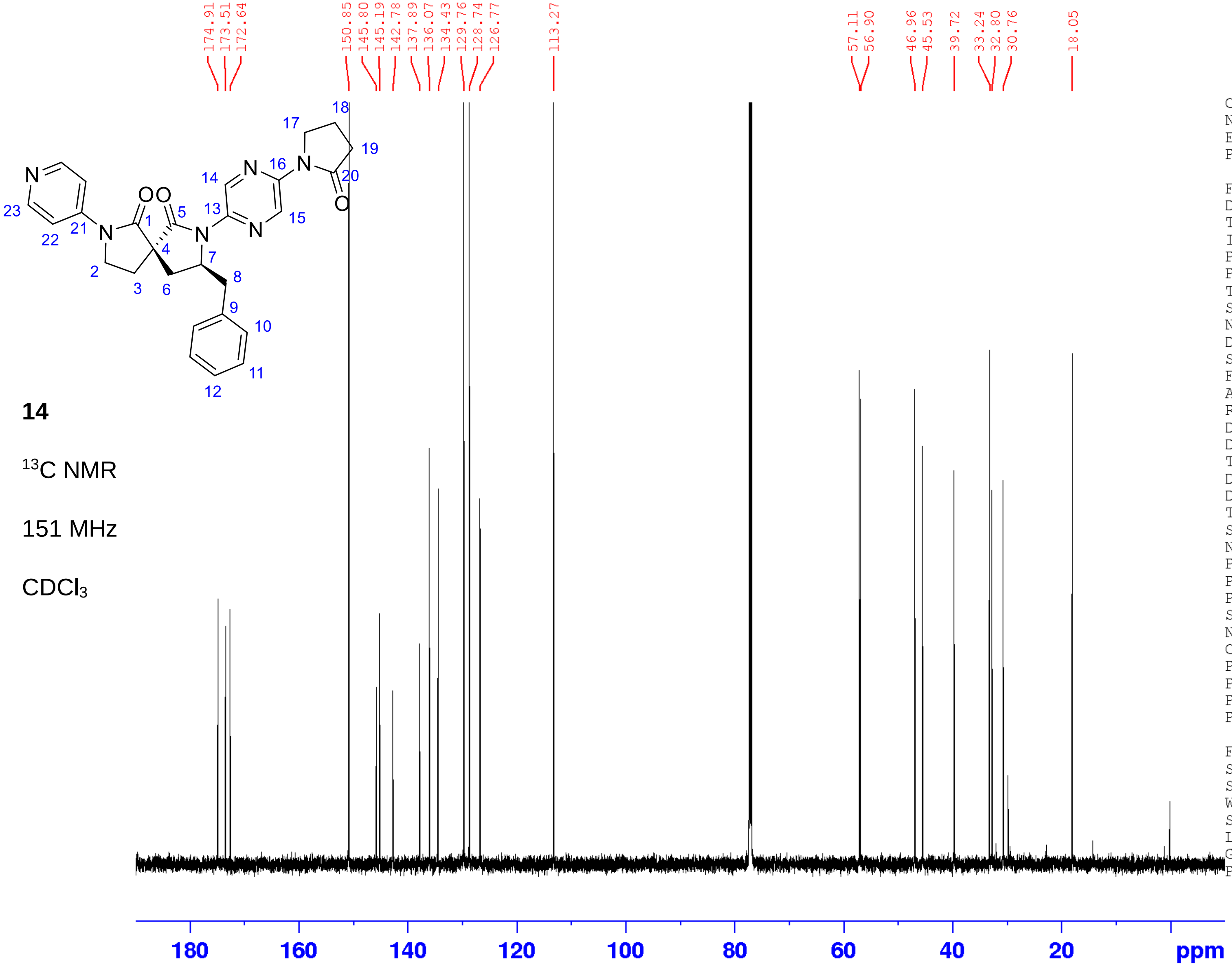

14

^{13}C NMR

151 MHz

$CDCl_3$

Current Data Parameters
NAME WR 3.300 (600)
EXPNO 11
PROCNO 1

F2 - Acquisition Parameters
Date_ 20220408
Time 1.20 h
INSTRUM spect
PROBHD Z114607_0188 (
PULPROG zgpg30
TD 65536
SOLVENT CDCl3
NS 1024
DS 4
SWH 36231.883 Hz
FIDRES 1.105709 Hz
AQ 0.9043968 sec
RG 186.92
DW 13.800 usec
DE 6.50 usec
TE 300.0 K
D1 2.00000000 sec
D11 0.03000000 sec
TD0 1
SFO1 150.9178988 MHz
NUC1 13C
P0 3.93 usec
P1 11.80 usec
PLW1 85.00000000 W
SFO2 600.1324005 MHz
NUC2 1H
CPDPRG[2 waltz65
PCPD2 70.00 usec
PLW2 27.00000000 W
PLW12 0.57327998 W
PLW13 0.28836000 W

F2 - Processing parameters
SI 32768
SF 150.9027910 MHz
WDW EM
SSB 0
LB 1.00 Hz
GB 0
PC 1.40

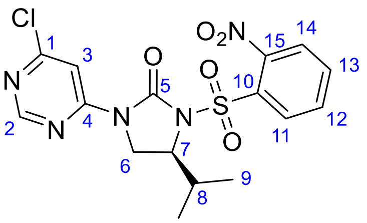

16

^{1}H NMR

400 MHz

$CDCl_3$

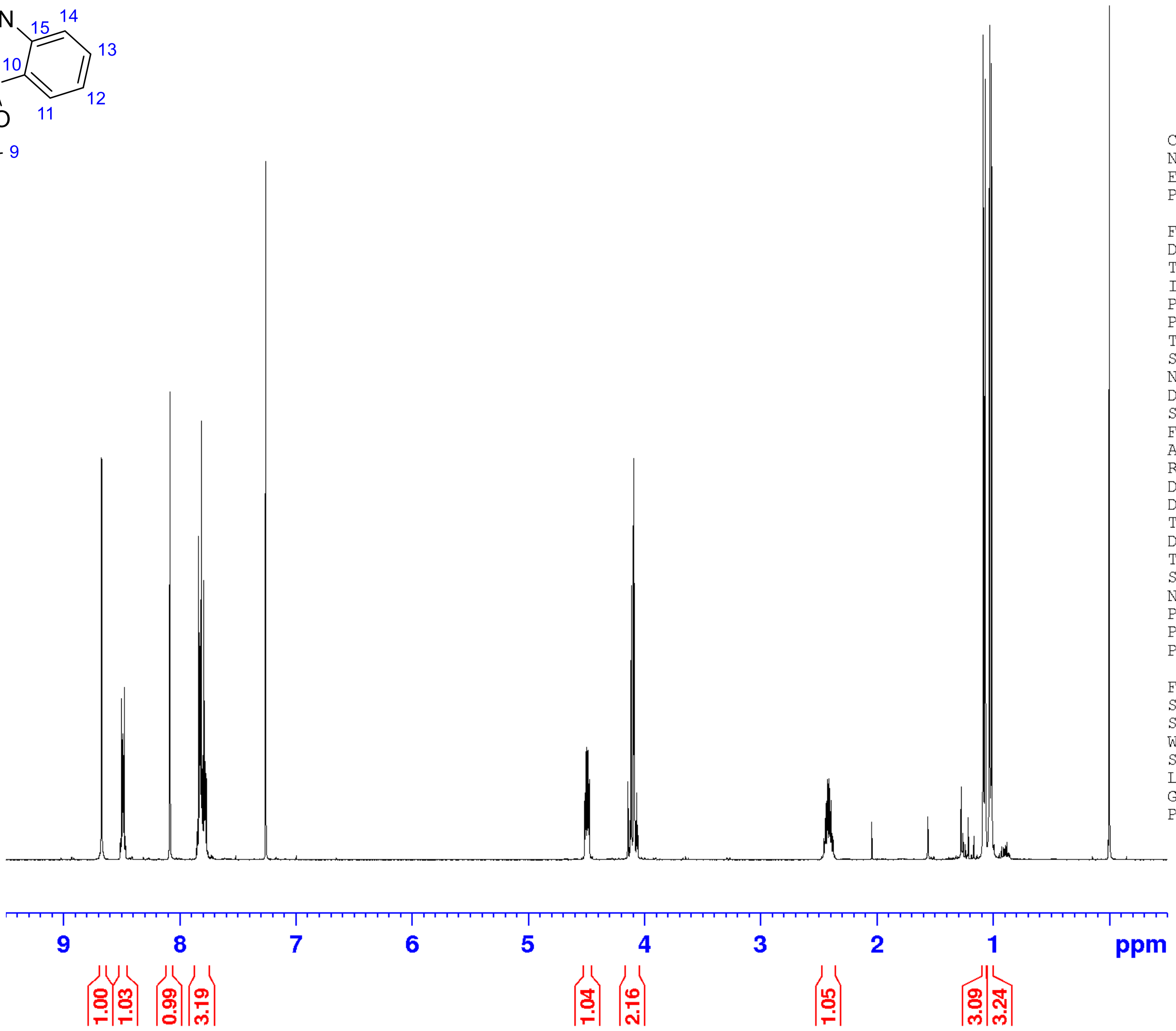


```
Current Data Parameters
NAME              WR 2.295
EXPNO                   10
PROCNO                   1

F2 - Acquisition Parameters
Date_             20220324
Time                 16.32 h
INSTRUM          AVIII_400
PROBHD      Z108618_0146 (
PULPROG               zg30
TD                   65536
SOLVENT              CDCl3
NS                      16
DS                       2
SWH               8223.685 Hz
FIDRES            0.250967 Hz
AQ               3.9845889 sec
RG                     256
DW                  60.800 usec
DE                   17.42 usec
TE                   300.0 K
D1              1.00000000 sec
TD0                      1
SFO1           400.1124708 MHz
NUC1                    1H
P0                    5.00 usec
P1                   15.00 usec
PLW1           17.29199982 W

F2 - Processing parameters
SI                   32768
SF             400.1100088 MHz
WDW                     EM
SSB                      0
LB                    0.30 Hz
GB                       0
PC                    1.00
```

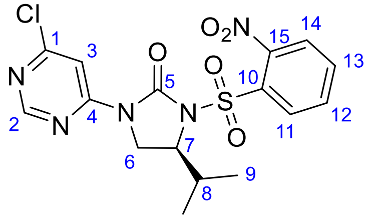

16

^{13}C NMR

101 MHz

$CDCl_3$

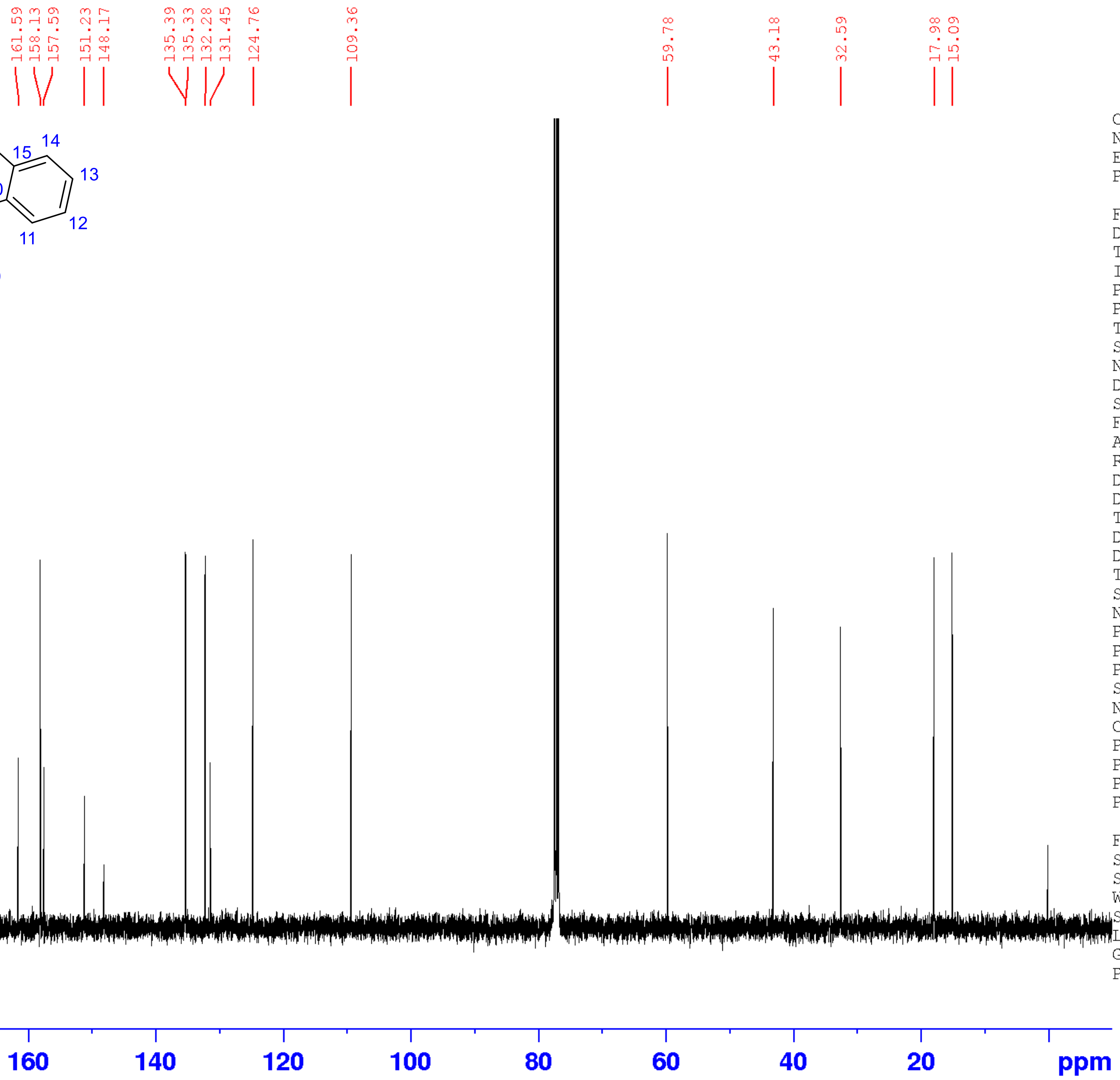

Current Data Parameters
NAME WR 2.295
EXPNO 11
PROCNO 1

F2 - Acquisition Parameters
Date_ 20220324
Time 17.46 h
INSTRUM AVIII_400
PROBHD Z108618_0146 (
PULPROG zgpg30
TD 96150
SOLVENT CDCl3
NS 1024
DS 4
SWH 24038.461 Hz
FIDRES 0.500020 Hz
AQ 1.9999200 sec
RG 2050
DW 20.800 usec
DE 6.50 usec
TE 300.0 K
D1 1.00000000 sec
D11 0.03000000 sec
TD0 1
SFO1 100.6178003 MHz
NUC1 13C
P0 3.00 usec
P1 9.00 usec
PLW1 96.68000031 W
SFO2 400.1116004 MHz
NUC2 1H
CPDPRG[2 waltz64
PCPD2 90.00 usec
PLW2 17.29199982 W
PLW12 0.48032999 W
PLW13 0.24160001 W

F2 - Processing parameters
SI 131072
SF 100.6077259 MHz
WDW EM
SSB 0
LB 1.00 Hz
GB 0
PC 1.40

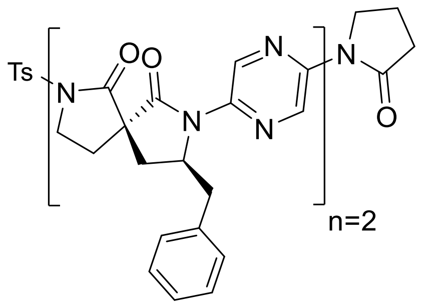

9

^{1}H NMR

600 MHz

$CDCl_3$

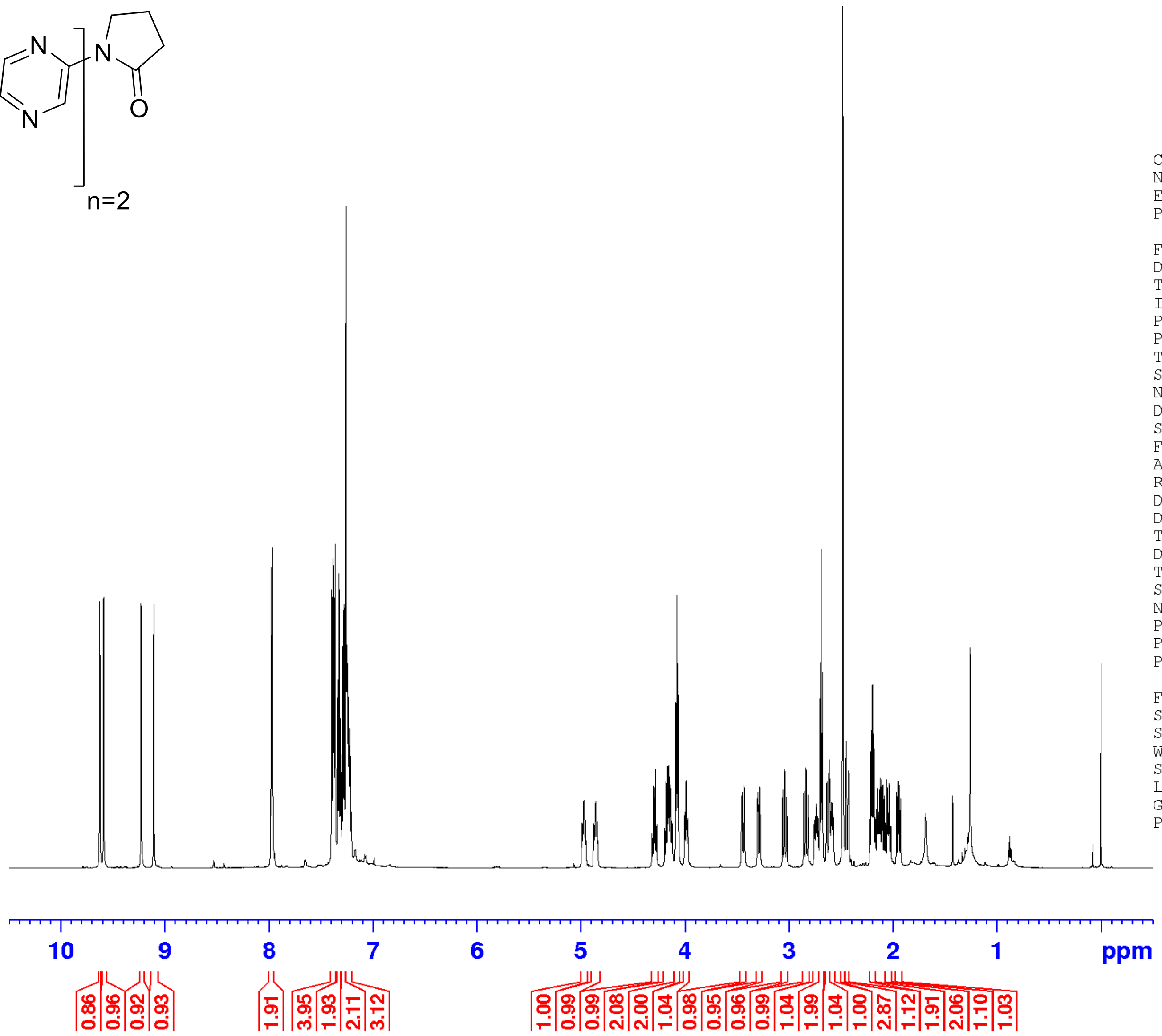


```
Current Data Parameters
NAME      WR 2.292 (600)
EXPNO                 10
PROCNO                 1

F2 - Acquisition Parameters
Date_           20220325
Time               22.56 h
INSTRUM            spect
PROBHD    Z114607_0188 (
PULPROG             zg30
TD                 65536
SOLVENT            CDCl3
NS                    16
DS                     2
SWH            12019.230 Hz
FIDRES          0.366798 Hz
AQ             2.7262976 sec
RG                 31.58
DW                41.600 usec
DE                 12.10 usec
TE                 300.0 K
D1            1.00000000 sec
TD0                    1
SFO1         600.1337058 MHz
NUC1                  1H
P0                  3.33 usec
P1                 10.00 usec
PLW1         26.60000038 W

F2 - Processing parameters
SI                 65536
SF           600.1300133 MHz
WDW                   EM
SSB                    0
LB                  0.30 Hz
GB                     0
PC                  1.00
```

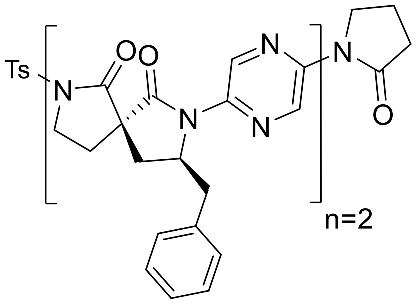

9

^{13}C NMR

151 MHz

$CDCl_3$

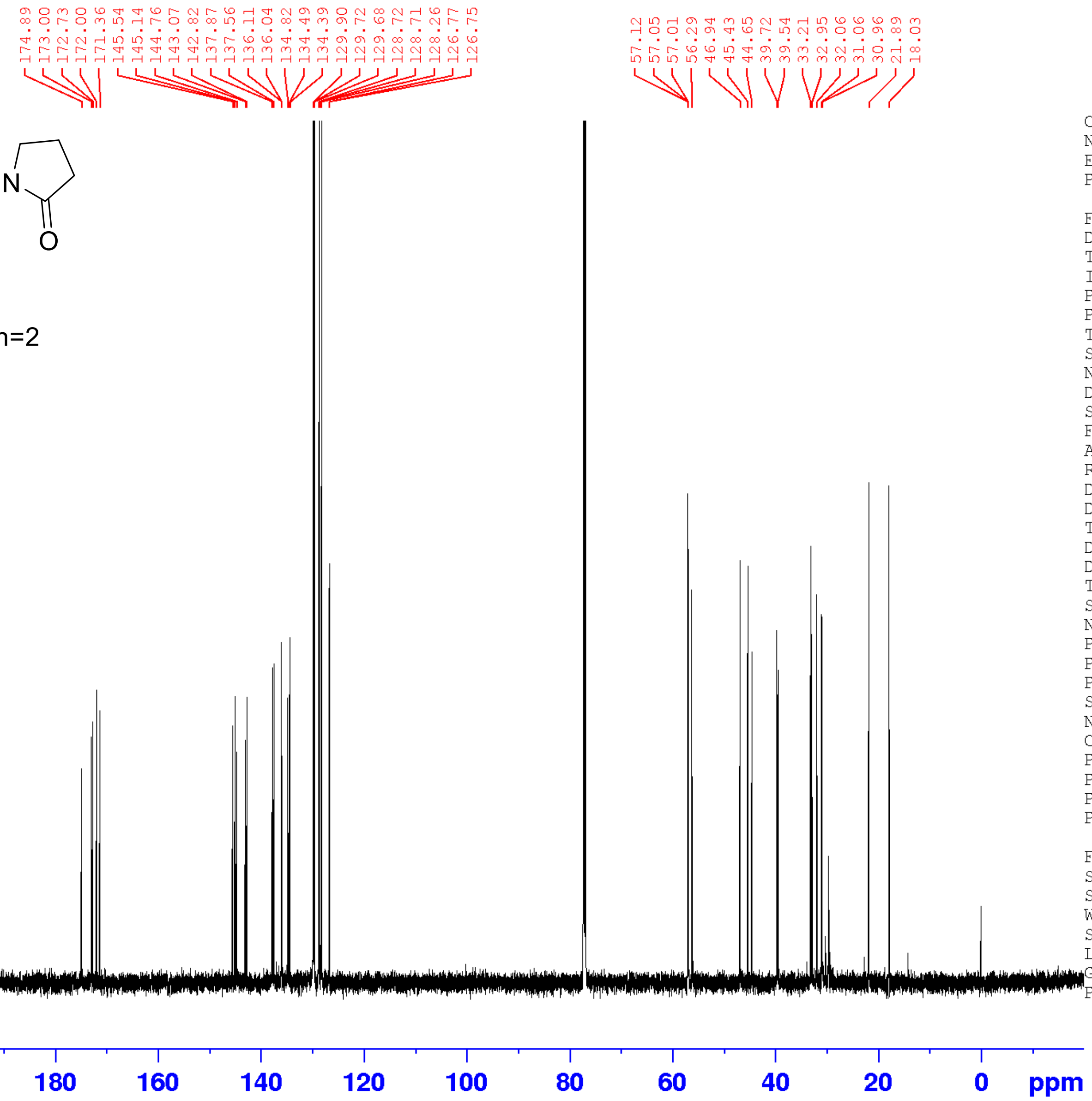


```
Current Data Parameters
NAME      WR 2.292 (600)
EXPNO                  11
PROCNO                  1

F2 - Acquisition Parameters
Date_            20220325
Time               23.47 h
INSTRUM             spect
PROBHD   Z114607_0188 (
PULPROG           zgpg30
TD                 65536
SOLVENT            CDCl3
NS                  1024
DS                     4
SWH            36231.883 Hz
FIDRES          1.105709 Hz
AQ             0.9043968 sec
RG                186.92
DW                13.800 usec
DE                  6.50 usec
TE                 300.0 K
D1            2.00000000 sec
D11           0.03000000 sec
TD0                    1
SFO1         150.9178988 MHz
NUC1                 13C
P0                  3.93 usec
P1                 11.80 usec
PLW1         85.00000000 W
SFO2         600.1324005 MHz
NUC2                  1H
CPDPRG[2         waltz65
PCPD2              70.00 usec
PLW2         27.00000000 W
PLW12         0.57327998 W
PLW13         0.28836000 W

F2 - Processing parameters
SI                 32768
SF           150.9027954 MHz
WDW                   EM
SSB                    0
LB                  1.00 Hz
GB                     0
PC                  1.40
```

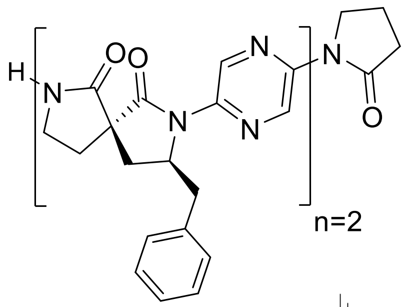

10

^{1}H NMR

600 MHz

$CDCl_3$

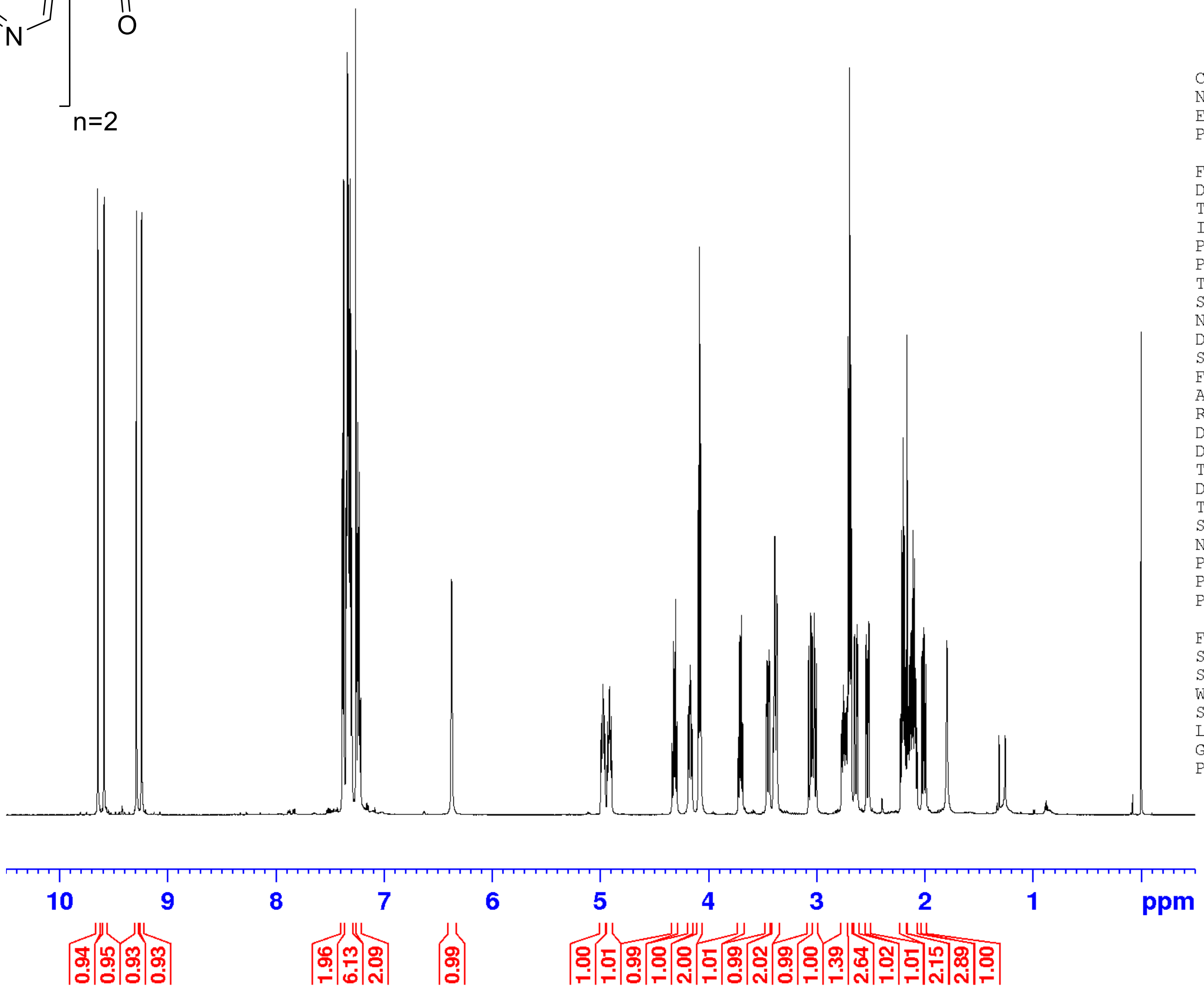


```
Current Data Parameters
NAME      WR 2.293 (600)
EXPNO                 10
PROCNO                 1

F2 - Acquisition Parameters
Date_           20220326
Time                0.40 h
INSTRUM            spect
PROBHD    Z114607_0188 (
PULPROG             zg30
TD                 65536
SOLVENT            CDCl3
NS                    16
DS                     2
SWH            12019.230 Hz
FIDRES          0.366798 Hz
AQ             2.7262976 sec
RG                 49.63
DW                41.600 usec
DE                 12.10 usec
TE                 300.0 K
D1            1.00000000 sec
TD0                    1
SFO1         600.1337058 MHz
NUC1                  1H
P0                  3.33 usec
P1                 10.00 usec
PLW1         26.60000038 W

F2 - Processing parameters
SI                 65536
SF           600.1300111 MHz
WDW                   EM
SSB                    0
LB                  0.30 Hz
GB                     0
PC                  1.00
```

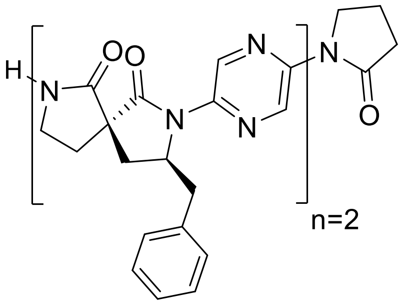

10

^{13}C NMR

151 MHz

$CDCl_3$

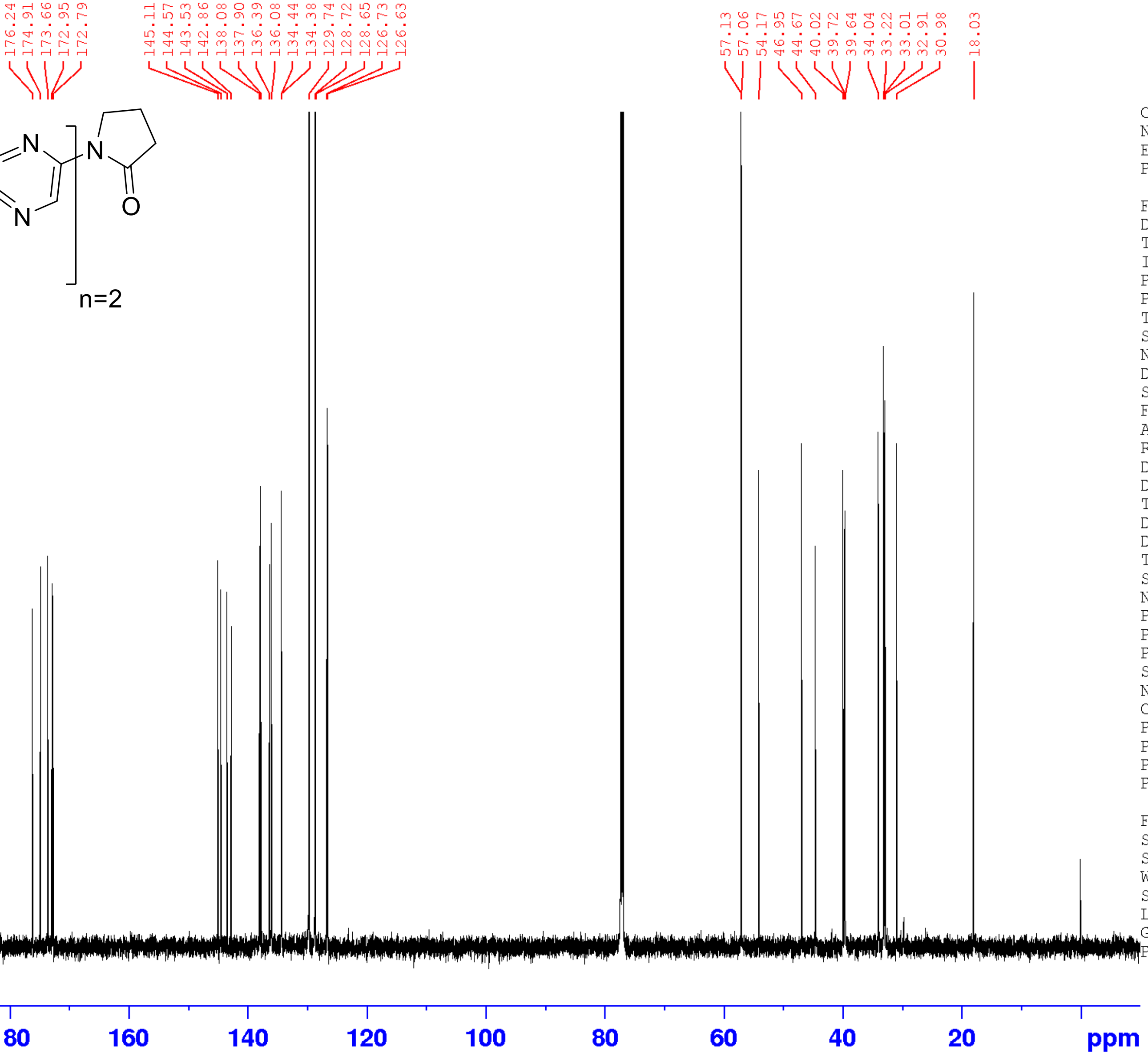


```
Current Data Parameters
NAME      WR 2.293 (600)
EXPNO                 11
PROCNO                 1

F2 - Acquisition Parameters
Date_           20220326
Time                1.31 h
INSTRUM            spect
PROBHD    Z114607_0188 (
PULPROG          zgpg30
TD                 65536
SOLVENT            CDCl3
NS                  1024
DS                     4
SWH           36231.883 Hz
FIDRES         1.105709 Hz
AQ            0.9043968 sec
RG               186.92
DW               13.800 usec
DE                 6.50 usec
TE                300.0 K
D1           2.00000000 sec
D11          0.03000000 sec
TD0                    1
SFO1        150.9178988 MHz
NUC1                 13C
P0                  3.93 usec
P1                 11.80 usec
PLW1        85.00000000 W
SFO2        600.1324005 MHz
NUC2                  1H
CPDPRG[2         waltz65
PCPD2              70.00 usec
PLW2        27.00000000 W
PLW12        0.57327998 W
PLW13        0.28836000 W

F2 - Processing parameters
SI                 32768
SF          150.9027950 MHz
WDW                   EM
SSB                    0
LB                  1.00 Hz
GB                     0
PC                  1.40
```

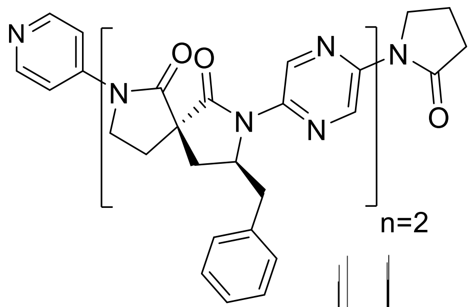

15

^{1}H NMR

600 MHz

$CDCl_3$

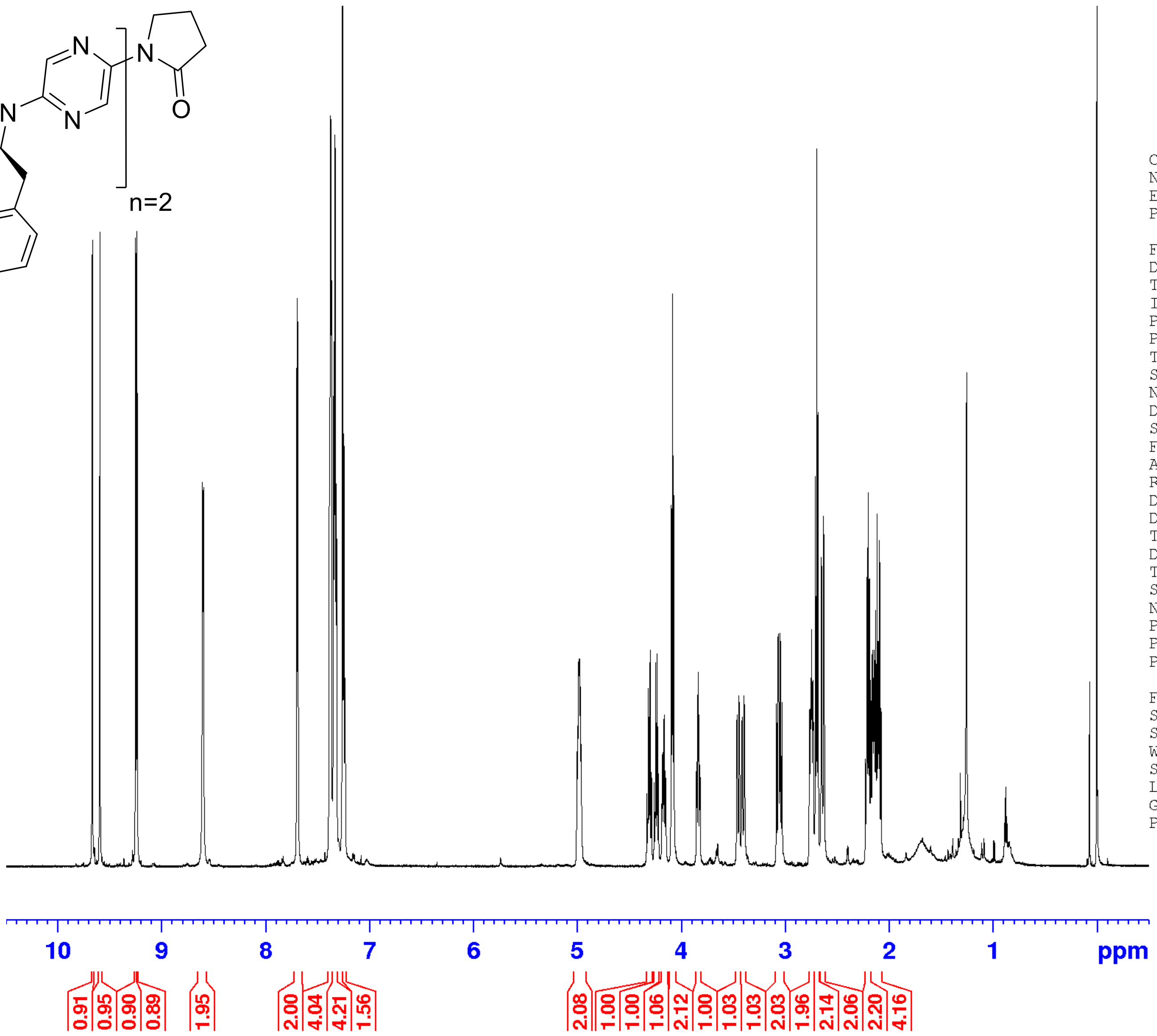


```
Current Data Parameters
NAME      WR 3.299 (600)
EXPNO                 12
PROCNO                 1

F2 - Acquisition Parameters
Date_           20220402
Time               14.02 h
INSTRUM            spect
PROBHD    Z114607_0188 (
PULPROG             zg30
TD                180286
SOLVENT            CDC13
NS                    16
DS                     0
SWH            18028.846 Hz
FIDRES          0.200003 Hz
AQ             4.9999318 sec
RG                  97.5
DW                27.733 usec
DE                  8.00 usec
TE                 300.0 K
D1            0.10000000 sec
TD0                    1
SFO1         600.1337060 MHz
NUC1                  1H
P0                  3.33 usec
P1                 10.00 usec
PLW1         26.60000038 W

F2 - Processing parameters
SI                262144
SF           600.1300136 MHz
WDW                   EM
SSB                    0
LB                  0.10 Hz
GB                     0
PC                  1.00
```

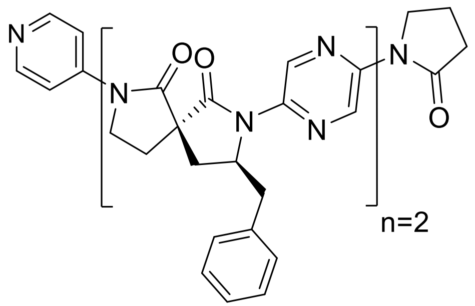

15

^{1}H NMR

600 MHz

d$_6$-DMSO

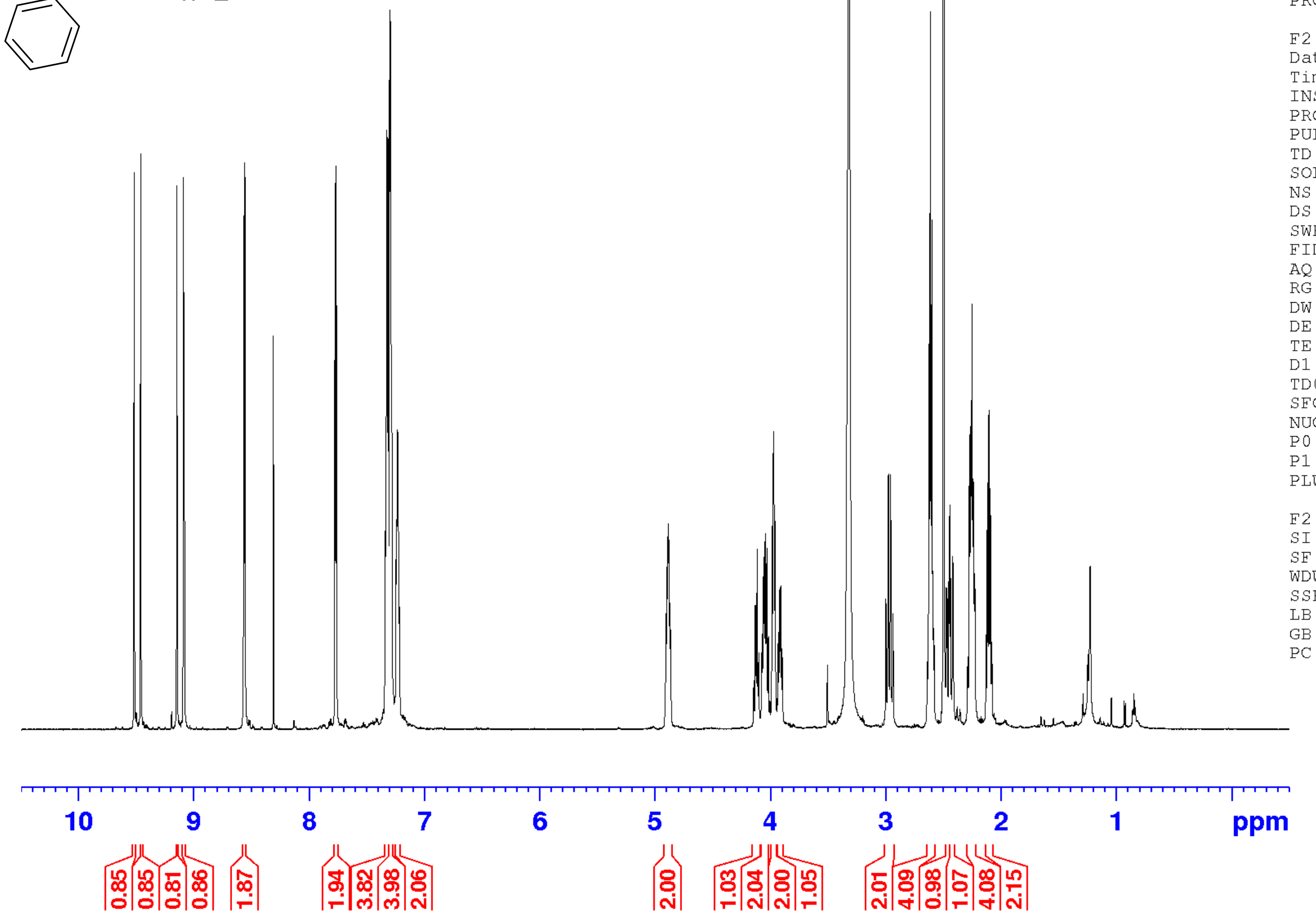


```
Current Data Parameters
NAME      WR 3.299 (DMSO Full)
EXPNO                       10
PROCNO                       1

F2 - Acquisition Parameters
Date_               20220505
Time                   19.02 h
INSTRUM                spect
PROBHD       Z114607_0188 (
PULPROG                 zg30
TD                     65536
SOLVENT                 DMSO
NS                        16
DS                         2
SWH             12019.230 Hz
FIDRES           0.366798 Hz
AQ              2.7262976 sec
RG                     83.95
DW                    41.600 usec
DE                     12.10 usec
TE                     300.0 K
D1             1.00000000 sec
TD0                        1
SFO1         600.1337058 MHz
NUC1                      1H
P0                      3.33 usec
P1                     10.00 usec
PLW1          26.60000038 W

F2 - Processing parameters
SI                     65536
SF           600.1300040 MHz
WDW                       EM
SSB                        0
LB                      0.30 Hz
GB                         0
PC                      1.00
```

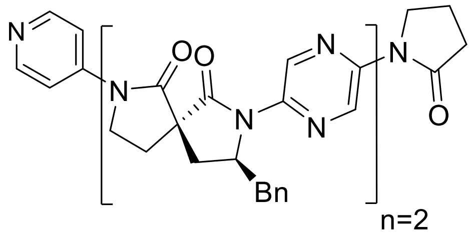

15

^{13}C NMR

151 MHz

$CDCl_3$

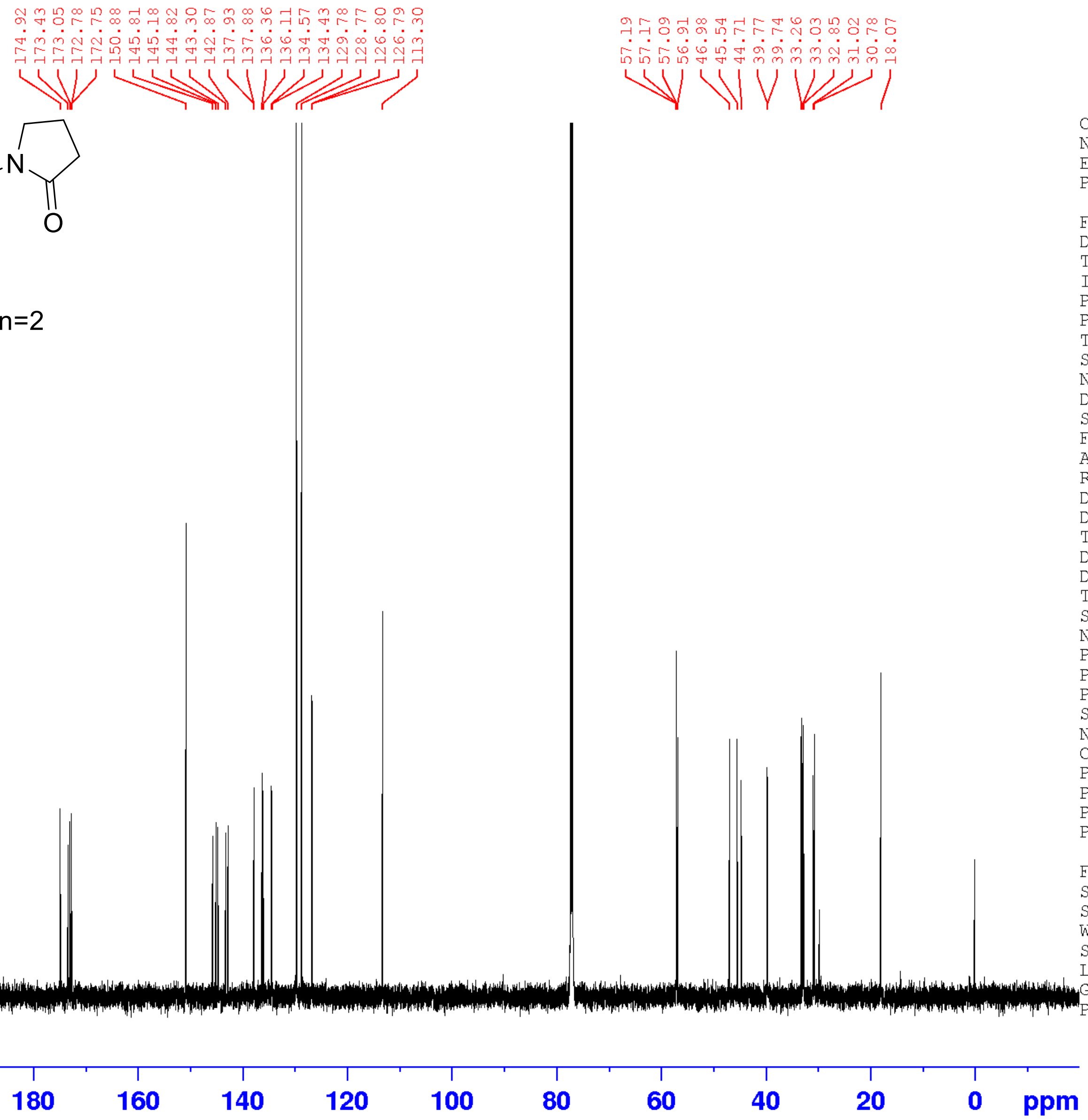


```
Current Data Parameters
NAME       WR 3.299 (600)
EXPNO                  11
PROCNO                  1

F2 - Acquisition Parameters
Date_            20220402
Time                14.01 h
INSTRUM             spect
PROBHD     Z114607_0188 (
PULPROG           zgpg30
TD                 65536
SOLVENT            CDCl3
NS                  2048
DS                     4
SWH            36231.883 Hz
FIDRES          1.105709 Hz
AQ             0.9043968 sec
RG                186.92
DW                13.800 usec
DE                  6.50 usec
TE                 300.0 K
D1            2.00000000 sec
D11           0.03000000 sec
TD0                    1
SFO1         150.9178988 MHz
NUC1                 13C
P0                  3.93 usec
P1                 11.80 usec
PLW1         85.00000000 W
SFO2         600.1324005 MHz
NUC2                  1H
CPDPRG[2         waltz65
PCPD2              70.00 usec
PLW2         27.00000000 W
PLW12         0.57327998 W
PLW13         0.28836000 W

F2 - Processing parameters
SI                 32768
SF           150.9027892 MHz
WDW                   EM
SSB                    0
LB                  1.00 Hz
GB                     0
PC                  1.40
```

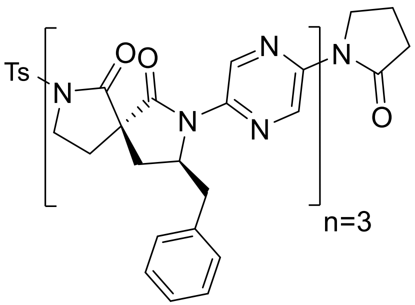

11

^{1}H NMR

600 MHz

$CDCl_3$

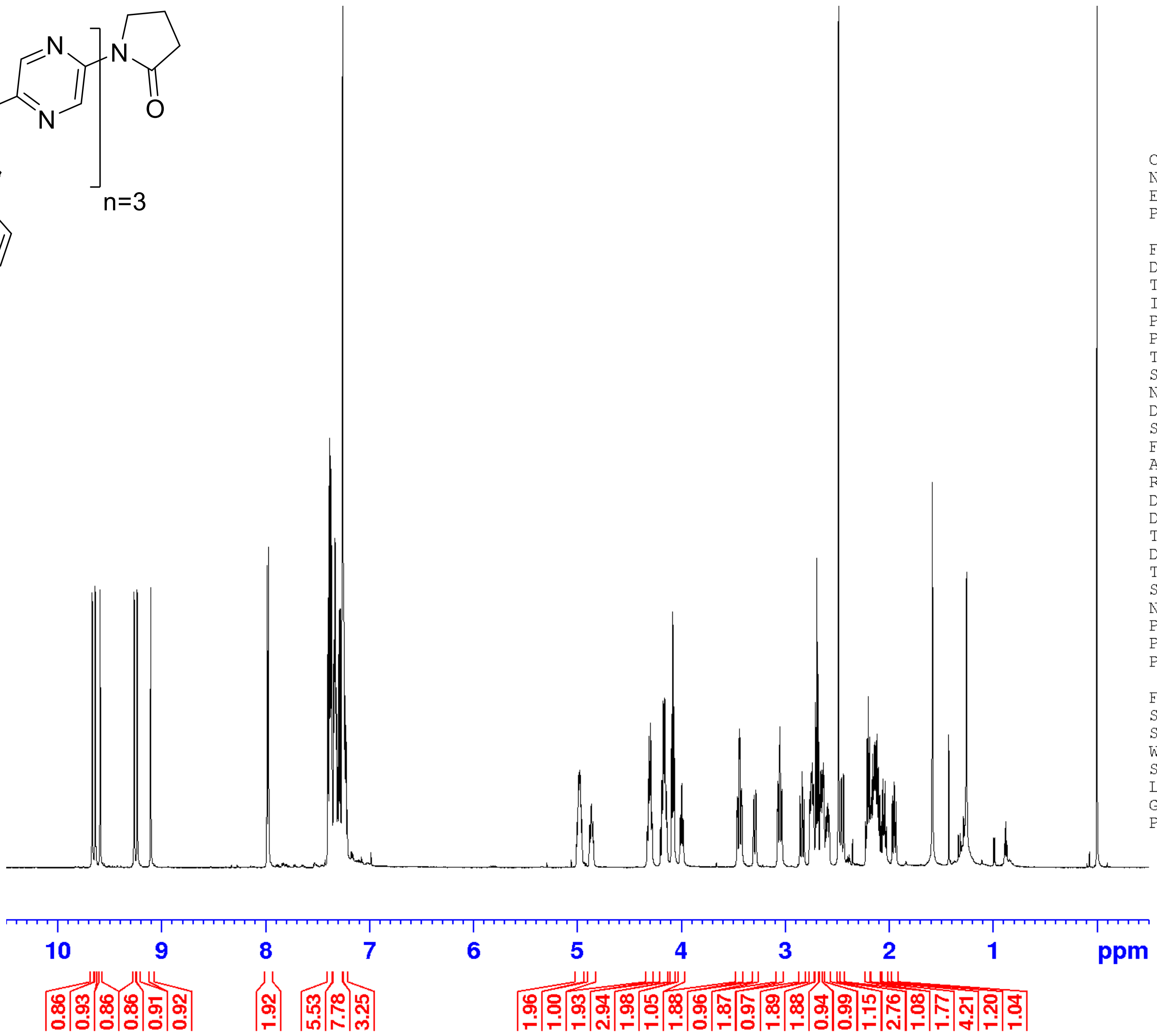


```
Current Data Parameters
NAME       WR 2.286 (600)
EXPNO                  20
PROCNO                  1

F2 - Acquisition Parameters
Date_            20220325
Time                22.50 h
INSTRUM             spect
PROBHD     Z114607_0188 (
PULPROG              zg30
TD                  65536
SOLVENT             CDCl3
NS                     16
DS                      2
SWH             12019.230 Hz
FIDRES           0.366798 Hz
AQ              2.7262976 sec
RG                  83.95
DW                 41.600 usec
DE                  12.10 usec
TE                  300.0 K
D1             1.00000000 sec
TD0                     1
SFO1          600.1337058 MHz
NUC1                   1H
P0                   3.33 usec
P1                  10.00 usec
PLW1          26.60000038 W

F2 - Processing parameters
SI                  65536
SF            600.1300145 MHz
WDW                    EM
SSB                     0
LB                   0.30 Hz
GB                      0
PC                   1.00
```

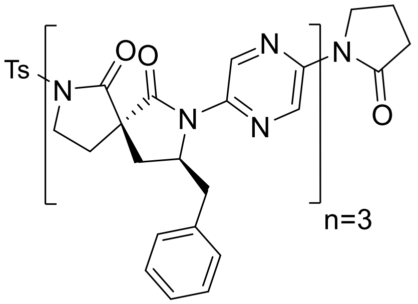

11

^{13}C NMR

151 MHz

$CDCl_3$

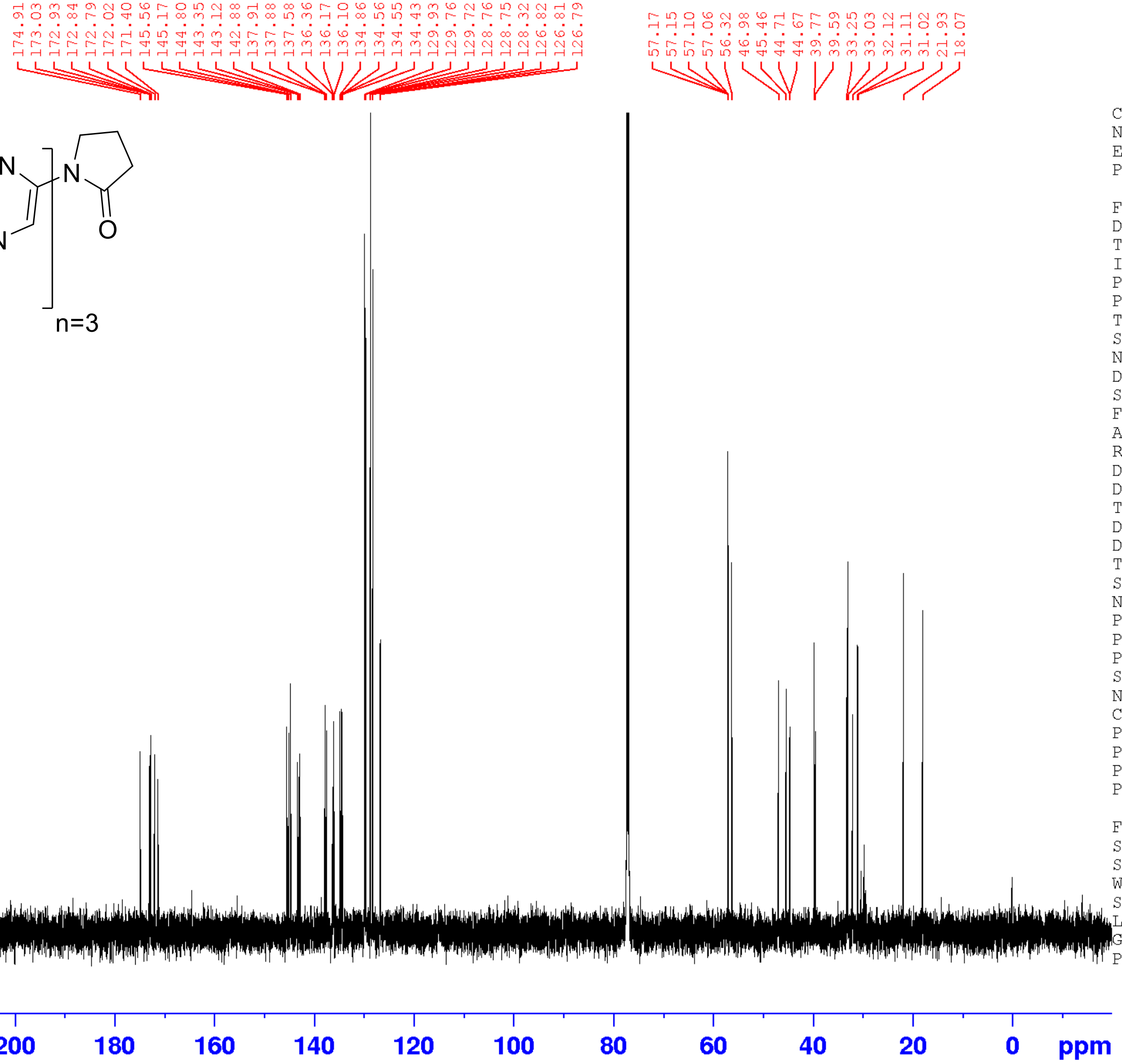


```
Current Data Parameters
NAME      WR 2.286 (600)
EXPNO                 11
PROCNO                 1

F2 - Acquisition Parameters
Date_           20220225
Time               13.08 h
INSTRUM            spect
PROBHD    Z114607_0188 (
PULPROG          zgpg30
TD                 65536
SOLVENT            CDCl3
NS                  1024
DS                     4
SWH            36231.883 Hz
FIDRES          1.105709 Hz
AQ             0.9043968 sec
RG                186.92
DW                13.800 usec
DE                  6.50 usec
TE                 300.0 K
D1            2.00000000 sec
D11           0.03000000 sec
TD0                    1
SFO1         150.9178988 MHz
NUC1                 13C
P0                  3.93 usec
P1                 11.80 usec
PLW1         85.00000000 W
SFO2         600.1324005 MHz
NUC2                  1H
CPDPRG[2         waltz65
PCPD2              70.00 usec
PLW2         27.00000000 W
PLW12         0.57327998 W
PLW13         0.28836000 W

F2 - Processing parameters
SI                 32768
SF           150.9027897 MHz
WDW                   EM
SSB                    0
LB                  1.00 Hz
GB                     0
PC                  1.40
```

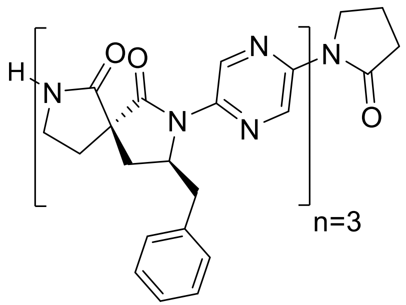

12

^{1}H NMR

600 MHz

$CDCl_3$

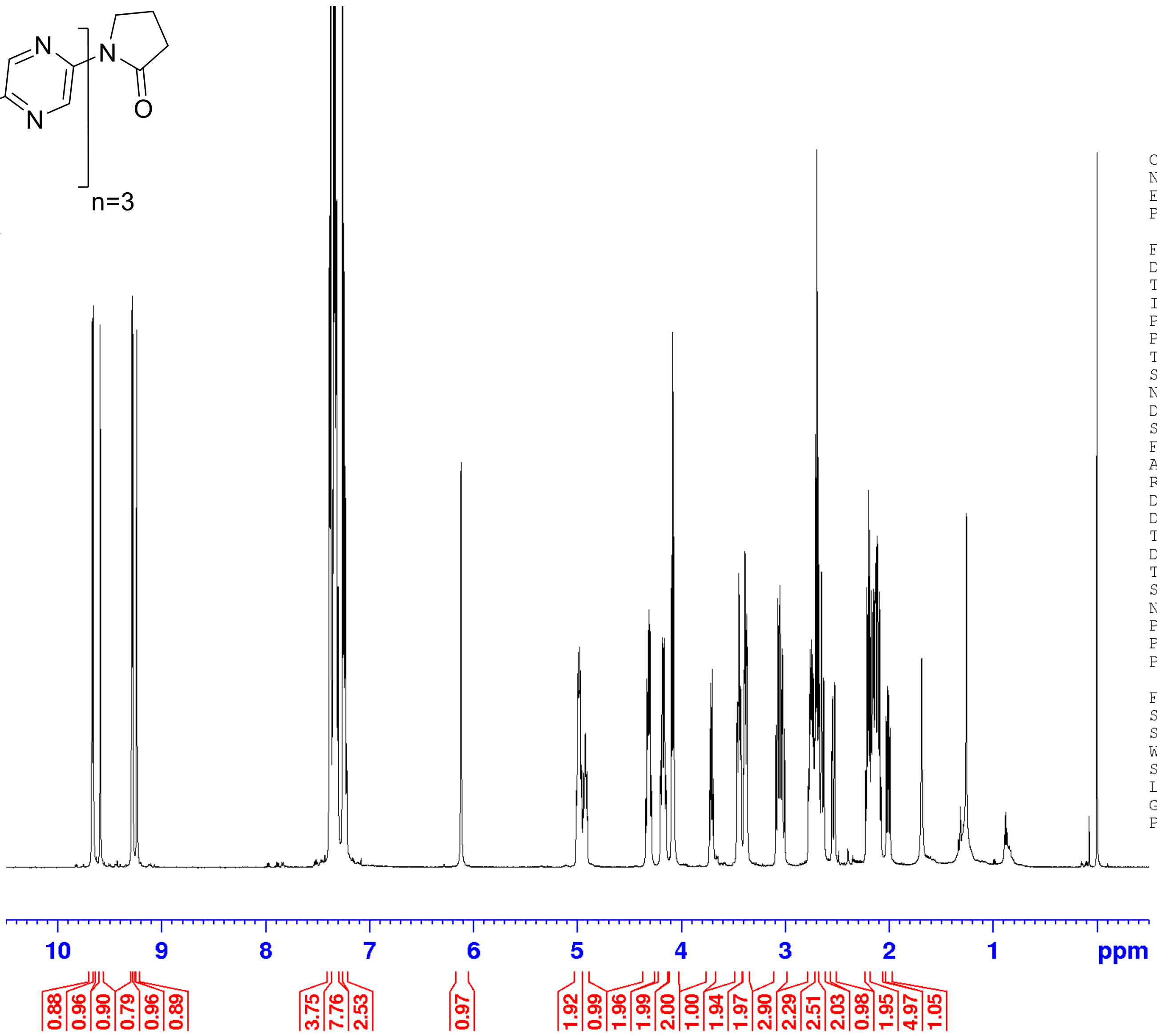


```
Current Data Parameters
NAME      WR 3.307 (600)
EXPNO                 10
PROCNO                 1

F2 - Acquisition Parameters
Date_           20220419
Time               15.27 h
INSTRUM            spect
PROBHD    Z114607_0188 (
PULPROG             zg30
TD                 65536
SOLVENT            CDCl3
NS                    16
DS                     2
SWH            12019.230 Hz
FIDRES          0.366798 Hz
AQ             2.7262976 sec
RG                 60.48
DW                41.600 usec
DE                 12.10 usec
TE                 300.0 K
D1            1.00000000 sec
TD0                    1
SFO1         600.1337058 MHz
NUC1                  1H
P0                  3.33 usec
P1                 10.00 usec
PLW1         26.60000038 W

F2 - Processing parameters
SI                 65536
SF           600.1300136 MHz
WDW                   EM
SSB                    0
LB                  0.30 Hz
GB                     0
PC                  1.00
```

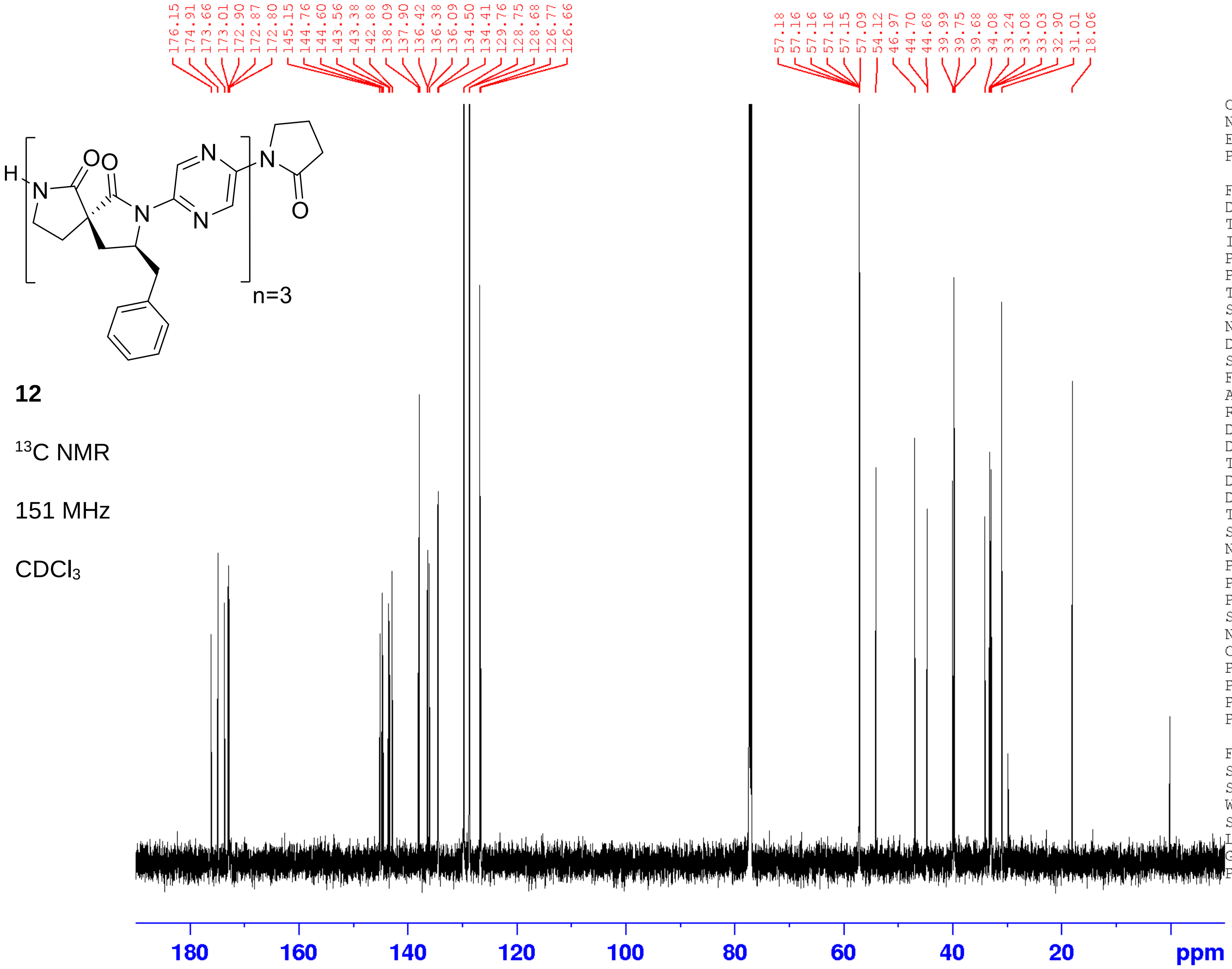

12

^{13}C NMR

151 MHz

$CDCl_3$


```
Current Data Parameters
NAME       WR 3.307 (600)
EXPNO                   11
PROCNO                   1

F2 - Acquisition Parameters
Date_             20220419
Time                 16.18 h
INSTRUM              spect
PROBHD    Z114607_0188 (
PULPROG            zgpg30
TD                   65536
SOLVENT              CDCl3
NS                    1024
DS                       4
SWH              36231.883 Hz
FIDRES            1.105709 Hz
AQ               0.9043968 sec
RG                  186.92
DW                  13.800 usec
DE                    6.50 usec
TE                   300.0 K
D1              2.00000000 sec
D11             0.03000000 sec
TD0                      1
SFO1           150.9178988 MHz
NUC1                   13C
P0                    3.93 usec
P1                   11.80 usec
PLW1           85.00000000 W
SFO2           600.1324005 MHz
NUC2                    1H
CPDPRG[2           waltz65
PCPD2                70.00 usec
PLW2           27.00000000 W
PLW12           0.57327998 W
PLW13           0.28836000 W

F2 - Processing parameters
SI                   32768
SF             150.9027917 MHz
WDW                     EM
SSB                      0
LB                    1.00 Hz
GB                       0
PC                    1.40
```

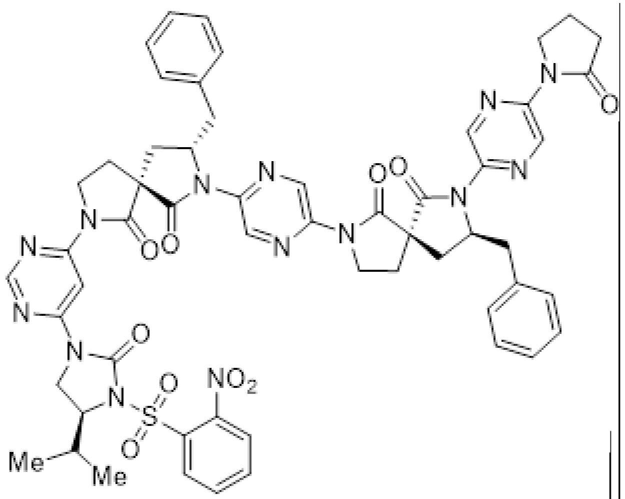

17

^{1}H NMR

600 MHz

$CDCl_3$

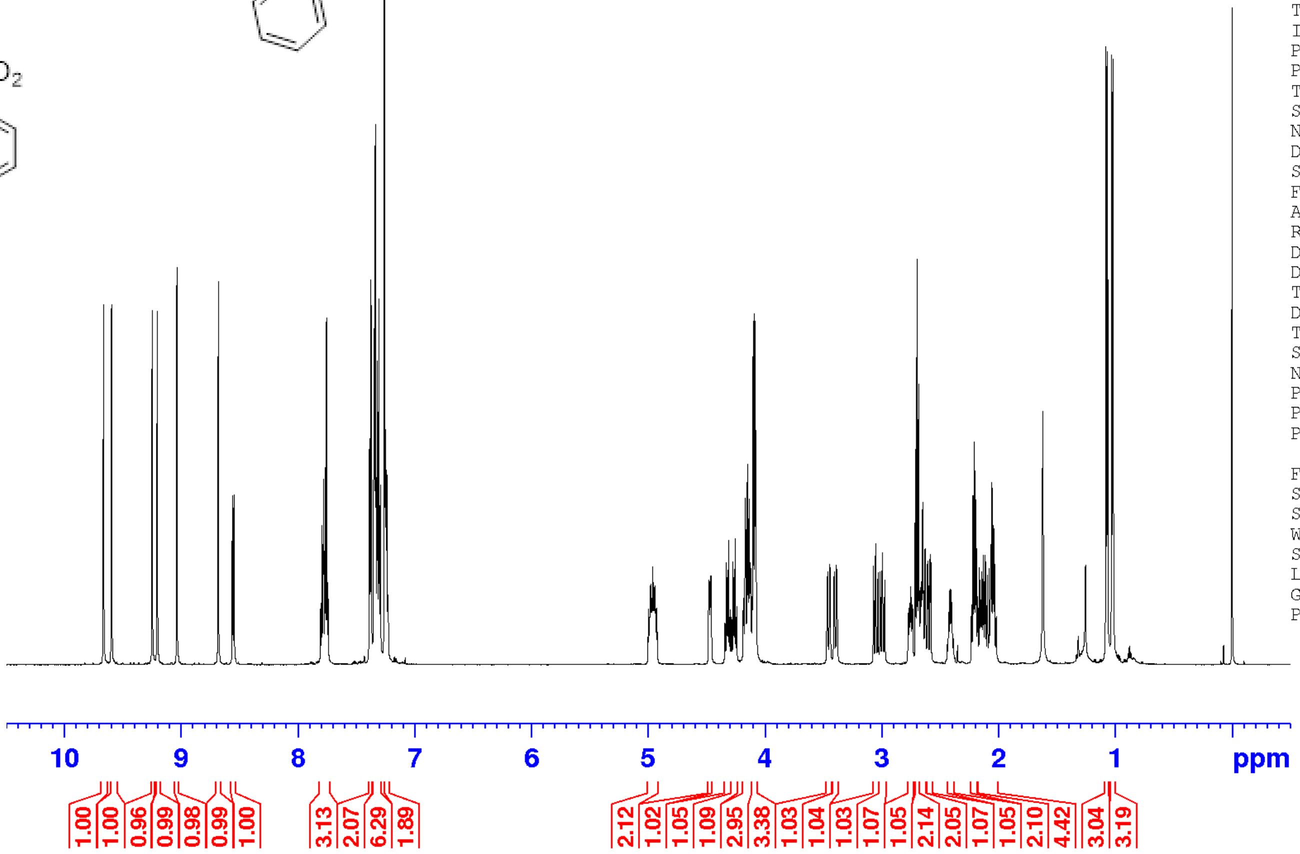


```
Current Data Parameters
NAME      WR 2.297 (600)
EXPNO                 10
PROCNO                 1

F2 - Acquisition Parameters
Date_           20220327
Time               12.34 h
INSTRUM            spect
PROBHD    Z114607_0188 (
PULPROG             zg30
TD                 65536
SOLVENT            CDCl3
NS                    16
DS                     2
SWH            12019.230 Hz
FIDRES          0.366798 Hz
AQ             2.7262976 sec
RG                 74.91
DW                41.600 usec
DE                 12.10 usec
TE                 300.0 K
D1            1.00000000 sec
TD0                    1
SFO1         600.1337058 MHz
NUC1                  1H
P0                  3.33 usec
P1                 10.00 usec
PLW1         26.60000038 W

F2 - Processing parameters
SI                 65536
SF           600.1300133 MHz
WDW                   EM
SSB                    0
LB                  0.30 Hz
GB                     0
PC                  1.00
```

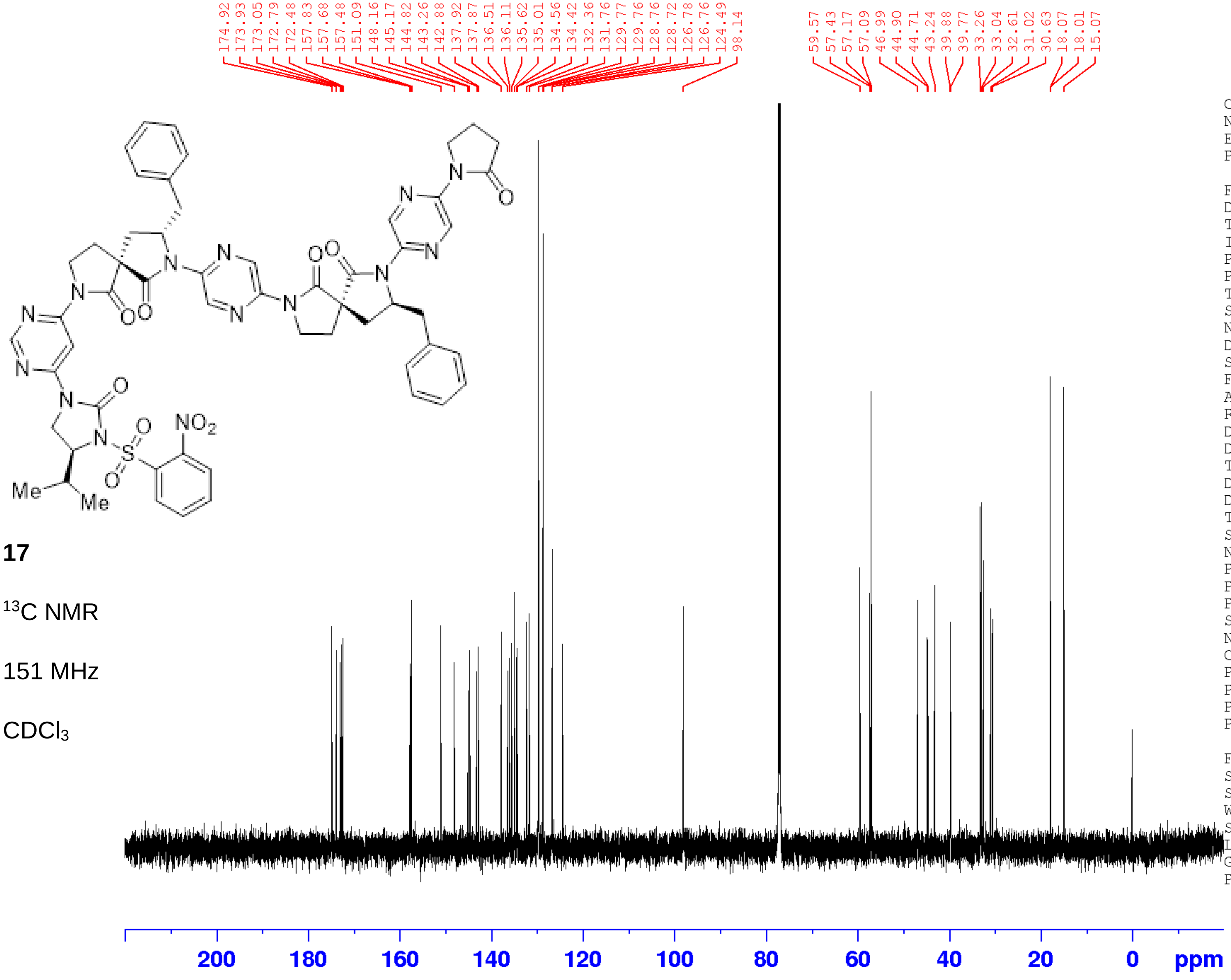

17

^{13}C NMR

151 MHz

$CDCl_3$

```
Current Data Parameters
NAME       WR 2.297 (600)
EXPNO                  11
PROCNO                  1

F2 - Acquisition Parameters
Date_            20220327
Time                13.26 h
INSTRUM             spect
PROBHD    Z114607_0188 (
PULPROG           zgpg30
TD                 65536
SOLVENT            CDCl3
NS                  1024
DS                     4
SWH           36231.883 Hz
FIDRES         1.105709 Hz
AQ            0.9043968 sec
RG               186.92
DW               13.800 usec
DE                 6.50 usec
TE                300.0 K
D1           2.00000000 sec
D11          0.03000000 sec
TD0                    1
SFO1        150.9178988 MHz
NUC1                 13C
P0                  3.93 usec
P1                 11.80 usec
PLW1        85.00000000 W
SFO2        600.1324005 MHz
NUC2                  1H
CPDPRG[2         waltz65
PCPD2              70.00 usec
PLW2        27.00000000 W
PLW12        0.57327998 W
PLW13        0.28836000 W

F2 - Processing parameters
SI                 32768
SF          150.9027902 MHz
WDW                   EM
SSB                    0
LB                  1.00 Hz
GB                     0
PC                  1.40
```

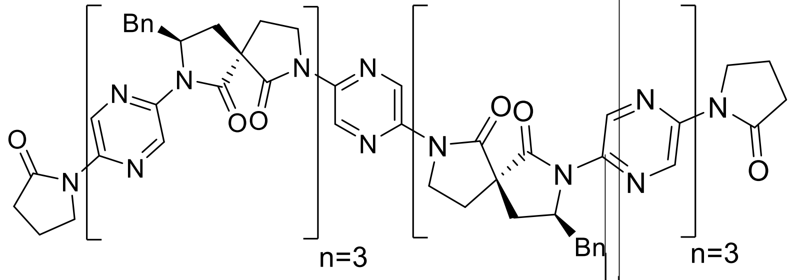

13

^{1}H NMR

600 MHz

$CDCl_3$

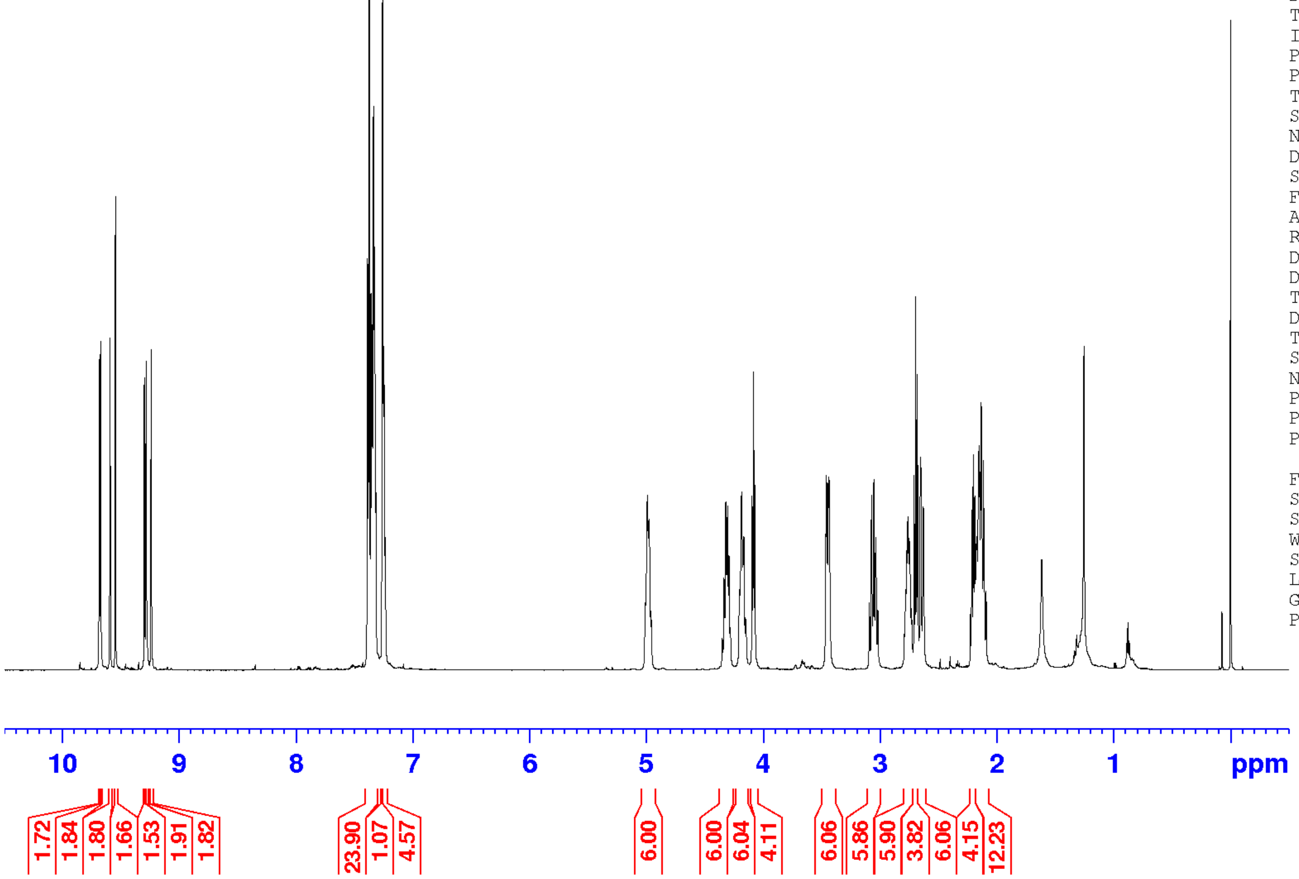


```
Current Data Parameters
NAME      WR 3.309 (600)
EXPNO                 30
PROCNO                 1

F2 - Acquisition Parameters
Date_           20220427
Time                3.00 h
INSTRUM            spect
PROBHD    Z114607_0188 (
PULPROG             zg30
TD                 65536
SOLVENT            CDCl3
NS                    64
DS                     2
SWH            12019.230 Hz
FIDRES          0.366798 Hz
AQ             2.7262976 sec
RG                 74.91
DW                41.600 usec
DE                 12.10 usec
TE                 300.0 K
D1            1.00000000 sec
TD0                    1
SFO1         600.1337058 MHz
NUC1                  1H
P0                  3.33 usec
P1                 10.00 usec
PLW1         26.60000038 W

F2 - Processing parameters
SI                 65536
SF           600.1300145 MHz
WDW                   EM
SSB                    0
LB                  0.30 Hz
GB                     0
PC                  1.00
```

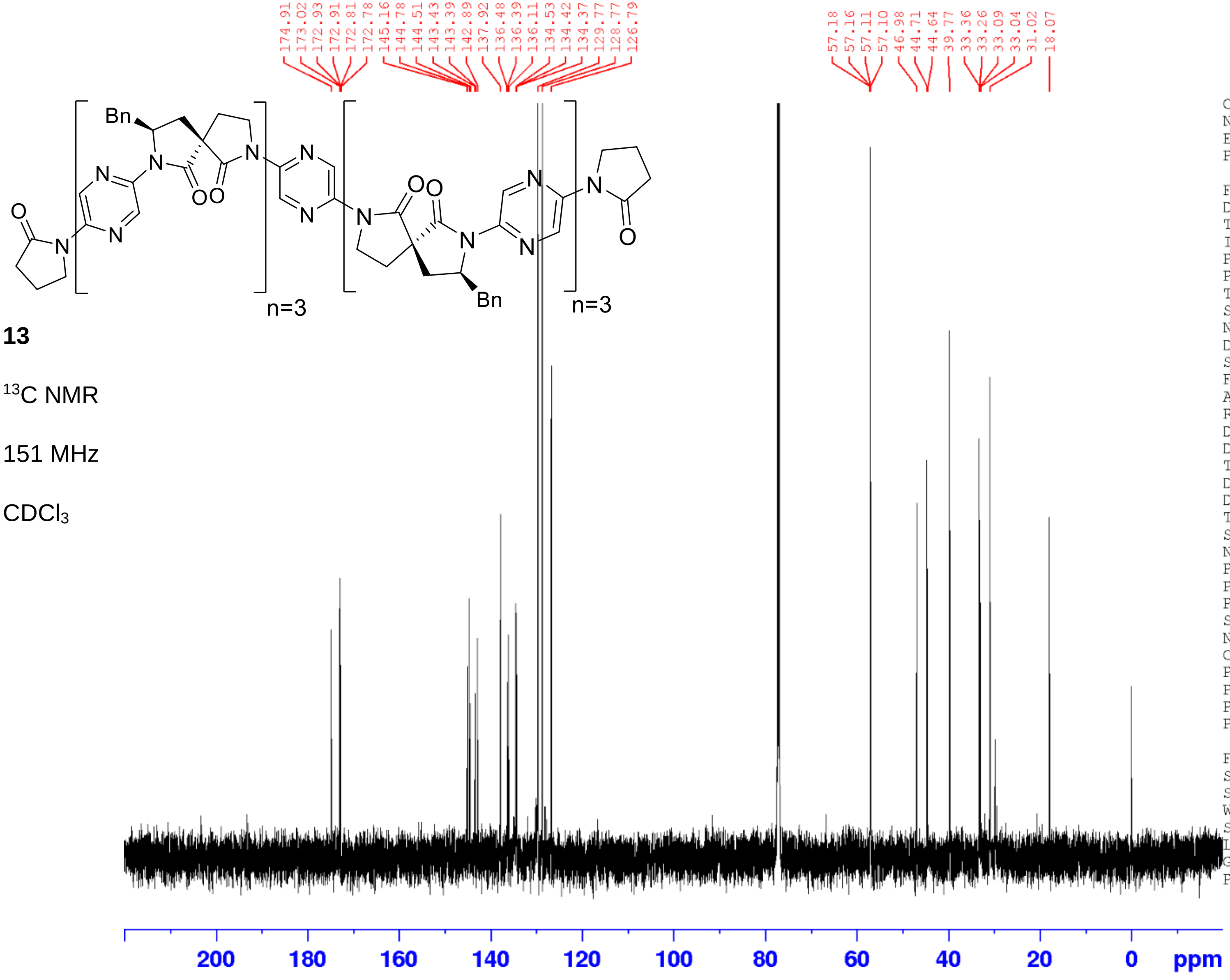

13

^{13}C NMR

151 MHz

$CDCl_3$

```
Current Data Parameters
NAME      WR 3.309 (600)
EXPNO                 11
PROCNO                 1

F2 - Acquisition Parameters
Date_           20220421
Time               18.00 h
INSTRUM            spect
PROBHD    Z114607_0188 (
PULPROG          zgpg30
TD                 65536
SOLVENT            CDCl3
NS                  1024
DS                     4
SWH            36231.883 Hz
FIDRES          1.105709 Hz
AQ             0.9043968 sec
RG                186.92
DW                13.800 usec
DE                  6.50 usec
TE                 300.0 K
D1            2.00000000 sec
D11           0.03000000 sec
TD0                    1
SFO1         150.9178988 MHz
NUC1                 13C
P0                  3.93 usec
P1                 11.80 usec
PLW1         85.00000000 W
SFO2         600.1324005 MHz
NUC2                  1H
CPDPRG[2         waltz65
PCPD2              70.00 usec
PLW2         27.00000000 W
PLW12         0.57327998 W
PLW13         0.28836000 W

F2 - Processing parameters
SI                 32768
SF           150.9027902 MHz
WDW                   EM
SSB                    0
LB                  1.00 Hz
GB                     0
PC                  1.40
```
